# Visible light activation of C–Cl and C–F bonds in persistent organic pollutants using cerium(iii) triamidoamine complex†

**DOI:** 10.1039/d5sc03626g

**Published:** 2025-07-23

**Authors:** Adrien Combourieu, Stella Christodoulou, Laurent Maron, Eachann Assendjee, Nicolas Casaretto, Bich Tuyen Phung, Akos Banyasz, Olivier Maury, Matthew Gregson, Ashley J. Wooles, Stephen T. Liddle, Cédric Tard, Grégory Nocton, Grégory Danoun

**Affiliations:** a Laboratoire de Chimie Moléculaire, CNRS, UMR 9168, Ecole Polytechnique, Institut Polytechnique de Paris Route de Saclay Palaiseau Cedex 91128 France gregory.danoun@polytechnique.edu; b Laboratoire de Physique et Chimie des Nano-Objets, CNRS, INSA, Université Paul Sabatier 31077 Toulouse France; c CNRS, ENS de Lyon, LCH, UMR 5182 F-69342 Lyon France; d Department of Chemistry, The University of Manchester Oxford Road Manchester M13 9PL UK

## Abstract

Procedures for activating and degrading compounds containing carbon–halogen bonds are highly sought after due to the environmental persistence and potential hazards of such compounds. Such activations are challenging because of the high stability of these bonds, particularly those with C–F bonds. Here, we report on the activation of carbon–halogen bonds, including C–F bonds, by the cerium(iii)-triamidoamine complex Ce^III^TREN^TIPS^ (1, TREN^TIPS^ = tris-(2-(tri-iso-propylsilylamidoethyl)amine)). Under light irradiation, 1 reaches a strongly negative excited state redox potential, and our measurements enable it to be estimated as −3.2 V relative to Cp_2_Fe^0/+^. Hence, the photo-reactivity of 1 with carbon–halogen bonds has been established with numerous examples, including Persistent Organic Pollutants (POPs) and fluorinated compounds. The photoactivation of POPs is rapid, but the photoactive nature of the cerium(iv) products precludes complete conversion. This study provides insight into the activation of POPs that may benefit the future design of photodegradation approaches for these highly problematic compounds.

## Abstract 2

Herein, we report the synthesis of a Ce(iii)-based complex, [Ce^III^TREN^TIPS^], possessing a redox potential of −3.2 V *vs.* Cp_2_Fe^0/+^ under light irradiation which enables C–Cl and C–F bond activation on very stable chemicals including POPs and PFAS.

## Graphical abstract

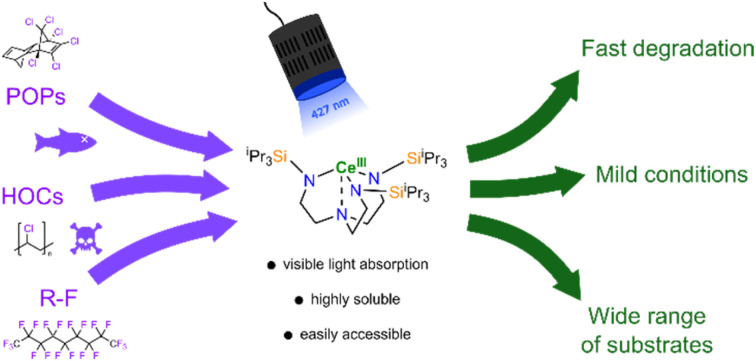

## Introduction

**Fig. 1:**
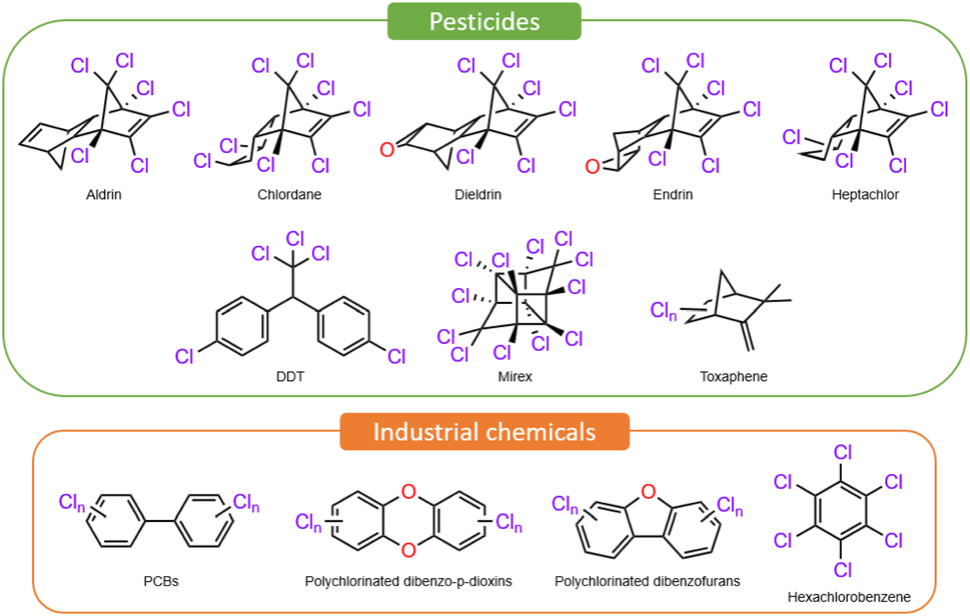
Examples of chlorinated POPs compounds.

Halogenated organic compounds (HOCs) have been utilized in various applications, ranging from flame retardants (brominated compounds) to pesticides and dielectrics (chlorinated substances), including anti-adhesive materials (perfluorinated molecules). However, HOCs exhibit many adverse health and environmental effects, as reported as early as the 1980s, due to their lipophilicity, which tends to facilitate their accumulation in the fatty tissues of living organisms and their resistance to biological degradation.^1^ For this reason, these compounds have been classified as Persistent Organic Pollutants (POPs), Fig. 1, which are currently of significant environmental and toxicological concern. Although these substances were mainly introduced into nature by anthropogenic activities, especially for food production after World War II, and have been banned in many countries since the mid-1970s due to their toxicity, their persistence and bioaccumulation in the environment still cause real environmental and toxicity concerns.^2,3^ The Stockholm Convention on POPs, adopted in 2001, established global mandates for the reduction and elimination of these chemicals, necessitating efficient and clean methods.^4^ Several treatments have been developed, including bioremediation,^5^ photochemical oxidation using reactive oxygen species,^6,7^ and electrochemical oxidation.^8^ However, reduction methods are less studied, primarily due to the very low reduction potentials of C–Cl or even C–F bonds (*E*^red^ < −2.5 V *vs.* Fc/Fc^+^). Thus, for example, recent studies have demonstrated the degradation of perfluorooctanoic acid through a redox-neutral process including a successive decarboxylative step,^9^ though a magnesium(i) complex enables reductive defluorination of poly(tetrafluoroethylene),^10^ which is a highly challenging substrate to activate.^11^

Divalent lanthanide complexes are well known to induce a single reductive process,^12,13^ allowing the activation of many difficult-to-activate substrates, including dinitrogen^14–17^ and carbon monoxide.^18,19^ However, these divalent lanthanides must be prepared and used stoichiometrically. Recently, methodologies using light as a trigger have been developed to generate *in situ* divalent lanthanides from trivalent complexes bearing suitable photosensitisers.^20–22^ The photochemical properties of Sm, Yb, and Eu can also enhance the reducing potential of their divalent form, leading to C–halide bond activation reactions.^23^ Cerium is also well-suited to light-induced processes,^24^ since it is one of the few lanthanide ions that can easily oxidize to the +IV oxidation state. While recent highlights of the light-induced reactivity of Ce^IV^ complexes have sparked growing interest in the community,^25–27^ fewer studies have investigated the photochemistry of Ce^III^, which possesses unique spectroscopic features involving an inter-configurational f-to-d transition in which energy can be modulated by the ligand carried by the cerium ion to, for example, activate inert chlorinated substrates, including aryl chlorides and polyvinyl chloride (PVC).^28,29^

The reductive potential of Ce^III^ is intimately dependent on the electronic nature of its coordination sphere, where, for example, the redox potential can vary from −2.2 V to −2.9 V *vs.* Cp_2_Fe^0/+^ for Ce^III^ supported by amide and/or guanidinate ligands.^30^ On the other hand, the use of even simple chloride ligands in the hexachlorocerate [Ce^III^Cl_6_]^3−^ trianion^28^ achieves a high excited state redox potential of approximately −3.45 V *vs.* Cp_2_Fe^0/+^, thereby facilitating the challenging reductive activation of aryl chlorides. Despite its strong reducing character, [Ce^III^Cl_6_]^3−^ possesses drawbacks, since its solubility is primarily restricted to very polar solvents, which can interfere with the reactivity of radical intermediates and present solubility issues with hydrophobic POPs, and it requires excitation in the energetic UV range (∼330 nm) compared to amide/guanidinate derivatives (∼420 nm).

Based on the above observations, considering the utility of tripodal ligand classes,^31–36^ we decided to investigate triamidoamine Ce^III^ complexes, which would, in principle, allow very low excited state redox potentials but avoid the abovementioned drawbacks. Here, we report the synthesis and characterization of a cerium(iii)-TREN^TIPS^ complex and, with and without light irradiation, its exceptional reactivity and selectivity toward halide substrates, including highly challenging C–F bonds, POPs, and PFAS. This work develops (photo)catalytic cerium-based reactivity with robust substrates, thus advancing the agenda for destroying environmentally persistent pollutants.

## Results and discussion

### Syntheses and structural analysis

**Fig. 2:**
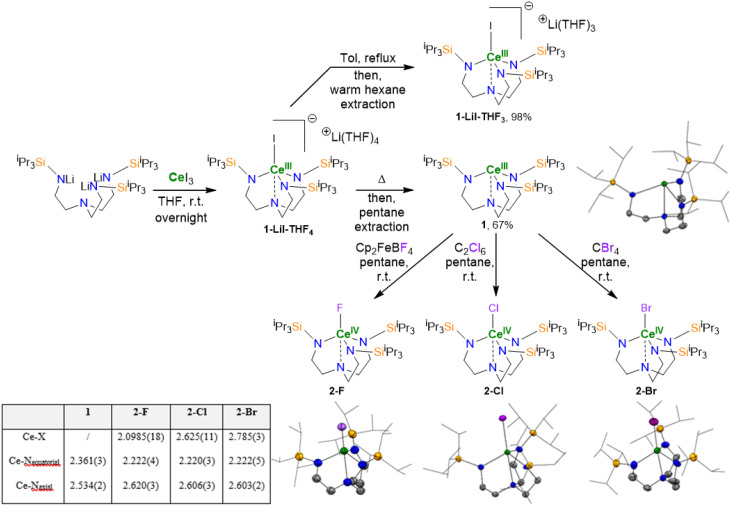
Synthesis of 1-LiI-THF_4_, 1-LiI-THF_3_, 1, 2-F, 2-Cl, and 2-Br and X-ray crystal structures of 1, 2-Br, 2-Cl, and 2-F with displacement ellipsoids at the 50% probability level (except for the ^i^Pr groups depicted in wireframe) recorded at 150 K. H atoms have been omitted for clarity. C atoms are in grey, N atoms are in blue, Si atoms are in orange, Ce atom is in green and F, Cl and Br atoms are in purple. X-ray crystal structures of 1-LiI-THF4 and 1-LiI-THF3 are omitted and can be found in ESI.†

The complex [Ce^III^TREN^TIPS^] (1) was synthesized by reacting previously reported^34^ Li_3_TREN^TIPS^ with CeI_3_ in stoichiometric amounts in THF overnight under an inert atmosphere. Upon extraction and subsequent crystallization in pentane, the LiI ‘ate’ complex of [Ce^III^TREN^TIPS^] (1-LiI-THF_4_) could be isolated. The base-free complex 1 was obtained by heating complex 1-LiI-THF_4_ at 60 °C under reduced pressure overnight to remove the stabilizing THF molecules. The resulting yellow solid was then extracted with pentane and crystallized to afford 1 in 67% yield from Li_3_TREN^TIPS^. The isolation of 1*vs.*1-LiI-THF_4_ is intimately dependent upon the work-up conditions. Furthermore, the contact ion pair complex 1-LiI-THF_3_ can also be obtained when the work-up conditions are varied, where it can be intuitively recognized that removal of Li-coordinated THF is key to the eventual isolation of 1 (Fig. 2). Unfortunately, although 1-LiI-THF_3_ could be isolated in high-yield, attempts to oxidize it led to intractable products, so we focused on 1. Complex 1 was oxidized to the corresponding Ce^IV^ derivative, 2-X (X = F, Cl, Br), using various halide-based oxidants. Thus, adding CBr_4_ to a solution of 1 in pentane led to an immediate change of color from yellow to purple, indicating the oxidation of the cerium center as observed by Scott on the [Ce^III^TREN^TBDMS^], which corresponds to the formation of 2-Br.^35^ A similar change in color could also be observed when 1 reacted with C_2_Cl_6_ or ferrocenium tetrafluoroborate (Cp_2_FeBF_4_),^37^ leading to the corresponding 2-Cl and 2-F, respectively. As expected, XRD analysis of each complex (2-F, 2-Cl, 2-Br) demonstrates the usefulness of the bulkier TIPS group compared to the TBDMS one, because the complexes were only observed in their monomeric form, with no trace of the formation of the mixed valent dimer observed by Scott.^35^ Structural analysis of 1 and 2-X (X = Br, Cl, F) reveals a contraction of the tripodal ligand around the cerium center upon oxidation, which is influenced by the coordination of the apical position of the cerium atom and by its oxidation state. A shortening of the Ce–N_equatorial_ distances in 1 and 1-LiI-THF_4_ was observed from 2.361(3) Å for 1 to 2.273(2) Å for 1-LiI-THF_4_, which is even shorter on the Ce^IV^ complexes (average of 2.221(2) Å for 2-Br, 2-Cl and 2-F). This contraction phenomenon was also observed on the Ce–N_axial_ bond distances at 2.534(2) Å for 1 and 2.664(5) Å for 1-LiI-THF_4_. However, 2-Br, 2-Cl, and 2-F showed a shorter distance of this bond (average of 2.61(2) Å for 2-Br, 2-Cl, and 2-F). This variation showcases the adaptability of the TREN moiety to reorganize around the cerium center easily during redox processes. This behavior is especially important for catalysis because it minimizes a significant energy barrier for the ligand reorganization during the catalytic processes.^38^ A typical dependence of the length of the Ce–halide bond towards the size of the halide was observed (2.785(3) Å for 2-Br, 2.625(11) Å for 2-Cl and 2.099(2) Å for 2-F). As expected, the distances are shorter than the ones described by Scott on the mixed valent dimer (3.115(5) Å for Ce–Br–Ce, 3.001(3) Å for Ce–Cl–Ce).^35^ The Ce–I bond in 1-LiI-THF_4_ was found to be longer than that reported by Scott with [ICe^IV^TREN^TBDMS^], which is likely due to the formal anionic charge in the former and TREN ligand sterics (3.128(6) Å for [ICe^IV^TREN^TBDMS^] *vs.* 3.2580(5) Å for 1-LiI-THF_4_). Interestingly, an elongation of the Ce–I bond in 1-LiI-THF_4_ (3.2580(5) Å) and in 1-LiI-THF_3_ (3.2810(4) Å) could be observed which is in accordance with a stepwise synthetic pathway in order to reach 1.

### Solution studies

^1^H NMR analysis of 1 showed a paramagnetic behavior, as expected for a Ce^III^ complex. The effective magnetic moment of 1-LiI-THF_3_ measured by the Evans method in C_6_D_6_ was *μ*_eff_ = 2.58 *μ*_B_, in good agreement with an f^1^ complex. The room-temperature value agreed with the solid-state magnetic data of 1 (Fig. S45†). Because the complex possesses an effective *C*_3v_ symmetry, the spectrum is relatively simple, with four signals corresponding to each equivalent groups of protons. The most upfield shifted paramagnetic signal at −17.1 ppm was attributed to the CH_2_ of the TREN scaffold in the α-position of the silylamide group owing to its proximity to the magnetic cerium center. In 2-X (X = F, Cl, Br), the ^1^H NMR spectra are diamagnetic, consistent with the presence of a Ce^IV^ center. The influence of the halogen atom coordinated with the cerium atom could be observed at the signal corresponding to the CH_2_ in the α-position of the silylamide group with a 2-Br signal at 5.14 ppm, a 2-Cl signal at 5.06 ppm and a 2-F signal at 4.68 ppm.

Complex 1 was studied by cyclic voltammetry to determine its redox potentials in the ground and excited states. The experiments were performed in THF, a polar solvent which could coordinate the cerium center and where 1 was stable. Other typical solvents used in electrochemistry, such as acetonitrile or DMF, react with 1, mainly due to the basicity of the amide group, which deprotonates the solvent. The choice of adapted supporting electrolytes was also critical for the same reason. Fluoride-based electrolytes, such as *n*-tetrabutylammonium tetrafluoroborate (TBABF_4_), reacted with 1, and tetraphenylborate salts TBABPh_4_ proved challenging due to their low solubility in THF. Thus, the *n*-tetrabutylammonium trifluoromethanesulfonimidate (TBATFSI, 0.1 M) was identified as the best supporting electrolyte, allowing the redox potential of 1 to be estimated at *E*_1/2_ = −0.50 V *vs.* Cp_2_Fe^0/+^ (Fig. S60†).1
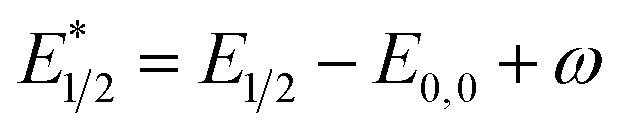


Using the Rehm–Weller formalism, eqn (1),^39^ the excited-state reduction potential 
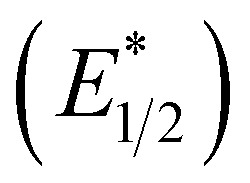
 was estimated at −3.2 V *vs.* Cp_2_Fe^0/+^ where 
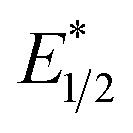
 is the excited state half-wave redox potential, *E*_0,0_ is the difference in energy between the zeroth vibrational state of the ground state and the first excited state (approximated by the intersection of the fluorescence excitation and emission spectra, 21 552 cm^−1^ here), and *ω* the work function, which is usually neglected in similar cases (see ESI†).

**Fig. 3:**
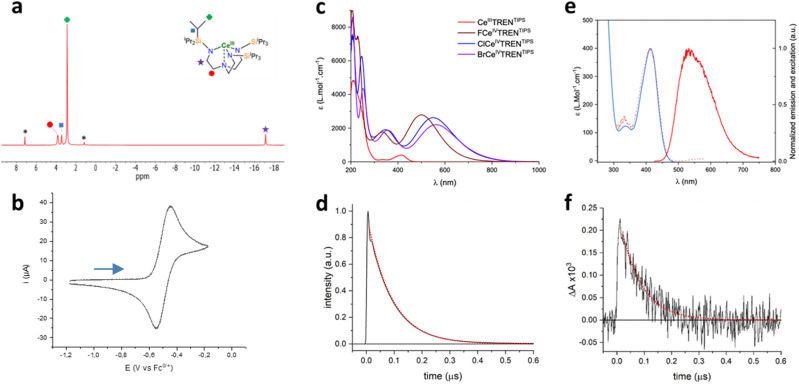
Characterization of 1 in solution. (a) ^1^H NMR of 1 in C_6_D_6_, * are for C_6_H_6_ residue and pentane. (b) Cyclic voltammetry in THF (1 at 4 mM, TBATFSI, 0.1 M, *v* = 50 mV s^−1^) of 1, (Fc = Cp_2_Fe). (c) Absorption spectra of 1 (5 × 10^−4^ M), 2-F (10^−4^ M), 2-Cl (10^−4^ M), and 2-Br (10^−4^ M) in pentane. (d) Photoluminescence decay of 1 obtained at 550 at 400 nm, respectively, upon 354 nm excitation. The dotted red line represents the results of the mono-exponential fit. (e) Emission (*λ*_ex_ = 400 nm) and excitation (*λ*_em_ = 550 nm) spectra of 1 at 298 K in pentane at 10^−6^ mol L^−1^. (f) Transient absorption decay of 1 obtained at 550 nm, respectively, upon 354 nm excitation. The dotted red line represents the results of the mono-exponential fit.

The absorption spectrum of a diluted yellow solution of 1 in thoroughly degassed pentane exhibits a broad transition centered around 415 nm with *ε* ≈ 400 M^−1^ cm^−1^ and a full width at half maxima of 63 nm (3675 cm^−1^). Using TD-DFT calculations, this band was assigned to a metal-to-metal excitation of f–d type. Deconvolution of the absorption spectrum (Fig. S47†) indicates the presence of high energy transition at 379 and 335 nm, assigned to ligand-to-metal charge transfer (LMCT) from the amido-ligand to the 5d_*z*^2^_ orbital of the Ce^III^ ion. Upon excitation at 415 nm, an intense green emission at *ca.* 532 nm is observed with a quantum yield of 34% (*vs.* coumarin 153) and a lifetime of 88 ns (Fig. S58†). The excitation spectrum of 1 revealed perfect overlap with the absorption spectrum. The Stokes shift of 5350 cm^−1^ is very large, indicating substantial rearrangement in the excited state. All these values are comparable to those obtained for related Ce^III^ amido- or guanidinato-reported in the literature.^40–42^ Interestingly, the emission band is broad with a shoulder at lower energy, nicely resolved into two separated transitions at low temperature (77 K) in MeTHF (Fig. S56†). These two transitions can be assigned to Ce^III^-based 5d → 4f emission from the ^2^D excited state to the two ground levels: ^2^F_5/2_ and ^2^F_7/2_. The energy difference between the two contributions is 2220 cm^−1^ in MeTHF at 77 K, which aligns with the splitting of the two 4f ground levels of Ce^III^.^43,44^ Transient absorption spectroscopy (TAS) in nanosecond regime was also performed at room temperature. Between 500–800 nm, no TAS signal was detected. In contrast, at 400 nm, a transient absorption decay was detected with a lifetime of 77 ns (Fig. 3f). The similarity between the TAS and luminescence lifetimes indicates that the same ^2^D excited state is responsible for the excited-state absorption and luminescence phenomena. Finally, these results strongly suggest that the ^2^D state is the lowest energy excited state responsible for the photo-induced reactivity.

**Fig. 4:**
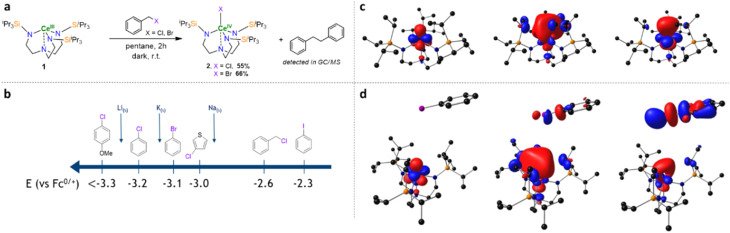
Reactivity and computational studies of 1. (a) Reactivity of 1 with benzyl chloride and bromide without light irradiation. (b) Redox scale of given alkyl- and aryl halides with Li, Na, and K given to provide additional context.^45,46^ (c) Left to right: SOMO, LUMO, and LUMO+1 orbitals of 1 complex and TDDFT of the transition at 400 nm. Hydrogen atoms are omitted for clarity. (d) Computational studies of the 1 adduct with the chloride aryl substrate implicated in the transition at 400 nm, left to right SOMO, LUMO+3, and LUMO+7.

The electronic structure of 1 was investigated at the DFT level (B3PW91 functional). The ground state is found, as expected, to be a doublet spin state with the unpaired spin density located at the Ce center. The unpaired electron is located in an f_*δ*_ orbital, as evidenced by the nature of the SOMO in Fig. 4c. The absorption spectrum of 1 was also simulated using TDDFT methods. The main features of the experimental spectrum are nicely reproduced, that is, the primary absorption in the UV part and a weaker absorption in the 400 nm range. According to the TDDFT, this transition is mainly described by SOMO to LUMO and LUMO+1 transitions, which are primarily 4f-to-5d transitions.

### Reactivity studies

The reactivity of 1 was tested on different halogenated substrates to evaluate the redox potential of this complex. Benzyl chloride and bromide were tested and appeared reactive at room temperature without any light stimulus in pentane. A change of color from yellow to purple took place, in good agreement with the evolution observed upon chemical oxidation of 1, resulting in the formation of 2-Cl or 2-Br, respectively. This oxidation occurs through a SET process, which leads to the formation of a benzyl radical, ultimately 1,2-diphenylethane, which was observed in GC-MS as the result of the radical homocoupling. Both 2-Cl and 2-Br were obtained in powder form from these reactions in 55% and 66% yields, respectively.

**Fig. 5:**
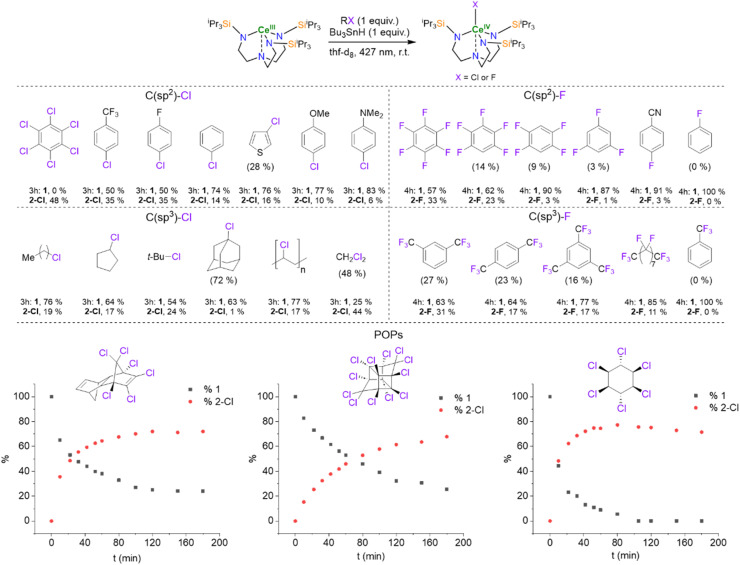
Scope of the substrates activated by 1. The reactions were performed in 20 μmol scale using 1,3,5-trimethoxybenzene as internal standard. Conversion of the substrates are indicated in brackets and the yields of 1 and 2-Cl or 2-F are indicated below the corresponding molecules.

Substrates that are more challenging to reduce (Fig. 5), such as aryl chloride, were also tested, but no reaction occurred in C_6_D_6_ without a light stimulus. However, under 427 nm light irradiation, the characteristic yellow-to-purple change in color was observed, demonstrating the enhanced reductive potential of 1 under light irradiation. The ^1^H NMR showed the formation of 2-Cl, while the GC-MS analysis confirmed the formation of the coupling product between the aryl radical and the deuterated benzene. A radical clock experiment was performed using 2-allyloxychlorobenzene in THF-d_8_ with 2 equiv. of dihydroanthracene (DHA) under 427 nm light irradiation (see ESI†). The formation of the product resulting from the 5-exo radical cyclization was confirmed by ^1^H-NMR and GC-MS analysis of the crude material. Additionally, using a specific radical trapping agent such as Bu_3_SnH also led to the formation of Bu_6_Sn_2,_ supporting the formation of a radical intermediate. This light-induced reductive SET process was tested on various halogenated products in the presence of Bu_3_SnH, first to support the presence of radical formation for each tested substrate and, second, to quench the C-centered radical that could react with 1 and deactivate it.

To evaluate the extent of the scope of substrates that can be activated by 1, reactions were followed up during the first hours using a variety of substrates to report the consumption of 1 and the formation of 2-Cl (Fig. S83†). Complex 1 proved to be active under 427 nm light irradiation, with a large panel of aryl chloride possessing electron-withdrawing groups, such as –CF_3_ (35% of 2-Cl in 3 h), or even with electron donating group, such as –F (13% of 2-Cl in 3 h),^47^ –OMe (10% of 2-Cl in 3 h) or –NMe_2_ (6% of 2-Cl in 3 h) (Fig. 5). By monitoring the kinetic evolutions of the different ArCl, we noted that the proto-dehalogenation reaction was more favorable when an electronically deficient ArCl was used. On the contrary, the aryl chloride-carrying electron-donating group showed a slow conversion even after 3 h. This finding is in agreement with the theory of the outer-sphere electron transfer (see ESI†), which relates the kinetic constant of the redox reaction with the relative redox potential of the substrates and, therefore, by the electron-withdrawing or donating nature of the group in the para position.^48^

DFT calculations were also used to understand the photochemical transfer of chloride from chlorobenzene to complex 1. The formation of a stable weak adduct was found computationally (Fig. 4d), where the chlorine points toward the cerium center. Thus, TDDFT calculations were carried out on this adduct to check how this weak coordination modifies the main features of the absorption spectrum of 1. It is interesting to note that the peaks remain more or less at the same position, in line with weak coordination, but the intensity is increased in the visible region. The transition around 400 nm, which is a f-to-d transition in 1, now appears as arising from the singly occupied cerium 4f orbital to orbitals mainly located at the chlorobenzene (Fig. 4d). Even more interestingly, the chlorobenzene orbital in LUMO+7 (as well as in LUMO+3 but to a lesser extent) displays significant C–Cl antibonding character, nicely accounting for the experimentally observed C–Cl bond breaking and Ce–Cl bond formation upon photoexcitation. Surprisingly, it appears that 1 is not completely converted into 2-Cl during the photochemical process since the addition of the amount of 1 and 2-Cl decreased over time, which correlates with a decrease in the reactivity of 1. This behavior originates from the instability of 2-Cl under light irradiation over time, since irradiation of 2-Cl at 427 nm leads to its degradation, whereas it is stable for days without light irradiation. This light sensitivity can be explained by a light-induced homolysis process of the Ce–Cl bond, which leads to the formation of chlorine radicals and the regeneration of 1, as described in the literature.^29^ This chlorine radical would then react with 1 or 2-Cl through a radical process (*e.g.* HAT process, *etc.*), leading to their degradation. This hypothesis is supported by the formation of 1 under irradiation and the presence of non-negligible amounts of H_3_TREN^TIPS^, which would be formed by protonolysis with any generated HCl. However, this photo-induced regeneration of 1 was inefficient since, after 5 h of irradiation, 11% of 1 was formed while 70% of 2-Cl was consumed (Fig. S69†). This behavior would partially distort the quantification of 1 and 2-Cl, leading to an underestimation of the conversion. However, for several substrates, the conversion of the halogenated starting materials was determined by ^1^H NMR (Fig. 5) when their characteristics signals were not overlapping with others signals.

Given the results on aryl chlorides, we sought to extend the scope to primary, secondary, and tertiary alkyl chlorides and polychlorinated substrates, including polymer (PVC) and POPs. Pleasingly, these challenging and important substrates were also found to react with 1 under light irradiation, especially with POPs, with high conversion in only a few hours. For instance, persistent insecticides such as aldrin, mirex, or lindane reached >60% dechlorination after less than 60 min, 2 h or 20 min, respectively.

**Scheme 1:**
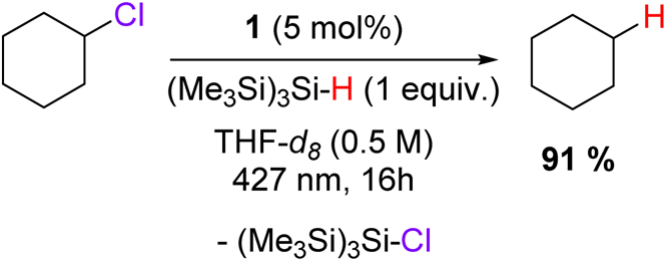
Catalytic dechlorination of cyclohexyl chloride. The reactions were performed in 0.25 mmol scale using 1,3,5-trimethoxybenzene as internal standard. The yield was determined by ^1^H NMR.

Given the scope of activation of chlorinated derivatives, the photochemical reduction of highly challenging fluorinated substrates was also examined. Different fluorinated arenes were tested in the same conditions as the chlorinated substrates *i.e.* in the presence of 1 equivalent of Bu_3_SnH in THF-d_8_ under 427 nm irradiation. Remarkably, 1 is able to activate C(sp^2^)–F as well as C(sp^3^)–F bonds on the activated substrates (C_6_F_6_, C_6_F_5_H, C_6_F_4_H_2_, (CF_3_)_3_C_6_H_3_) or less activated fluorinated compounds (C_6_F_3_H_3_, FC_6_H_4_CN, 1,3-(CF_3_)_2_C_6_H_4_ and 1,4-(CF_3_)_2_C_6_H_4_). However, non-activated fluorinated aryls such as C_6_H_5_F and C_6_H_5_CF_3_ were not activated by 1, even after a prolonged irradiation. It is worth noting that multiple defluorination processes occurred at similar rates during the process, as observed by the formation of multiple unidentified side products in ^19^F NMR analysis of the crude reaction mixtures and by comparison between the rates of the consumption of the fluorinated starting materials and the production of 2-F. However, the formation of 2-F could clearly be identified and followed by ^1^H NMR analysis as being the dominant pathway, providing informative data regarding the C–F bond activation. As expected, the electronic nature of the fluoro-aryl has a marked influence on the reduction reaction. Perfluorobenzene (33% of 2-F in 4 h) was significantly faster than pentafluorobenzene (23% of 2-F in 4 h), tetrafluorobenzene (3% of 2-F in 4 h) and trifluorobenzene (1% of 2-F in 4 h) while fluorobenzene was completely untouched. However, with extended irradiation, chemically relevant consumption of these difficult fluorinated substrates could be reached, such as 23% for C_6_F_4_H in 12 h and 8% for C_6_F_3_H_3_ in 27 h. This electronic tendency was not suitable for the trifluoromethylated aryl substrates. Indeed, either 1,3- or 1,4-bis-trifluoromethylbenzene were consumed at a similar rate (≈25% of conversion), while the consumption of 1,3,5-tris-trifluoromethylbenzene was slower (16%). As for fluorinated benzene substrates, increasing the irradiation time allowed them to reach good conversions (≈50% in 27 h). Finally, perfluorononane, a representative example of a PFAS, was also tested, and we were pleased to observe the activation of this substrate by 1 (13% in 4 h). However, similar to 2-Cl, 2-F was not stable under 427 nm irradiation over a long period, leading to degradation products similar to 2-Cl, particularly to H_3_TREN^TIPS^ (Fig. S69†). The fate and potential use in catalysis of the highly reactive fluorine radical from the Ce–F bond homolysis is currently under investigation and will be reported in due course. In the reaction presented above, the Bu_3_Sn–H bond was homolytically cleaved, and the resulting Bu_3_Sn˙ dimerized to give Bu_6_Sn_2_. No chlorinated or fluorinated tin species were detected indicating no possible regeneration of 1 after halide abstraction. In order to proceed with this regeneration and perform catalytic transformations, other hydride donors such as silane derivatives were tested. As such (Me_3_Si)_3_SiH has proven effective and allowed the catalytic conversion of chloro-cyclohexane into cyclohexane using 5 mol% of 1 in 0.5 M THF-d_8_ solution (Scheme 1). Unfortunately, these conditions were not directly applicable for polychlorinated substrates such as POPs, and other hydride donors are currently under investigation.

## Conclusions

In summary, we synthesized and characterized a series of Ce^III^- and Ce^IV^-based complexes bearing the TREN^TIPS^ ligand. The redox potential of [Ce^III^TREN^TIPS^] (1) (−0.50 V *vs.* Cp_2_Fe^0/+^) allowed SET to several halogenated substrates. Moreover, unlike the widely used [Ce^III^Cl_6_]^3−^, 1 was also found to induce reductive SET in nonpolar solvents such as benzene or pentane. Thanks to the peculiar photophysical properties of 1, its redox potential can be significantly lowered (−3.2 V *vs.* Cp_2_Fe^0/+^) under visible light irradiation (427 nm). As a result, an extended scope of chlorinated and fluorinated substrates, including highly challenging polyhalogenated substrates, was successfully activated. The oxidized complexes formed in the SET process with halide substrates, 2-Cl or 2-F, also possessed photo-induced reactivities, probably originating from Ce–Cl and Ce–F bond homolysis, and are currently under investigation. The striking result presented in this article concerns the rapid and successful light-induced activation of polyhalogenated molecules, such as POPs, which cause environmental and toxicity concerns. Three popular persistent insecticides were successfully degraded. These promising initial activation results on anthropogenic POPs and PFAS compounds of major environmental concern significantly advances Ce^III^ complexes as potential candidates for developing photo-degradation technologies for molecules that are highly problematic in the environment, and may benefit the future design of photodegradation approaches for these highly problematic compounds.

## Author contributions

A. C. carried out all experiments and analyzed the data with the help of E. A. M. G., A. J. W., and S. T. L. developed the synthesis and characterization of 1-LiI-THF_3_ and in parallel 1. L. M. and S. C. carried out the computational data. N. C. and A. C. measured and analyzed the X-ray data. A. C. and B. T. P. carried out the catalytic tests with hydrosilanes derivatives. A. C. and C. T. worked out the electrochemistry measurements. A. B. and O. M. performed the photophysical measurements. G. N. and G. D. designed and supervised the project and analyzed and interpreted all the data. G. D. wrote the manuscript with contributions from all authors.

## Conflicts of interest

There are no conflicts to declare.

## Supplementary Material

SC-OLF-D5SC03626G-s001

SC-OLF-D5SC03626G-s002

SC-OLF-D5SC03626G-s003

## Data Availability

The data supporting this article have been included as part of the ESI,† including detailed experimental procedures and characterization data. Crystal data for 1-LiI-THF_4_, 1-LiI-THF_3_, 1, 2-F, 2-Cl and 2-Br are available from the Cambridge Crystallographic Data Center under CCDC: 2387121, 2387122, 2387384, 2387125, 2387124, 2387123. Computational output is available on request.
