# Supplementary material for: Visible light activation of C–Cl and C–F bonds in persistent organic pollutants using cerium(iii) triamidoamine complex
Source: Chem Sci. 2025 Jul 23;16(34):15510–7. doi: 10.1039/d5sc03626g (PMC12306684; doi:10.1039/d5sc03626g)
Supplement: SC-016-D5SC03626G-s002 [file SC-016-D5SC03626G-s002.pdf]

Supplementary Information (SI) for Chemical Science.
This journal is © The Royal Society of Chemistry 2025

Visible Light Activation of C-Cl and C-F bonds in Persistent Organic Pollutants using Cerium(III) Triamidoamine Complex

Adrien Combourieu,[a] Stella Christodoulou,[b] Laurent Maron,[b,*] Eachann Assendjee,[a] Nicolas Casaretto,[a] Bich Tuyen Phung,[a] Akos Banyasz,[c] Olivier Maury,[c] Matthew Gregson,[d] Ashley J. Wooles,[d] Stephen T. Liddle,[d] Cédric Tard,[a] Grégory Nocton,[a,*] and Grégory Danoun[a,*]

[a.] *Laboratoire de Chimie Moléculaire, CNRS UMR 9168, Ecole Polytechnique, Institut Polytechnique de Paris, Route de Saclay, 91128, Palaiseau Cedex, France.*
[b.] *Laboratoire de Physique et Chimie des Nano-Objets, CNRS & INSA, Université Paul Sabatier, 31077 Toulouse, France.*
[c.] *CNRS, ENS de Lyon, LCH, UMR 5182, F-69342 Lyon, France.*
[d.] *Department of Chemistry, The University of Manchester, Oxford Road, Manchester, M13 9PL, UK.*
Gregory.nocton@polytechnique.edu, Gregory.danoun@polytechnique.edu

Table des matières

I. Synthetic procedures 7
a) Synthesis of [Li_3TREN^{TIPS}] 7
b) Synthesis of [$Li(THF)_4$][$ICeTREN^{TIPS}$] (1-LiI-THF_4) 7
c) Synthesis of [$Li(THF)_3$][$ICeTREN^{TIPS}$] (1-LiI-THF_3) 7
d) Synthesis of [$Ce^{III}TREN^{TIPS}$] (1) 8
e) Synthesis of [$ClCe^{IV}TREN^{TIPS}$] (2-Cl) 8
f) Synthesis of [$BrCe^{IV}TREN^{TIPS}$] (2-Br) 9
g) Synthesis of [$FCe^{IV}TREN^{TIPS}$] (2-F) 9
h) Synthesis of 1-allyloxy-2-chlorobenzene 10
i) Reactivity with chlorinated substrates 10
j) Reactivity with fluorinated substrates 10
II. Spectral data 11
a) Spectral data for Li_3TREN^{TIPS} 11
b) Spectral data for [$Li(THF)_4$][$ICeTREN^{TIPS}$] (1-LiI-THF_4) 13
c) Spectral data for [$Li(THF)_3$][$ICeTREN^{TIPS}$] (1-LiI-THF_3) 15
d) Spectral data for [$Ce^{III}TREN^{TIPS}$] (1) 16
e) Spectral data for [$ClCe^{IV}TREN^{TIPS}$] (2-Cl) 22
f) Spectral data for [$BrCe^{IV}TREN^{TIPS}$] (2-Br) 25
g) Spectral data for [$FCe^{IV}TREN^{TIPS}$] (2-F) 28
i) Spectra of 1-allyloxy-2-chlorobenzene 32
III. X-Ray crystal structures 33
IV. Magnetic measurements 44
V. EPR measurements for Chapter I 45
VI. UV-visible and fluorescence spectra 46

a) Experimental data....46
VII. Electrochemistry....55
a) Cyclic voltammetry measurements....55
b) Estimation of the work function ωf....57
VIII. Reactivity....58
a) Photochemical degradation of [ClCe^{IV}TREN^{TIPS}] (2-Cl)....58
b) Radical clock experiment....61
c) Reactivity with CH_2Cl_2....63
d) Reactivity with chlorinated substrates....66
1) NMR spectra....72
e) Reactivity with fluorinated substrates....80
1) Kinetic data....80
2) NMR spectra....86
3) Photochemical degradation of [FCe^{IV}TREN^{TIPS}] (2-F)....94
IX. Computational details....97
X. References....122

General methods and materials

General methods :

All reactions were performed using standard Schlenk-line techniques under dry N_2 or gloveboxes (MBraun) under N_2 atmosphere with concentrations of O_2 and H_2O lower than 1 ppm. Glassware was dried at 110 °C overnight before use.

Solvents were dried either using either CaH_2 (MeCN, CH_2Cl_2) or sodium, using benzophenone as an indicator (pentane, benzene, THF, C_6D_6, THF-d_8, toluene-d_8), degassed at least thrice using freeze-pump-thaw techniques and distilled under reduced pressure. Deuterated solvents were purchased from Eurisotop and purified similarly to non-deuterated solvents.

All reagents have been distilled on CaH_2 and degassed, or sublimated and before use.

$Ce(OTf)_3$ and $CeCl_3$ were bought from Strem Chemicals and used without purification. CeI_3 was provided by Alfa Aesar and used as provided.

NMR spectroscopy :

NMR spectra were recorded on a Bruker Avance III-300 MHz spectrometer using standard 5 mm NMR tubes with J. Young valves. Chemical shifts are given in parts per million (ppm), and referenced to residual solvent residual peaks or internal reference. Spectra were analysed using MestreNova software, with a systematic baseline correction for integration.

Without any specification, all reactions depicted in NMR spectra are equimolar.

Gas chromatography separation, mass spectrometry and flame ionization detector characterization :

GC-MS and GC-FID were respectively carried out with a Varian 450-GC equipped with Varian 240-MS and a Bruker Scion 436-GC equipped with a split mode, capillary injection system, and flame ionization detector, using an SGE apolar IDBP5 (15 m x 0.25 mm x 0.25 µm) column and helium as carrier gas.

Gaseous substrates were analysed using an Inficon Micro GC fusion.

UV-visible, infrared spectroscopies and luminescence measurements :

UV-visible measurements were carried out in a quartz cuvette bearing a J. Young tape (see Figure S2a) on an Agilent Technologies Carry 60 UV equipped with a Xenon flash lamp (80 Hz) in double beam mode at 300 nm/min from 200 nm to 1000 nm with a baseline correction, using pentane as solvent. Spectra were plotted in wavenumber, fitted with fityk[1] using gaussian functions, and plotted again in wavelength.

Fluorescence spectra were recorded on a Horiba Fluoromax-4 using a 150 W CW ozone-free xenon lamp, from 250 nm to 800 nm with 2 nm for entrance and exit slits at 1 nm/s.

Transient Absorption Spectroscopy measurements were done using a Magnitude envision spectrometer.

Electrochemical measurements :

Cyclic voltammetry experiments were recorded using a Metrohm Autolab PGSTAT302N potentiometer, and the data were processed using OriginLab 2019 software. The experiments were performed inside an N_{2-F}illed glovebox.

The electrochemical cell consisted of a homemade 5-necks 5 mL cell, with a silver wire as quasi-reference electrode, a glassy carbon disk (3 mm diameter) as working electrode and a platinum wire as counter-electrode. The solution used for electrochemical analysis was 4 mM in analyte and 100 mM in electrolyte (*n*-tetrabutylammonium bis-trifluoromethanesulfonimidate, TBATFSI) in 5 mL of THF.

All data were collected in a positive-feedback IR compensation mode. At the end of the experiment, ferrocene was added as an internal standard for calibration. The potential of the analyte was reported *versus* ferrocene (noted Fc).

EPR measurements:

EPR spectrum were recorded either on a Jeol JES-X310 spectrometer working in X-band with a modulation frequency of 0.01 mT or on a Bruker ESP 500E using a standard rectangular (4102ST) EPR cavity (Bruker ER4131VT). Otherwise mentioned, data have been recorded at 273 K

EPR spectrum of **1** was recorded by Eric Rivière on a Bruker ELEXSYS 500 spectrometer equipped with a Bruker ER 4116DM X band resonator, an Oxford Instruments continuous flow ESR 900 cryostat, and an ITC 503 temperature control system. The conditions used were the following: microwave frequency = 9.636 GHz, microwave power = 4.0 mW, modulation amplitude = 8 Gauss, modulation frequency = 100 KHz, Gain = 50 dB, and temperature = 10 K

EPR data were simulated using EasySpin program in MatLab R2024a, using a homemade program provided in appendix. All atoms included in the simulations were weighted with their natural abundancy, and spectra were simulated using “garlic” sub-program. Line broadening was evaluated using an average of Lorentzian and Gaussian broadenings. Final optimizations of the simulations were done using least-square fitting “esfit” subprogram, with Levenberg-Marquardt algorithm, with optimisation target as “data as if” and scaling set to “maxabs”. Data were exported and plotted using Origin 2019 64 bits.

Elemental analyses :

Elemental analysis samples were analysed by Mikroanalytisches Labor Pascher in double determination C, H, N under an inert atmosphere.

X-Ray Diffraction measurements :

Single crystal diffraction measurements were carried on a STOE Stadivari diffractometer using a Mo source (λ_{K_α} = 0.710 Å) and an EIGER 2 detector. The crystal was kept at 150 K during data collection using an Oxford Instruments cryostat. Using Olex2[18], the structures were solved with the SHELXT[19,20] structure solution program using Intrinsic Phasing and refined with the SHELXL refinement package using Least Squares minimization.

All structures are represented with displacement ellipsoids are represented at 50% probability, and hydrogen atoms are omitted for clarity. Isopropyl groups are displayed in wireframe style for improved reading of the structure.

All atoms in structures based on TREN^{TIPS} ligand are labelled in a similar way (see **Figure S45** for the labelling in Ce^{III}TREN^{TIPS}).

Magnetism measurements :

Magnetic susceptibility data were collected on a powdered sample of the compound with a SQUID-based sample magnetometer Quantum design MPMS5.

Photochemistry setup :

Light irradiations were carried out using Kessil lamps (https://kessil.com/products/science_main.php).

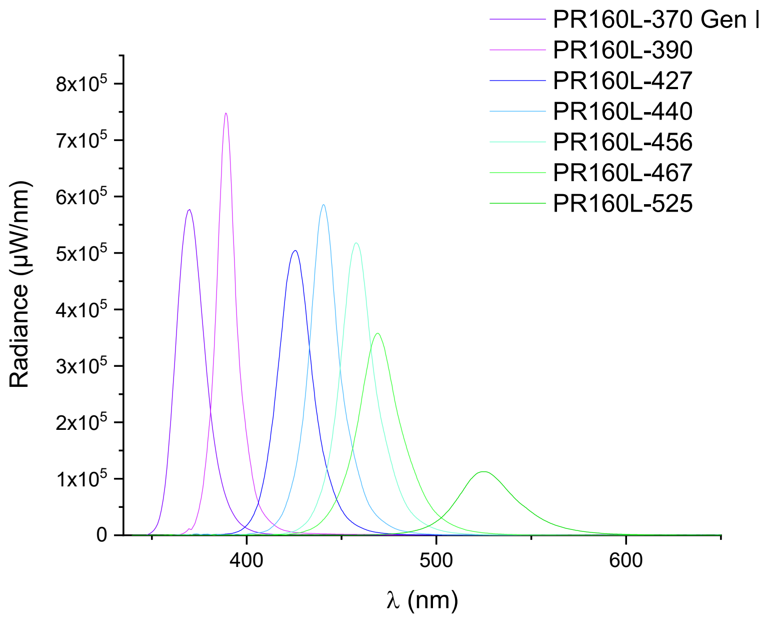

Figure S1. Emission spectra of Kessil photochemistry lamps. Provided with courtesy by Kessil©

Photochemistry experiments were carried out using a homemade 3D-printed box (see **Figure S2b**) designed by Olivier Bec and Joseph Youssef, in which both NMR tubes could be irradiated.

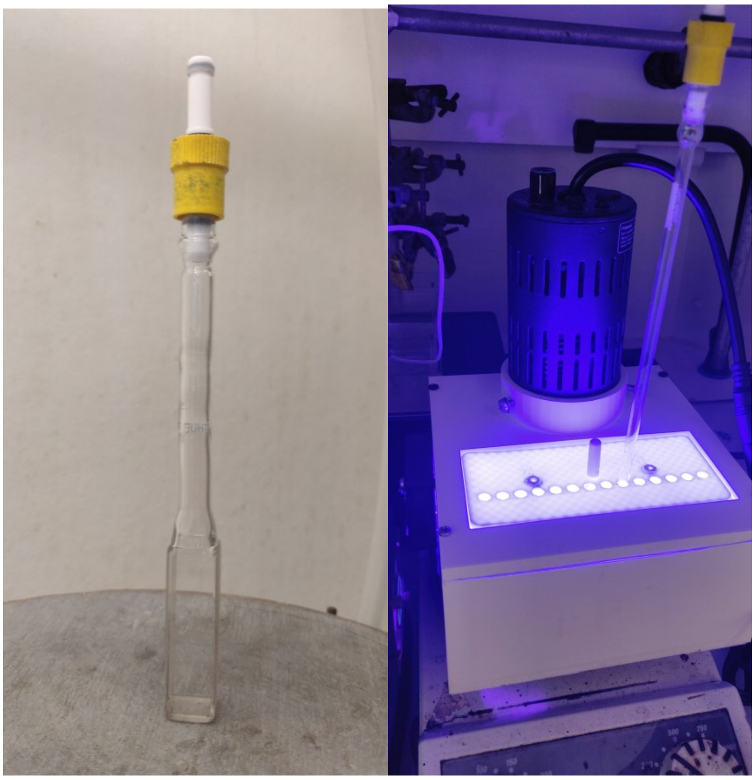

Figure S2. a) J. Young quartz cuvette used for UV-vis measurements (left). b) Homemade 3D-printed photochemistry box used for irradiation experiments (right).

Concerning the synthesis of [Li(THF)$_3$][ICeTREN^{TIPS}]: Solvents were dried by passage through activated alumina towers, degassed, and stored over either activated molecular sieves or potassium mirrors. ^{1}H and $^{7}Li\{^{1}H\}$ NMR spectra were recorded on a JEOL JNM-ECZ 400 MHz spectrometer operating at 399.78 MHz and 155.37 MHz respectively. Chemical shifts are quoted in ppm and are relative to tetramethylsilane (^{1}H) or 1M LiCl in D_2O (7Li). FTIR spectra were recorded on a Bruker Alpha spectrometer with a Platinum-ATR module in the glovebox. UV/Vis/NIR spectra were recorded on a Perkin Elmer Lambda 1050 spectrometer where data were collected in 4 mm path length cuvettes and were run versus the appropriate reference solvent. Elemental microanalyses were carried out by Mr. Martin Jennings at the Micro Analytical Laboratory, Department of Chemistry, University of Manchester.

I. Synthetic procedures

a) Synthesis of [Li_3TREN^{TIPS}]

Li_3Tren^{TIPS} was prepared in a similar procedure as previously described in the literature (https://doi.org/10.1107/S0021889810030499)

In a 250 mL Schlenk flask containing tris-(2-aminoethyl)amine (12.2 g, 83 mmol, 1 equiv.) was added 200 mL of dry THF. The mixture was stirred for 5 minutes and cooled down to -78°C. ^{n}BuLi (100 mL, 250 mmol, 3 equiv., 2.5 M in hexanes) was added dropwise over 15 minutes, and the mixture was left warming up to room temperature for 6 h. Without purification, the reaction medium was cooled to -78°C, and ClSi^{i}Pr$_3$ (53.5 mL, 250 mmol, 3 equiv.) was added dropwise over 10 minutes. The reaction medium gradually turned yellow, and a white solid appeared. The mixture was left warming overnight (approximately 14 h). Volatiles were removed under reduced pressure, and the reaction medium was filtered on a porosity 3 fritted filter to remove LiCl precipitate, then on the Teflon® filter to remove any remaining solid. 40 mL of dry pentane was added to the yellow liquid (to prevent solidification upon further cooling) and cooled down to -78 °C. ^{n}BuLi (100 mL, 250 mmol, 3 equiv., 2.5 M in hexanes) was added dropwise over 15 minutes, yielding a white solid, and the mixture was left warming up to room temperature overnight. A white solid was recovered on a fritted filter and rinsed using cold pentane, yielding 39.4 g of white powder (η = 74%).

^{1}H NMR (300 MHz, C_6D_6, 298 K): δ 3.09 (m, 6H, C**H$_2$**CH$_2$Si^{i}Pr$_3$), 2.25 (m, 6H, CH$_2$C**H$_2$**Si^{i}Pr$_3$), 1.16 (m, 63H, CH$_2$CH$_2$Si^i**Pr$_3$**)

^{13}C NMR (300 MHz, C_6D_6, 298 K): δ 57.3 (**C**H$_2$), 43.2 (**C**H$_2$), 19.8 (CH(**C**H$_2$)$_2$), 12.6 (**C**H(CH$_2$)$_2$).

FTIR ν (cm^{-1}) (powder): 2931 (s), 2883 (s), 2858 (s), 2774 (m), 1460 (vs), 1362 (s), 1341 (s), 1270 (s), 1073 (s), 1055 (s), 1027 (s), 1005 (s), 991 (vs), 963 (vs), 933 (vs), 878 (vs), 777 (vs), 658 (vs), 638 (vs)

b) Synthesis of [Li(THF)$_4$][ICeTREN^{TIPS}] (1-LiI-THF$_4$)

To a suspension of CeI_3 (2.00 g, 3.8 mmol, 1 eq.) in 30 mL of THF was added a solution of Li_3TREN^{TIPS} (2.43 g, 3.8 mmol, 1 eq.) in 25 mL of THF. The supernatant became readily yellow, and the mixture was left under stirring overnight. Volatiles were removed under reduced pressure (2 evaporation with pentane), extracted with pentane until the supernatant was colourless, filtered using a Teflon® filter, and set to cool at -40 °C in about 40 mL of pentane. After 30 minutes, a large amount of yellowish precipitate appeared, and after removal of the supernatant, the yellowish-beige solid (5.865 g), corresponding to [Li(THF)$_4$][ICe^{III}TREN^{TIPS}] (**1-LiI-THF$_4$**) was obtained.

c) Synthesis of [Li(THF)$_3$][ICeTREN^{TIPS}] (1-LiI-THF$_3$)

THF (20 ml) was added to a precooled (–78 °C) mixture of [CeI$_3$(THF)$_4$][22] (5.67 g, 7 mmol) and [TREN^{TIPS}Li$_3$] (4.64g, 7 mmol). The resulting colourless solution was allowed to warm to room temperature with stirring over 72 h to afford a dichroic green/red solution. Volatiles were removed under reduced pressure and the resulting yellow solid was boiled briefly in toluene (20 mL), then volatiles were removed *in vacuo* to afford a yellow powder, which was extracted into warm hexanes (30 mL) and the solvent reduced in volume (10 ml) and stored at room temperature to afford yellow crystals of [Li(THF)$_3$][ICeTREN^{TIPS}] (**1-LiI-THF$_3$**). Yield: 7.59 g, 98%. Attempts to oxidise **1-LiI-THF$_3$** (e.g. 0.5 I_2, AgBPh$_4$, FcOTf) led to the formation of intractable products.

^{1}H NMR (C_6D_6, 298K) δ 3.56 (9H, s, C**H**(CH$_3$)$_3$), 3.37 (54H, s, CH(C**H$_3$**)$_3$), 2.90 (6H, s, C**H$_2$**), 2.81 (12H, s, OC**H$_2$**CH$_2$), 1.02 (12H, s, OCH$_2$C**H$_2$**), –14.15 (6H, s, C**H$_2$**) ppm.

^{7}Li{^{1}H} NMR (C_6D_6, 298K) δ 2.32 (br, s) ppm.

$^{29}Si\{^1H\}$ NMR (C_6D_6, 298K) no resonances observed.

FTIR ν (cm^{-1}) (powder): 2955 (m), 2934 (m), 2881 (m), 2854 (s), 2831 (m), 1459 (s), 1259 (m), 1074 (m), 1058 (w), 1041 (s), 1030 (m), 1007 (m), 987 (w), 931 (s), 881 (s), 805 (w), 738 (s), 668 (s), 623 (m), 562 (w), 542 (w), 507 (s), 440 (m), 418 (m),

UV-vis (THF) λ_{max} (ε/$mol^{-1}cm^{-1}$): 408 (282), 272 (285) nm.

Elemental analysis for $C_{45}H_{99}CeILiN_4O_3Si_3$, Found (Theoretical) C, 49.02; H, 9.05; N, 5.08 %. Found: C, 48.77 (49.02); H, 9.30 (9.05); N,4.91 (5.08)

Magnetic moment (Evans method, C_6D_6, 298 K) μ_{eff} = 2.58 μ_B.

d) Synthesis of [$Ce^{III}TREN^{TIPS}$] (1)

To a suspension of CeI_3 (2.00 g, 3.8 mmol, 1 eq.) in 30 mL of THF was added a solution of Li_3TREN^{TIPS} (2.43 g, 3.8 mmol, 1 eq.) in 25 mL of THF. The supernatant became readily yellow, and the mixture was left under stirring overnight. Volatiles were removed under reduced pressure (2 evaporation with pentane), extracted with pentane until the supernatant was colourless, filtered using a Teflon® filter, and set to cool at -40°C in about 40 mL of pentane. After 30 minutes, a large amount of yellowish precipitate appeared, and after removal of the supernatant, the yellowish-beige solid (5.865 g), corresponding to [$Li(THF)_4$][$ICe^{III}TREN^{TIPS}$] (**1-LiI-THF$_4$**), was dried under vacuum at 50°C for 2 days. The yellow solid was then extracted with pentane (total volume of 50 mL), then crystallized at -40 °C overnight. Yellow crystals from the first two fractions were gathered and combined to afford [$Ce^{III}TREN^{TIPS}$] (**1**) (2.286 g, 67 %).

^1H NMR (300 MHz, C_6D_6, 298 K) δ 3.81 (s, 6H, C**H$_2$**CH$_2$SiPr$^i_3$), 3.41 (s, 3H, CH$_2$CH$_2$Si(C**H**(CH$_3$)$_2$)$_3$), 2.94 (s, 60H, CH$_2$CH$_2$Si(C**H**(CH$_3$)$_2$)$_3$ and CH$_2$CH$_2$Si(CH(C**H$_3$**)$_2$)$_3$), -17.56 (s, 6H, CH$_2$C**H$_2$**SiPr$^i_3$)

^{13}C NMR (300 MHz, C_6D_6, 298 K) δ 19.2 (s), 17.9 (s), -5 (s)

FTIR ν (cm^{-1}) (powder) : 2935 (s), 2881 (s), 2859 (s), 2836 (s), 2820 (s), 1458 (vs), 1092 (vs), 1070 (s), 1002 (vs), 943 (vs), 923 (vs), 913 (s), 877 (vs), 768 (vs), 752 (vs), 738 (vs), 666 (vs), 628 (vs)

Elemental analysis for $CeC_{33}H_{75}N_4Si_3$, Found (Theoretical): C, 52.78 (52.68) H, 10.03 (10.05); N, 7.46 (7.45)

e) Synthesis of [$ClCe^{IV}TREN^{TIPS}$] (2-Cl)

Given the light sensitivity of **2-Cl**, the reaction should be carried out in the dark.

Benzyl chloride (16 µL, 0.14 mmol, 1 eq.) was added to a solution of **1** (100 mg, 0.14 mmol, 1 eq.) in 2 mL of pentane. The solution turned dark blue within minutes. The mixture was left under stirring in the dark for 2 hours. The volatiles were removed under reduced pressure for 30 minutes, and the deep purple solid was rinsed with 3 x 1 mL of MeCN on a fritted filter. After drying under vacuum for 30 minutes, a purple solid could be recovered (61 mg, 55 %). Deep purple crystals suitable for XRD could be grown from a concentrated solution in THF at -40 °C for a few hours.

^1H NMR (300 MHz, C_6D_6, 298 K) δ 5.05 (t (J = 4.8 Hz), 6H, C**H$_2$**CH$_2$SiPr$^i_3$), 3.34 (t (J = 4.7 Hz), 6H, CH$_2$C**H$_2$**SiPr$^i_3$), 1.52 (sept (J = 7.5 Hz), 9H, CH$_2$CH$_2$Si(C**H**(CH$_3$)$_2$)$_3$); 1.20 (s, 54H, CH$_2$CH$_2$Si(CH(C**H$_3$**)$_2$)$_3$

^{13}C NMR (300 MHz, C_6D_6, 298 K) δ 67.2 (s, 3C, **C**H$_2$CH$_2$Si(CH(CH$_3$)$_2$), 49.2 (s, 3C, CH$_2$**C**H$_2$Si(CH(CH$_3$)$_2$), 19.4 (s, 18C, CH$_2$CH$_2$ Si(CH(**C**H$_3$)$_2$), 13.9 (s, 9C, CH$_2$CH$_2$Si(**C**H(CH$_3$)$_2$)

FTIR ν (cm^{-1}) (powder) : 2937 (s), 2924 (s), 2885 (s), 2860 (s), 1455 (s), 1037 (vs), 1009 (vs), 917 (vs), 880 (vs), 710 (vs), 371 (vs), 634 (vs), 625 (vs)

Elemental analysis for $ClCeC_{33}H_{75}N_4Si_3$, Found (Theoretical): C, 50.48 (50.31) H, 9.64 (9.60); N, 7.24 (7.11)

f) Synthesis of [BrCe^{IV}TREN^{TIPS}] (2-Br)

Given the high light sensitivity of **2-Br**, the reaction should be carried out in the dark.

Benzyl bromide (16 µL, 0.14 mmol, 1 eq.) was added to a solution of **1** (100 mg, 0.14 mmol, 1 eq.) in 2 mL of pentane. The solution turned dark blue within minutes. The mixture was left under stirring in the dark for 2 hours. The volatiles were removed under reduce pressure for 30 minutes, and the dark blue solid was rinsed with 3 x 1 mL of MeCN on a fritted filter. After drying under vacuum for 30 minutes, a purple solid could be recovered (71.5 mg, 66 %). Deep purple crystals could be grown from a solution in 0.5 mL of THF at -40°C overnight.

^{1}H NMR (300 MHz, C_6D_6, 298 K) δ 5.15 (t (J = 4.7 Hz), 6H, C**H$_2$**CH$_2$SiPr$^i_3$), 3.32 (t (J = 4.7 Hz), 6H, CH$_2$C**H$_2$**SiPr$^i_3$), 1.55 (sept (J = 7.5 Hz), 9H, CH$_2$CH$_2$Si(C**H**(CH$_3$)$_2$)$_3$); 1.22 (s, 54H, CH$_2$CH$_2$Si(CH(C**H$_3$**)$_2$)$_3$

^{13}C NMR (300 MHz, C_6D_6, 298 K) δ 67.7 (s, 3C, **C**H$_2$CH$_2$Si(CH(CH$_3$)$_2$), 49.1 (s, 3C, CH$_2$**C**H$_2$Si(CH(CH$_3$)$_2$), 19.6 (s, 18C, CH$_2$CH$_2$ Si(CH(**C**H$_3$)$_2$), 14.2 (s, 9C, CH$_2$CH$_2$Si(**C**H(CH$_3$)$_2$)

FTIR ν (cm^{-1}) (powder) : 2937 (s), 2885 (s), 2860 (s), 1457 (s), 1271 (vs), 1038 (vs), 1009 (vs), 989 (vs), 918 (vs), 880 (vs) 708 (vs) 672 (vs), 629 (vs)

Elemental analysis for $BrCeC_{33}H_{75}N_4Si_3$, Found (Theoretical): C, 47.92 (47.62) H, 9.18 (9.08); N, 6.70 (6.73)

g) Synthesis of [FCe^{IV}TREN^{TIPS}] (2-F)

Compound **1** (100 mg, 0.14 mmol, 1 eq.) was dissolved in 2 mL of pentane and added to ferrocenium tetrafluoroborate (36 mg, 0.14 mmol, 1 eq.). The colour turned immediately dark red, and the reaction was left stirring for 10 hours. The volatiles were removed under reduced pressure, and the dark red solid was rinsed with MeCN until the supernatant was colourless. The remaining solid was finally extracted with pentane, and volatiles were removed under reduced pressure, yielding [FCe^{IV}TREN^{TIPS}] (**2-F**) as a dark red powder (30 mg, 28%). Crystals suitable for XRD could be grown from a saturated solution in THF at -40 °C.

^{1}H NMR (300 MHz, C_6D_6, 298 K) δ 4.68 (t (J = 4.5 Hz), 6H, C**H$_2$**CH$_2$SiPr$^i_3$), 3.41 (t (J = 4.5 Hz), 6H, CH$_2$C**H$_2$**SiPr$^i_3$), 1.40 (sept (J = 7.1 Hz), 9H, CH$_2$CH$_2$Si(C**H**(CH$_3$)$_2$)$_3$); 1.20 (s, 54H, CH$_2$CH$_2$Si(CH(C**H$_3$**)$_2$)$_3$

^{13}C NMR (300 MHz, C_6D_6, 298 K) δ 64.9 (s, 3C, **C**H$_2$CH$_2$Si(CH(CH$_3$)$_2$), 48.9 (s, 3C, CH$_2$**C**H$_2$Si(CH(CH$_3$)$_2$), 19.2 (s, 18C, CH$_2$CH$_2$Si(CH(**C**H$_3$)$_2$), 13.2 (s, 9C, CH$_2$CH$_2$Si(**C**H(CH$_3$)$_2$)

^{19}F NMR (300 MHz, THD-d_8, 298 K) δ 311.85 (s, 1F, Ce-**F**)

FTIR ν (cm^{-1}) (powder) : 2939 (s), 2861 (s), 1460 (s), 1052 (vs), 1010 (vs), 919 (vs), 879 (vs), 717 (s), 371 (vs), 672 (vs), 636 (vs)

Elemental analysis for $FCeC_{33}H_{75}N_4Si_3$, Found (Theoretical): C, 50.78 (51.38) H, 9.79 (9.80); N, 7.28 (7.26)

h) Synthesis of 1-allyloxy-2-chlorobenzene

1-allyloxy-2-chlorobenzene was synthesized according to a literature procedure[23].

2-chlorophenol (0.8 mL, 8 mmol, 1 eq.), potassium carbonate (2.2 g, 16 mmol, 2 eq.), 3-bromo-1-propene (1.4 mL, 16 mmol, 2 eq.), and 5 mL of dry acetonitrile were mixed in a Schlenk flask and heated for 20 h at 70 °C. The mixture was allowed to cool down to room temperature, and 35 mL of diethyl ether was added. The organic mixture was washed with water, washed twice with a saturated KOH solution, dried with magnesium sulfate, and concentrated in vacuo at 250 mBar for 4h. 1-allyloxy-2-chlorobenzene could be isolated as a colorless oil (808 mg, 60%).

^{1}H NMR (300 MHz, $CDCl_3$, 298 K) δ 7.38 (dd, J = 7.6, 1.6 Hz, 1H), 7.20 (ddd, J = 8.5, 7.6, 1.6 Hz, 1H), 6.97 – 6.85 (m, 2H), 6.08 (ddt, J = 17.2, 10.3, 5.1 Hz, 1H), 5.48 (dq, J = 17.2, 1.7 Hz, 1H), 5.32 (dq, J = 10.3, 1.7 Hz, 1H), 4.63 (dt, J = 5.1, 1.7 Hz, 3H).

i) Reactivity with chlorinated substrates

<u>General procedure for C-Cl activation:</u>

Compound **1** (15 mg, 20 µmol), chlorinated substrate (20 µmol), tributyltin hydride (5.3 µL, 20 µmol), and an internal standard, 1,3,5-trimethoxybenzene (3.3 mg, 20 µmol), were mixed in 0.5 mL of THF-d_8, and sealed in a J. Young NMR tube, and irradiated for designated amounts of time. The reaction was followed by ^{1}H spectroscopy.

j) Reactivity with fluorinated substrates

<u>General procedure for C-F activation:</u>

Compound **1** (15 mg, 20 µmol), fluorinated substrate (20 µmol), tributyltin hydride (5.3 µL, 20 µmol), and an internal standard, trimethoxybenzene (3.3 mg, 20 µmol), were mixed in 0.5 mL of THF-d_8, and sealed in a J. Young NMR tube, and irradiated for designated amounts of time. The reaction was followed by ^{1}H and ^{19}F NMR spectroscopy.

II. Spectral data

a) Spectral data for Li_3TREN^{TIPS}

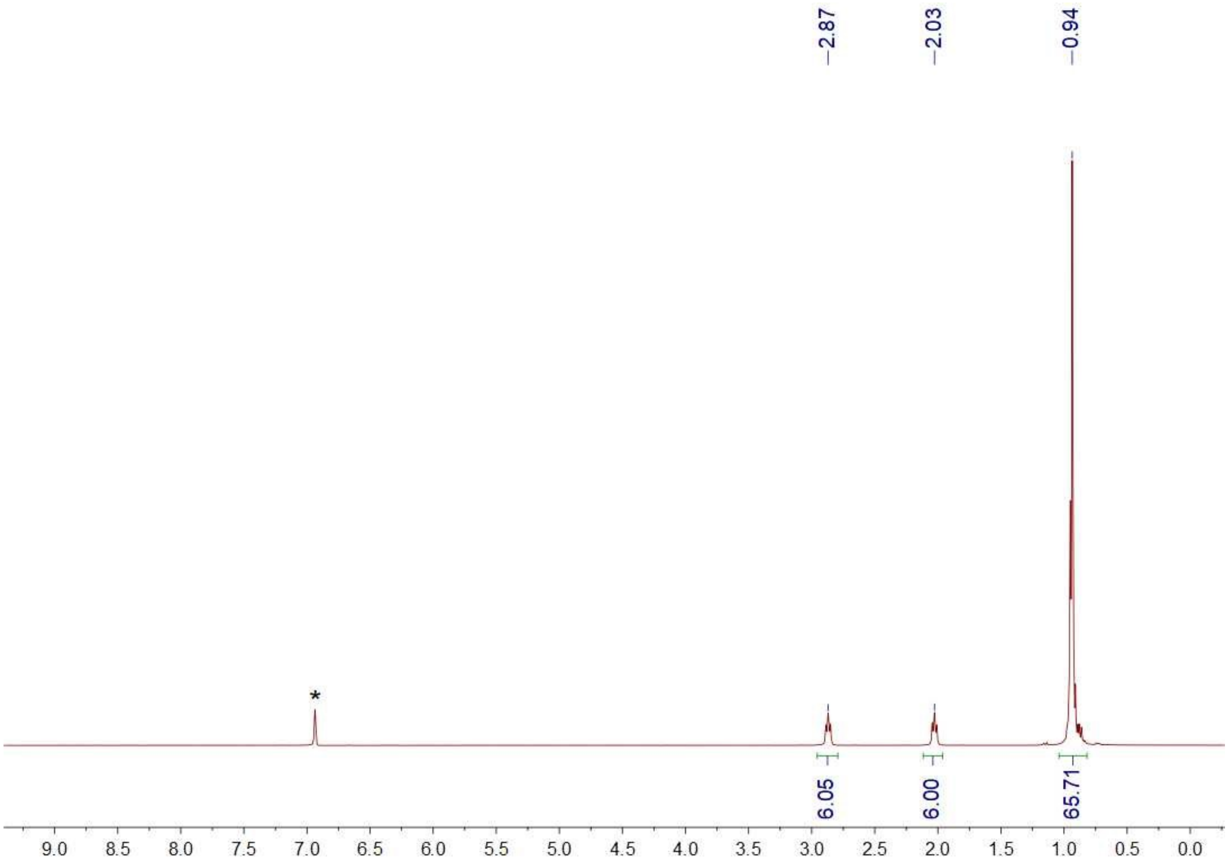

Figure S3. ^{1}H NMR spectrum of [Li_3TREN^{TIPS}] in C_6D_6. Solvent residual peak is indicated with *

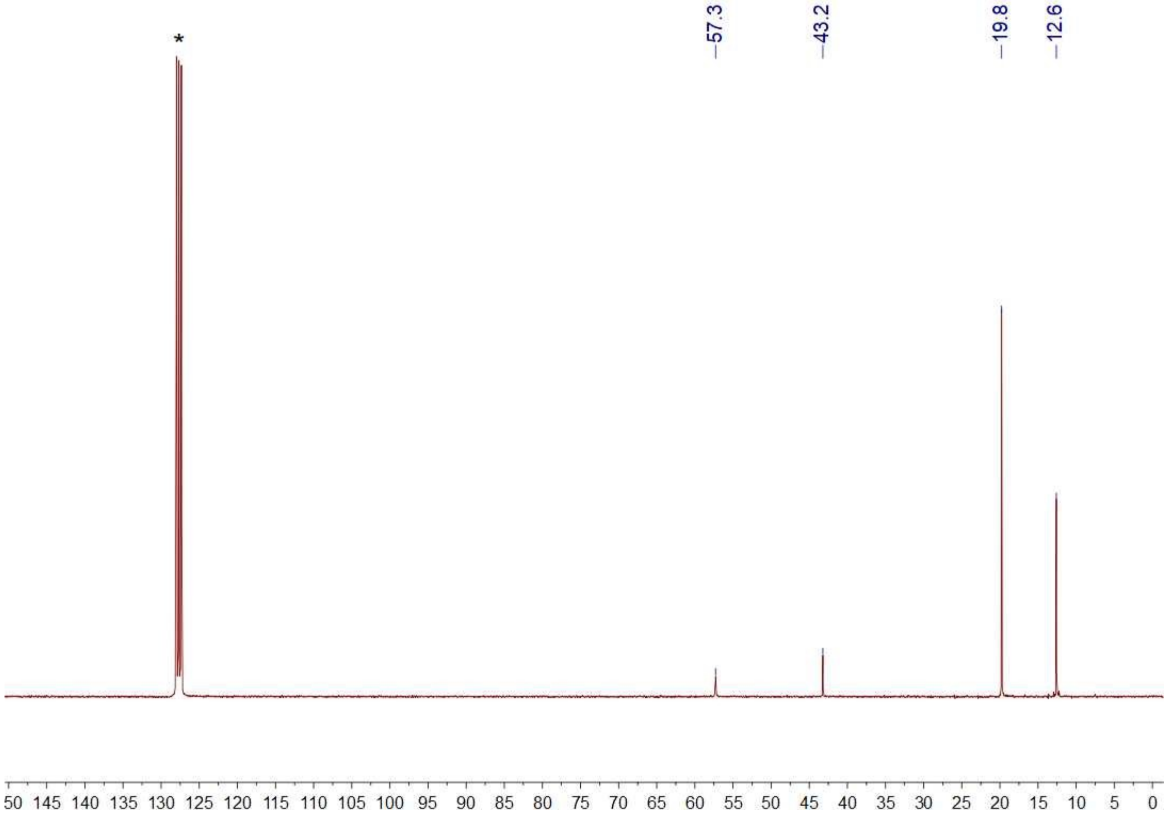

Figure S4. $^{13}C\{^{1}H\}$ NMR spectrum of [Li_3TREN^{TIPS}] in C_6D_6. Solvent residual peak is indicated with *

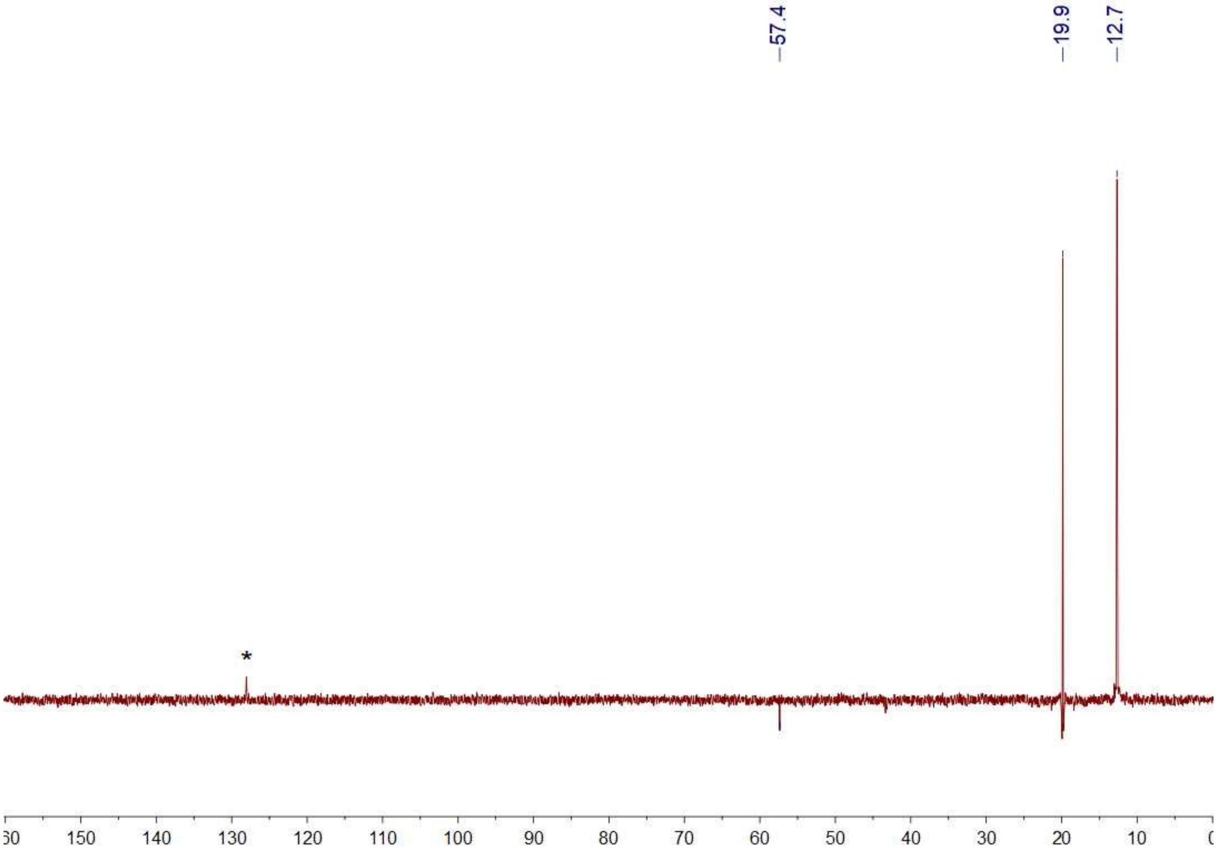

Figure S5. DEPT 135 $^{13}C\{^1H\}$ spectrum of [Li_3TREN^{TIPS}] in C_6D_6. Solvent residual peak is indicated with *

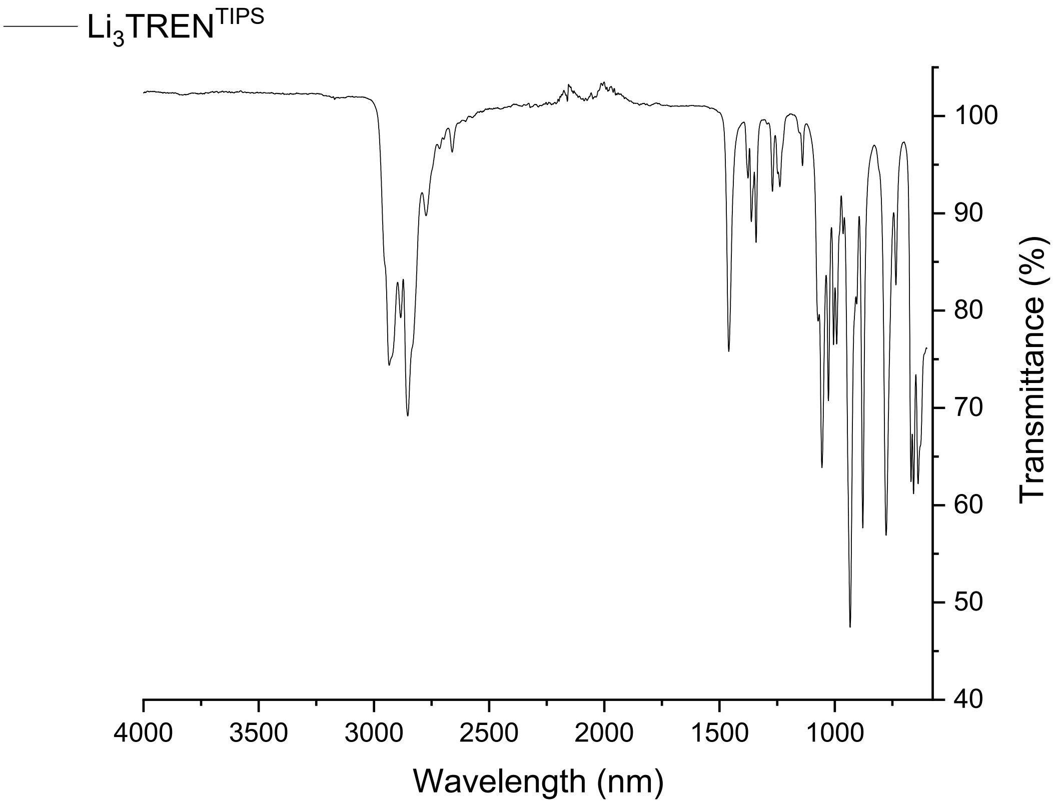

Figure S6. IR spectrum of neat Li_3TREN^{TIPS}

b) Spectral data for $[Li(THF)_4][ICeTREN^{TIPS}]$ (1-LiI-THF$_4$)

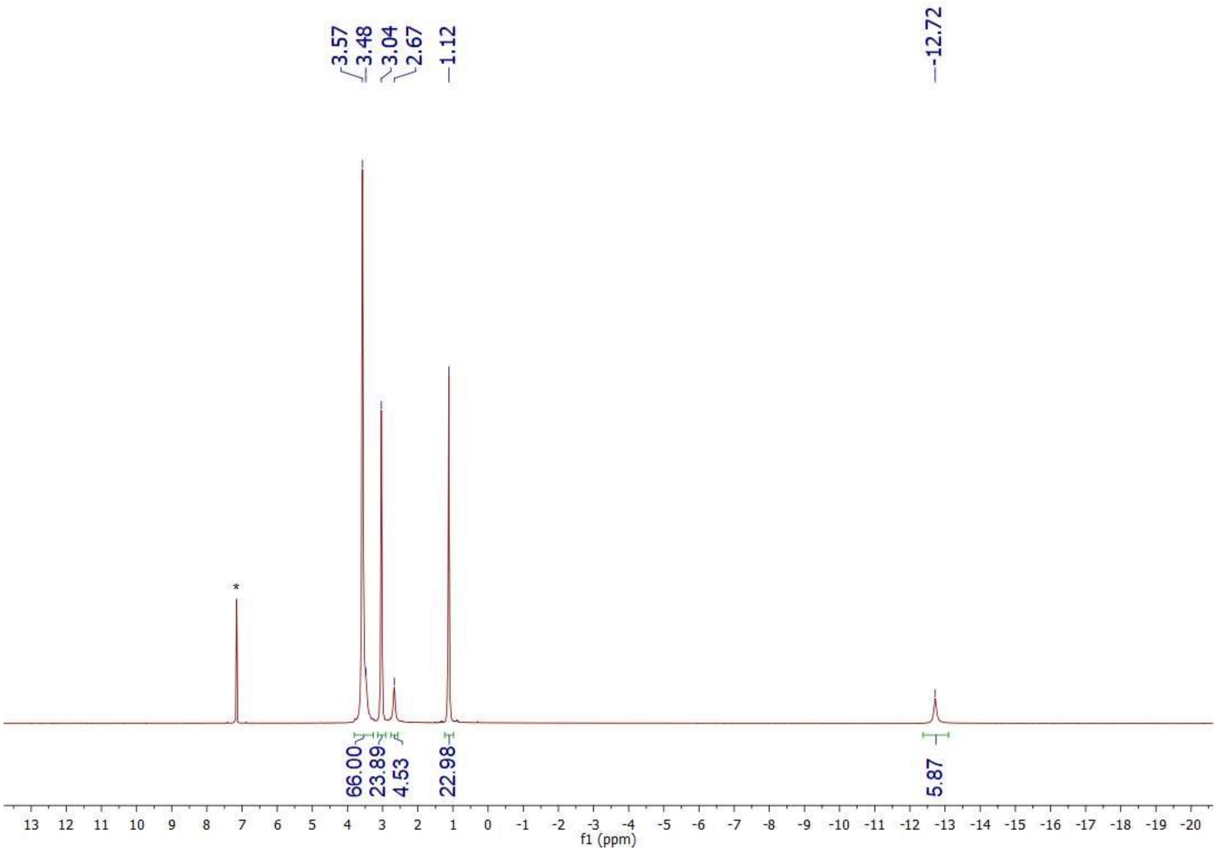

Figure S7. ^{1}H NMR of **1-LiI-THF$_4$** in C_6D_6 at 298 K. Solvent residual peak is indicated with *

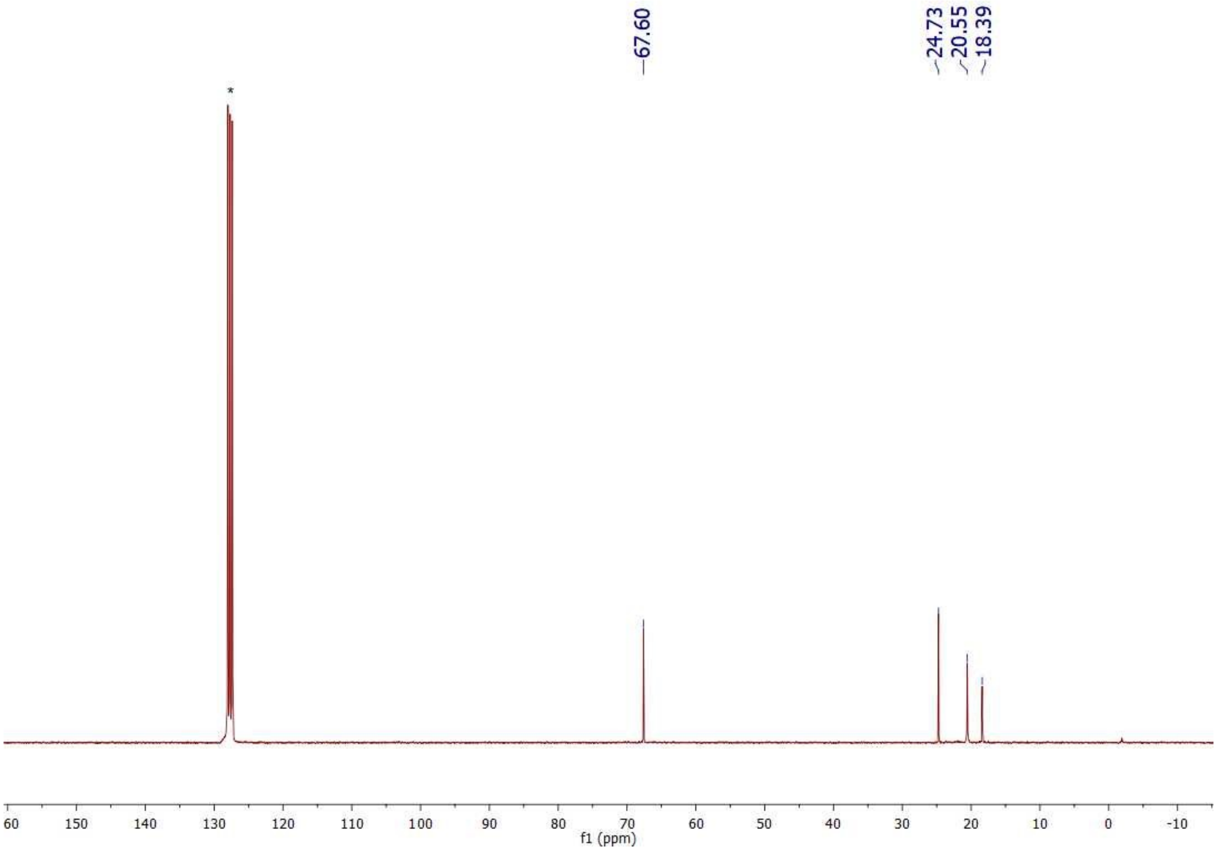

Figure S8. $^{13}C\{^1H\}$ NMR of **1-LiI-THF$_4$** in C_6D_6 at 298 K. Solvent residual peak is indicated with *

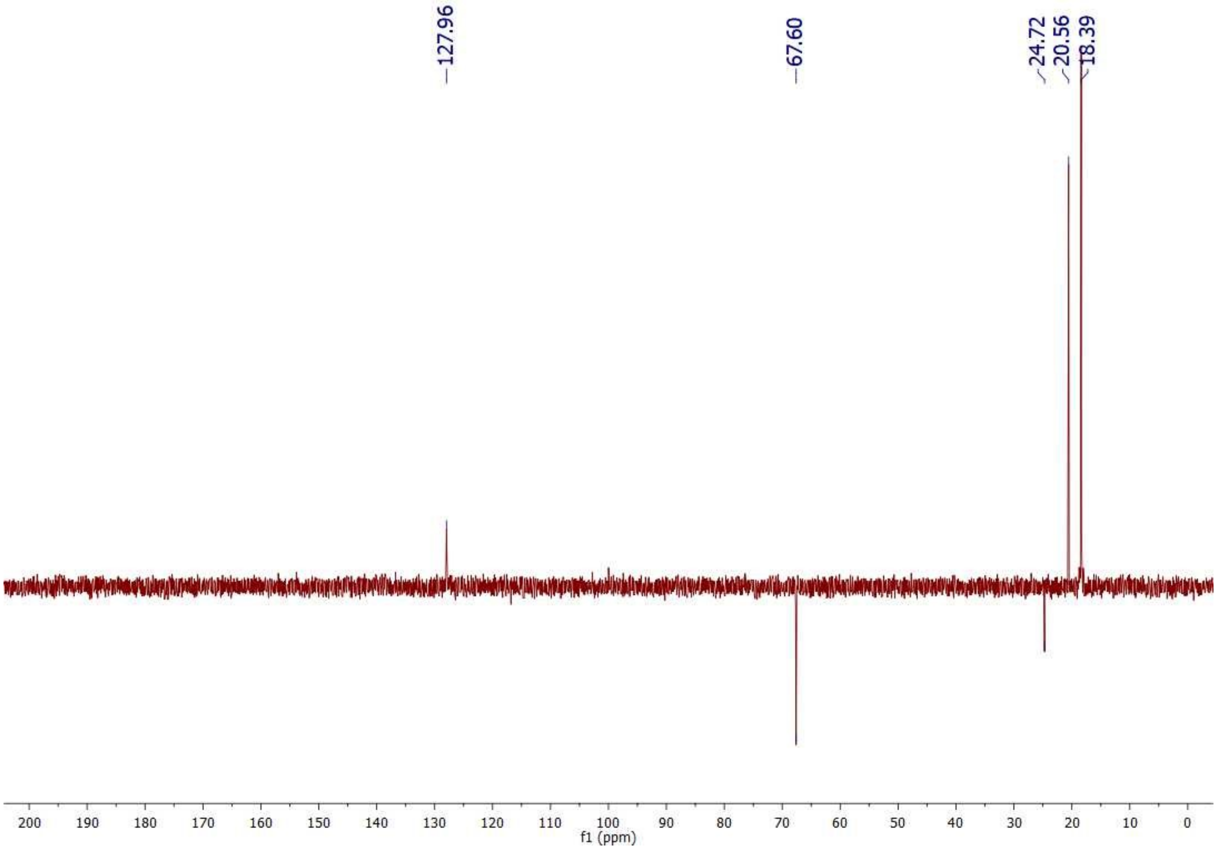

Figure S9. DEPT 135 $^{13}C\{^{1}H\}$ spectrum of 1-LiI-THF$_4$ in C_6D_6 at 298 K

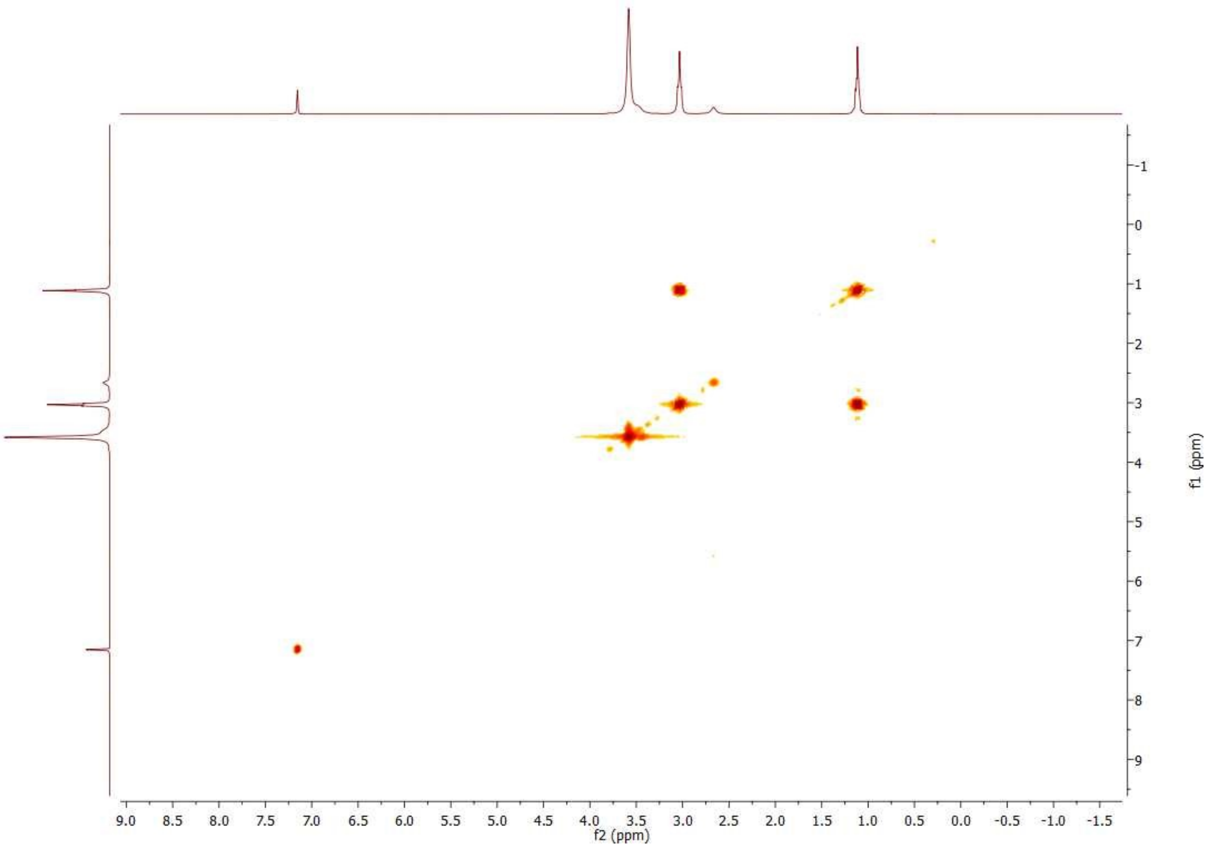

Figure S10. ^{1}H-^{1}H COSY spectrum of **1-LiI-THF$_4$** in C_6D_6 at 298 K

c) **Spectral data for [Li(THF)$_3$][ICeTREN^{TIPS}] (1-LiI-THF$_3$)**

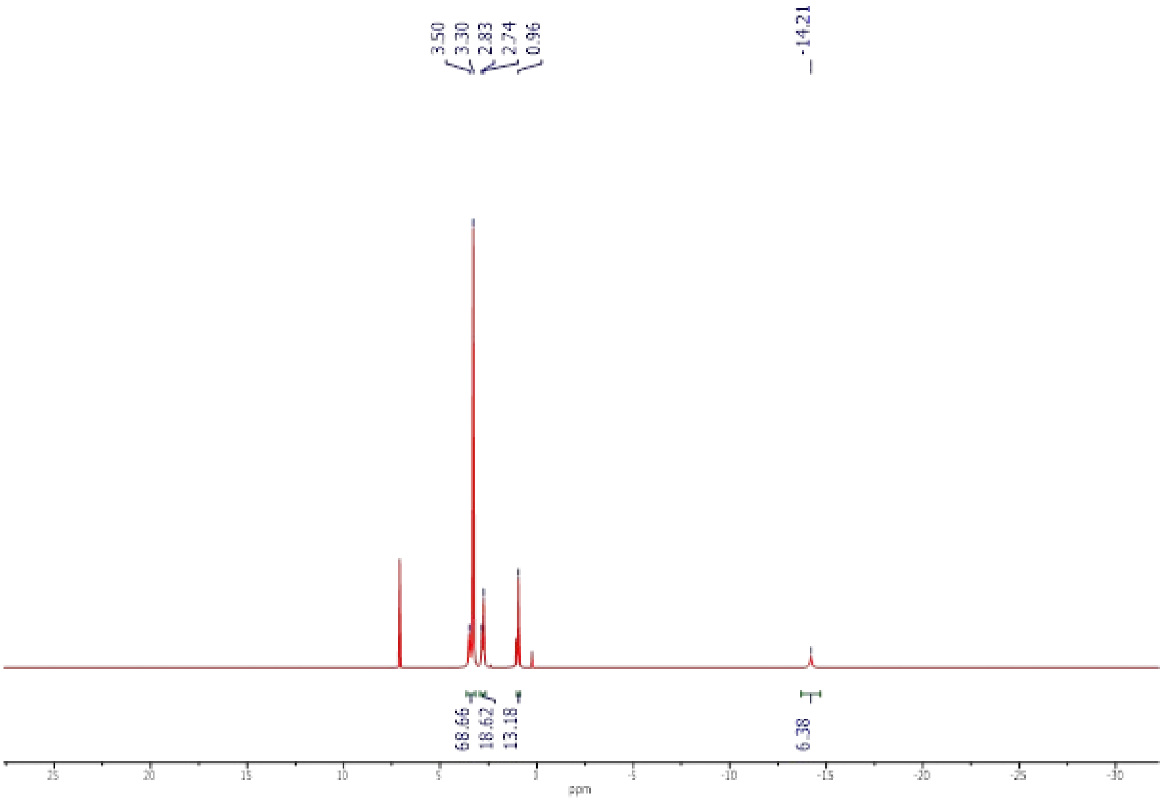

Figure S11. ^{1}H NMR of **1-LiI-THF$_3$** in C_6D_6 at 298 K

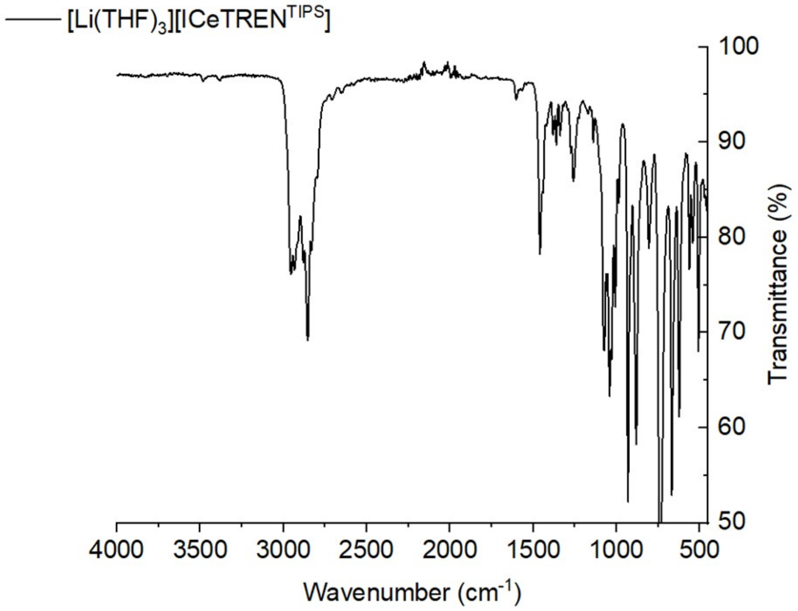

Figure S12. IR spectrum of **1-LiI-THF$_3$** at 298 K

d) Spectral data for [Ce^III^TREN^TIPS^] (1)

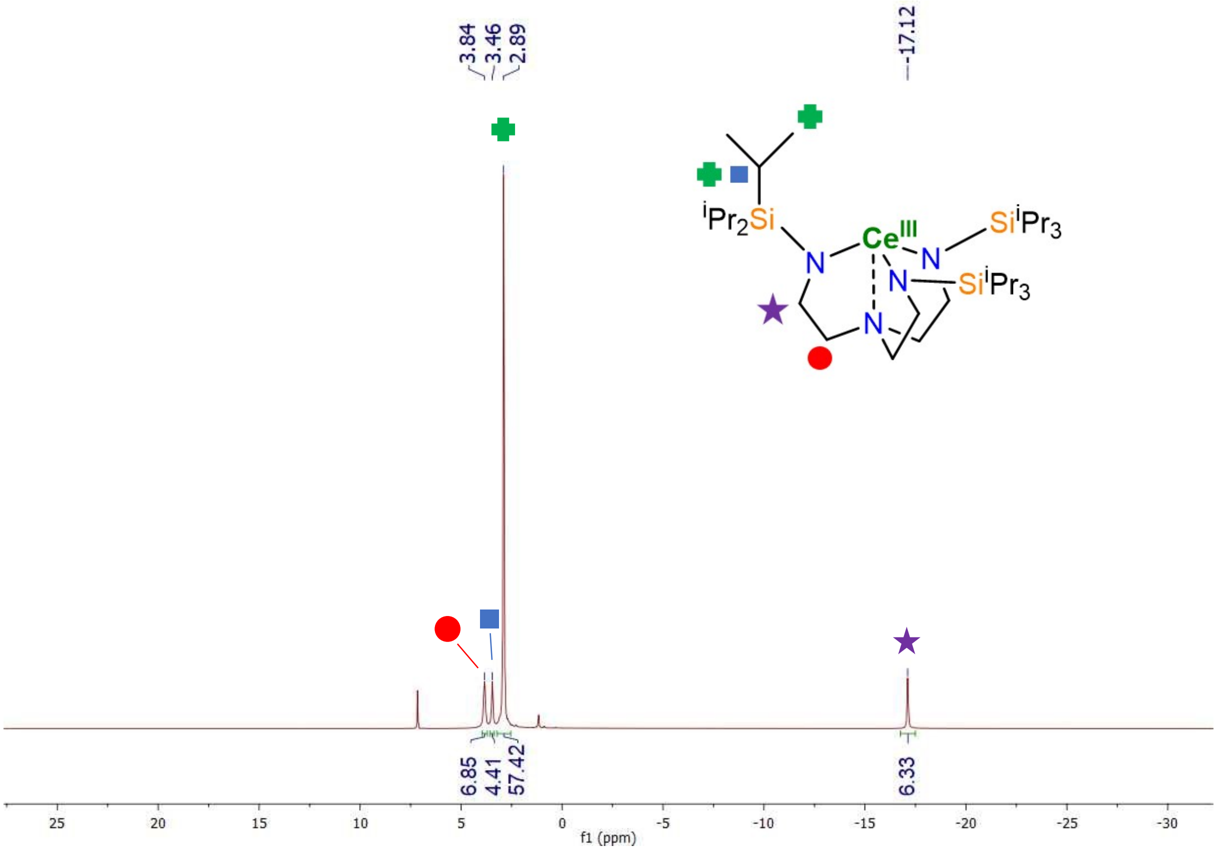

Figure S13. ^1H NMR of **1** in C_6D_6 at 298 K. Residual peaks at 2.49 ppm and 1.15 ppm correspond to the formation of H_3TREN^{TIPS}, created by the hydrolysis of the complex by the residual water in the deuterated solvent.

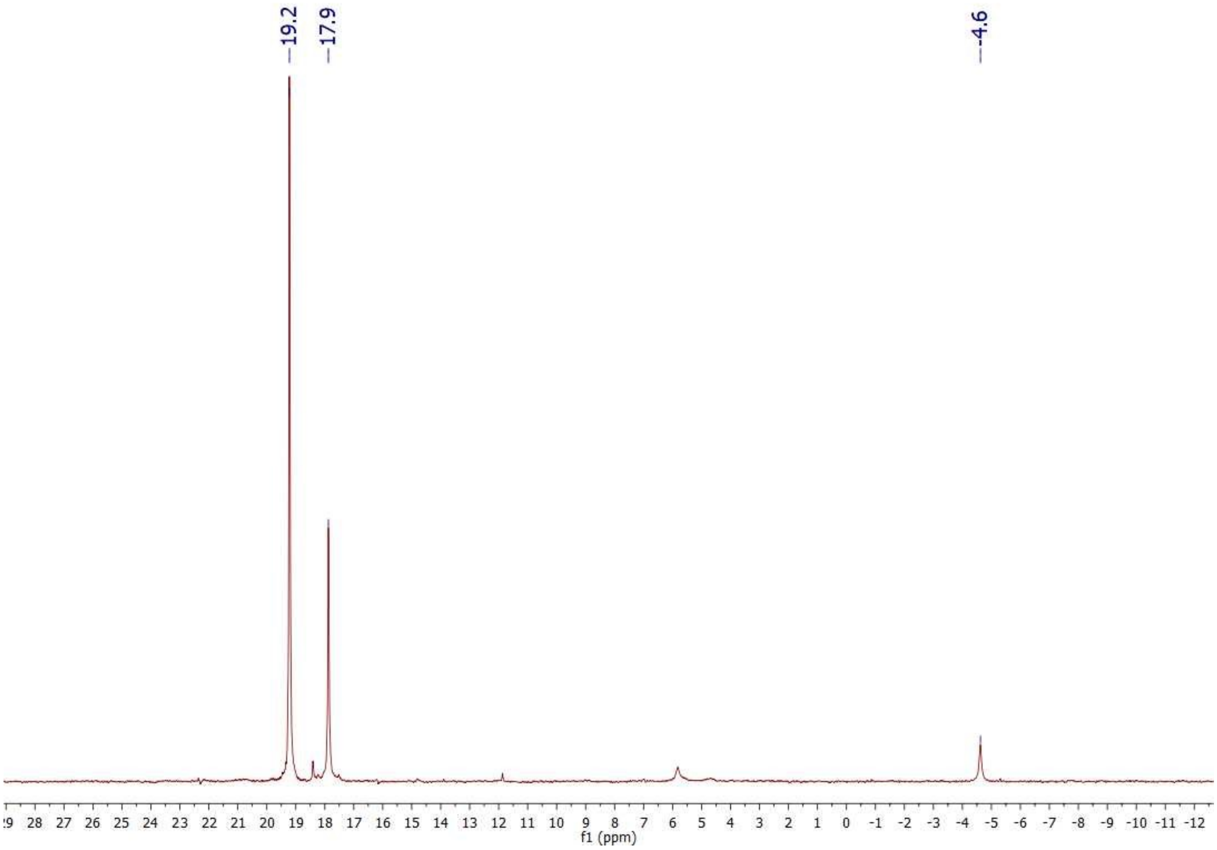

Figure S14. ^{13}C NMR of **1** in C_6D_6 at 298 K

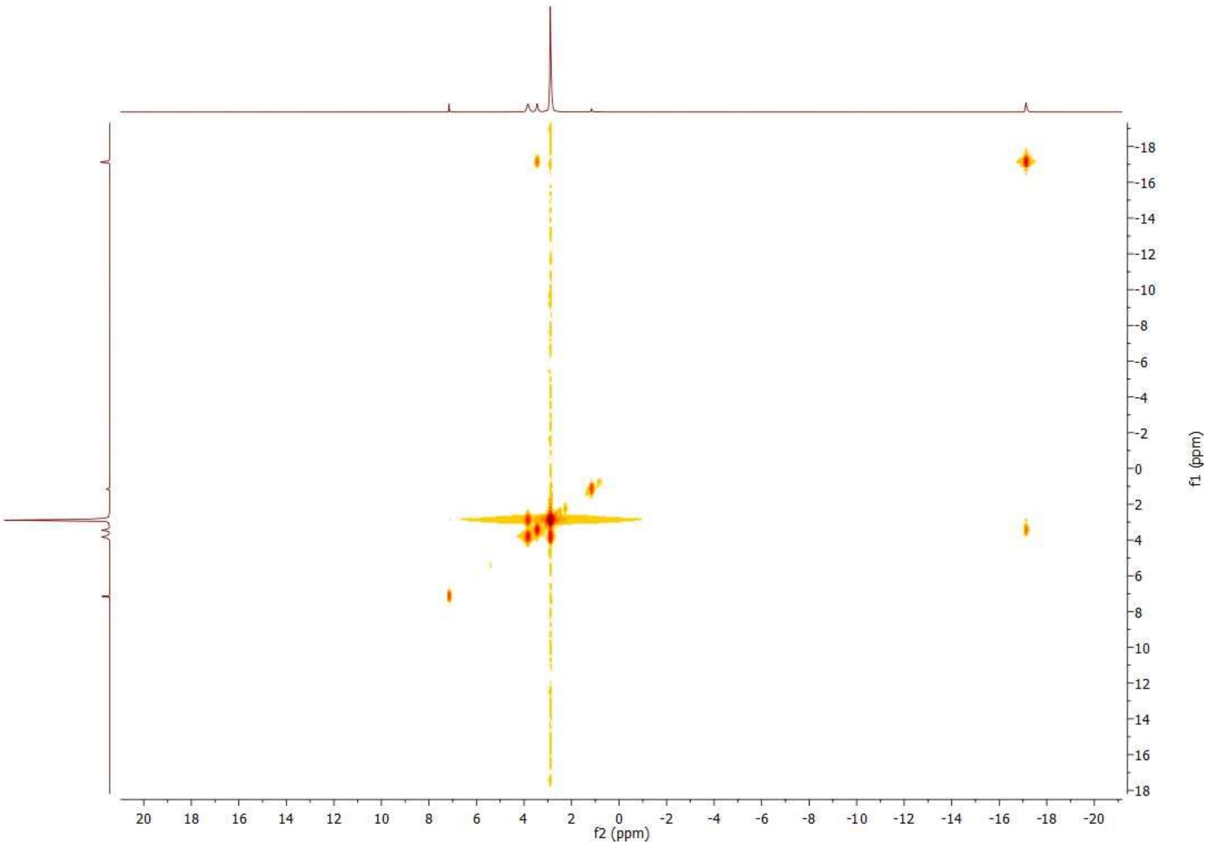

Figure S15. ^1H-^1H COSY spectrum of **1** in C_6D_6 at 298 K

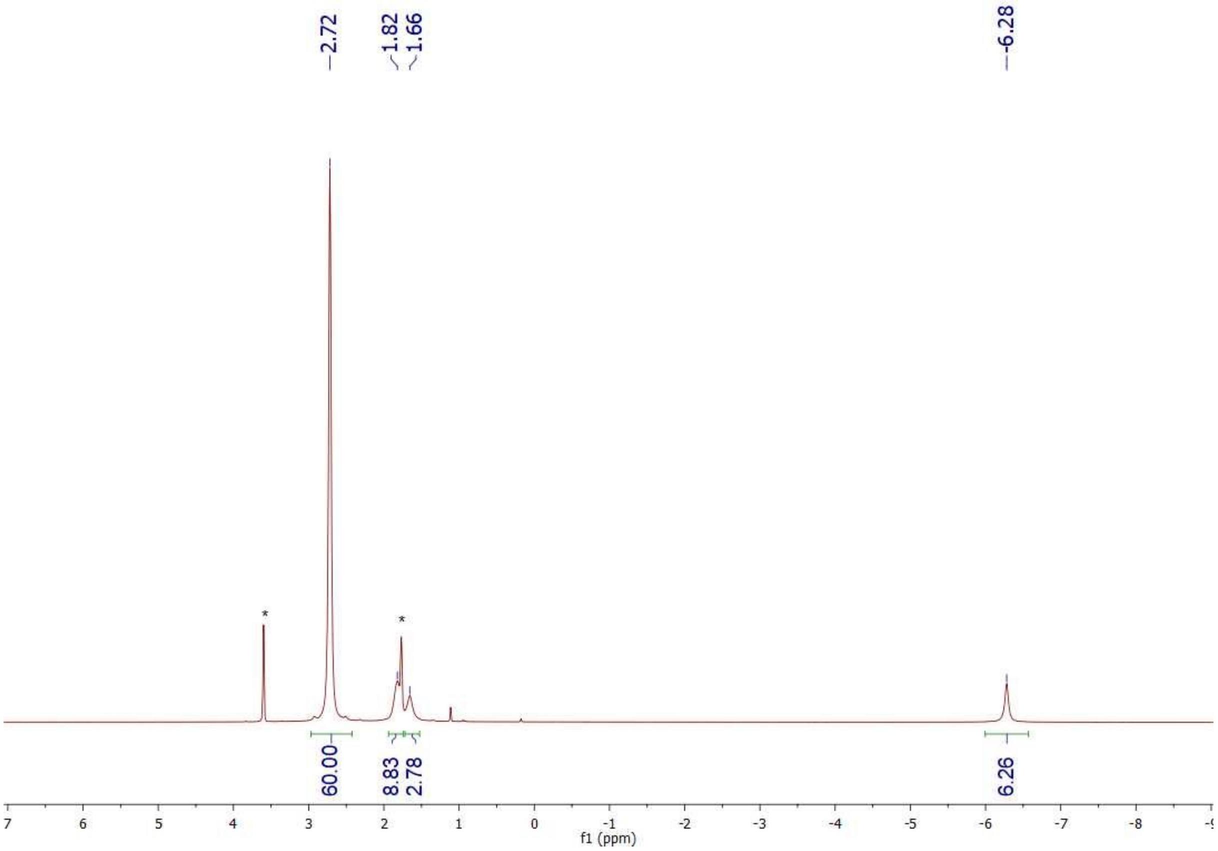

Figure S16. ^1H NMR of **1** in THF-d_8 at 298 K

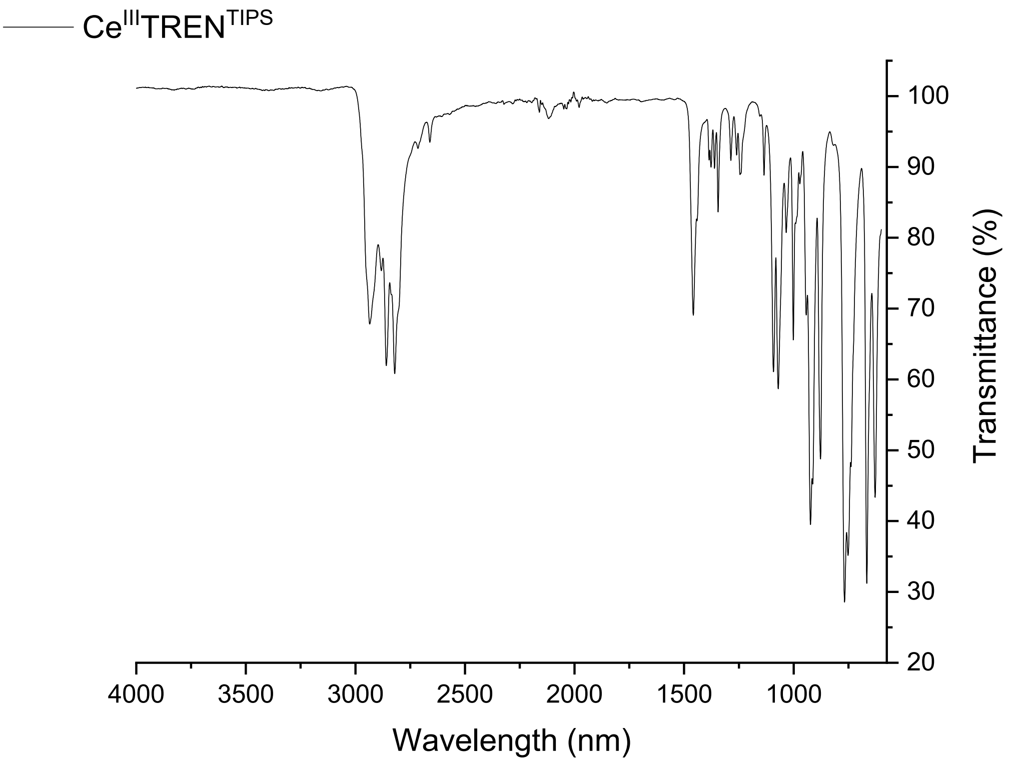

Figure S17. IR spectrum of **1** at 298 K

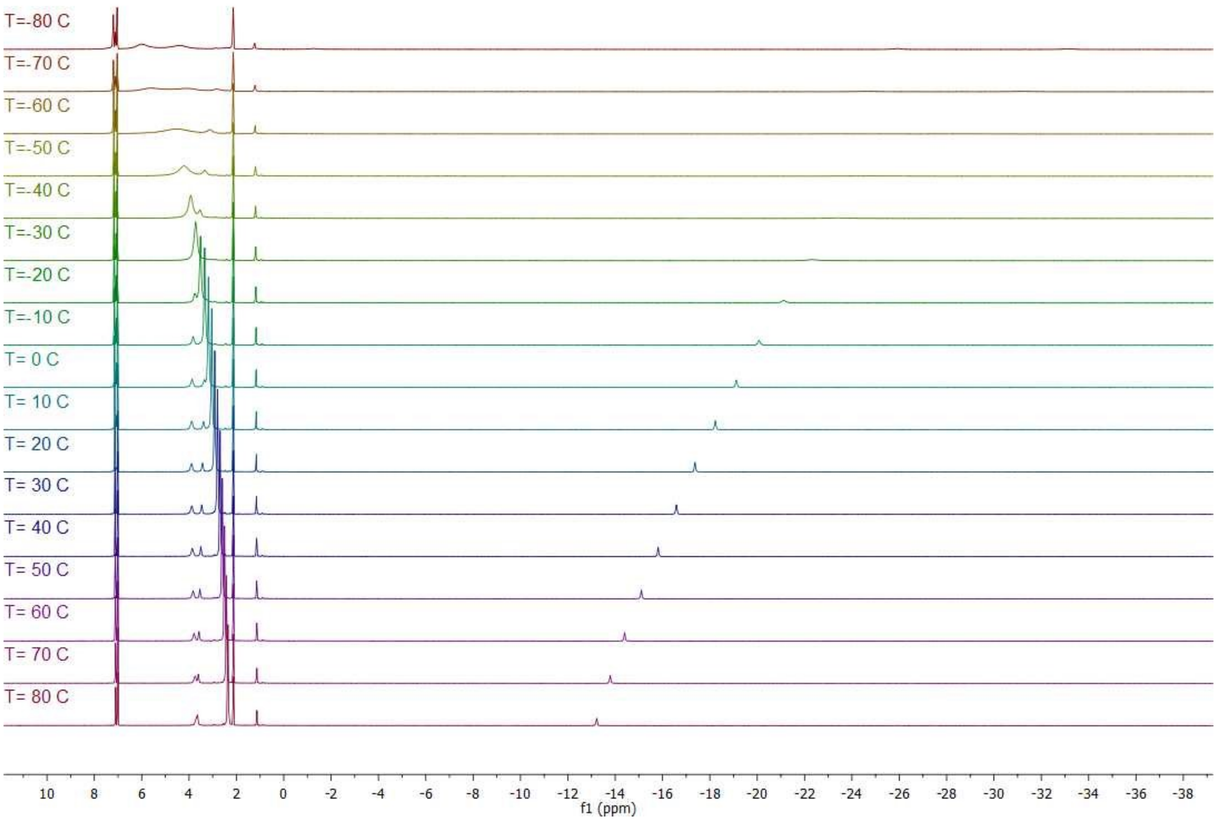

Figure S18. VT NMR of **1** in toluene-d_8 in the -80°C to 80°C temperature range.

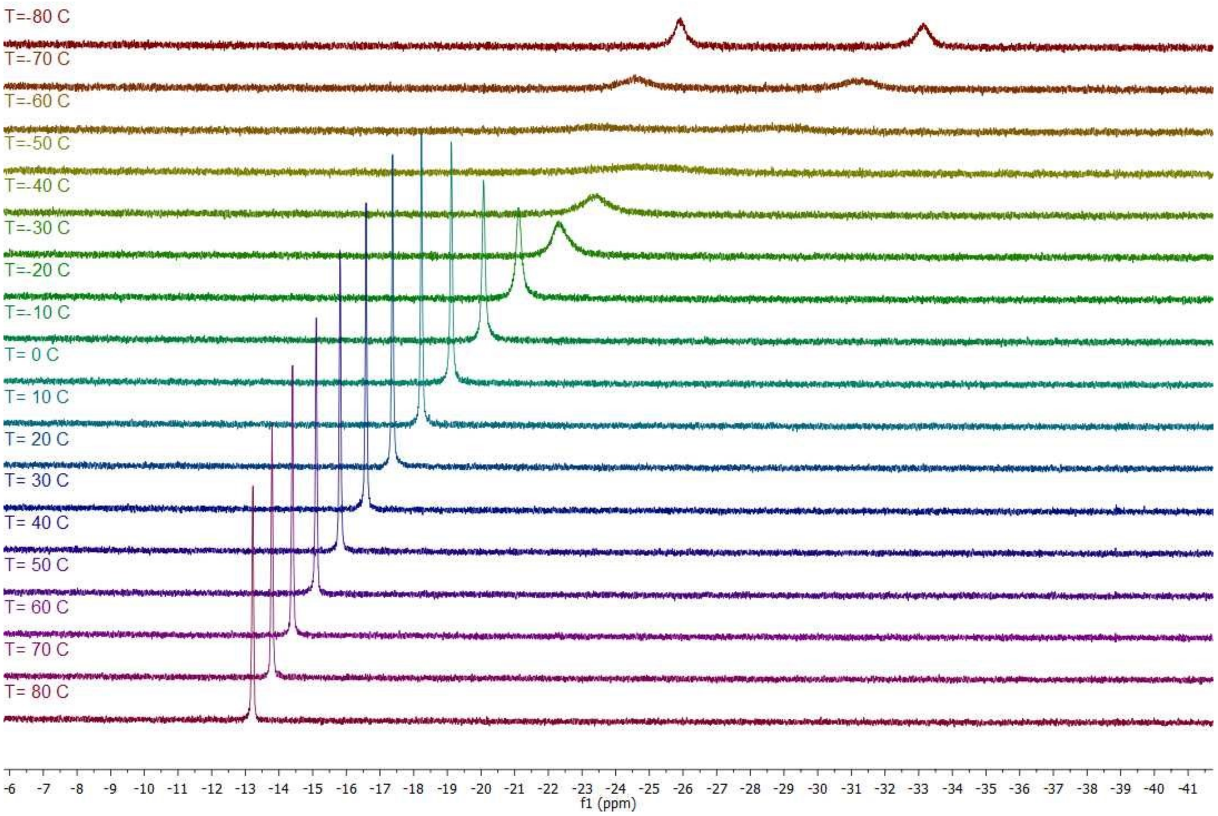

Figure S19. VT NMR of **1** (zoom on the -8 ppm to -38 ppm range) in toluene-d_8 in the -80°C to 20°C temperature range.

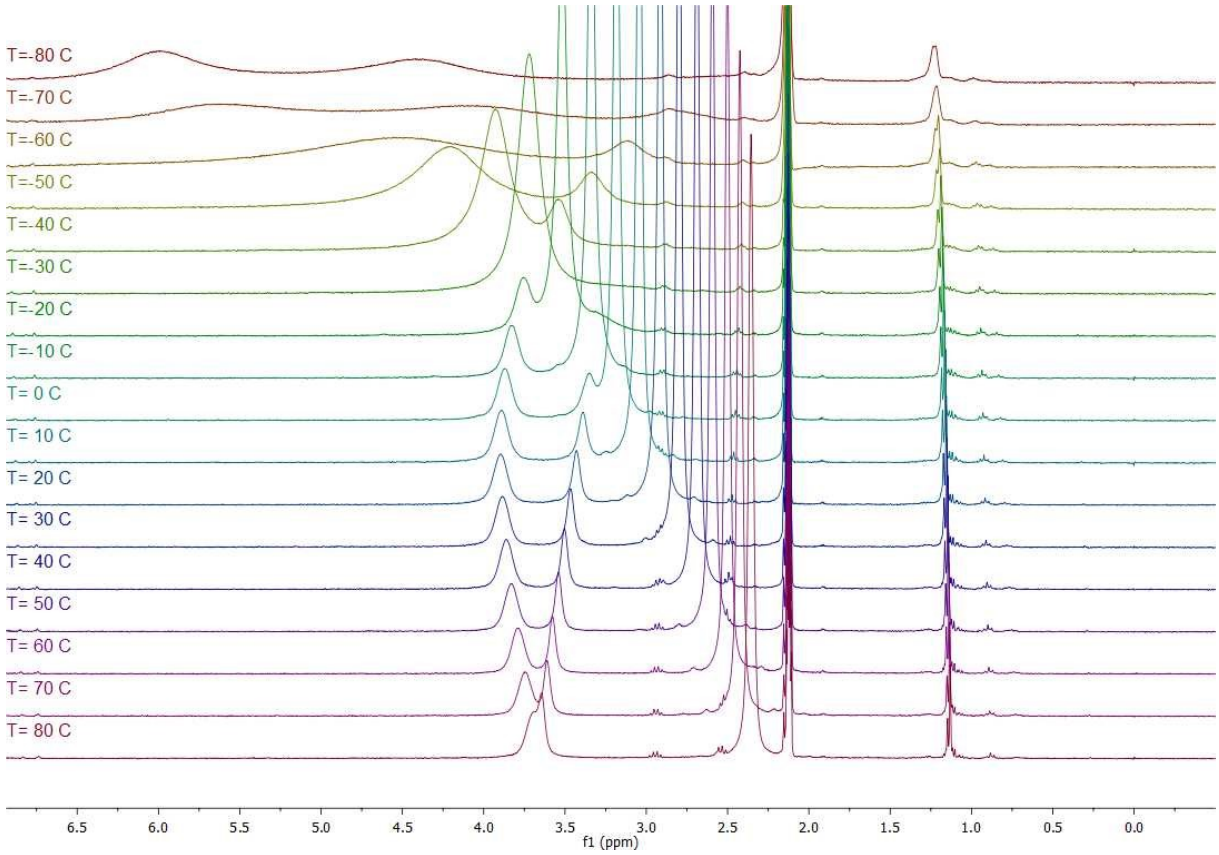

Figure S20. VT NMR of **1** (zoom on the 6 ppm to 0 ppm range) in toluene-d_8 in the -80°C to 20°C temperature range.

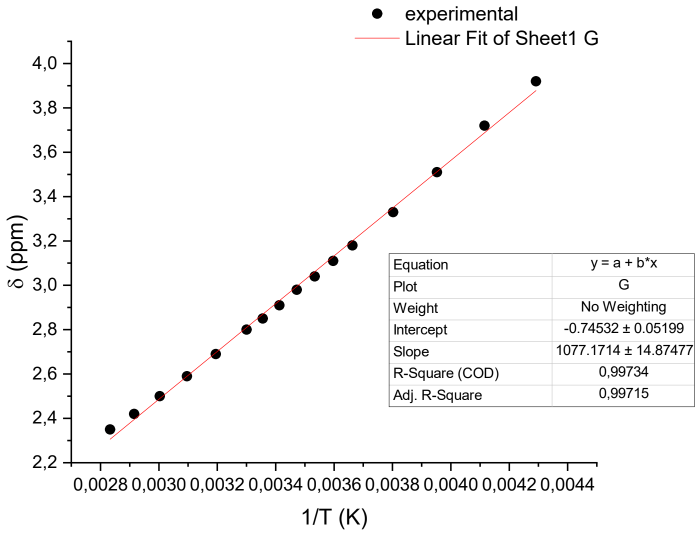

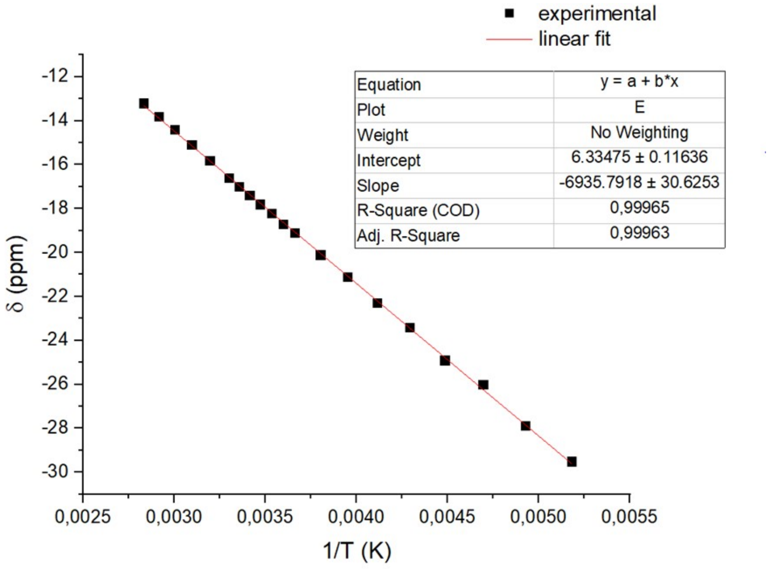

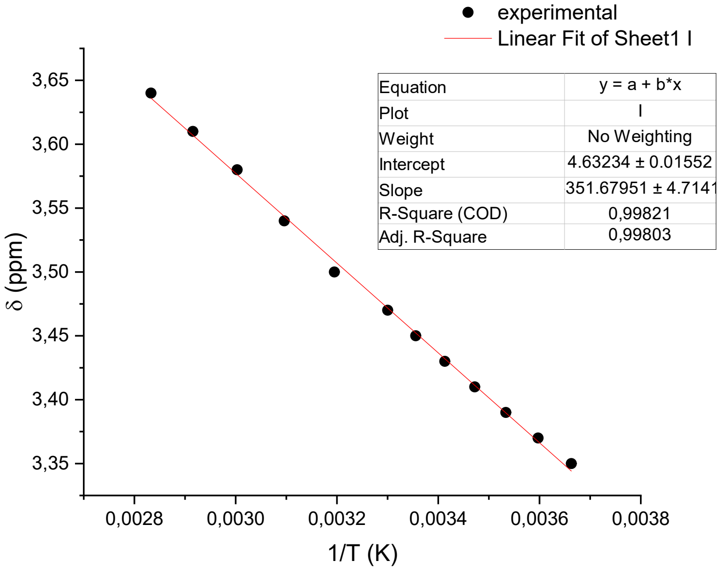

Figure S21. Linear regressions δ=f(1/T) on peaks at -17 ppm, 3.2 ppm, and 3.5 ppm in toluene-d_8 in the -80°C to 80°C temperature range for **1**.

e) Spectral data for [ClCe^{IV}TREN^{TIPS}] (2-Cl)

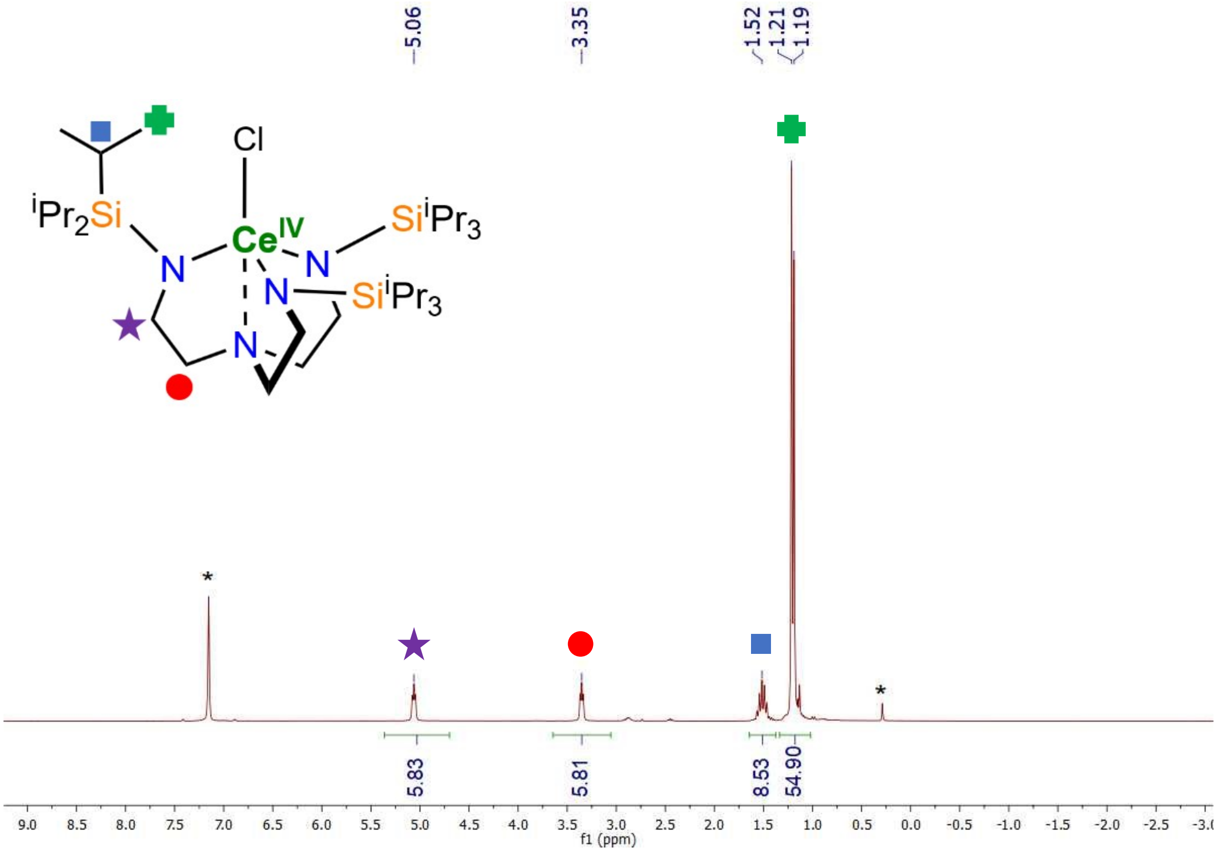

Figure S22. ^{1}H NMR of **2-Cl** in C_6D_6 at 298 K. Residual peaks at 2.86 ppm and 2.45 ppm correspond to the formation of H_3TREN^{TIPS}, created by the hydrolysis of the complex by the residual water in the deuterated solvent. Solvent residual peak and residues of silicon grease are indicated with *

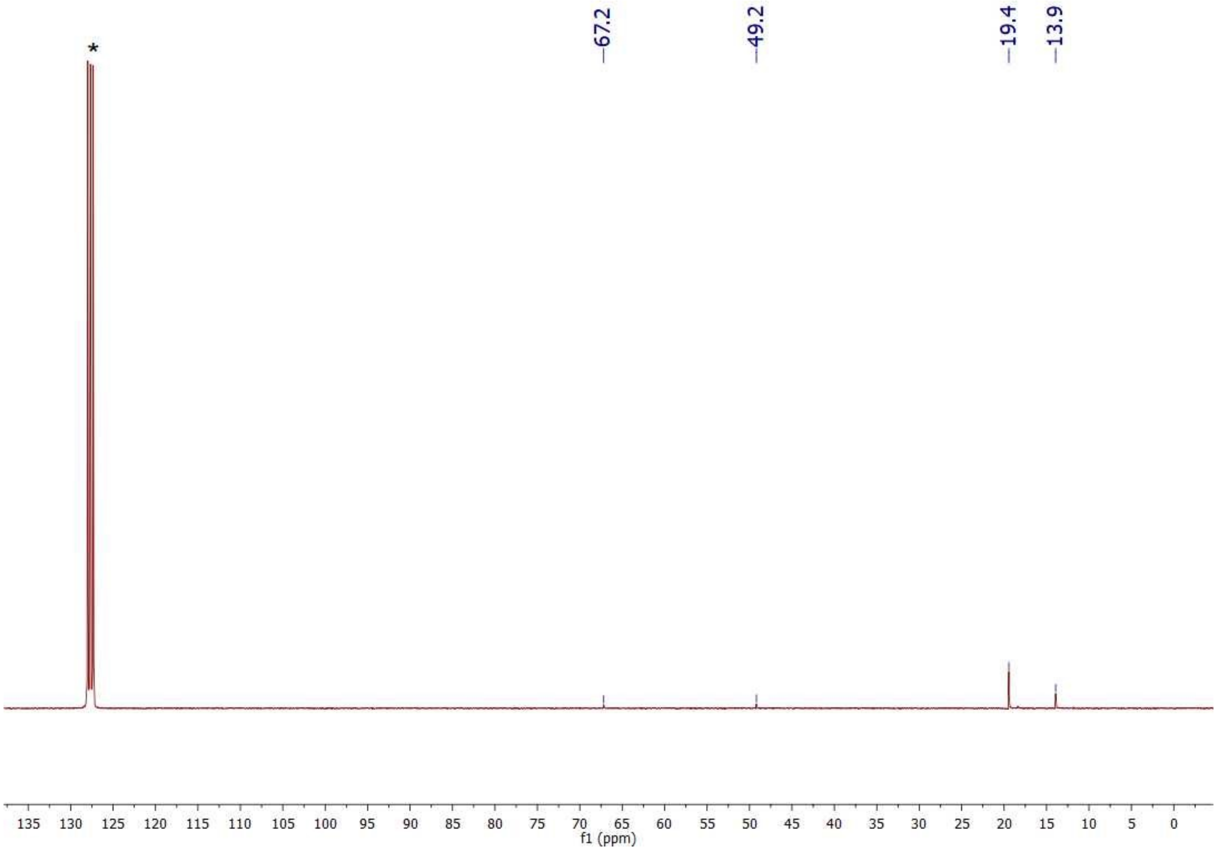

Figure S23. $^{13}C\{^1H\}$ spectrum of **2-Cl** in C_6D_6 at 298 K. Solvent residual peak is indicated with *

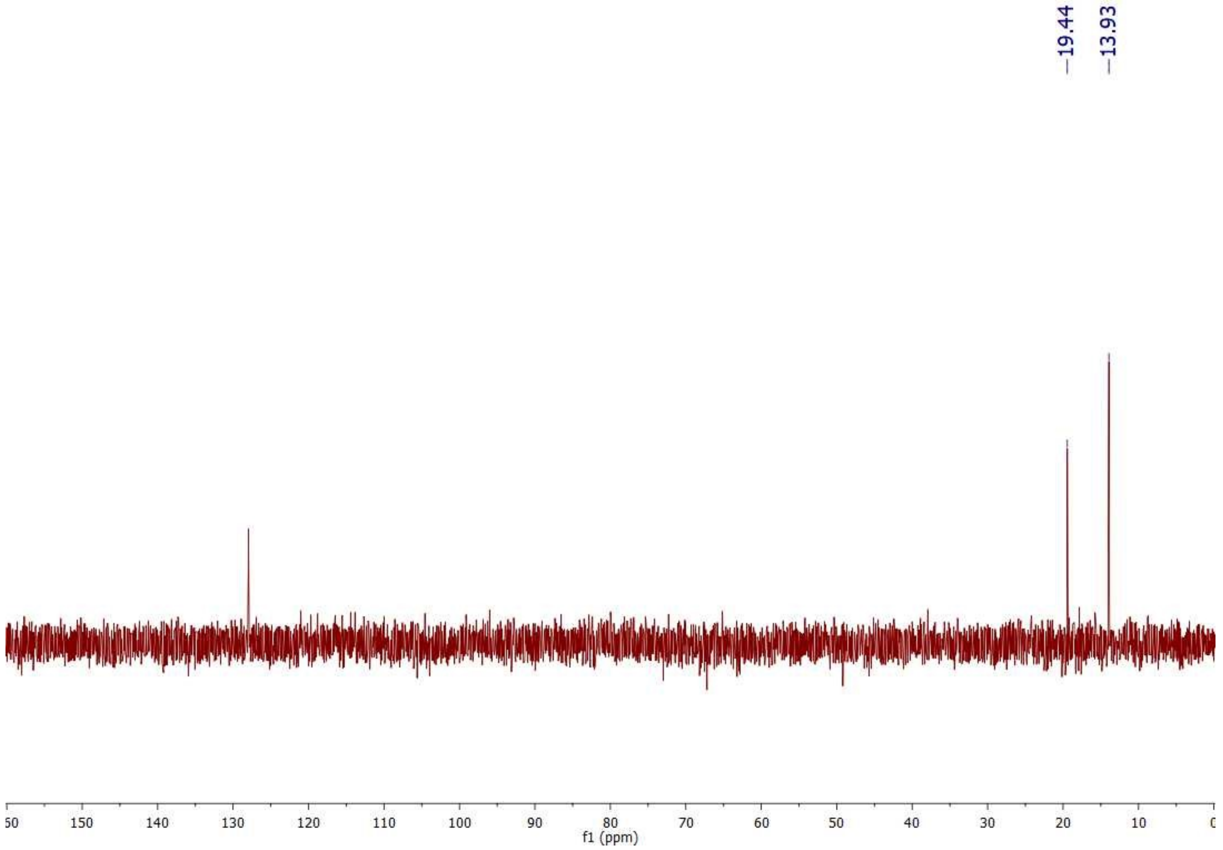

Figure S24. DEPT 135 $^{13}C\{^{1}H\}$ spectrum of **2-Cl** in C_6D_6 at 298 K

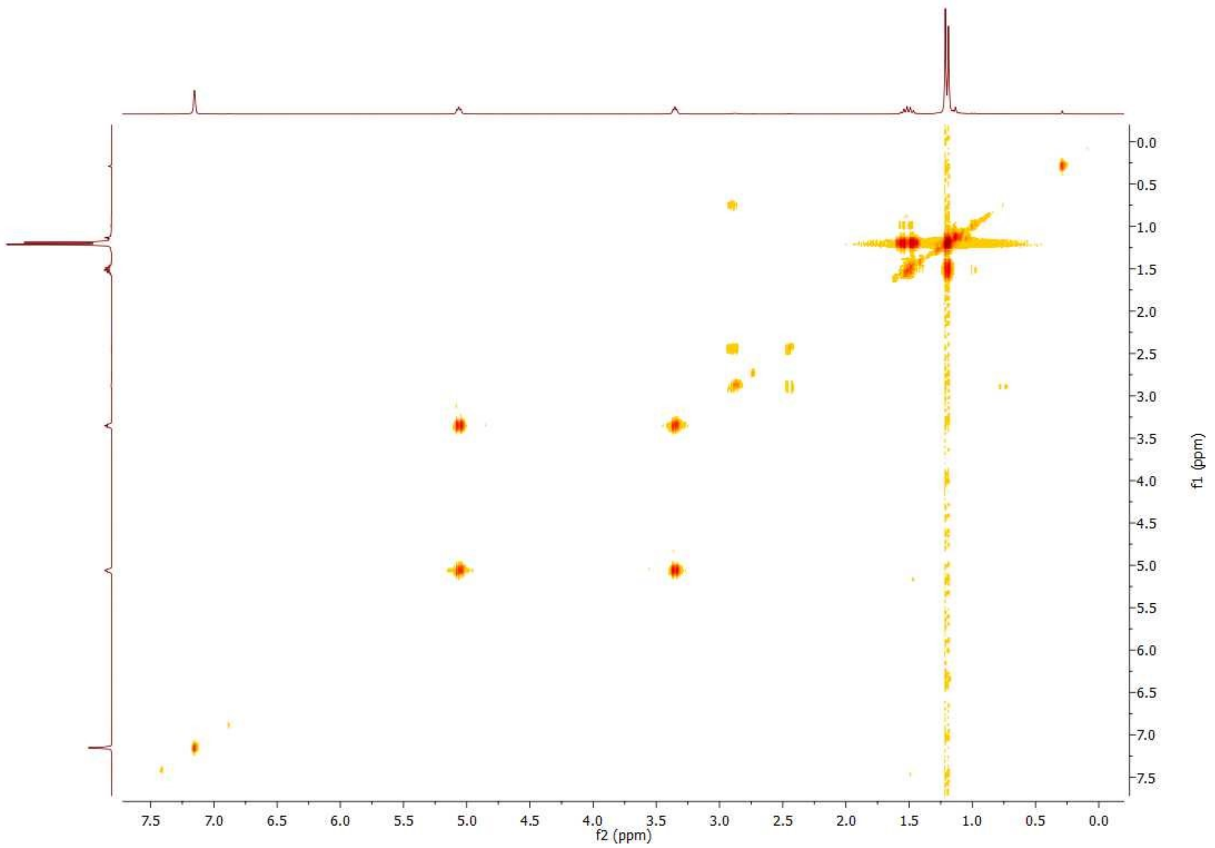

Figure S25. ^{1}H-^{1}H COSY spectrum of **2-Cl** in C_6D_6 at 298 K

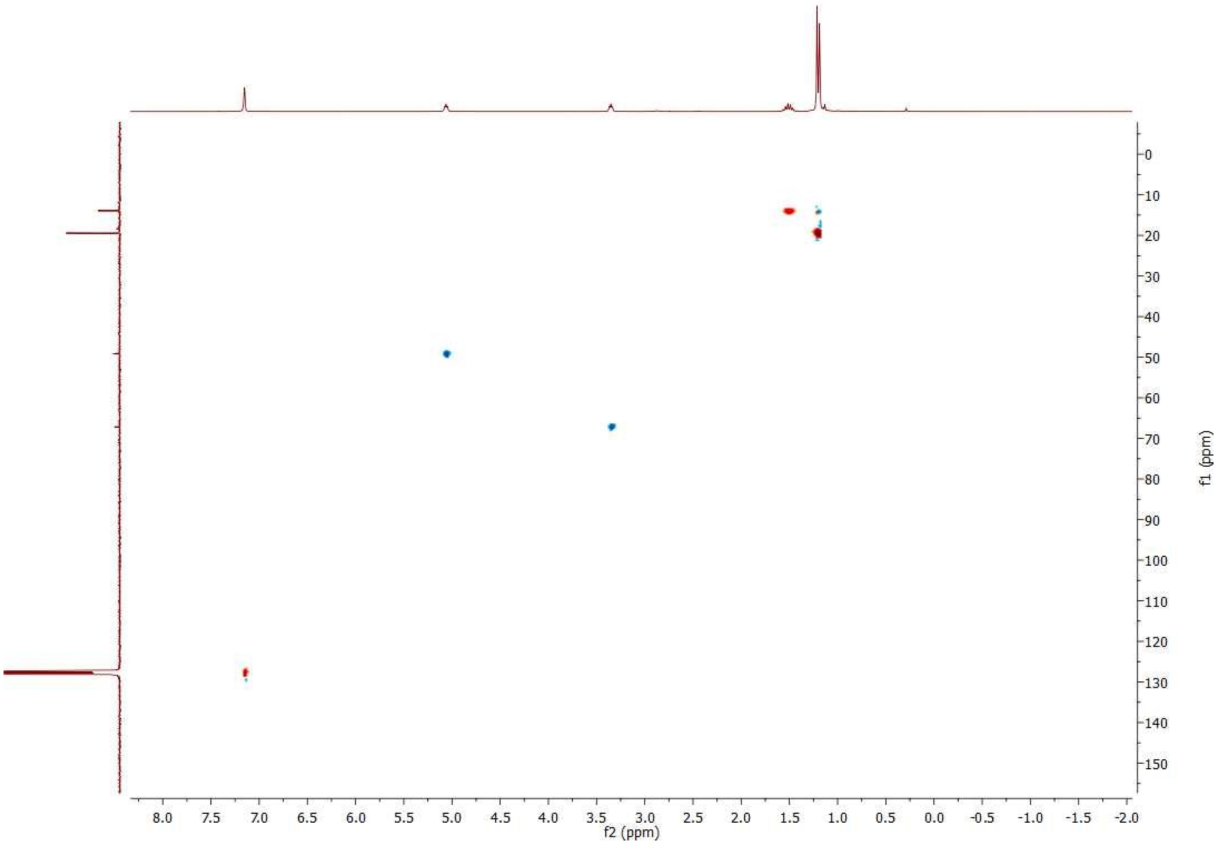

Figure S26. ^{1}H-^{13}C HSQC spectrum of **2-Cl** in C_6D_6 at 298 K

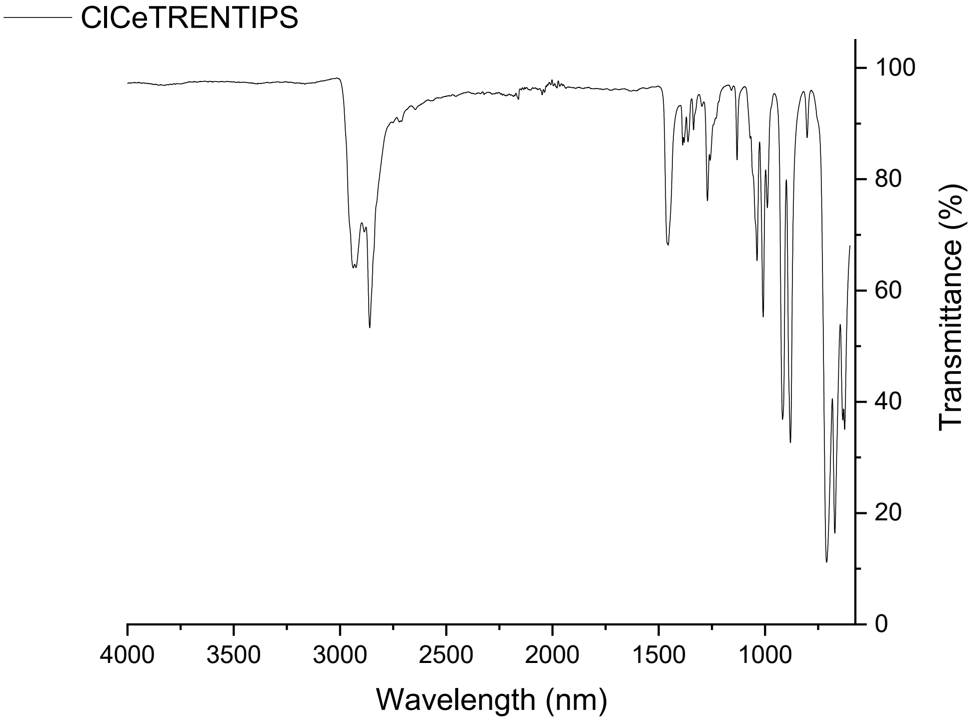

Figure S27. IR spectrum of **2-Cl** at 298 K

f) Spectral data for [BrCe^{IV}TREN^{TIPS}] (2-Br)

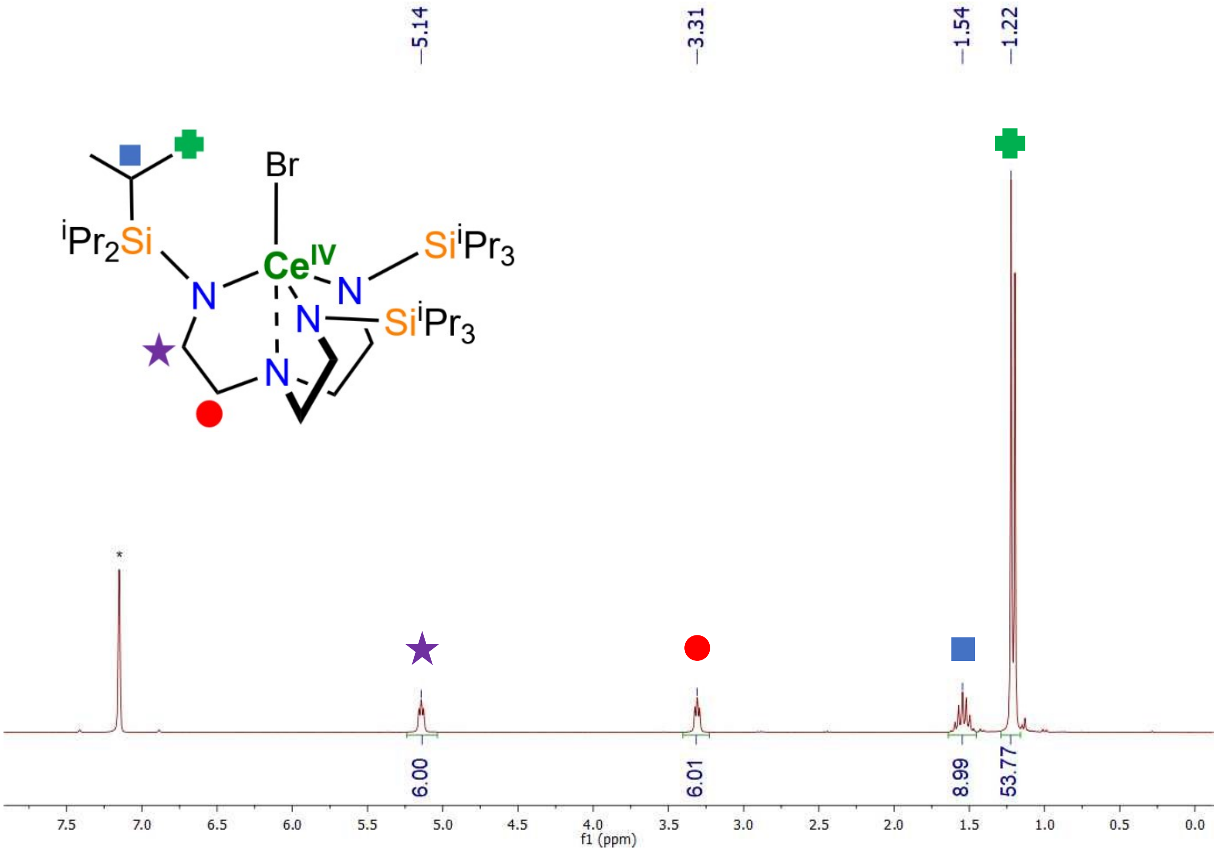

Figure S28. ^{1}H NMR spectrum of **2-Br** in C_6D_6 at 298 K. Solvent residual peak is indicated with *

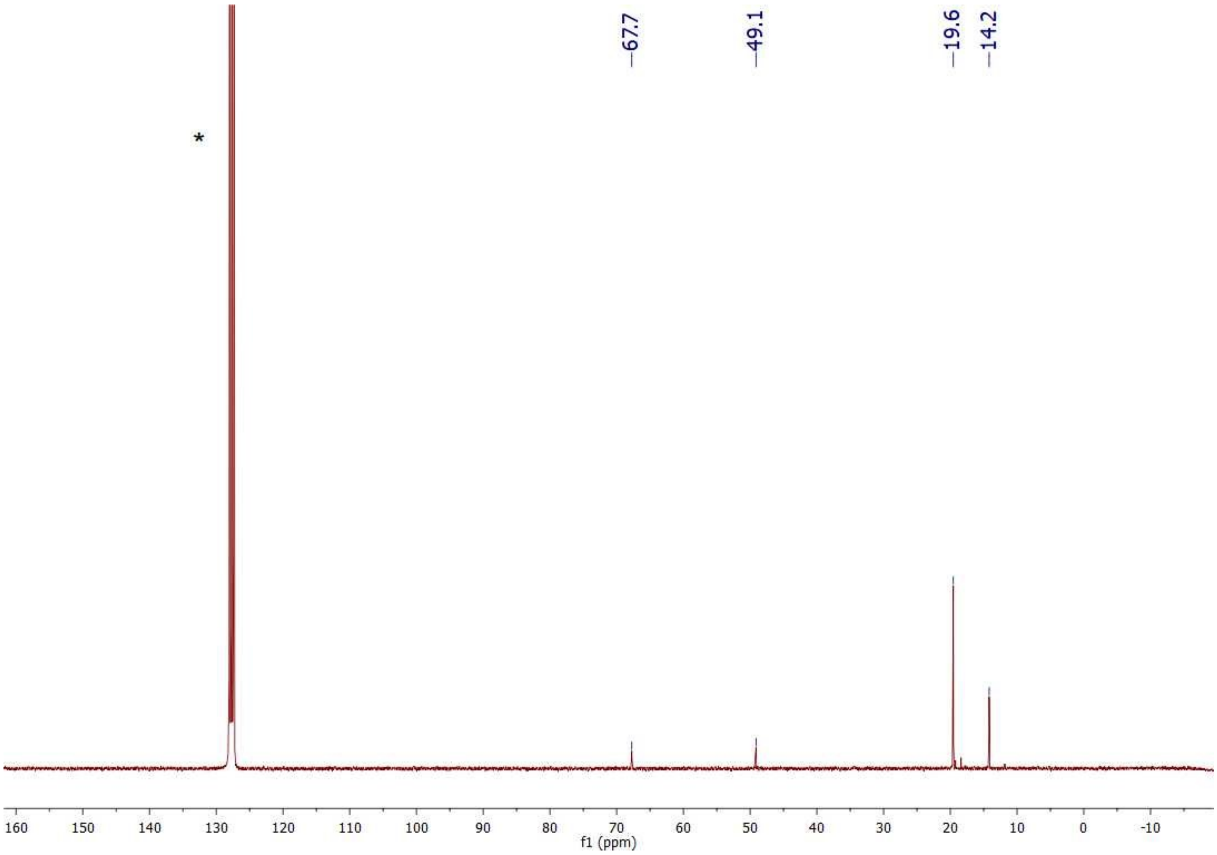

Figure S29. ^{13}C{^{1}H} NMR spectrum of **2-Br** in C_6D_6 at 298 K. Solvent residual peak is indicated with *

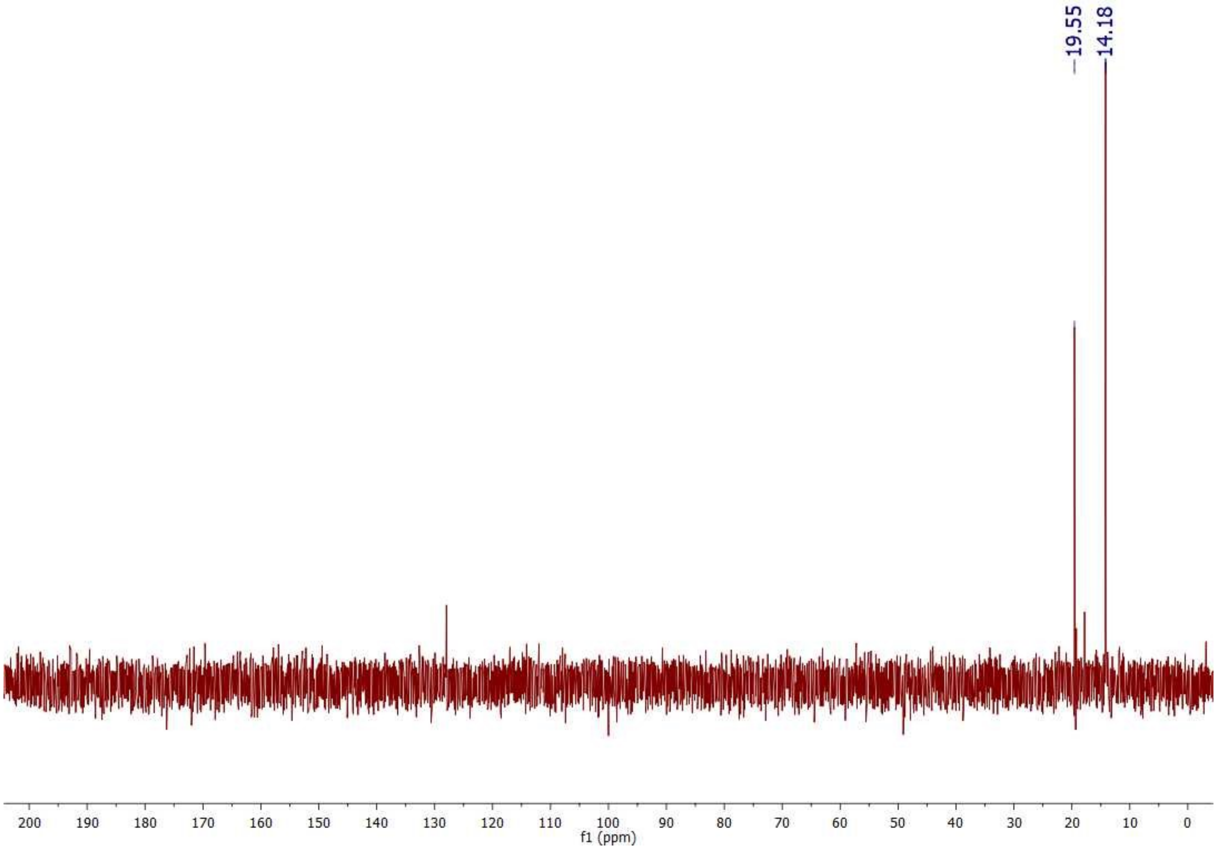

Figure S30. DEPT 135 $^{13}C\{^{1}H\}$ spectrum of **2-Br** in C_6D_6 at 298 K

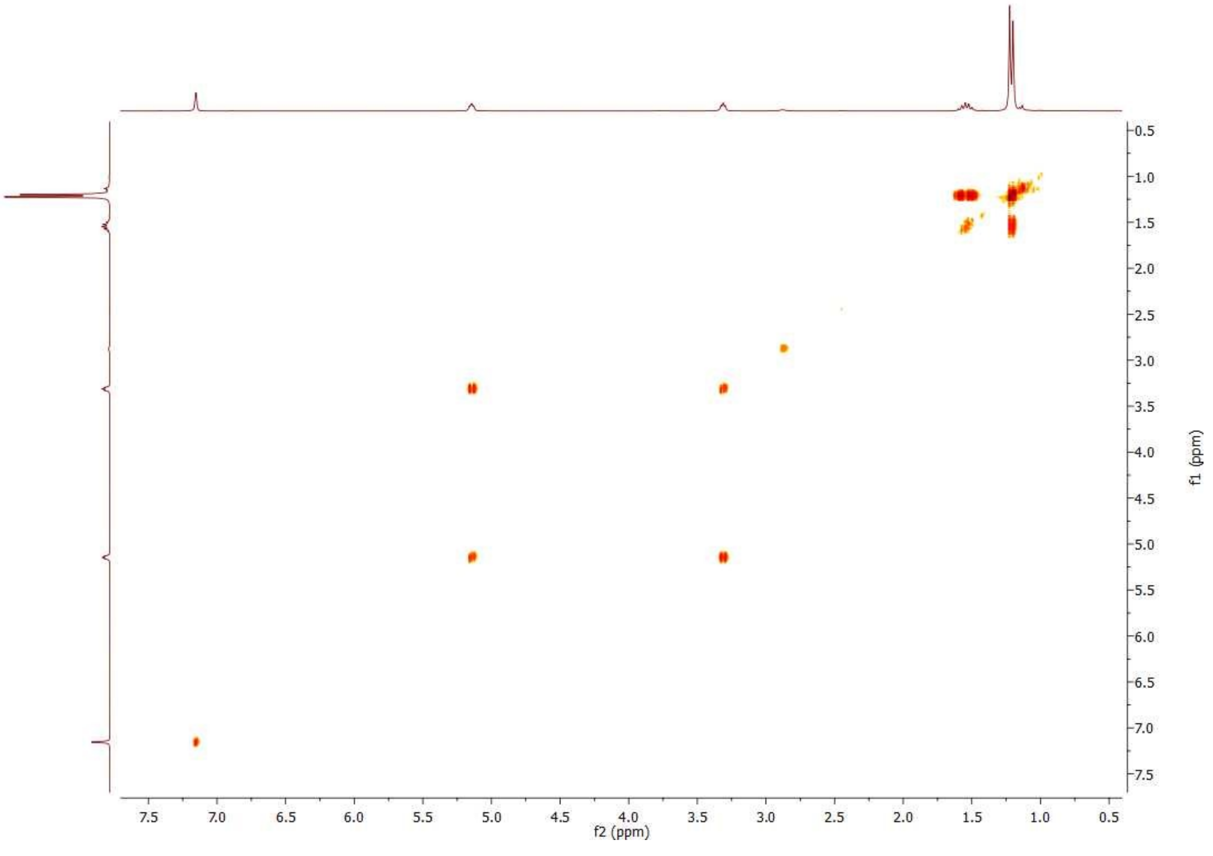

Figure S31. ^{1}H-^{1}H COSY spectrum of **2-Br** in C_6D_6 at 298 K

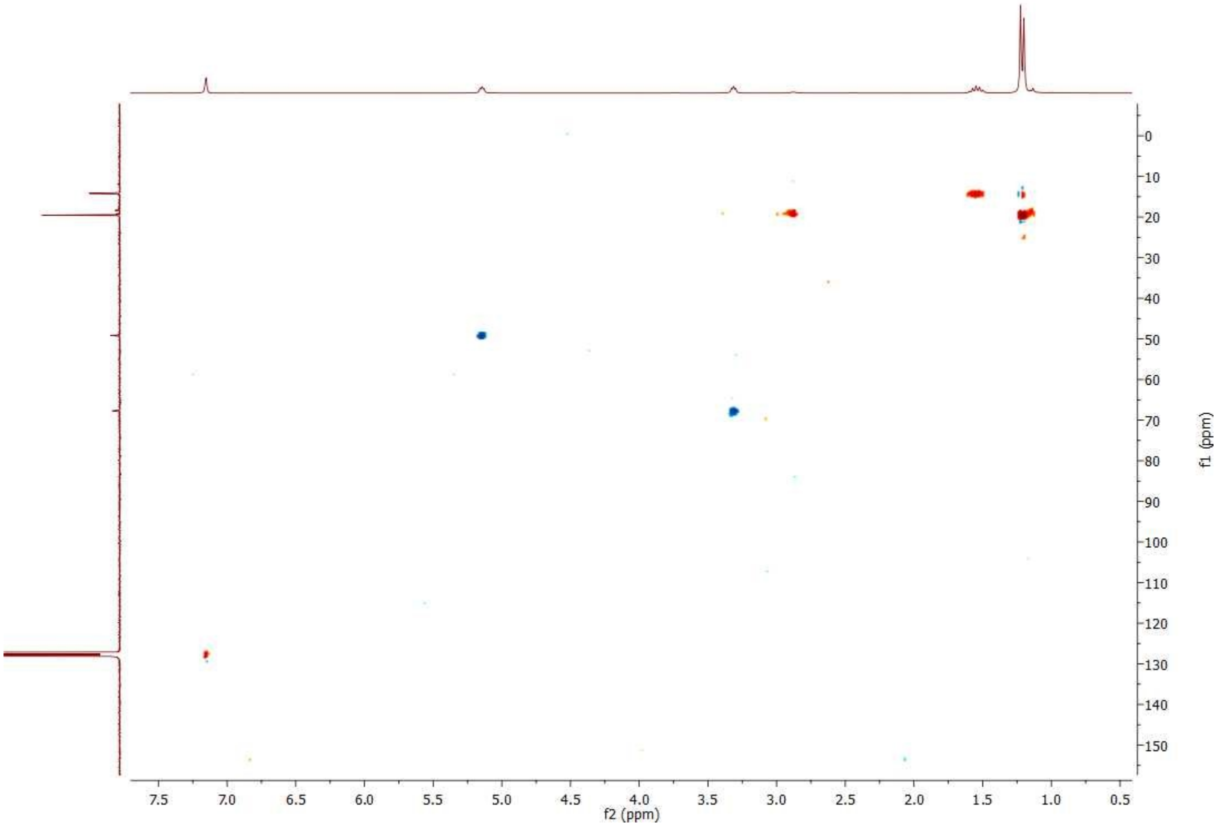

Figure S32. ^{1}H-^{13}C HSQC spectrum of **2-Br** in C_6D_6 at 298 K

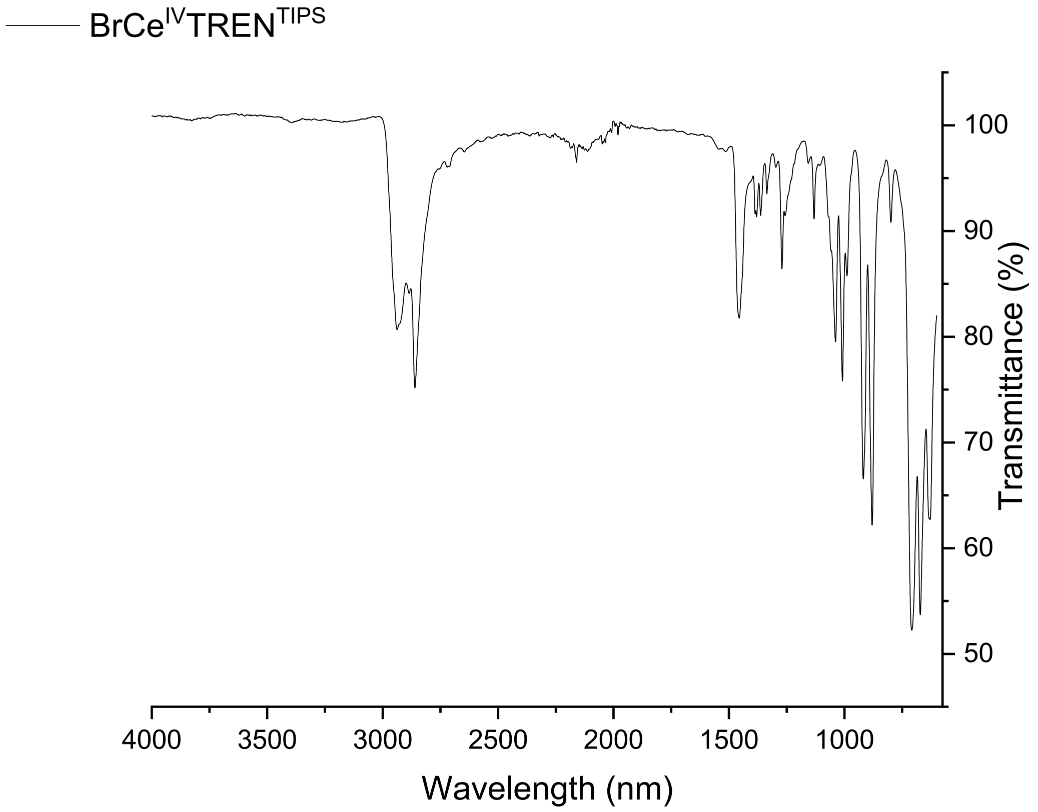

Figure S33. IR spectrum of **2-Br** at 298 K

g) Spectral data for [FCe^{IV}TREN^{TIPS}] (2-F)

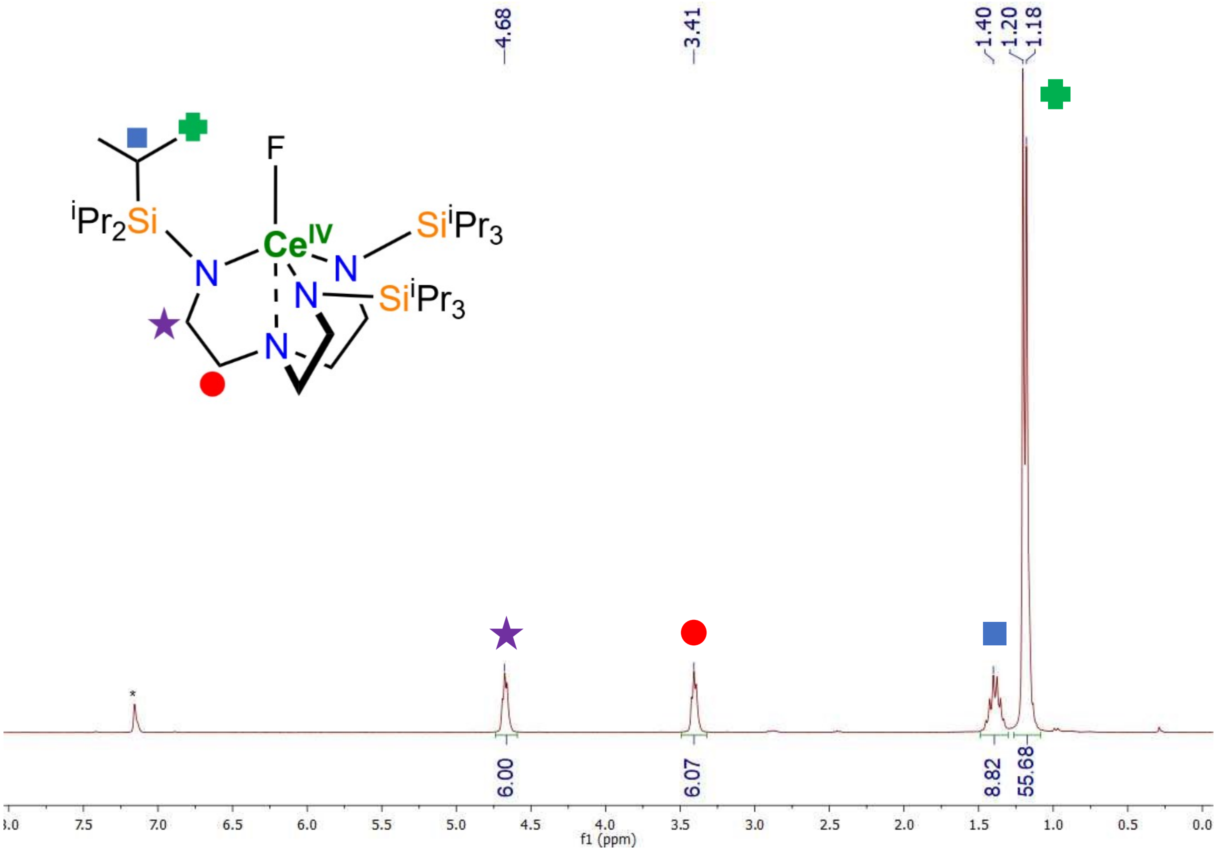

Figure S34. ^{1}H NMR spectrum of **2-F** in C_6D_6 at 298 K. Solvent residual peak is indicated with *

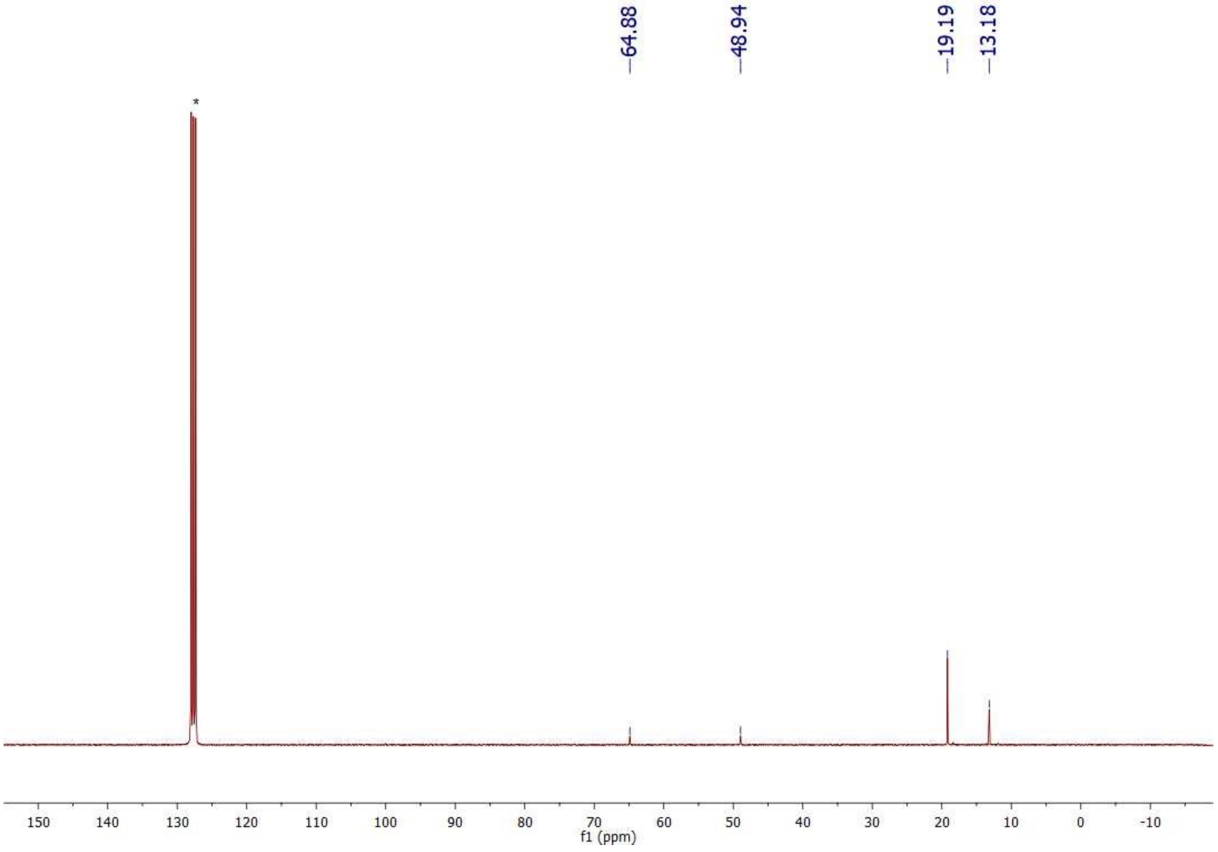

Figure S35. ^{13}C{^{1}H} NMR spectrum of **2-F** in C_6D_6 at 298 K. Solvent residual peak is indicated with *

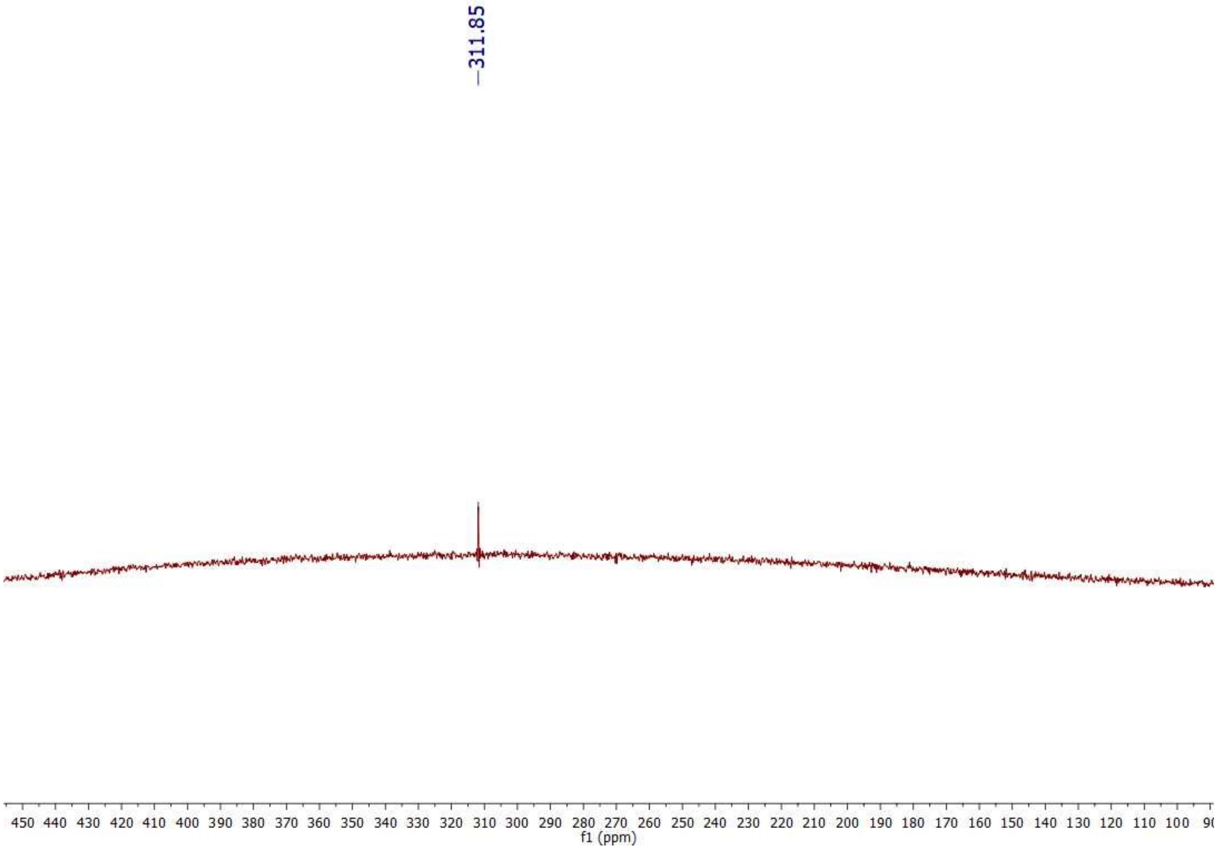

Figure S36. $^{19}F\{^1H\}$ NMR spectrum of **2-F** in THF-d_8 at 298 K

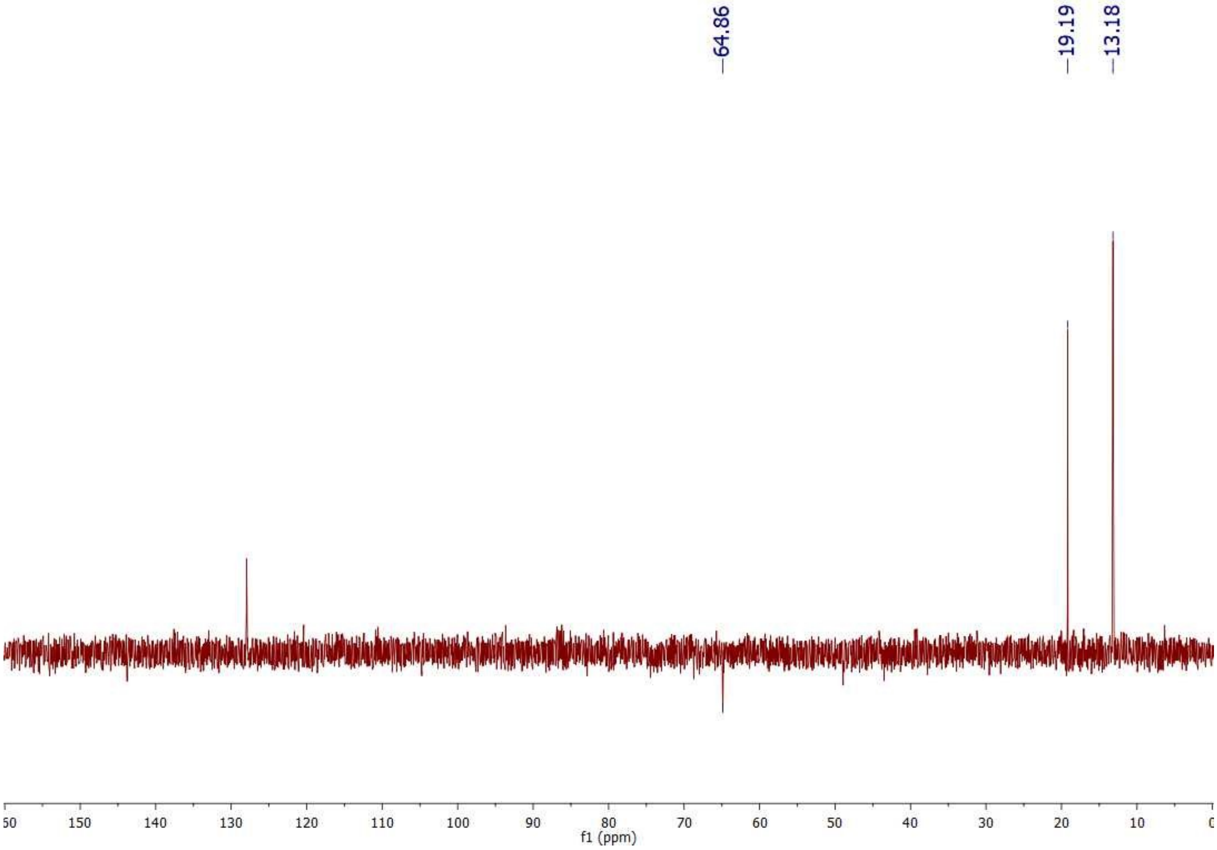

Figure S37. DEPT 135 $^{13}C\{^1H\}$ spectrum of **2-F** in C_6D_6 at 298 K. Solvent residual peak is indicated with *

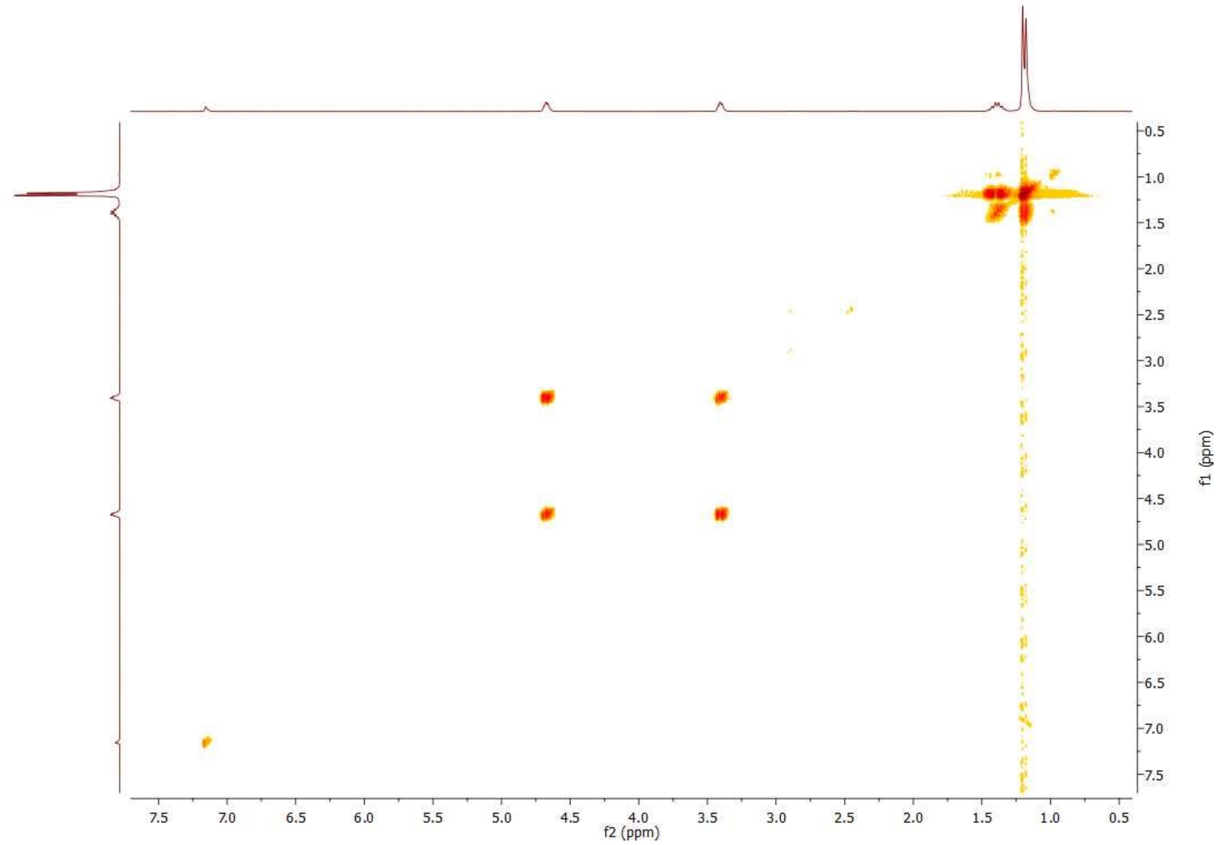

Figure S38. ^1H-^1H COSY spectrum of **2-F** in C_6D_6 at 298 K

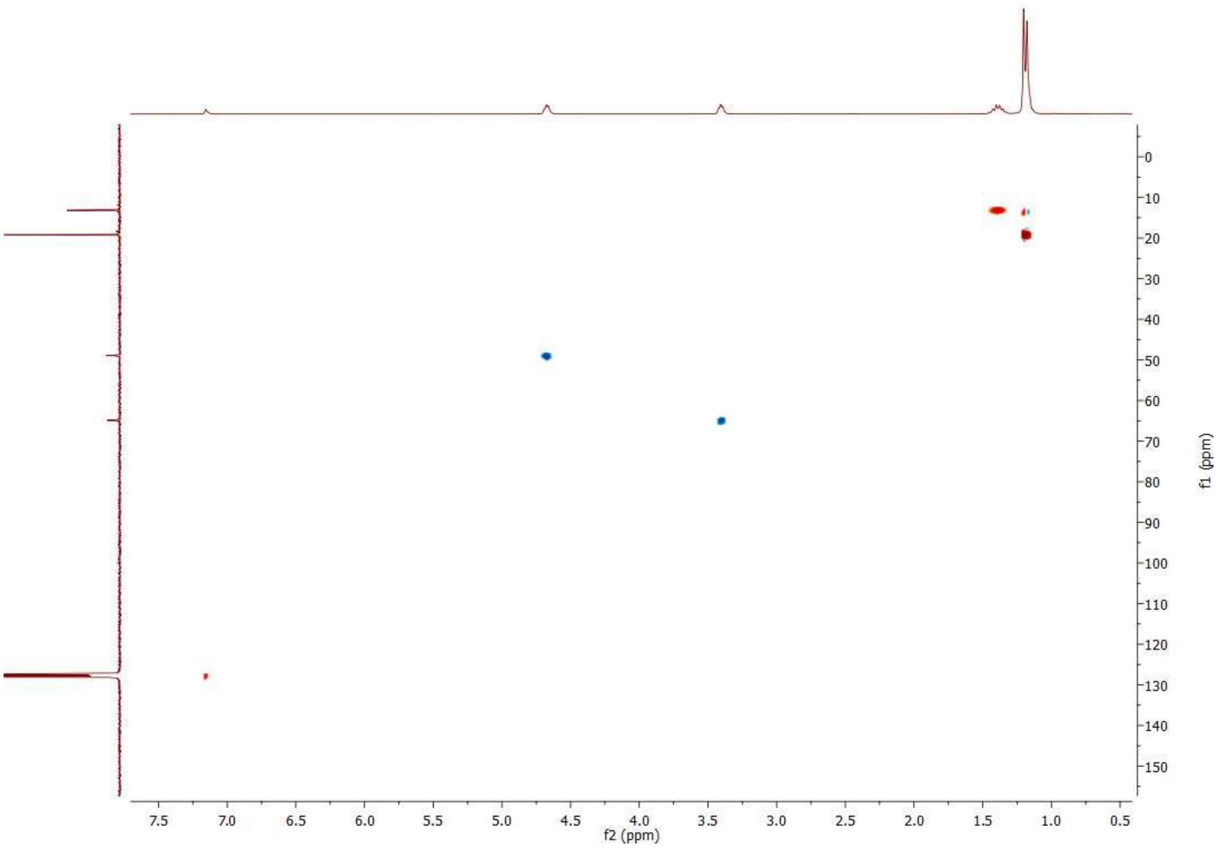

Figure S39. ^1H-^{13}C HSQC spectrum of **2-F** in C_6D_6 at 298 K

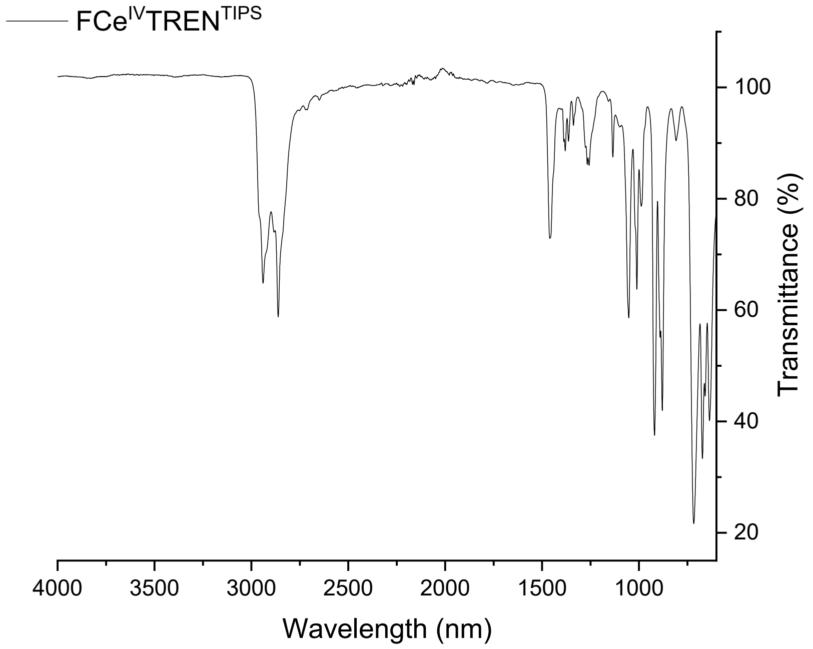

Figure S40. IR spectrum of **2-F** at 298 K

i) Spectra of 1-allyloxy-2-chlorobenzene

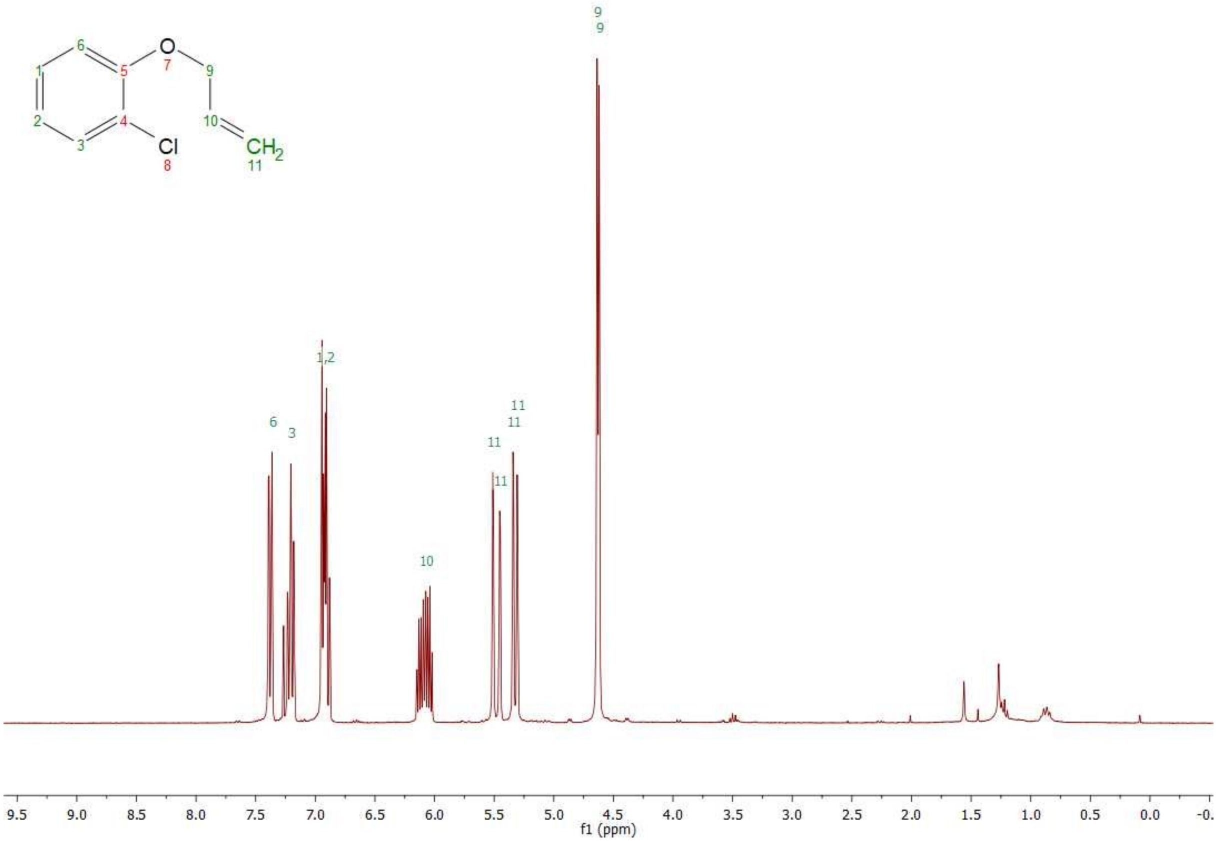

Figure S41. ^{1}H NMR of 1-allyloxy-2-chlorobenzene in $CDCl_3$ at 298 K

III. X-Ray crystal structures

Identification code	Li_3TREN^{TIPS}	$[Ce^{III}TREN^{TIPS}]$ (**1**)
Empirical formula	$C_{99}H_{224}Li_9N_{12}Si_9$	$C_{33}H_{75}CeN_4Si_3$
Formula weight	7592.63	752.36
Temperature/K	150	150
Crystal system	monoclinic	monoclinic
Space group	$P2_1/c$	$P2_1/n$
a/Å	31.680(2)	9.4990(8)
b/Å	16.5524(12)	22.531(2)
c/Å	23.4351(18)	19.0088(17)
α/°	90	90
β/°	96.896(6)	91.907(3)
γ/°	90	90
Volume/Å^3	12199.8(16)	4066.0(6)
Z	4	4
ρ_{calc} g/cm^3	1.033	1.229
μ/mm^{-1}	0.142	1.233
F(000)	4220.0	1604.0
Radiation	Mo K_α (λ = 0.71073)	Mo K_α (λ = 0.71073)
2Θ range for data collection/°	4.348 to 54.206	4.288 to 52.744
Index ranges	$-40 \le h \le 40, -19 \le k \le 21, -29 \le l \le 30$	$-11 \le h \le 11, -28 \le k \le 28, -21 \le l \le 23$
Reflections collected	86385	73441
Independent reflections	26598 [R_{int} = 0.0845, R_{sigma} = 0.1733]	8313 [R_{int} = 0.1032, R_{sigma} = 0.0572]
Data/restraints/parameters	26598/72/1216	8313/0/388
Goodness-of-fit on F2	1.041	0.977
Final R indexes [I>=2σ (I)]	R_1 = 0.1114, wR_2 = 0.3150	R_1 = 0.0318, wR_2 = 0.0729
Final R indexes [all data]	R_1 = 0.2424, wR_2 = 0.3756	R_1 = 0.0443, wR_2 = 0.0767
Largest diff. peak/hole / e Å^{-3}	1.84/-0.61	0.60/-1.12
CCDC number		2387384

Table S1. Crystal data and structure refinement for **Li_3TREN^{TIPS}** and **1**

**Identification code**	$[Li(THF)_4]$ $[ICe^{III}TREN^{TIPS}]$ (**1-LiI-THF$_4$**)	$[ClCe^{IV}TREN^{TIPS}]$ (**2-Cl**)
Empirical formula	$C_{49}H_{107}CeILiN_4O_4Si_3$	$C_{33}H_{75}CeClN_4Si_3$
Formula weight	1174.61	787.81
Temperature/K	150	150
Crystal system	orthorhombic	monoclinic
Space group	$Pna2_1$	$P2_1/n$
a/Å	17.7985(6)	12.9683(8)
b/Å	17.8628(8)	15.5387(9)
c/Å	19.3154(6)	20.3785(12)
α/°	90	90
β/°	90	90.572(5)
γ/°	90	90
Volume/Å^3	6141.0(4)	4106.3(4)
Z	4	4
ρ_{calc} g/cm^3	1.270	1.274
μ/mm^{-1}	1.342	1.287
F(000)	2468.0	1672.0
Radiation	Mo K_α (λ = 0.71073)	Mo Kα (λ = 0.71073)
2Θ range for data collection/°	4.56 to 52.742	4.54 to 52.04
Index ranges	-22≤ h ≤ 21, -22 ≤ k ≤ 22, -24 ≤ l ≤ 24	-15 ≤ h ≤ 16, -19 ≤ k ≤ 18, -14 ≤ l ≤ 25
Reflections collected	42273	31438
Independent reflections	11846 [R_{int} = 0.0214, R_{sigma} = 0.0274]	8013 [R_{int} = 0.0760, R_{sigma} = 0.1053]
Data/restraints/parameters	11846/136/619	8013/0/397
Goodness-of-fit on F2	1.085	0.910
Final R indexes [I>=2σ (I)]	R_1 = 0.0331, wR_2 = 0.0849	R_1 = 0.0378, wR_2 = 0.0667
Final R indexes [all data]	R_1 = 0.0417, wR_2 = 0.0888	R_1 = 0.0806, wR_2 = 0.0737
Largest diff. peak/hole / e Å^{-3}	1.11/-0.92	0.72/-0.42
CCDC number	2387121	2387124

Table S2. Crystal data and structure refinement for **1-LiI-THF$_4$** and **1-^{t}BuCN**

**Identification code**	[$BrCe^{IV}TREN^{TIPS}$] (**2-Br**)	[$FCe^{IV}TREN^{TIPS}$] (**2-F**)
Empirical formula	$C_{33}H_{75}CeBrN_4Si_3$	$C_{264}H_{600}Ce_8F_8N_{32}Si_{24}$
Formula weight	832.27	6170.84
Temperature/K	150	150
Crystal system	monoclinic	monoclinic
Space group	$P2_1/n$	$P2_1/n$
a/Å	13.0115(10)	20.3562(10)
b/Å	15.7902(9)	19.9580(9)
c/Å	20.3365(17)	20.6752(11)
α/°	90	90
β/°	90.712(7)	103.739(4)
γ/°	90	90
Volume/Å^3	4177.9(5)	8159.4(7)
Z	4	1
ρ_{calc} g/cm^3	1.323	1.256
μ/mm^{-1}	2.158	1.234
F(000)	1744.0	3280.0
Radiation	Mo K_α (λ = 0.71073)	Mo K_α (λ = 0.71073)
2Θ range for data collection/°	4.506 to 56.564	4.54 to 61.288
Index ranges	$-17 \leq h \leq 17$, $-16 \leq k \leq 21$, $-27 \leq l \leq 26$	$-29 \leq h \leq 27$, $-22 \leq k \leq 28$, $-29 \leq l \leq 29$
Reflections collected	113832	77535
Independent reflections	10348 [R_{int} = 0.0524, R_{sigma} = 0.0264]	22744 [R_{int} = 0.0287, R_{sigma} = 0.0274]]
Data/restraints/parameters	10348/0/413	22744/0/1214
Goodness-of-fit on F2	1.066	1.035
Final R indexes [I>=2σ (I)]	R_1 = 0.0280, wR_2 = 0.0624	R_1 = 0.0371, wR_2 = 0.0869
Final R indexes [all data]	R_1 = 0.0434, wR_2 = 0.0677	R_1 = 0.0609, wR_2 = 0.0998
Largest diff. peak/hole / e Å^{-3}	0.87/-0.42	0.68/-0.87
CCDC number	2387123	2387125

Table S3. Crystal data and structure refinement for **2-Br** and **2-F**

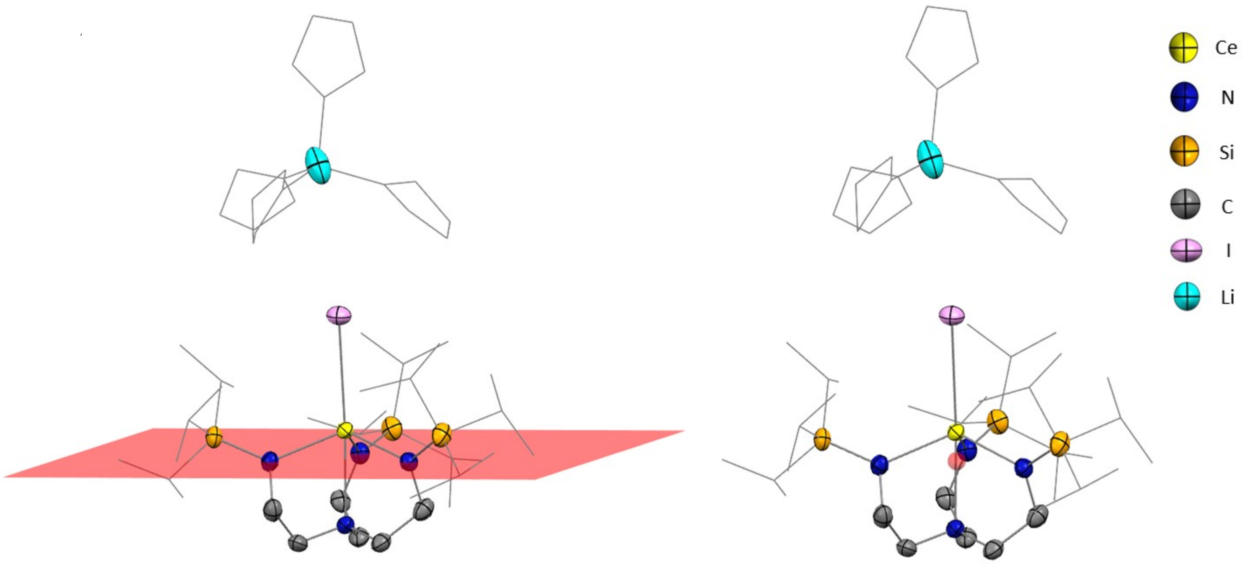

Figure S42. Position of plane (A) and centroid (B) used for distance and bond measurements on **1-LiI-THF$_4$**, [Li(THF)$_4$][ICe^{III}TREN^{TIPS}].

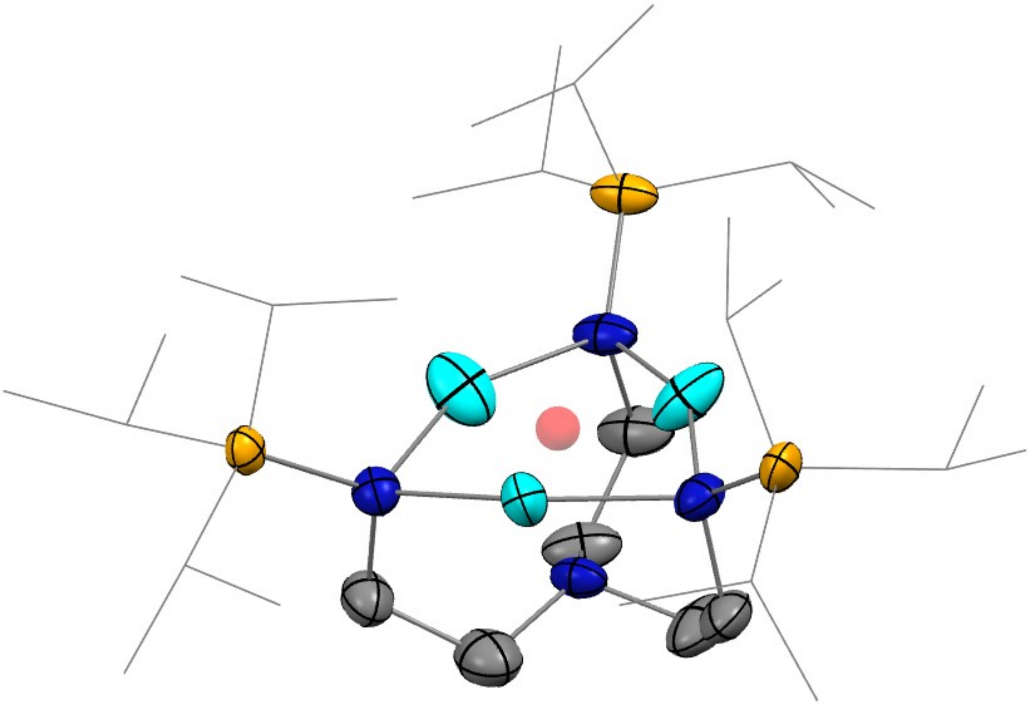

Figure S43. Position of the centroid defined by the three lithium ions in **Li$_3$TREN^{TIPS}**

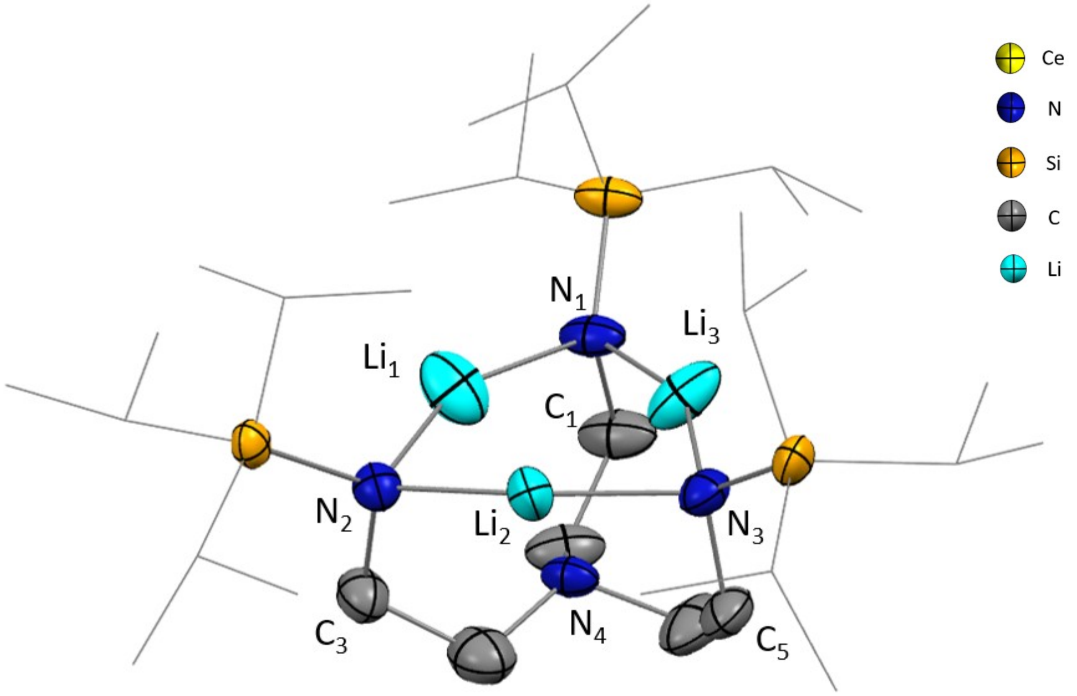

Figure S44. Molecular structure of **Li_3TREN^{TIPS}**. Displacement ellipsoids are represented at 50% probability. Hydrogen atoms have been omitted for clarity, and isopropyl groups have been represented in wireframe style. Three molecules are present per asymmetric unit, but only one was shown for clarity. Values given in **Table S4** were selected on one of the molecules.

Bond	Length (Å)
Cent-N_1	2.163
Cent-N_2	2.183
Cent-N_3	2.273
Cent-N_4	2.045
N_1-Si_1	1.716(5)
N_2-Si_2	1.706(5)
N_3-Si_3	1.709(5)
N_1-C_1	1.488(7)
N_2-C_3	1.480(6)
N_3-C_5	1.484(7)
N_1-Li_1	1.970(11)
N_1-Li_3	1.965(11)
N_2-Li_1	1.947(12)
N_2-Li_2	1.990(12)
N_3-Li_2	1.976(13)
N_3-Li_3	1.966(12)

Bonds	Angle (°)
C_1-N_1-Cent	104.00
C_3-N_2-Cent	103.64
C_5-N_3-Cent	105.07

Bond	Mean value (Å)
Cent-$N_{equatorial}$	2.206
N-Si	1.710(9)

Table S4. Selected bond lengths and angles for **Li_3TREN^{TIPS}**

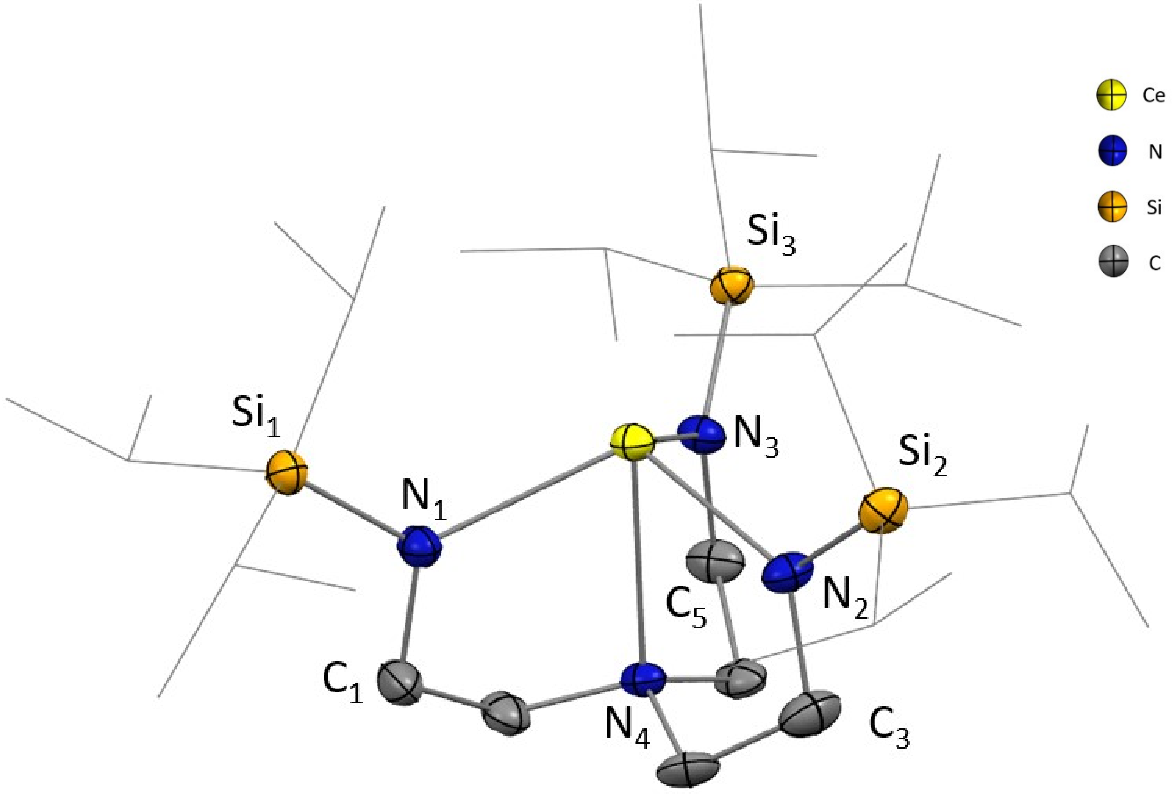

Figure S45. Molecular structure of **1**. Displacement ellipsoids are represented at 50% probability. Hydrogen atoms have been omitted for clarity, and isopropyl groups have been represented in wireframe style.

Bond	Length (Å)	Bonds	Angle (°)
Ce-N_1	2.366(2)	C_1-N_1-Ce	119.34(16)
Ce-N_2	2.358(2)	C_3-N_2-Ce	118.64(17)
Ce-N_3	2.359(2)	C_5-N_3-Ce	119.23(17)
Ce-N_4	2.534(2)	Ce-Cent-N_4	177.1
N_1-Si_1	1.716(2)		
N_2-Si_2	1.724(2)		
N_3-Si_3	1.720(2)	**Bond**	**Mean value (Å)**
N_1-C_1	1.469(3)	Ce-$N_{equatorial}$	2.361(3)
N_2-C_3	1.464(3)	Ce-N_{plane}	0.798
N_3-C_5	1.466(3)	N-Si	1.720(3)

Table S5. Selected bond lengths and angles for **1**

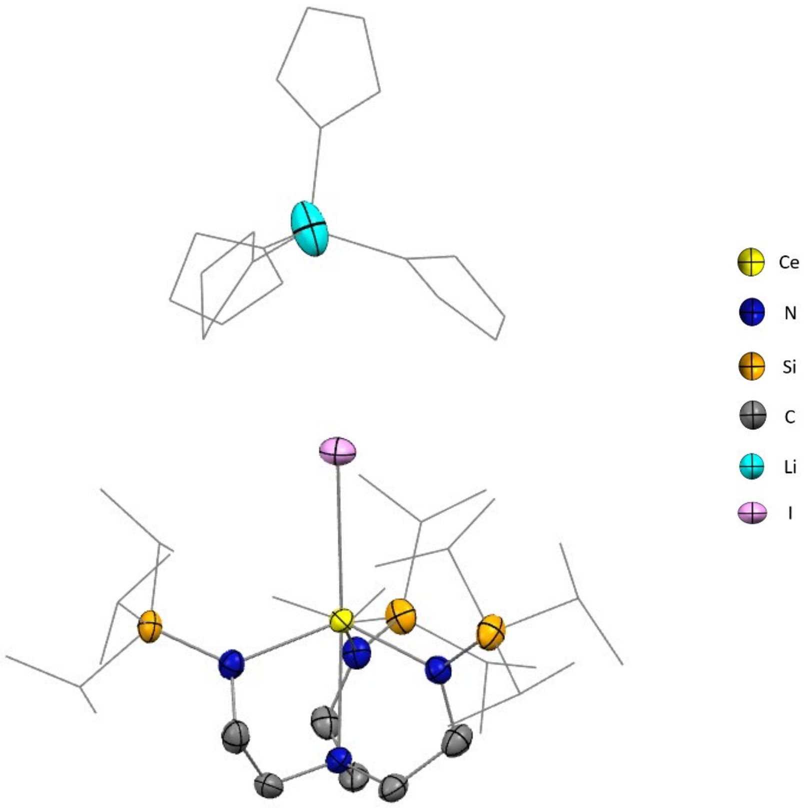

Figure S46. Structure of $[Li(THF)_4][ICe^{III}TREN^{TIPS}]$ (**1-LiI-THF$_4$**). Displacement ellipsoids are represented at 50% probability. Hydrogen atoms have been omitted for clarity, and isopropyl groups have been represented in wireframe style. The molecule presents a slight disorder on the isopropyl ligands which was modelled but not plotted for clarity.

Bond	Length (Å)	Bonds	Angle (°)
Ce-N_1	2.380(5)	C_1-N_1-Ce	114.2(4))
Ce-N_2	2.374(5)	C_3-N_2-Ce	110.7(3)
Ce-N_3	2.364(5)	C_5-N_3-Ce	112.7(4)
Ce-N_4	2.664(5)	Ce-Cent-N_4	172.0(3)
N_1-Si_1	1.720(5)		
N_2-Si_2	1.705(5)		
N_3-Si_3	1.711(5)		
N_1-C_1	1.453(9)		
N_2-C_3	1.478(8)	**Bond**	**Mean value (Å)**
N_3-C_5	1.472(9)	Ce-$N_{equatorial}$	2.273(2)
Ce-I	3.2580(5)	Ce-N_{plane}	0.809(5)
Li-I	4.343	N-Si	1.712(1)

Table S6. Selected bond lengths and angles for **1-LiI-THF$_4$**, $[Li(THF)_4][ICe^{III}TREN^{TIPS}]$.

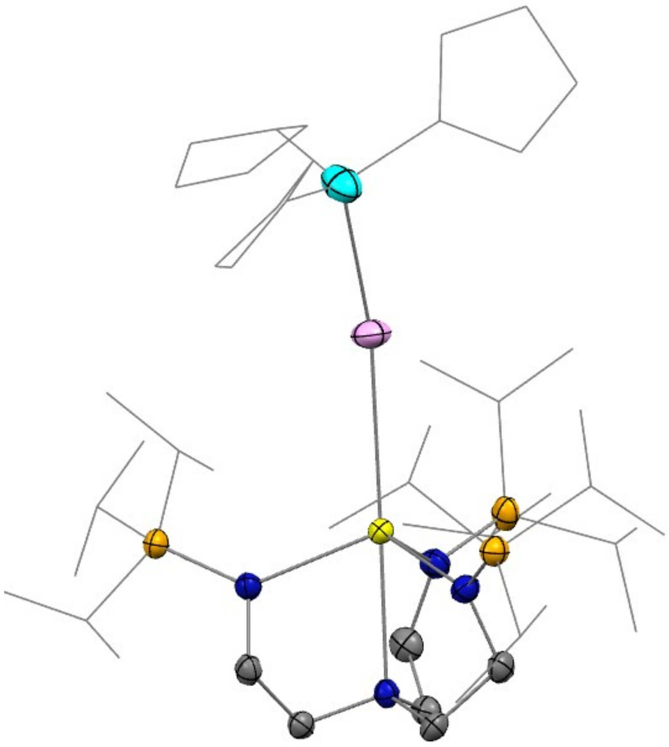

Figure S47. Structure of $[Li(THF)_3][ICe^{III}TREN^{TIPS}]$ (1-LiI-THF$_3$). Displacement ellipsoids are represented at 50% probability. Hydrogen atoms have been omitted for clarity, and isopropyl groups have been represented in wireframe style. The molecule presents a slight disorder on the isopropyl ligands. The disorder was modeled but not plotted for clarity.

Bond	Length (Å)	Bonds	Angle (°)
Ce-N$_1$	2.349(3)	C$_1$-N$_1$-Ce	111.4(2)
Ce-N$_2$	2.357(3)	C$_3$-N$_2$-Ce	110.9(2)
Ce-N$_3$	2.380(3)	C$_5$-N$_3$-Ce	111.4(2)
Ce-N$_4$	2.670(3)	Ce-Cent-N$_4$	176.0(2)
N$_1$-Si$_1$	1.719(3)		
N$_2$-Si$_2$	1.715(3)		
N$_3$-Si$_3$	1.707(4)		
N$_1$-C$_1$	1.469(4)		
N$_2$-C$_3$	1.477(5)	Bond	Mean value (Å)
N$_3$-C$_5$	1.471(5)	Ce-N$_{equatorial}$	2.362(2)
Ce-I	3.2810(4)	Ce-N$_{plane}$	0.803(3)
Li-I	2.767(8)	N-Si	1.714(1)

Table S7. Selected bond lengths and angles for 1-LiI-THF$_3$, $[Li(THF)_3][ICe^{III}TREN^{TIPS}]$.

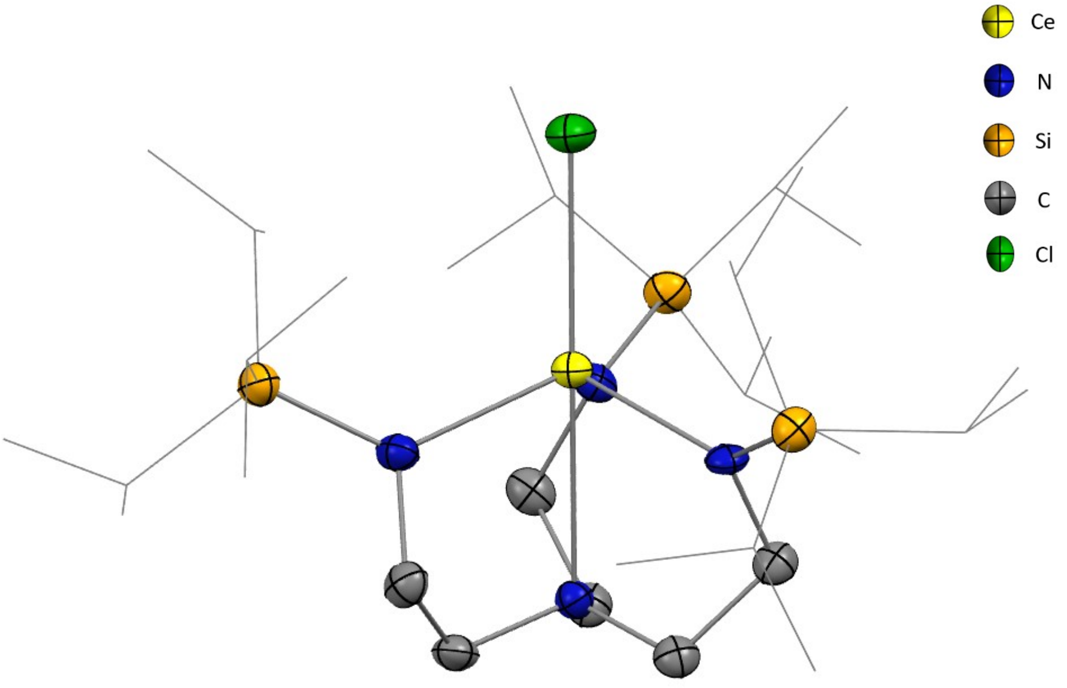

Figure S48. Structure of **2-Cl**. Displacement ellipsoids are represented at 50% probability. Hydrogen atoms have been omitted for clarity, and isopropyl groups have been represented in wireframe style. The molecule presents a slight disorder on the isopropyl ligands, which was modelled but not plotted for clarity.

Bond	Length (Å)	Bonds	Angle (°)
Ce-N_1	2.202(3)	C_1-N_1-Ce	109.39(19)
Ce-N_2	2.225(3)	C_3-N_2-Ce	113.8(2)
Ce-N_3	2.233(3)	C_5-N_3-Ce	111.4(2)
Ce-N_4	2.606(3)	Ce-Cent-N_4	177.4
N_1-Si_1	1.746(3)		
N_2-Si_2	1.747(3)		
N_3-Si_3	1.736(3)		
N_1-C_1	1.476(5)		
N_2-C_3	1.479(5)		
N_3-C_5	1.480(5)		
Ce-Cl	2.625(11)		

Bond	Mean value (Å)
Ce-$N_{equatorial}$	2.220(3)
Ce-N_{plane}	0.694
N-Si	1.743(2)

Table S8. Selected bond lengths and angles for **2-Cl**.

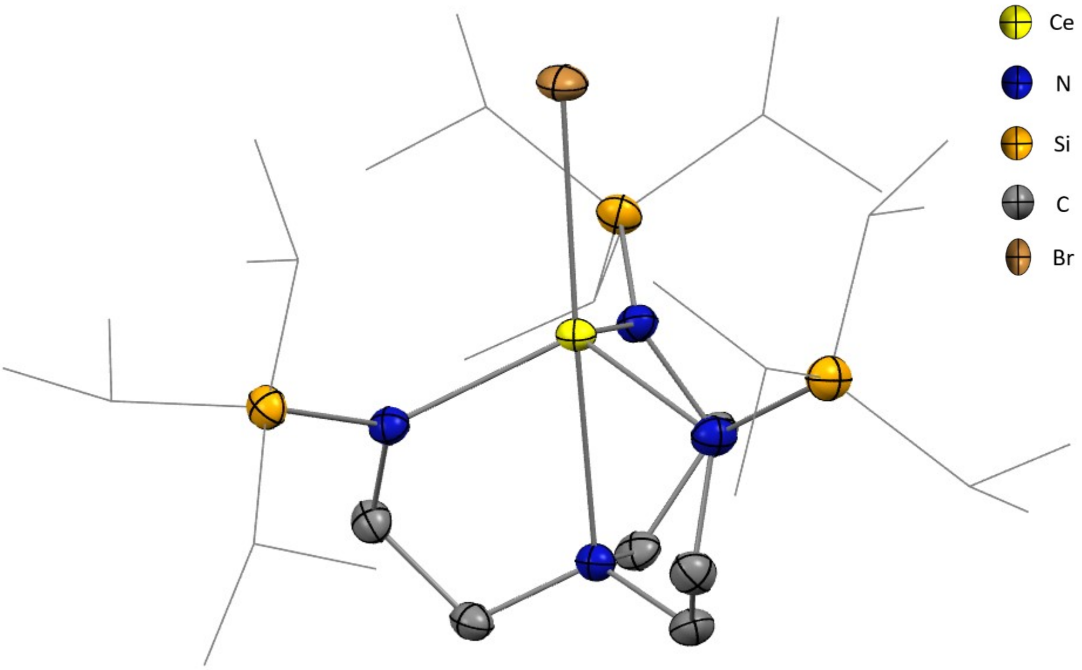

Figure S49. Structure of **2-Br**. Displacement ellipsoids are represented at 50% probability. Hydrogen atoms have been omitted for clarity, and isopropyl groups have been represented in wireframe style. The molecule presents a slight disorder on the isopropyl ligands. The disorder was modelled but not plotted for clarity.

Bond	Length (Å)	Bonds	Angle (°)
Ce-N_1	2.229(2)	C_1-N_1-Ce	113.43(15)
Ce-N_2	2.232(2)	C_3-N_2-Ce	111.31(14)
Ce-N_3	2.206(2)	C_5-N_3-Ce	109.03(15)
Ce-N_4	2.603(2)	Ce-Cent-N_4	177.8
N_1-Si_1	1.743(2)		
N_2-Si_2	1.743(2)		
N_3-Si_3	1.750(2)		
N_1-C_1	1.481(3)	**Bond**	**Mean value (Å)**
N_2-C_3	1.483(3)	Ce-$N_{equatorial}$	2.222(5)
N_3-C_5	1.473(3)	Ce-N_{plane}	0.687
Ce-Br	2.785(3)	N-Si	1.754(3)

Table S9. Selected bond lengths and angles for **2-Br.**

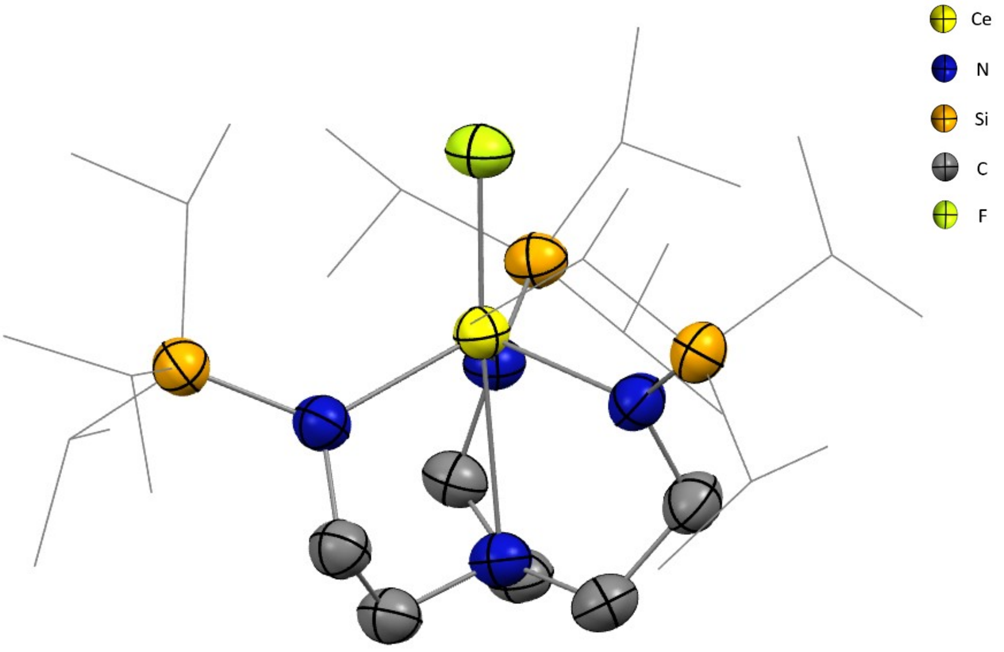

Figure S50. Structure of **2-F.** Displacement ellipsoids are represented at 50% probability. Hydrogen atoms have been omitted for clarity, and isopropyl groups have been represented in wireframe style. The molecule presents a slight disorder on the isopropyl ligands. The disorder was modelled but not plotted for clarity. NB : two molecules are present in the asymmetric unit, but only one was displayed here.

Bond	Length (Å)	Bonds	Angle (°)
Ce-N_1	2.227(3)	C_1-N_1-Ce	114.3(2)
Ce-N_2	2.223(3)	C_3-N_2-Ce	111.9(2)
Ce-N_3	2.235(2)	C_5-N_3-Ce	116.21(19)
Ce-N_4	2.620(3)	Ce-Cent-N_4	174.42
N_1-Si_1	1.734(3)		
N_2-Si_2	1.737(3)		
N_3-Si_3	1.738(3)		
N_1-C_1	1.482(4)	**Bond**	**Mean value (Å)**
N_2-C_3	1.477(4)	Ce-$N_{equatorial}$	2.222(4)
N_3-C_5	1.467(4)	Ce-N_{plane}	0.722
Ce-F	2.0985(18)	N-Si	1.736(5)

Table S10. Selected bond lengths and angles for **2-F**.

IV. Magnetic measurements

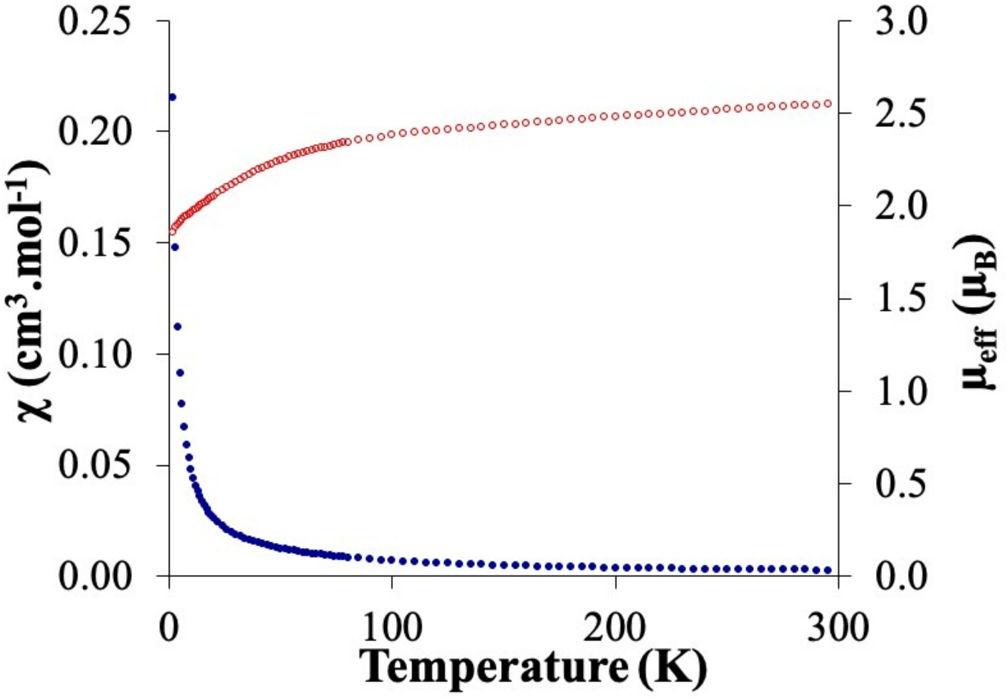

Figure S51. Solid-state temperature-dependent magnetic data of **1** recorded at 0.5 T, χ *vs.* T plot in blue and μ_{eff} *vs.* T plot in red. Data plot by Eric Rivière.

V. EPR measurements for Chapter I

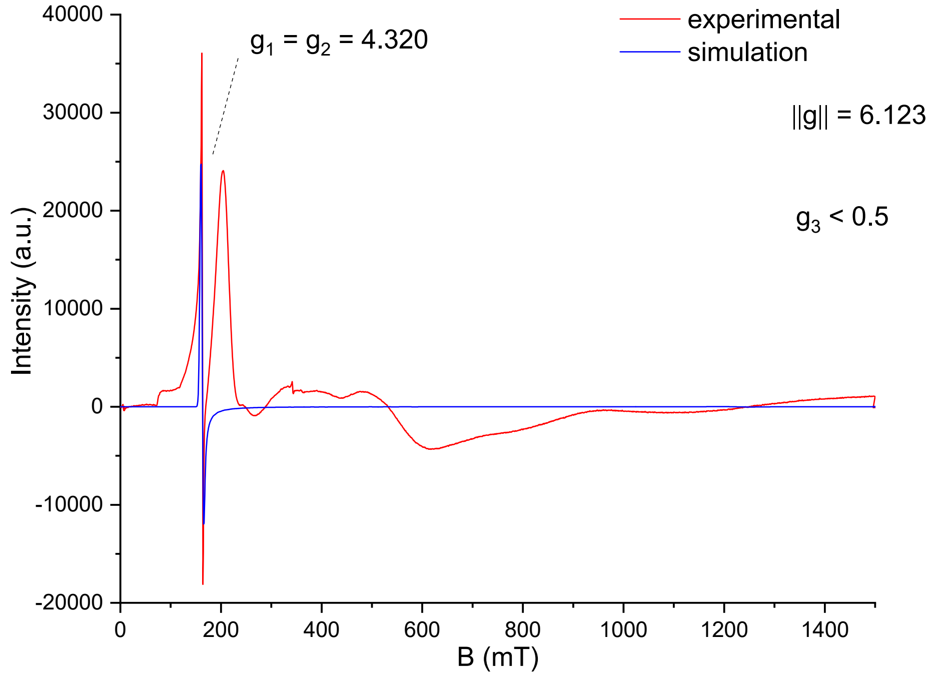

Figure S52. X-band EPR spectrum of powder of **1** at 10 K. Simulation of the "axial" system

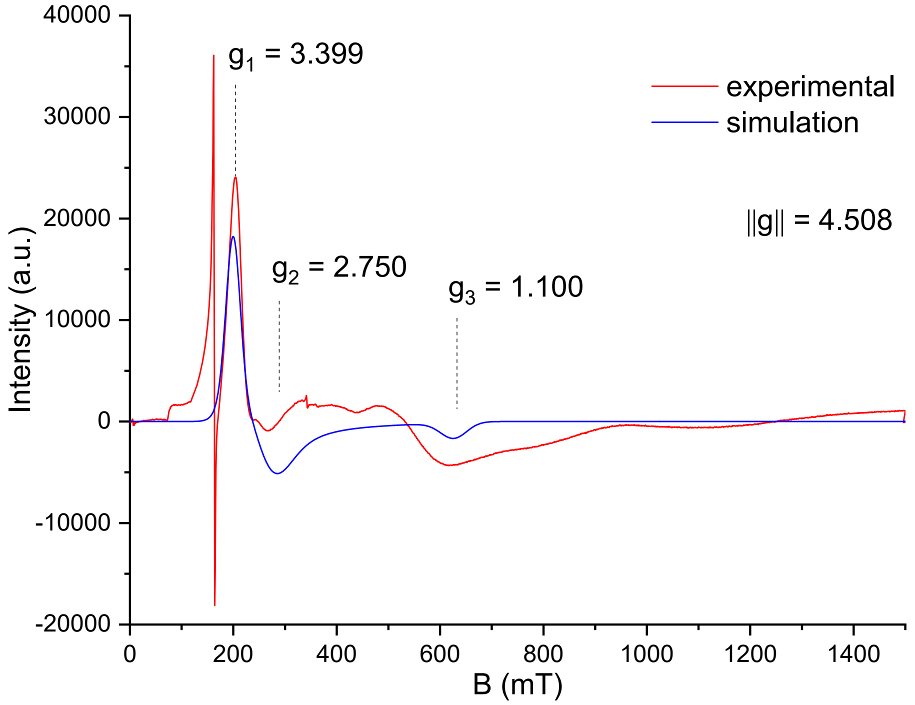

Figure S53. X-band EPR spectrum of powder of **1** at 10 K. Simulation of the second system

VI. UV-visible and fluorescence spectra

a) Experimental data

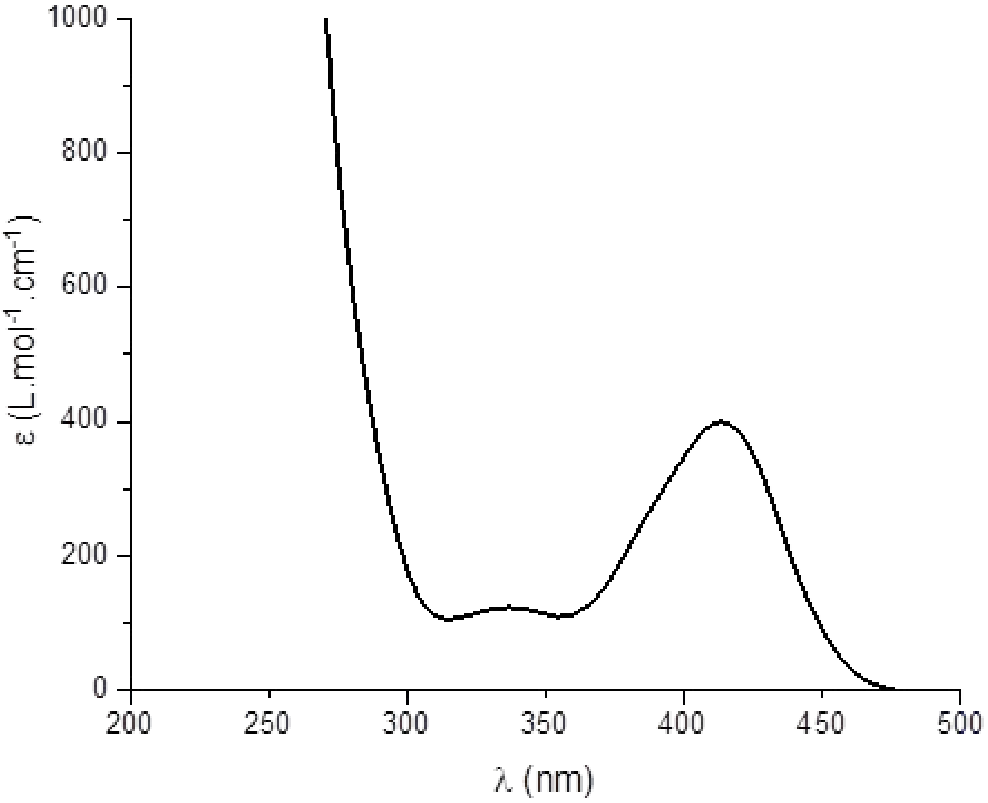

Figure S54. UV-visible spectrum of **1** in pentane at 298 K

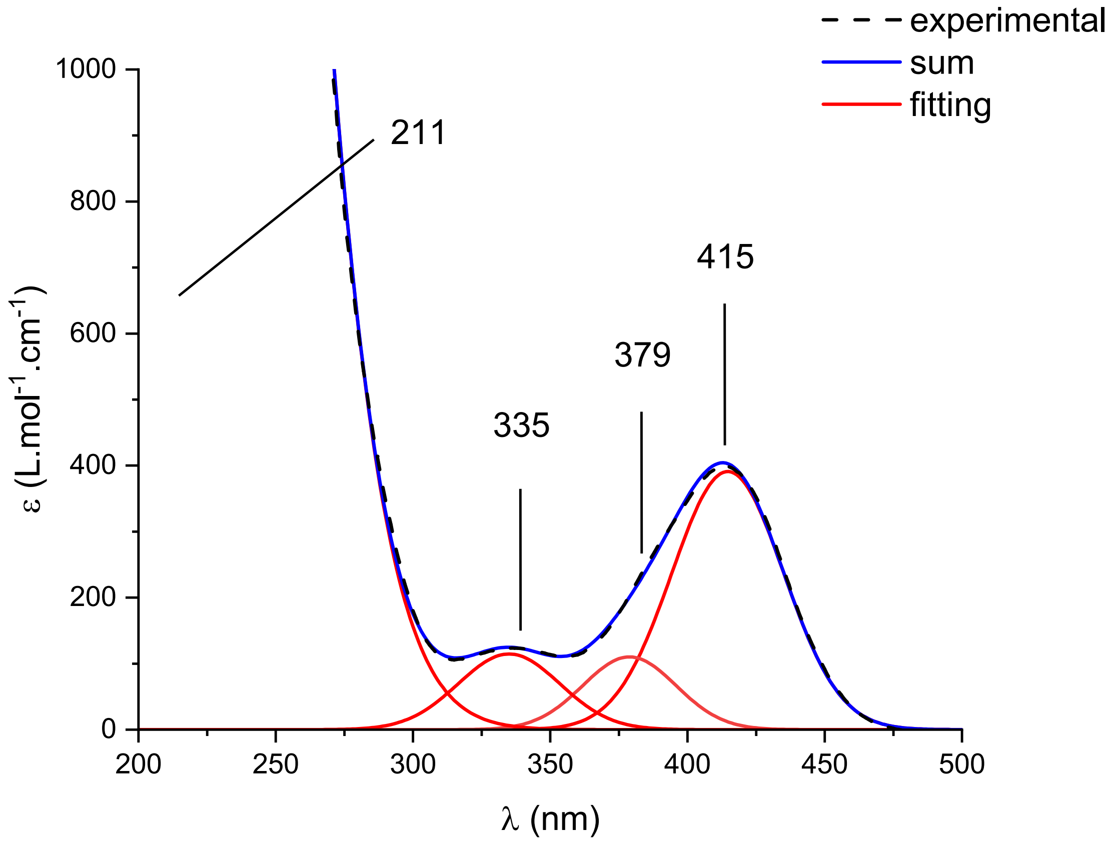

Figure S55. Deconvoluted UV-visible spectrum of **1** in pentane

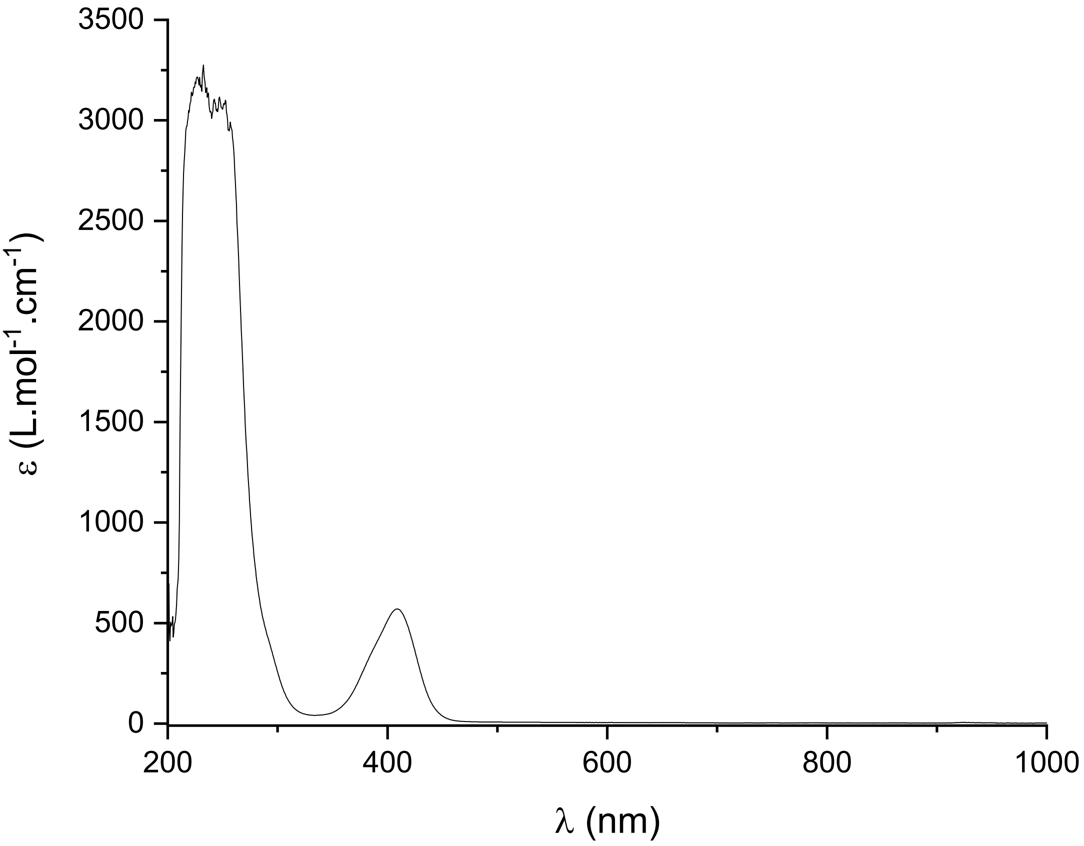

Figure S56. UV-visible spectrum of **1** in THF at 298 K

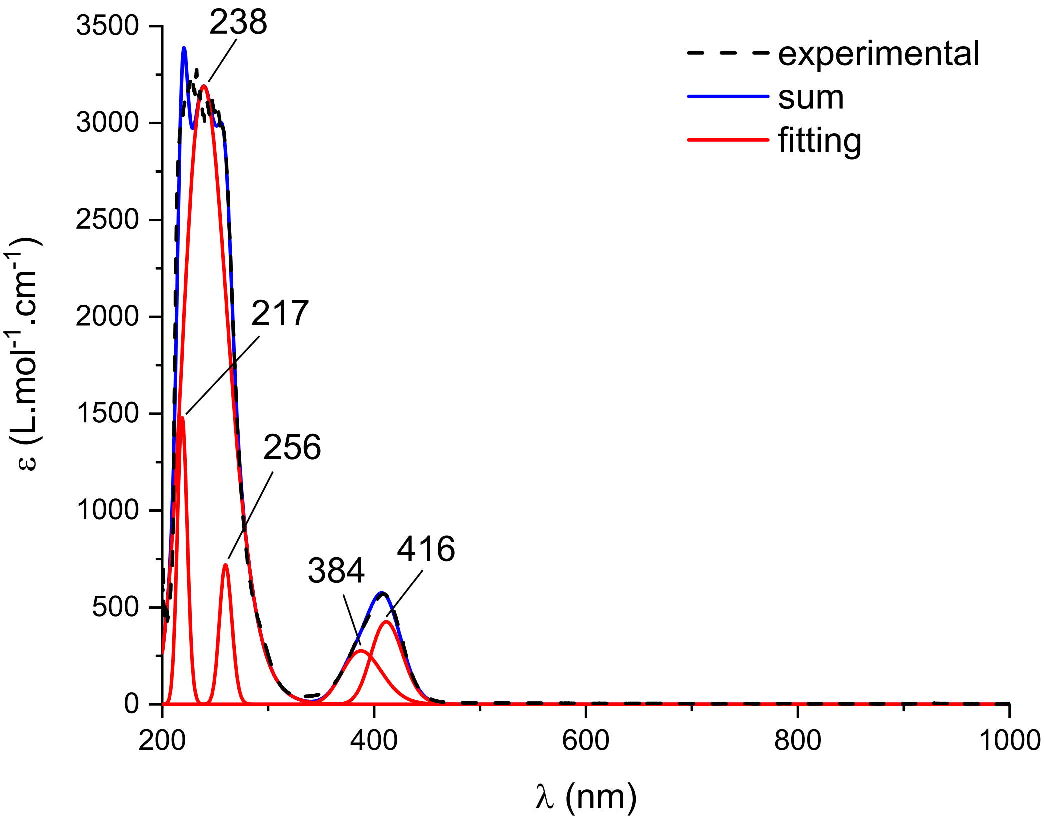

Figure S57. Deconvoluted UV-visible spectrum of **1** in THF at 298 K

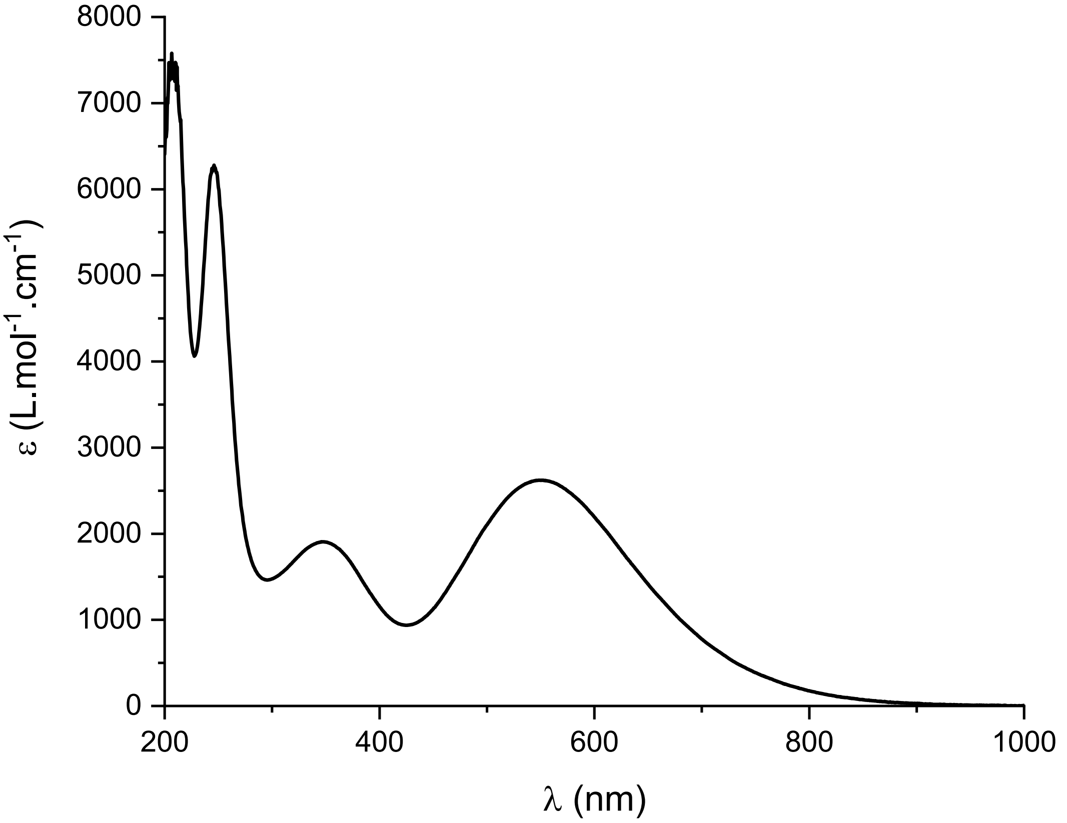

Figure S58. UV-visible spectrum of **2-Cl** in pentane at 298 K

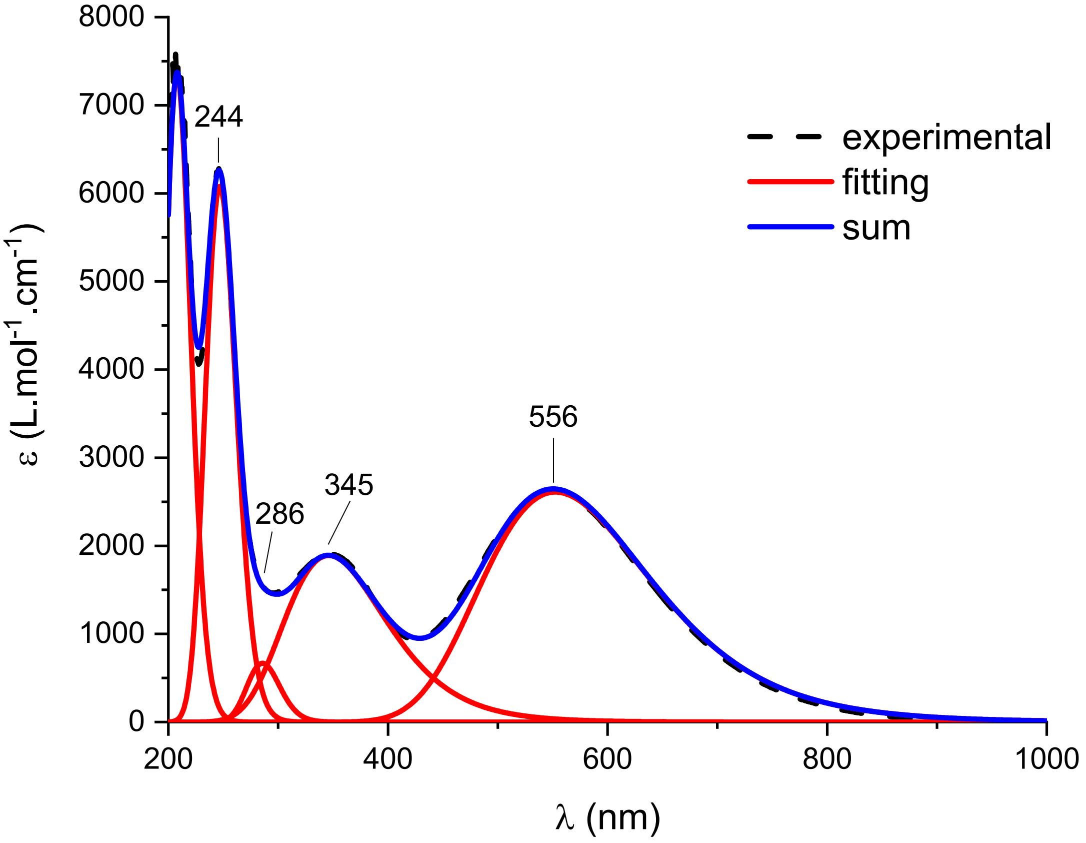

Figure S59. Deconvoluted UV-visible spectrum of **2-Cl** in pentane

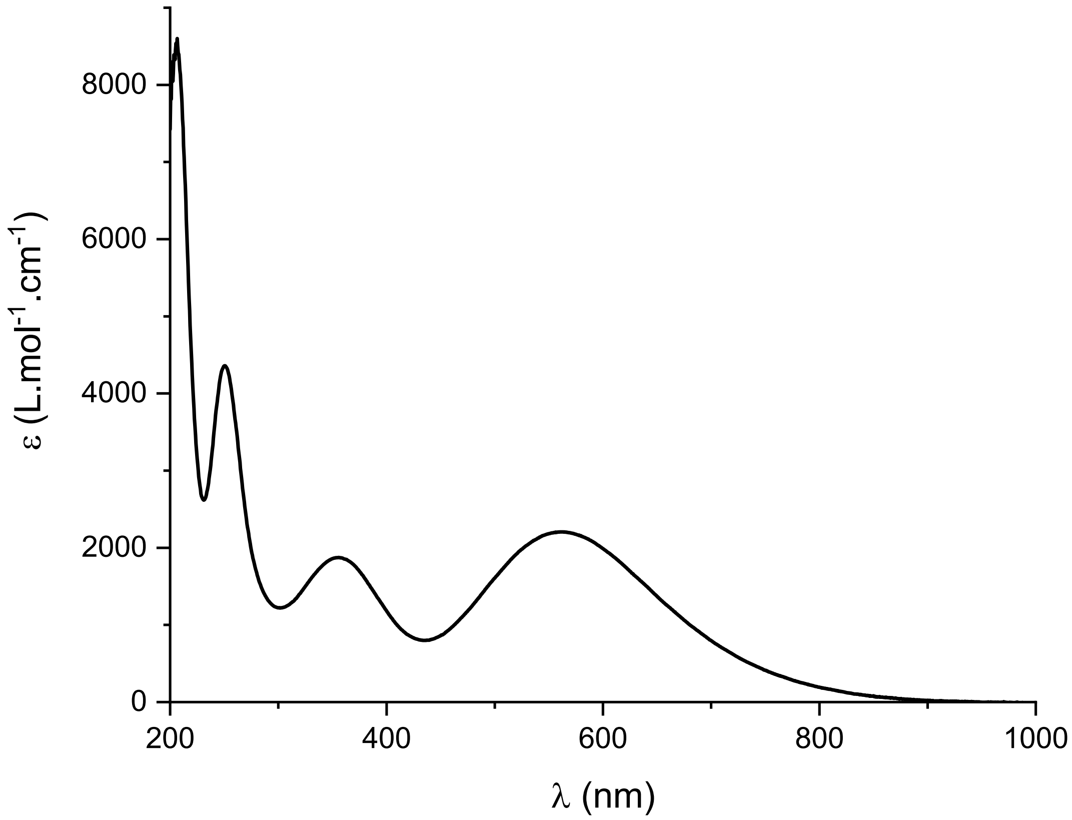

Figure S60. UV-visible spectrum of **2-Br** in pentane at 298 K

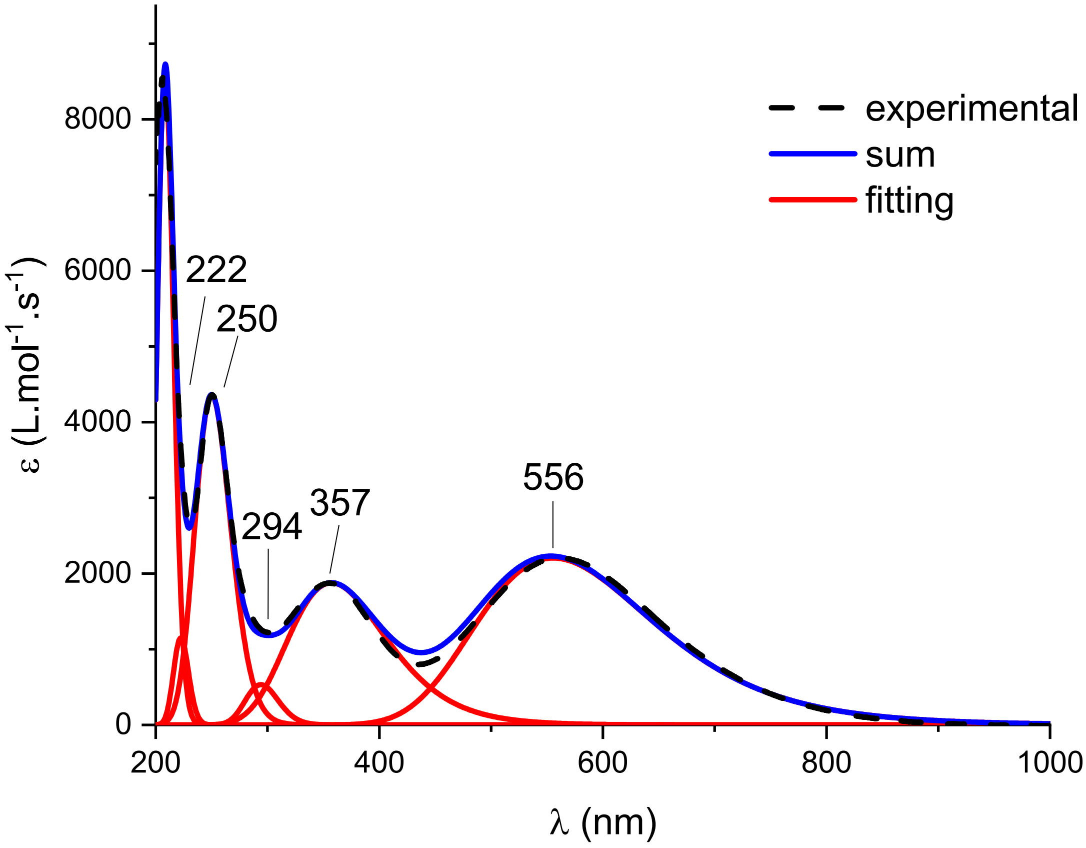

Figure S61. Deconvoluted UV-visible spectrum of **2-Br** in pentane

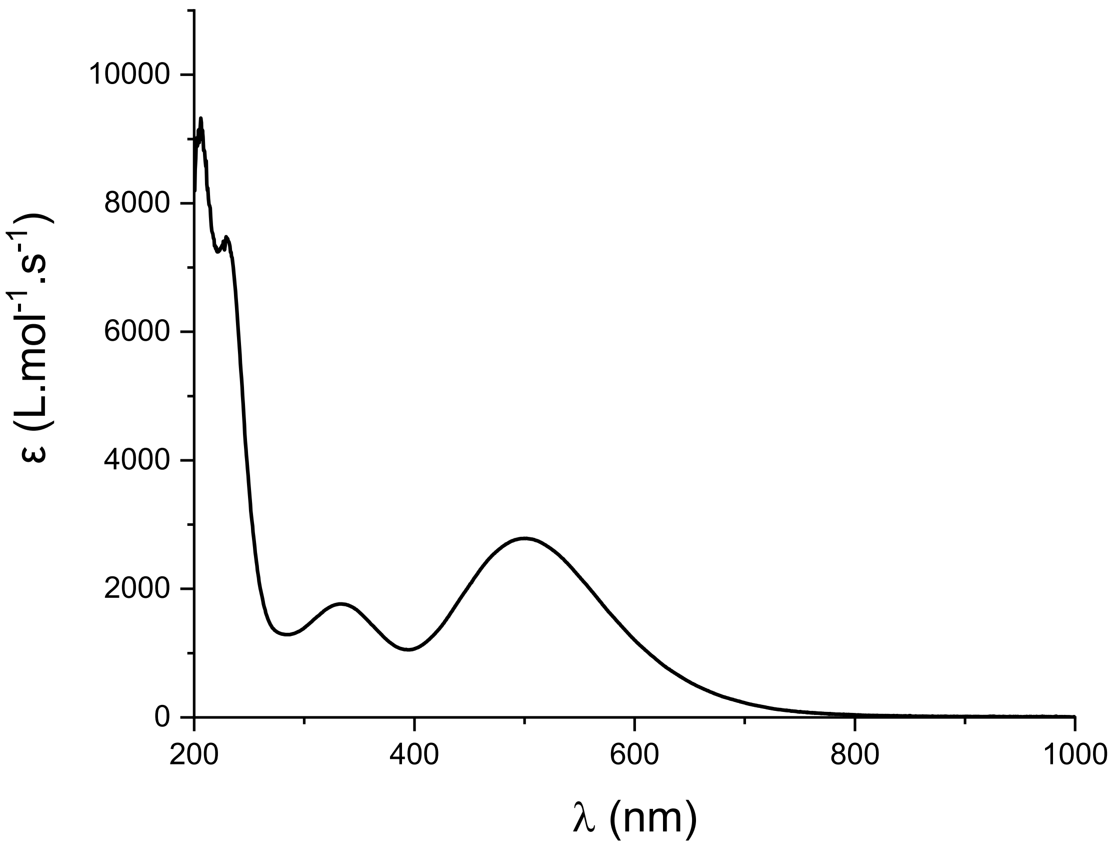

Figure S62. UV-visible spectrum of **2-F** in pentane at 298 K

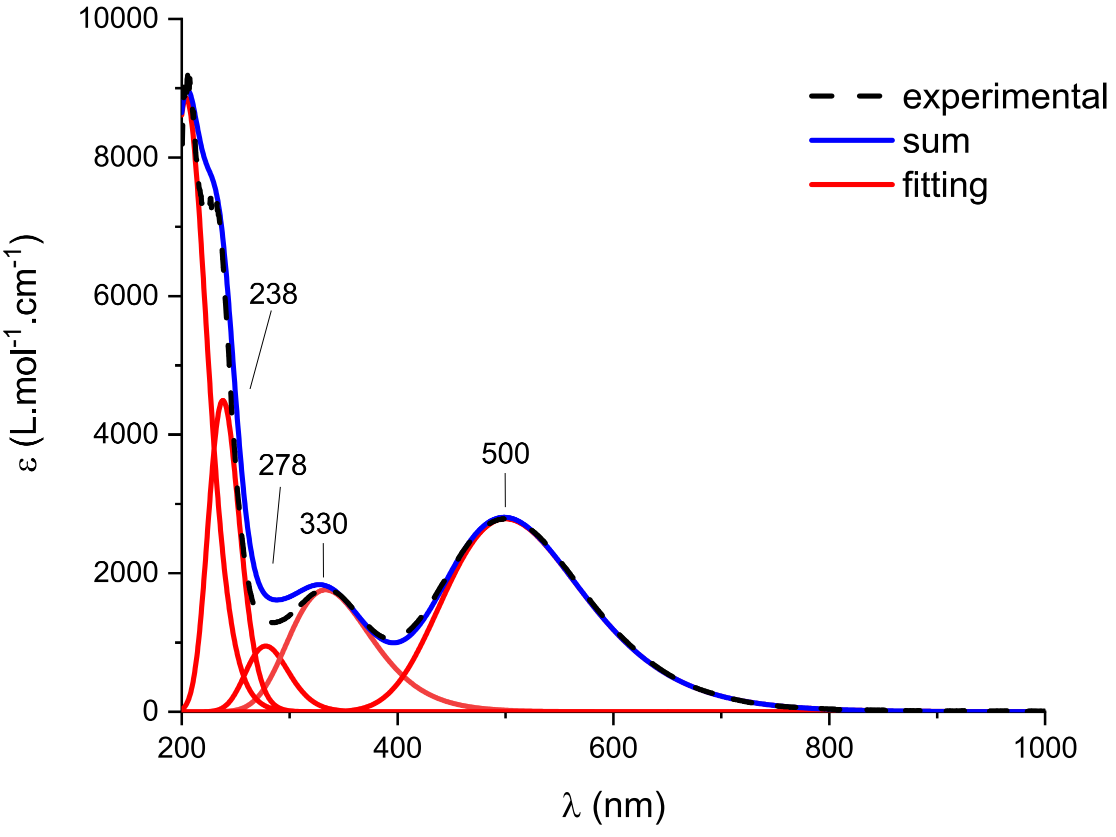

Figure S63. Deconvoluted UV-visible spectrum of **2-F** in pentane.

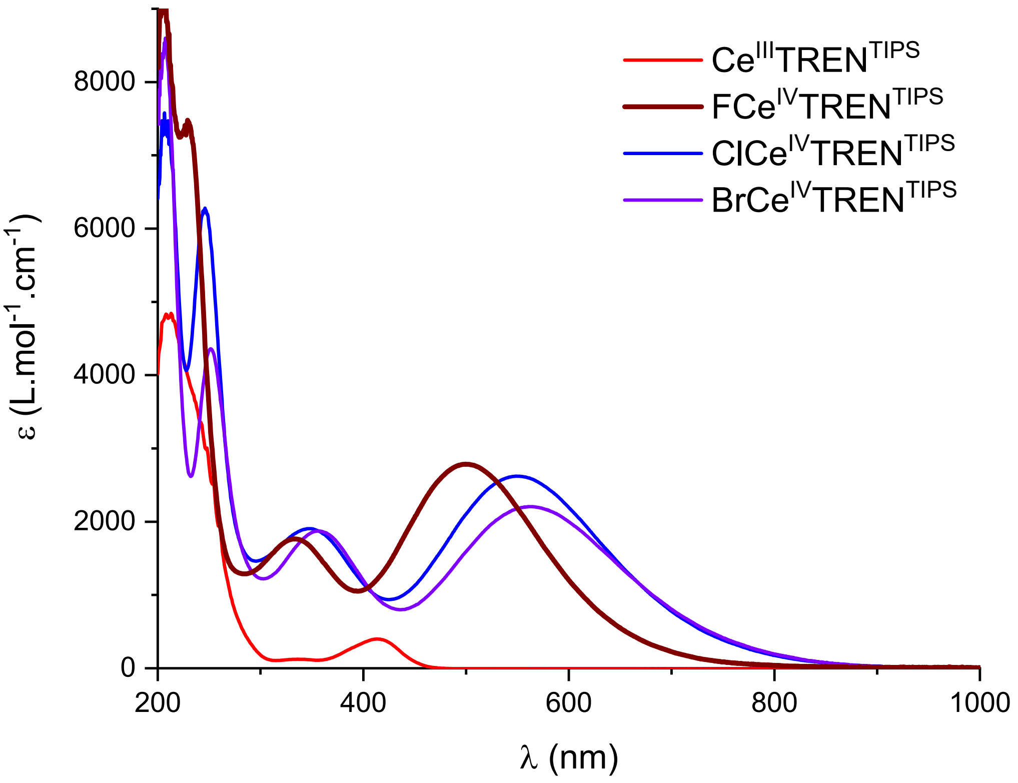

Figure S64. Superimposition of absorption spectra of **1**, **2-Cl**, **2-Br** and **2-F** in pentane at 298 K

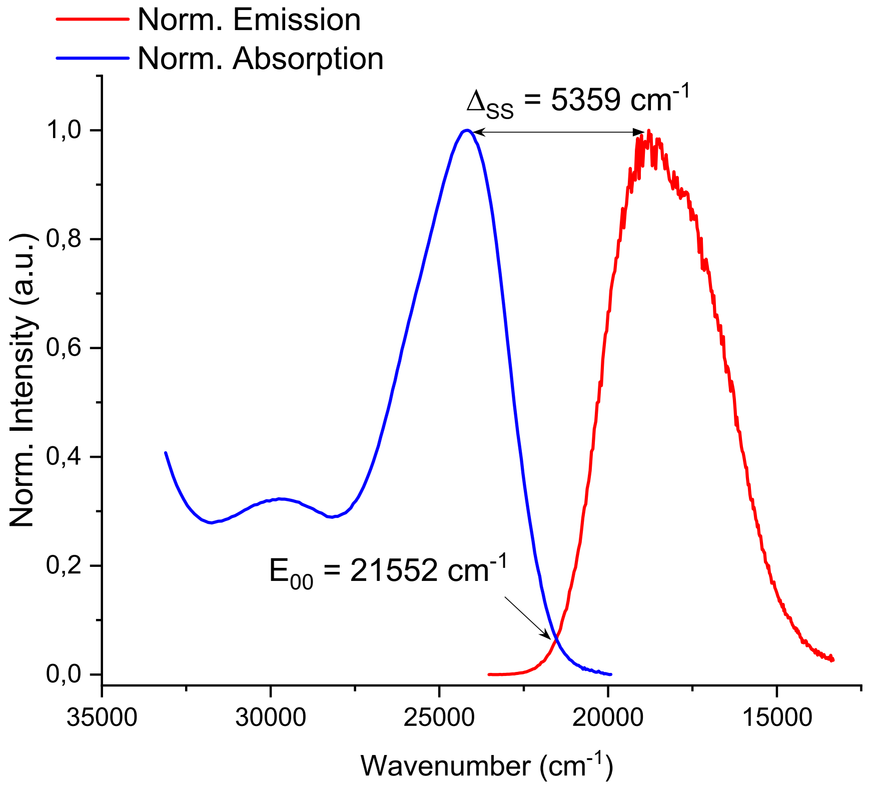

Figure S65. Normalized emission and absorption spectra of **1** in pentane, including measurements of Stokes shift and zero-zero electronic transition. Figure plot by Akos Banyasz and Olivier Maury

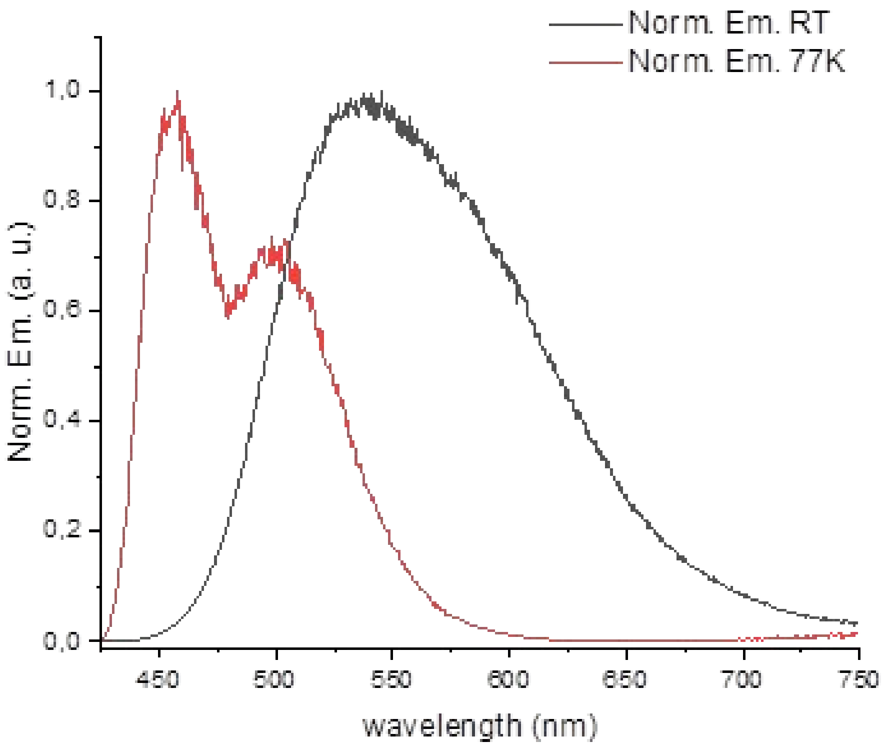

Figure S66. Emission spectra of **1** in 2-MeTHF at room temperature (Black curve) and 77 K (Red curve). Figure plotted by Akos Banyasz and Olivier Maury

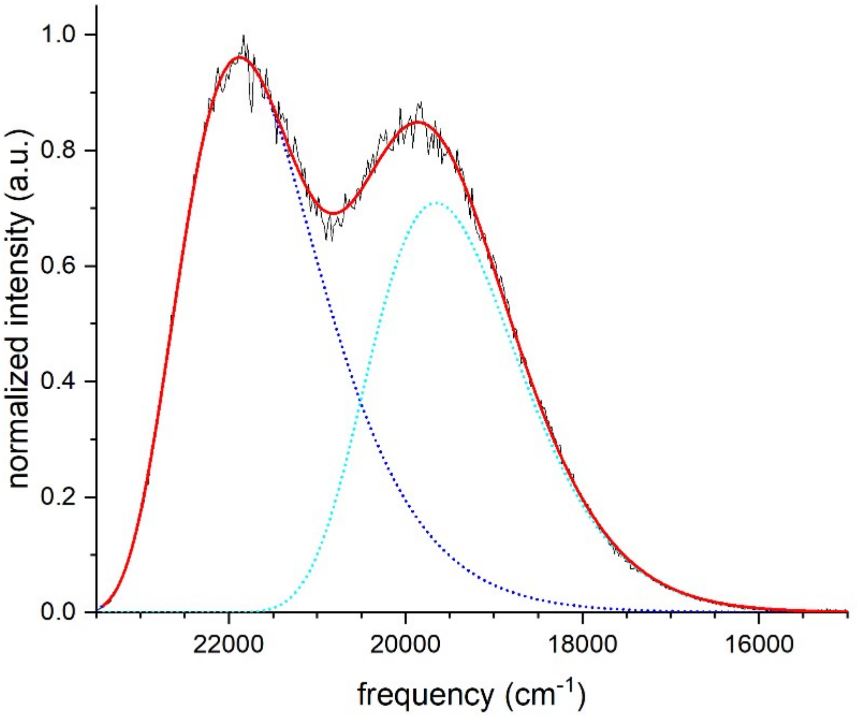

Figure S67. Emission spectra of **1** in 2-MeTHF at 77 K (black) presented on frequency scale along with the lognorm fit of the two-constituting bands (1 in dark and 2 in light blue) and the sum (red) of the two-lognorm functions. Figure realised by Akos Banyasz and Olivier Maury

The 77 K emission spectrum of [Ce^{III}TREN^{TIPS}], **1**, was converted to frequency scale then λ^2 correction was applied on the emission intensity. The obtained spectrum was fitted by the sum of two simplified lognorm functions[3] to characterize the parameters of the two involved transitions. The frequencies corresponding to the peak maximum (ν_{max}) and to the full-width at half-maximum (ν_{FWHM}) are calculated according to equations 2 and 3 and presented in **Table S11**.

Equation 1. $I_\nu = A * \exp\left(-\beta^2\left(ln\left(\frac{a-\nu}{b}\right)\right)^2\right)$

Equation 2. $\upsilon_{max} = a - b$

Equation 3. $\upsilon_{FWHM} = b\ sinh(\sqrt{ln2}/\beta)$

	A	B
A	0.96 (0.01)	0.71 (0.01)
β^2	5.4 (0.3)	10.0 (0.3)
a (10^3 cm^{-1})	24.5 (0.1)	23.4 (0.1)
b (10^3 cm^{-1})	2.6 (0.1)	3.8 (0.1)
ν_{max} (10^3 cm^{-1})	21.9 (0.1)	19.7 (0.1)
ν_{FWHM} (10^3 cm^{-1})	1.9 (0.1)	2.0 (0.1)

Table S11. Result of the lognorm fit of the 77 K emission spectrum of **1** according to equations 1-3. Standard errors are in parenthesis. Data provided by Akos Banyasz and Olivier Maury

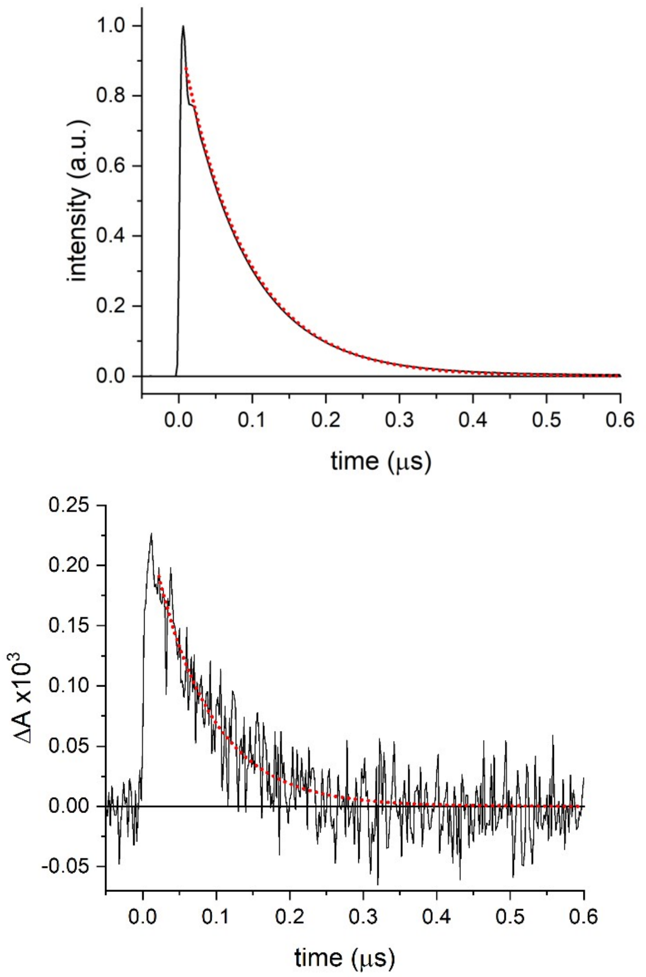

Figure S68. Luminescence (left) and transient absorption (right) decays of **1** obtained at 550 and 400 nm, respectively, upon 354 nm excitation. The dotted red line represents the monoexponential fit. Figure realised by Akos Banyasz and Olivier Maury

a) b)

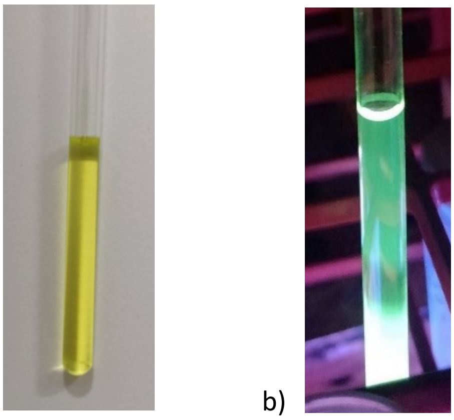

Figure S69. a) **1** under ambient light, b) **1** under irradiation at 427 nm at 298 K

VII. Electrochemistry

a) Cyclic voltammetry measurements

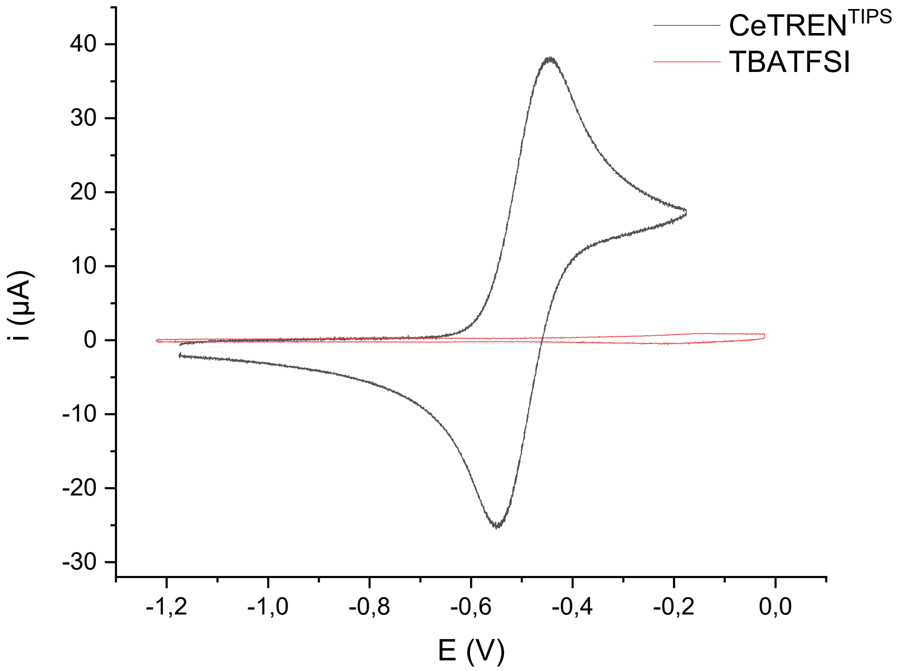

Figure S70. Cyclic voltammogram at 50 mV.s^{-1} scan rate of supporting electrolyte (100 mM) and **1** (4 mM).

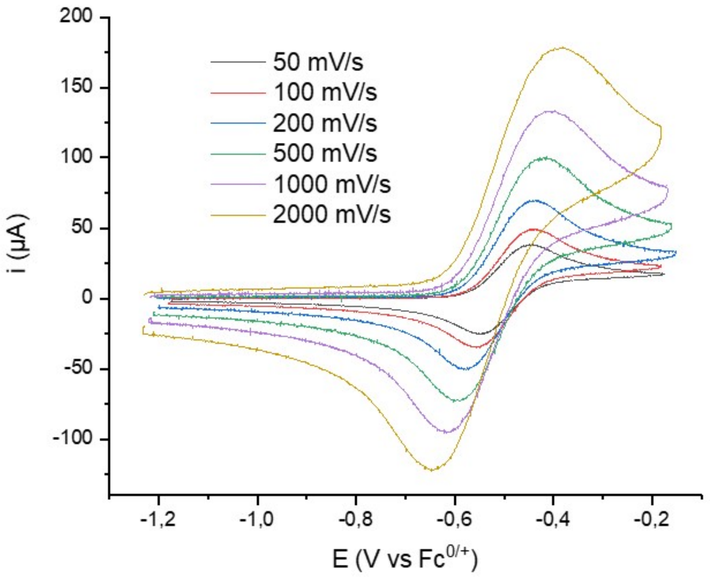

Figure S71. Cyclic voltammograms at various scan rates of 1 (4 mM)

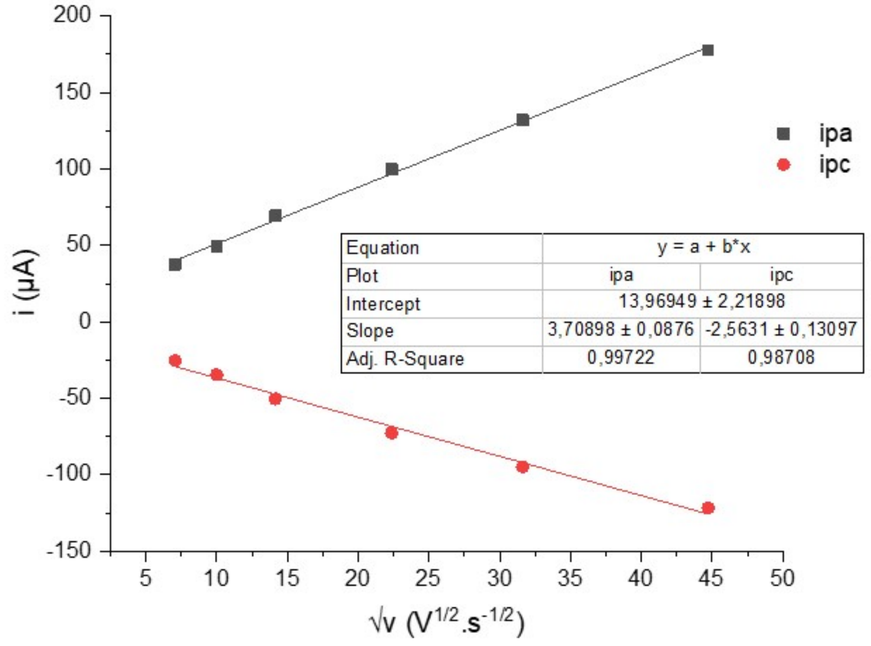

Figure S72. Modelling of the relationship between i_p and square root of scan rate to check the validity of Randles-Ševčík equation

v (mV.s^{-1})	$\sqrt{v}$ (mV$^{1/2}$.s$^{-1/2}$)	i_{pa} (µA)	i_{pc} (µA)	E_{pa} (V)	E_{pc} (V)	ΔV (V)
**50**	7.07	37.5	-25.3	-0.45	-0.56	0.11
**100**	10	49.3	-34.6	-0.44	-0.51	0.12
**200**	14.14	69.4	-50.4	-0.44	-0.58	0.14
**500**	22.36	99.7	-72.7	-0.42	-0.60	0.18
**1000**	31.62	132	-95	-0.41	-0.62	0.21
**2000**	44.72	177.8	-121.8	-0.39	-0.64	0.25

Table S12. Experimental data of the influence of the scan rate over the intensities and the potentials of anodic and cathodic peaks in cyclic voltammetry of **1**

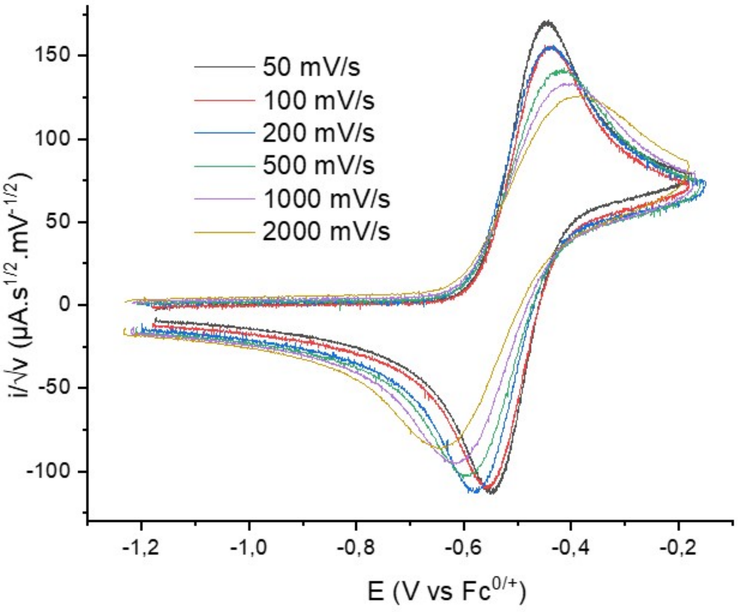

Figure S73. Cyclic voltammograms of **1** at different scan rates, normalized by $\sqrt{v}$

b) Estimation of the work function ω_f

As detailed in Appendix, $\omega_f = \frac{e^2}{\varepsilon a}$ where e is the elementary charge of the electron (e = 1.6 10^{-19} C), ε the dielectric permittivity of the medium and a the ion-radical pair distance.

Considering $\varepsilon = \varepsilon_r \varepsilon_0$ with ε_r being the relative permittivity of the solvent (here THF, $\varepsilon_r = 7.58$)[4] and $\varepsilon_0 = 8.85.10^{-12}\ N.m^2.C^{-2}$, and a being the distance separating the ion-radical pair, it can be assumed that $a \approx 15$ Å that is the size of $Ce^{III}TREN^{TIPS}$ **1**. Thus:

$\omega_f < 0.09\ V$

VIII. Reactivity

a) Photochemical degradation of [ClCe^{IV}TREN^{TIPS}] (2-Cl)

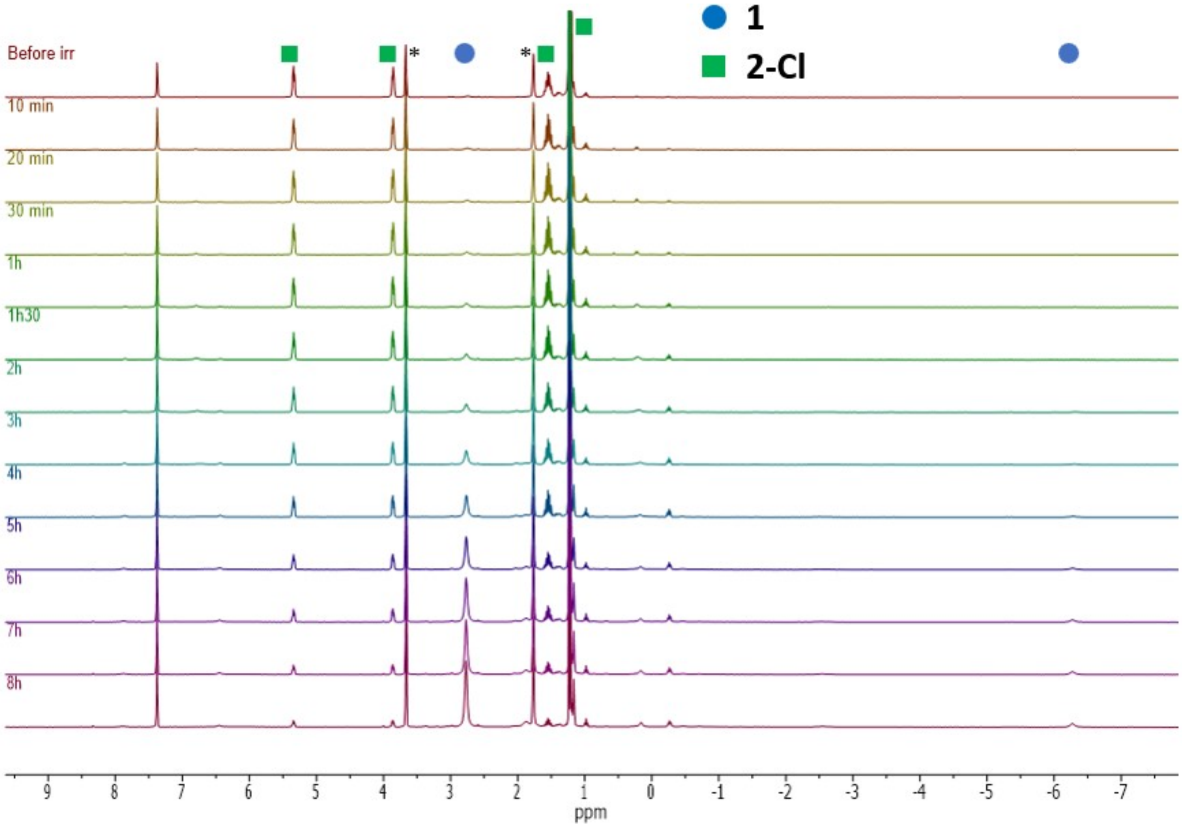

Figure S74. ^{1}H NMR spectra of the photochemical degradation of **2-Cl** (5 mg, 6.3 µmol) in THF-d_8 (0.4 mL). Solvent residual peak is indicated with *

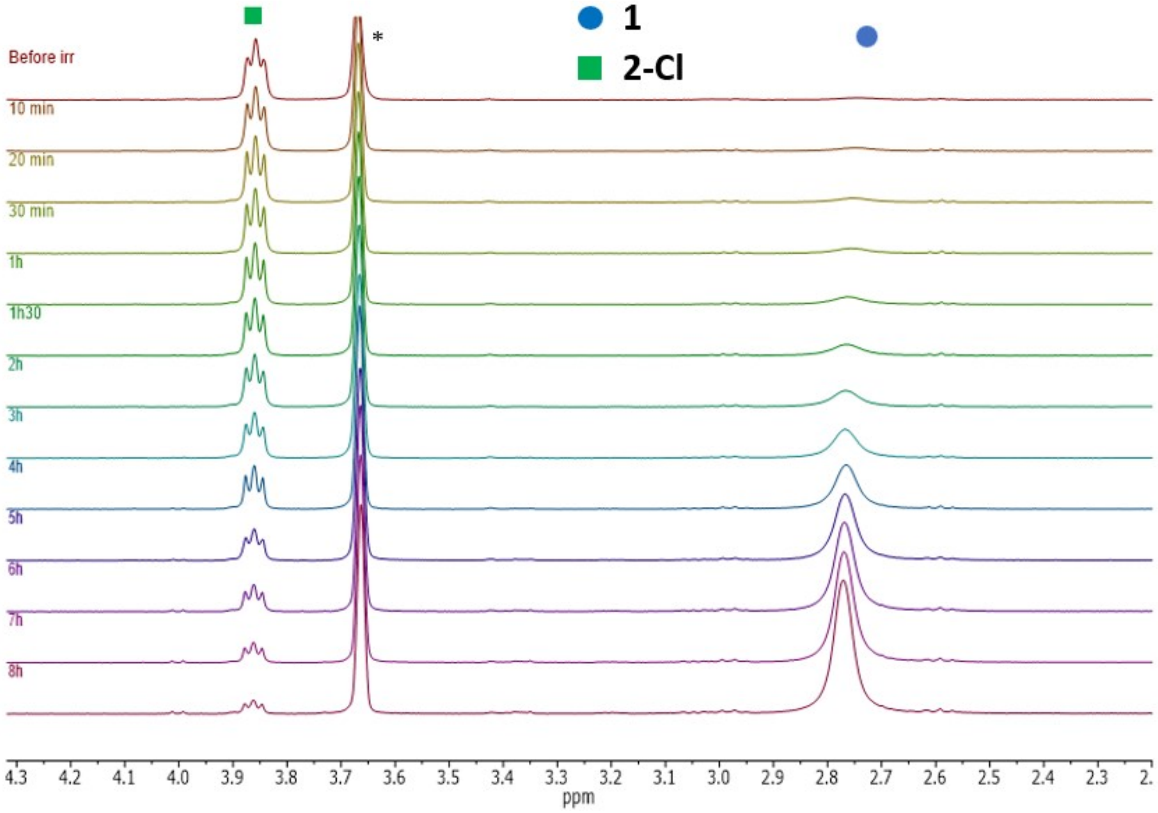

Figure S75. ^{1}H NMR spectra of the photochemical degradation of **2-Cl** (5 mg, 6.3 µmol) in THF-d_8 (0.4 mL). Zoom between 2.2 and 4.3 ppm. Solvent residual peak is indicated with *

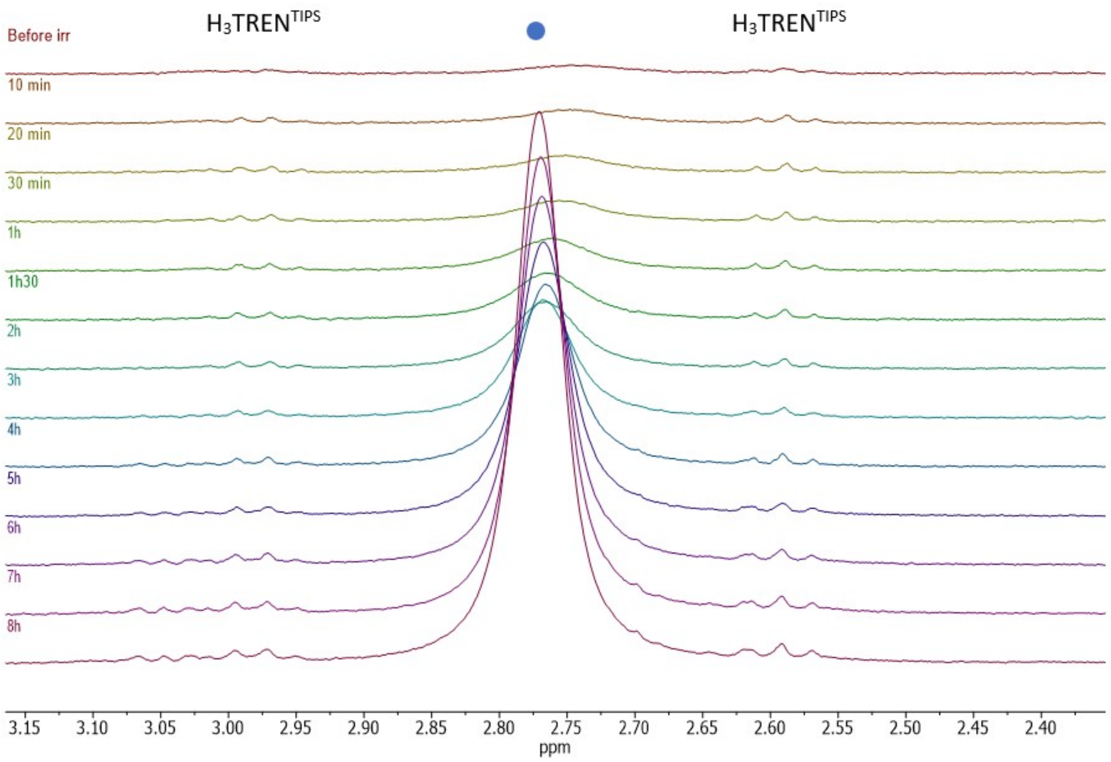

Figure S76. Zoom on the 2.3 and 3.2 ppm range.

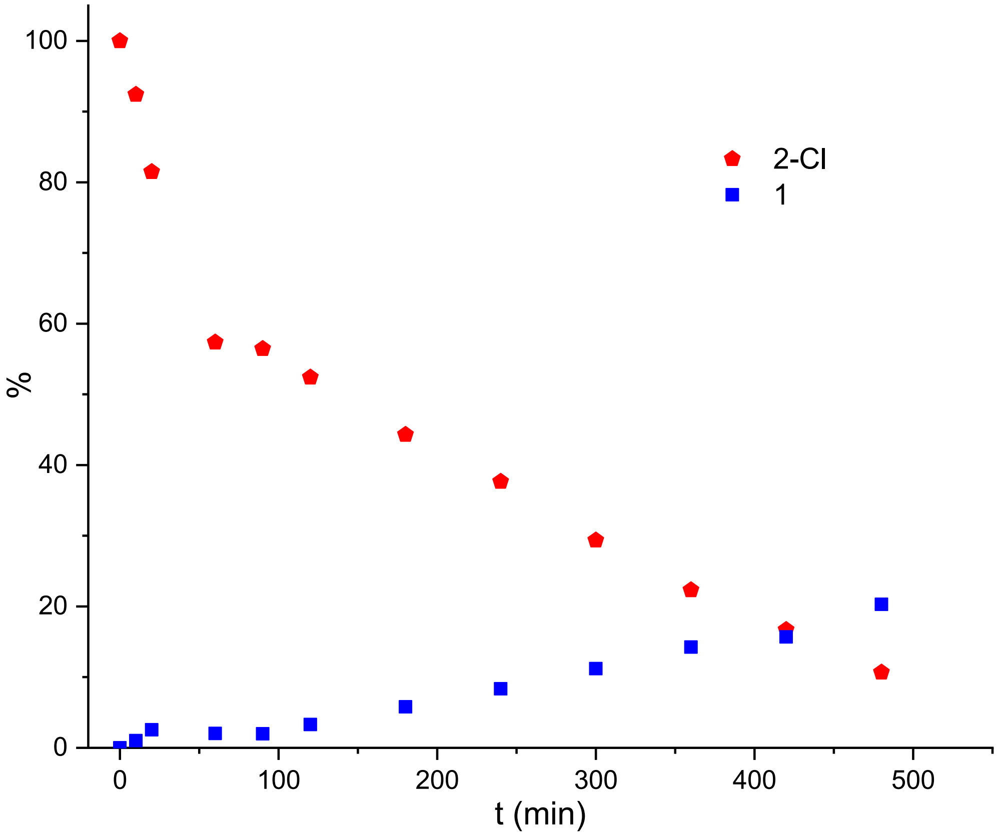

Figure S77. Photochemical degradation of **2-Cl** (5 mg, 6.3 µmol) at 427 nm in THF-d_8 (0.4 mL). NB : between 20 and 60 min, and 240 and 300 min of irradiation, the NMR tube was stored at 0 °C overnight. The point at 60 min seems to be an outlier, which could be due to the insufficient warming up to room temperature before measurement

t (min)	% **2-Cl**	% H_3TREN^{TIPS}	% **1**
0	100	2.2	0
10	92.42	2.43	1.02
20	81.47	1.89	2.55
60	57.37	1.9	2.02
90	56.45	1.87	1.99
120	52.42	1.7	3.29
180	44.3	1.47	5.8
240	37.68	1.69	8.35
300	29.36	1.93	11.2
360	22.33	2.33	14.26
420	16.71	2.47	15.69
480	10.68	2.31	20.3

Table S13. Experimental data of the photochemical degradation of **2-Cl** (5 mg, 6.3 µmol) in THF-d_8 (0.4 mL)

b) Radical clock experiment

Compound **1** (10 mg, 13 µmol, 1 eq.), 1-allyloxy-2-chlorobenzene (2 µL, 13 µmol, 1 eq.), and dihydroanthracene (5 mg, 26 µmol, 2 eq.) were dissolved in THF-d_8 in a J. Young NMR tube, and irradiated for 12 h at 427 nm. After irradiation, the reaction medium was colourless.

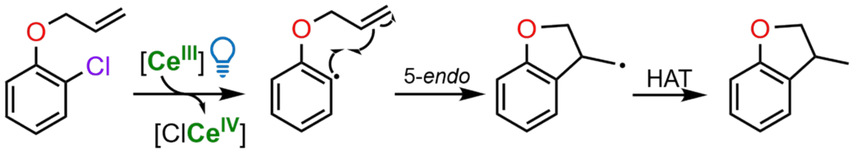

Figure S78. Mechanism of the radical clock experiment using 1-allyloxy-2-chlorobenzene.

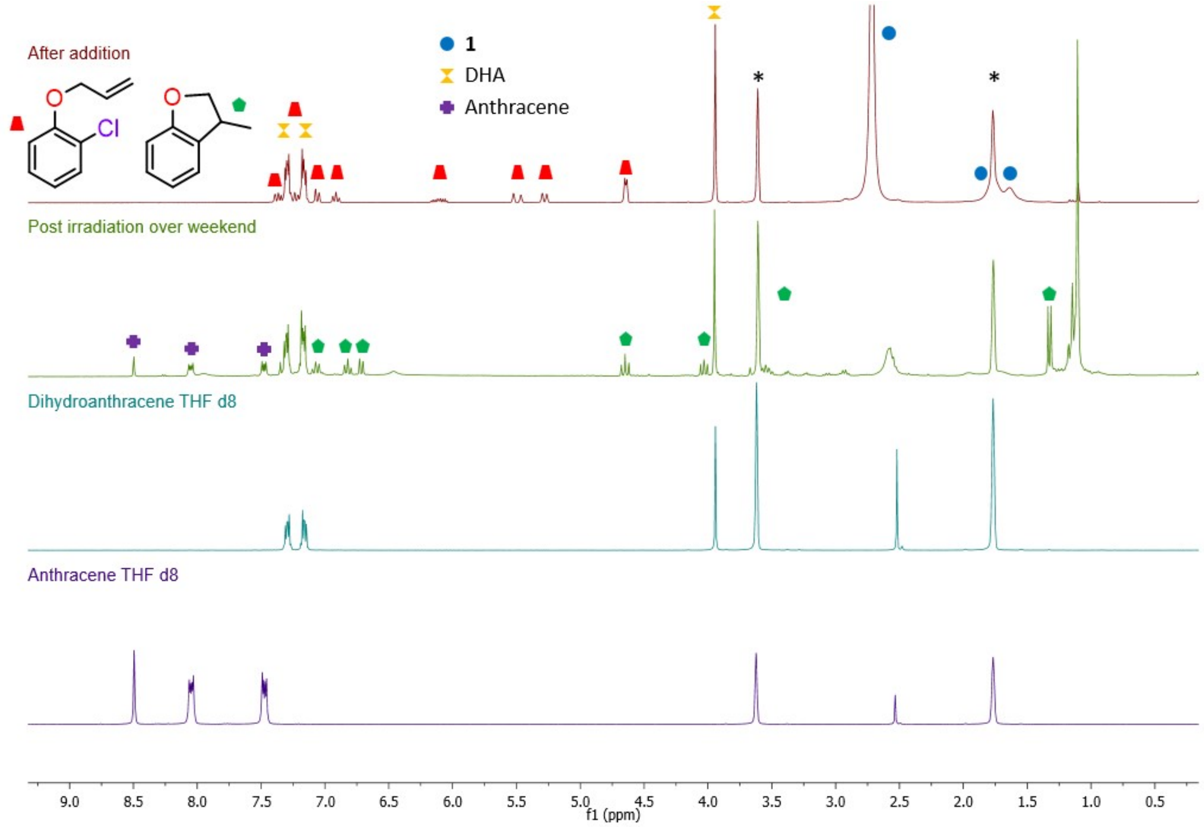

Figure S79. Stacking of spectra before irradiation (red), after irradiation (green) of radical clock experiment, with dihydroanthracene (light blue) and anthracene (purple) in THF d_8. Solvent residual peaks are indicated with *

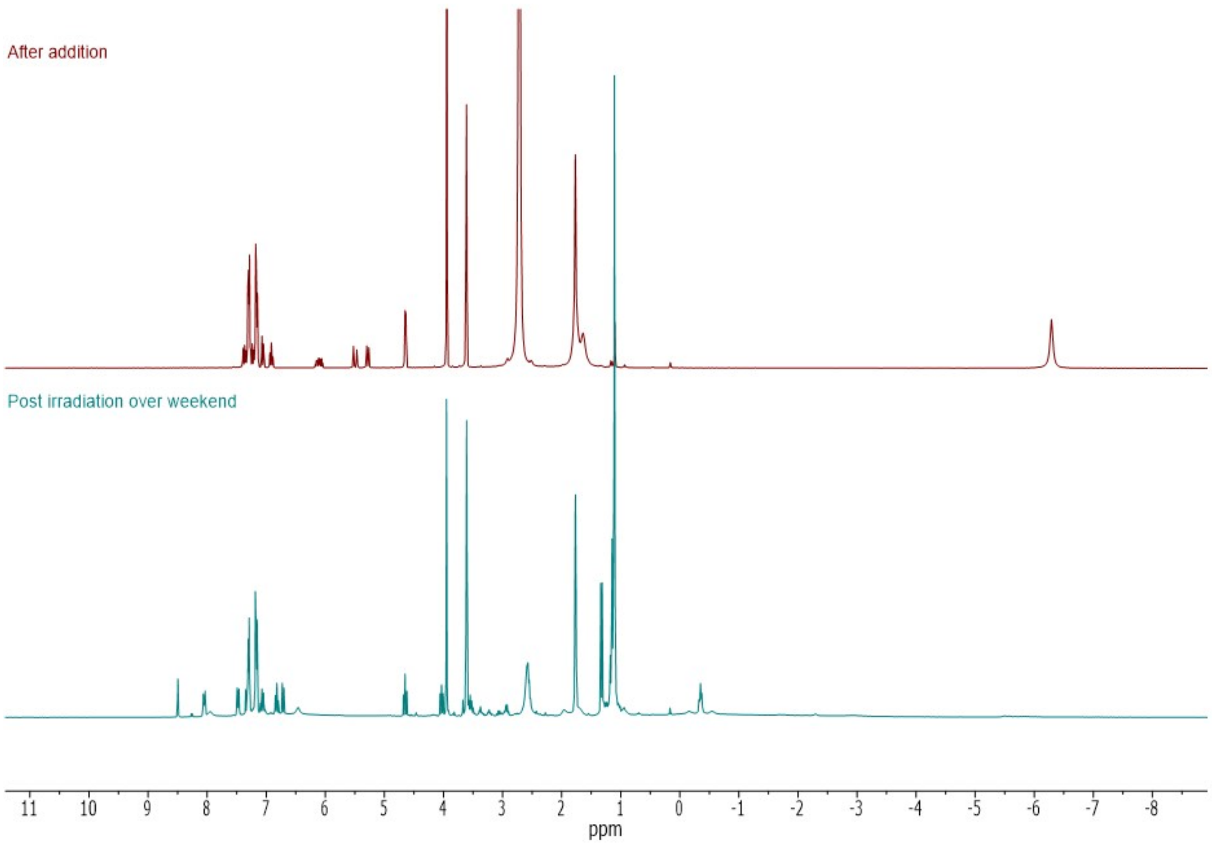

Figure S80. ^{1}H NMR spectra of the reaction medium for radical clock experiment before and after over weekend irradiation at 427 nm.

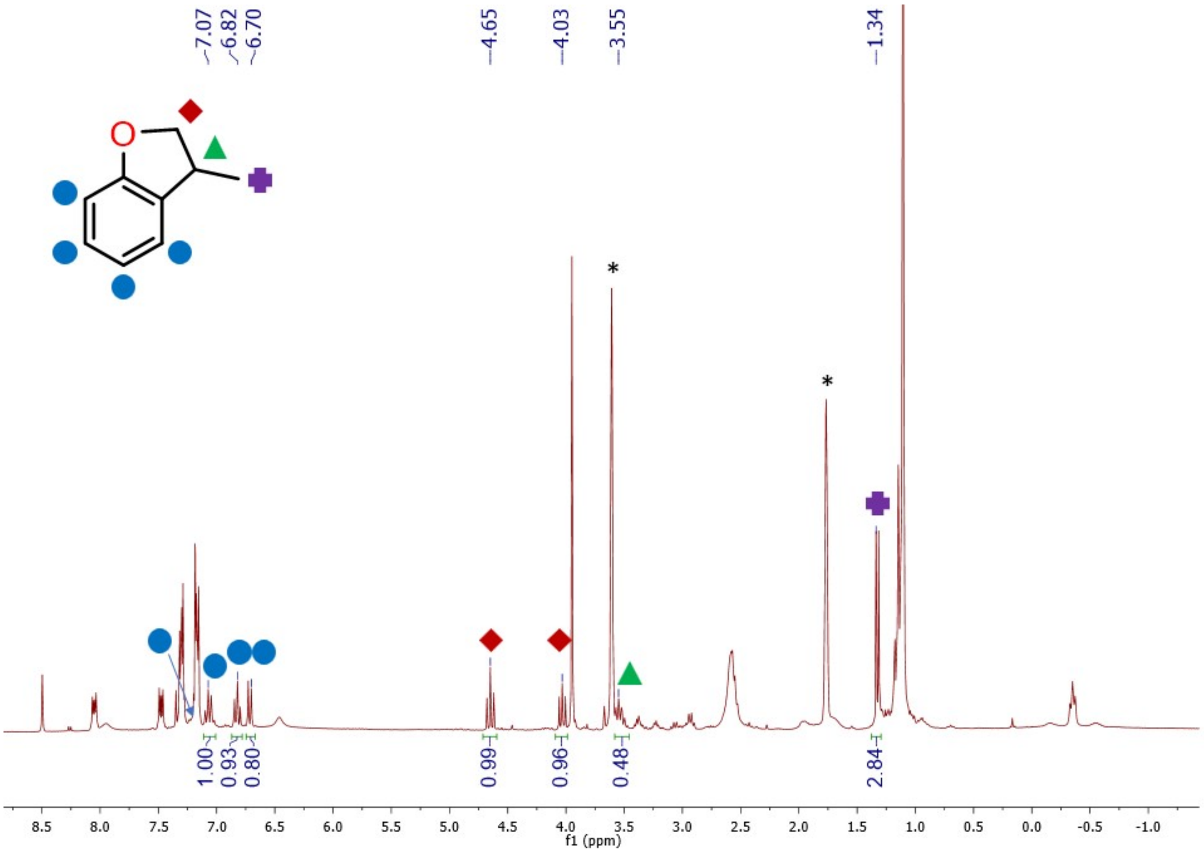

Figure S81. ^{1}H NMR of the reaction medium of the radical clock experiment in THF d_8. The presence of 3-methyl-2,3-dihydrobenzofuran in the spectra correspond to the one reported in the literature.[1]

c) Reactivity with CH_2Cl_2

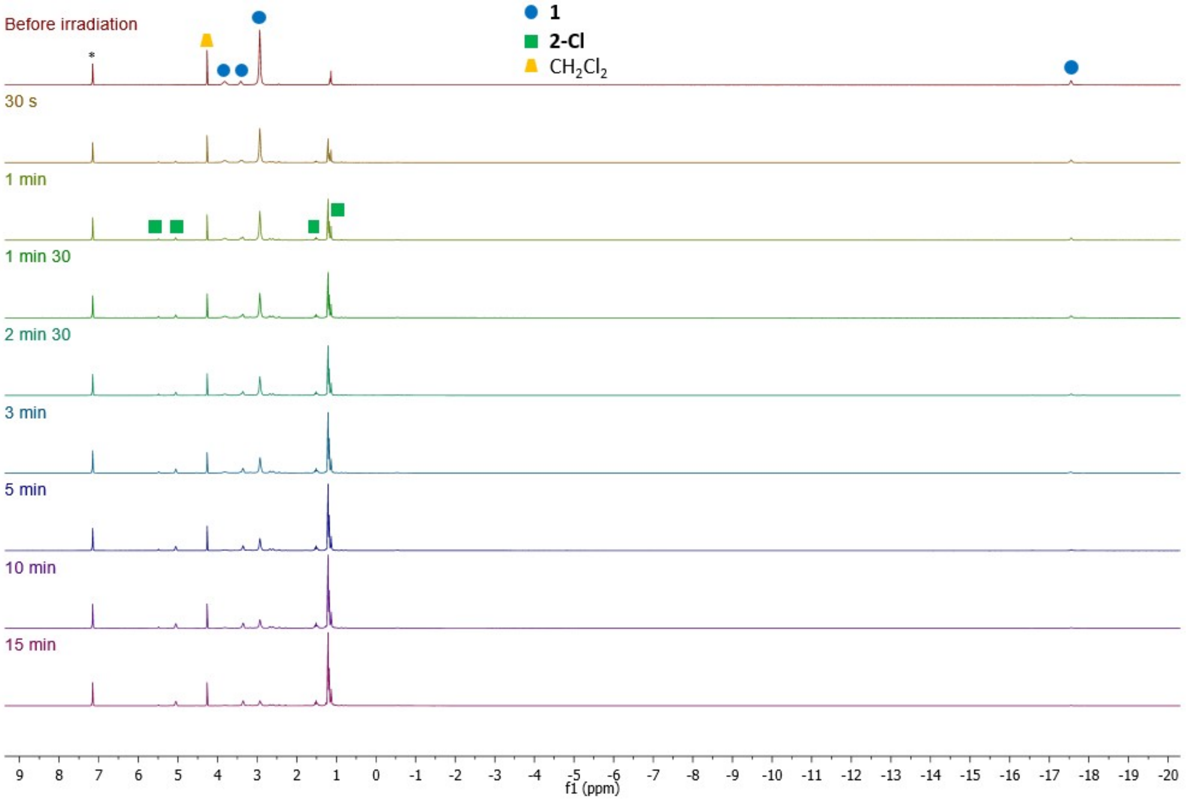

Figure S82. Kinetic study of the photochemical reaction between **1** (12.7 mg, 17 µmol, 1 eq.) and CH_2Cl_2 (2 µL, 13 µmol, 1 eq.) under irradiation at 427 nm in C_6D_6 at 298 K

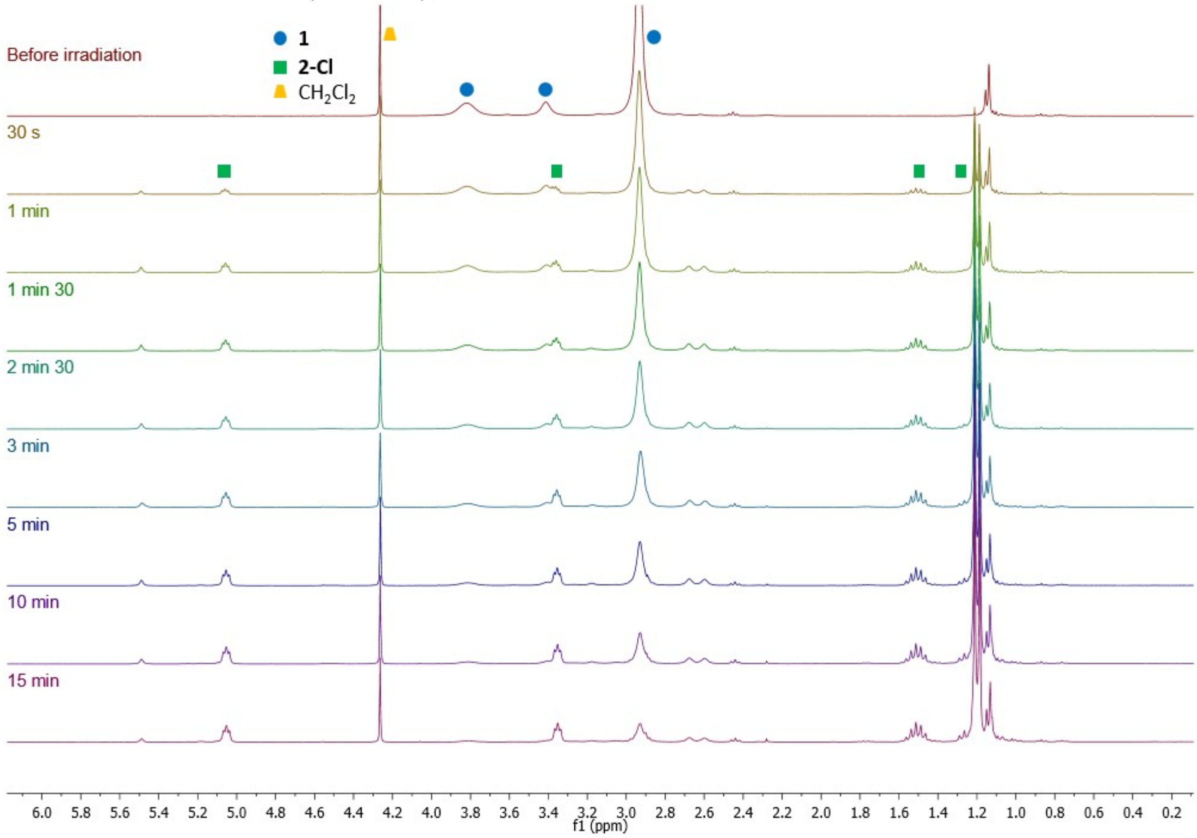

Figure S83. Kinetic study of the photochemical reaction between **1** (12.7 mg, 17 µmol, 1 eq.) and CH_2Cl_2 (2 µL, 13 µmol, 1 eq.) under irradiation at 427 nm in C_6D_6 at 298 K. Zoom on the region between 6.0 ppm and 0.2 ppm. Solvent residual peak is indicated by *

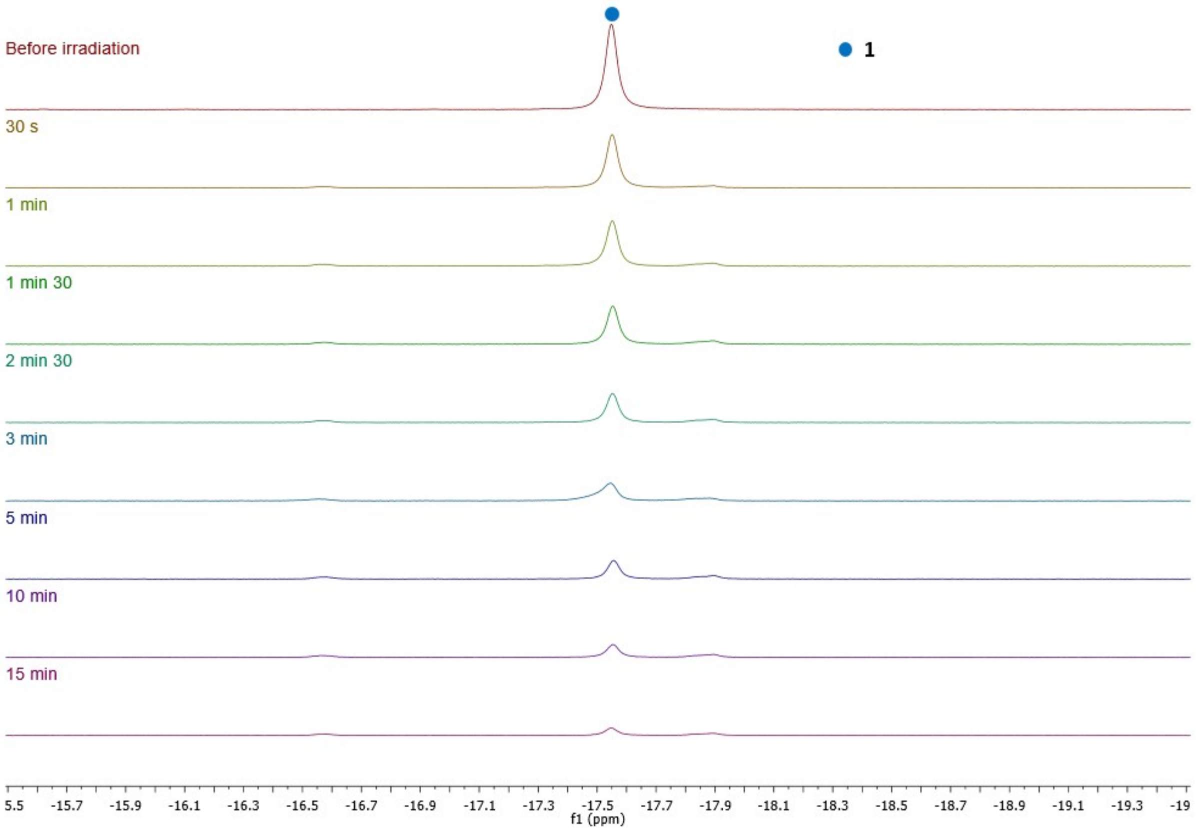

Figure S84. Kinetic study of the photochemical reaction between **1** (12.7 mg, 17 µmol, 1 eq.) and CH_2Cl_2 (2 µL, 13 µmol, 1 eq.) under irradiation at 427 nm in C_6D_6 at 298 K. Zoom on the -15.5 ppm and -19.4 ppm range. Solvent residual peak is indicated by *

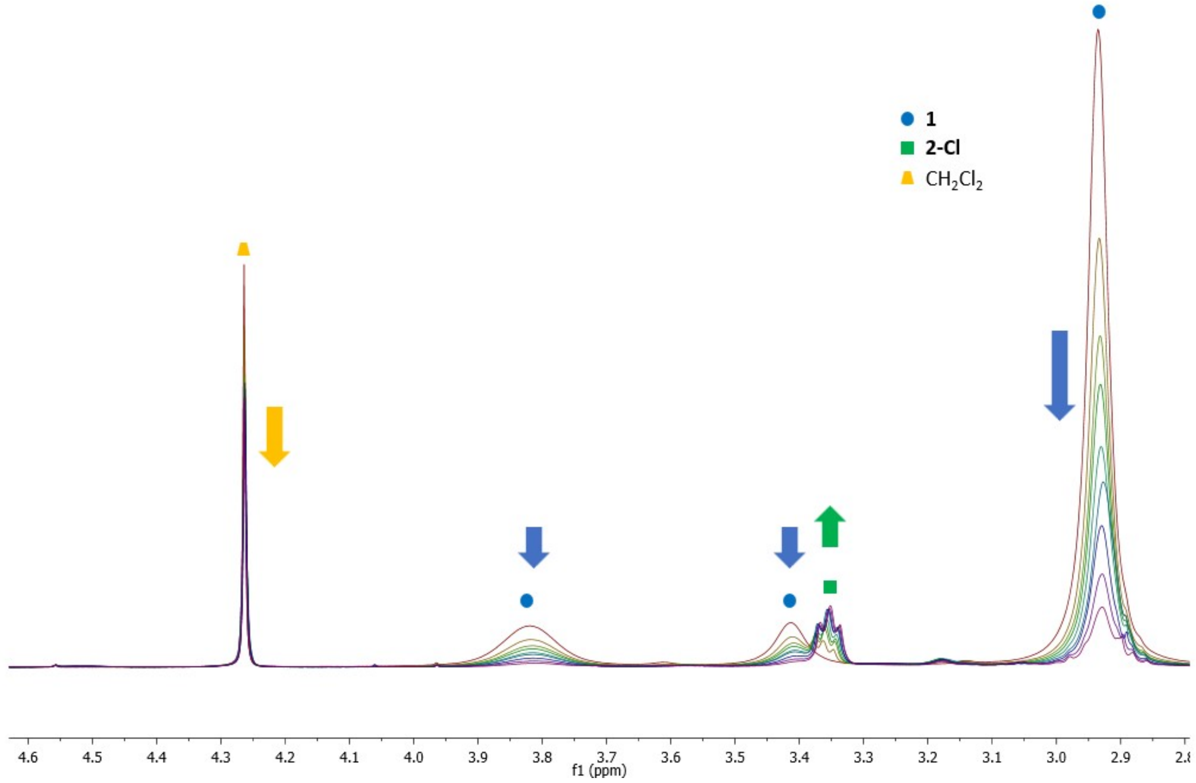

Figure S85. Kinetic study of the photochemical reaction between **1** (12.7 mg, 17 µmol, 1 eq.) and CH_2Cl_2 (2 µL, 13 µmol, 1 eq.) under irradiation at 427 nm for the first 10 minutes at 298 K. Zoom on the region between 4.4 ppm and 2.8 ppm.

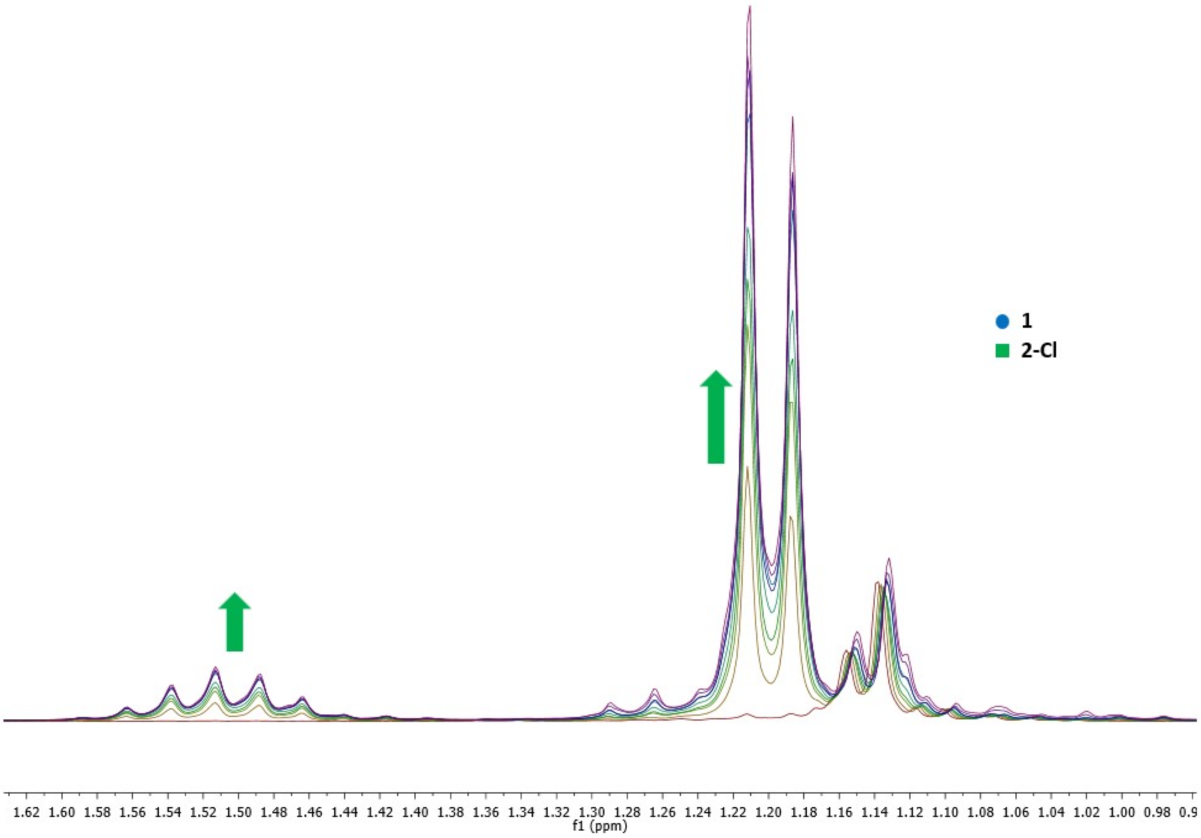

Figure S86. Kinetic study of the photochemical reaction between **1** (12.7 mg, 17 µmol, 1 eq.) and CH_2Cl_2 (2 µL, 13 µmol, 1 eq.) under irradiation at 427 nm for the first 10 minutes at 298 K. Zoom on the region between 1.65 ppm and 0.9 ppm.

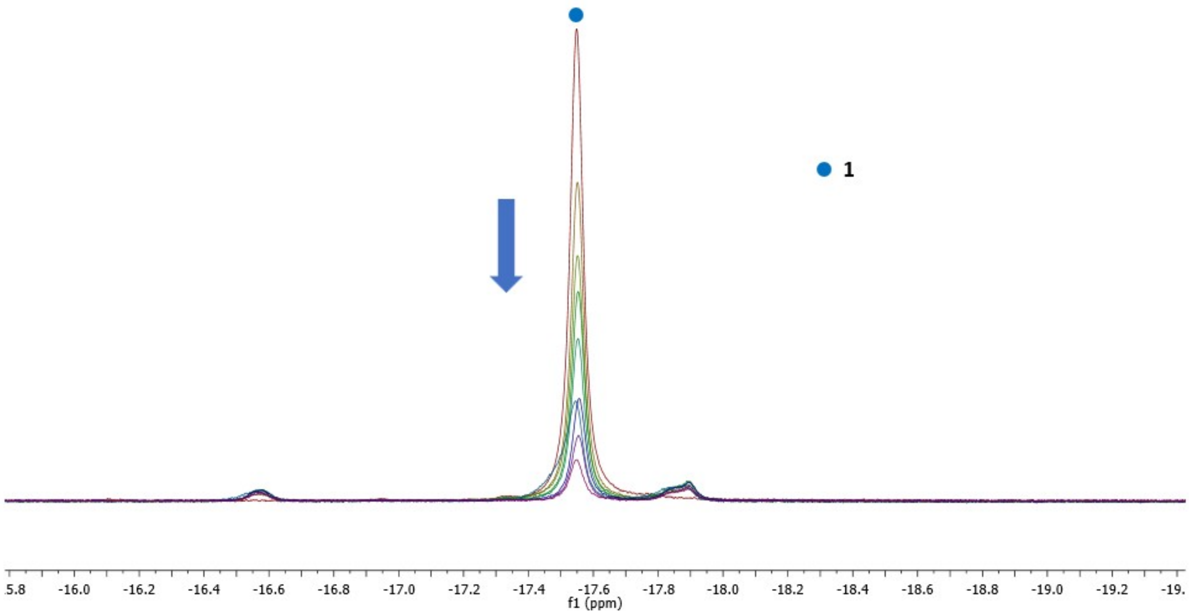

Figure S87. Kinetic study of the photochemical reaction between **1** (12.7 mg, 17 µmol, 1 eq.) and CH_2Cl_2 (2 µL, 13 µmol, 1 eq.) under irradiation at 427 nm for the first 10 minutes at 298 K. Zoom on the region between -15.8 ppm and -19.3 ppm.

d) Reactivity with chlorinated substrates

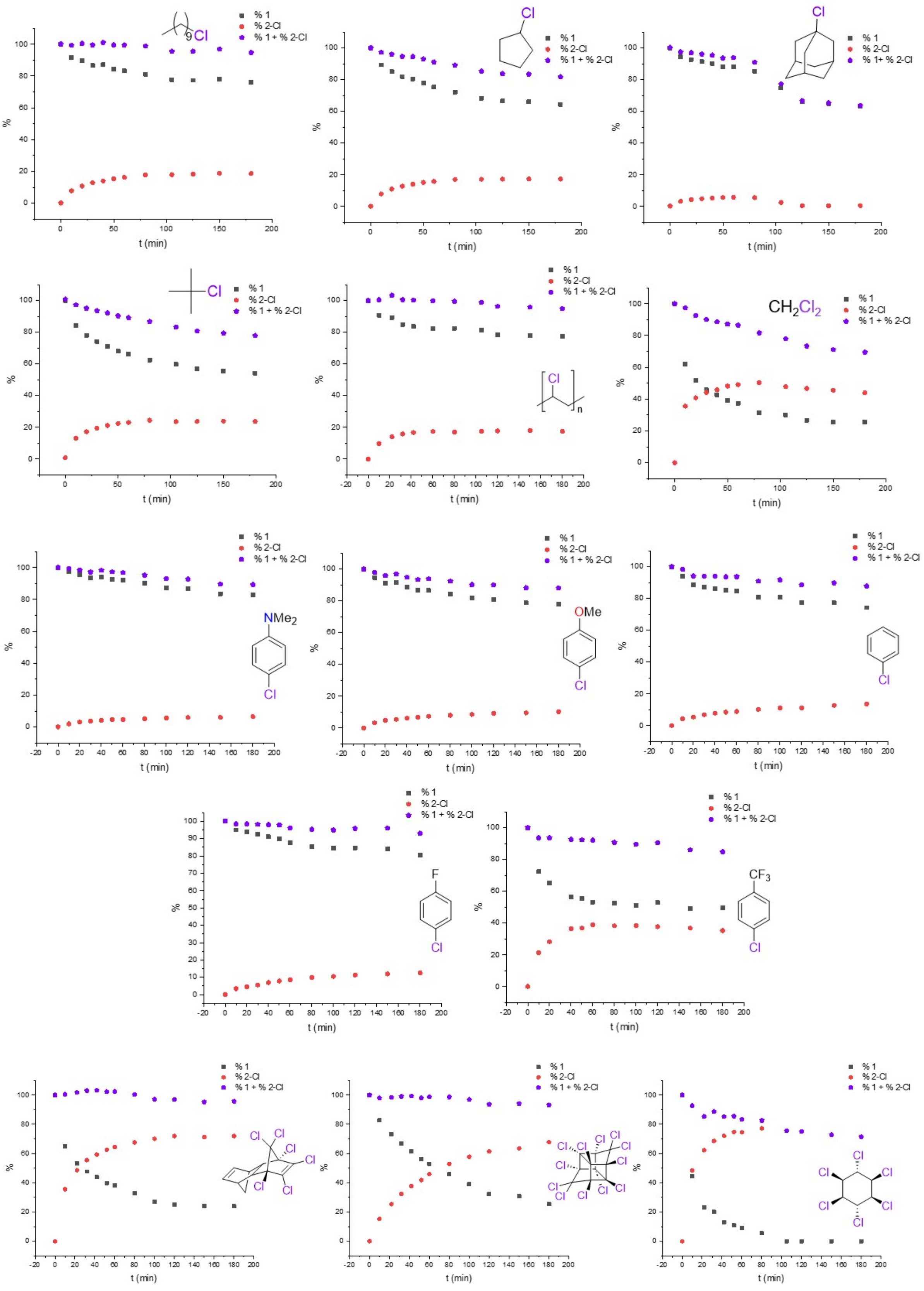

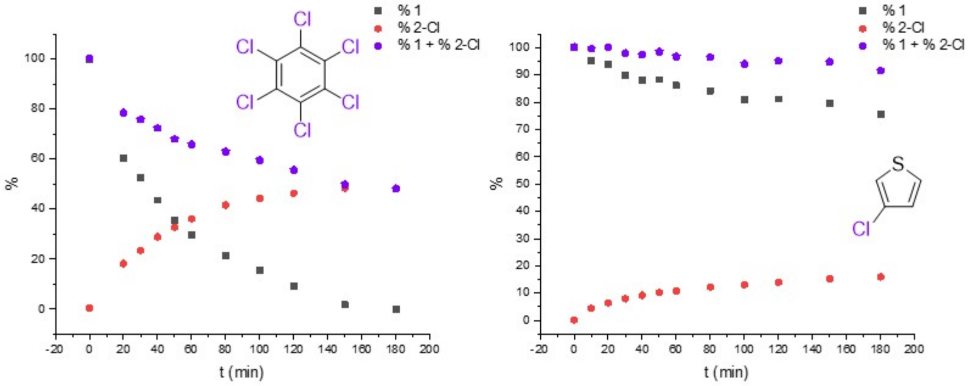

Figure S88. Evolution of quantities of **1**, **2-Cl**, in the function of irradiation time with various chlorinated substrates. Yields of formation of **2-Cl**, were calculated using the integration of a characteristic signal of the internal standard in ^{1}H NMR and calibrated on the initial integration of **1**

Note S1. For **Table S15**, yields of formation of **2-Cl**, were calculated using the integration of a characteristic signal of internal standard in ^{1}H NMR and calibrated on the initial integration of **1.**

CH_2Cl_2

t (min)	% **1**	% 2-Cl	% RX	% **1** + % **2-Cl**
0	100	0	100	100.0
10	61.89	35.55	79.64	97.44
20	51.81	40.84	72.16	92.65
30	45.94	44.17	69.58	90.11
40	42.72	45.88	67.15	88.6
E50	39.06	48.18	65.00	87.24
60	37.4	49.12	63.58	86.52
80	31.47	50.32	60.60	81.79
105	30.03	47.86	59.12	77.89
125	26.76	46.69	56.11	73.45
150	25.54	45.58	53.43	71.12
180	25.48	44.05	51.83	69.53
240	18.3	39.76	42.85	58.06

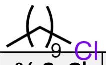

t (min)	% **1**	% 2-Cl	% **1** + % **2-Cl**
0	100	0.2	100.2
10	91.61	7.77	99.38
20	89.75	10.77	100.5
30	86.69	12.9	99.59
40	87.22	13.95	101.2
50	84.2	15.41	99.61
60	83.22	16.28	99.5
80	81.15	17.77	98.92
105	77.61	17.96	95.57
125	77.34	18.25	95.59
150	78.04	18.92	96.96
180	76.06	18.71	94.77
240	31.23	3.87	35.10

Table S14. Percentages of compounds 1 and 2-Cl during photochemical degradation of dichloromethane and 2-1-chlorodecane

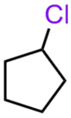

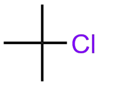

t (min)	% **1**	% 2-Cl	% **1** + % **2-Cl**
0	100	0.15	100.2
10	89.35	7.95	97.3
20	85.16	10.98	96.14
30	81.95	12.8	94.75
40	80.56	13.98	94.54
50	77.92	15.14	93.06
60	75.28	15.83	91.11
80	72.12	17.04	89.16
105	68.28	17.14	85.42
125	66.48	17.32	83.8
150	66.08	17.42	83.5
180	64.46	17.3	81.76
240	23.73	3.3	27.03

t (min)	% **1**	% 2-Cl	% **1** + % **2-Cl**
0	100	0.81	100.8
10	84.11	13.14	97.25
20	77.97	17.21	95.18
30	74.19	19.45	93.64
40	71.14	21.14	92.28
50	68	22.41	90.41
60	66.14	23.02	89.16
80	62.41	24.4	86.81
105	59.68	23.6	83.28
125	57.03	23.68	80.71
150	55.49	23.87	79.36
180	54.11	23.72	77.83
240	46.07	22.99	69.06

Table S15. Percentages of compounds 1 and 2-Cl during photochemical degradation of chlorocyclopentane and 2-methyl-2-chloropropane

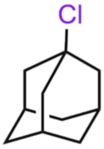

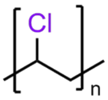

t (min)	% **1**	% 2-Cl	% RX	% **1** + % **2-Cl**
0	100	0.28	100	100.3
10	94.38	3.21	85.13	97.59
20	92.71	4.3	78.73	97.01
30	91.37	4.84	72.66	96.21
40	90.16	5.29	68.09	95.45
50	88.12	5.63	64.18	93.75
60	88.09	5.79	61.48	93.88
80	85.4	5.58	54.92	90.98
105	74.78	2.51	46.42	77.29
125	66.28	0.5	40.73	66.78
150	64.93	0.44	36.32	65.37
180	63.18	0.54	28.22	63.72
240	60.57	0.39	16.78	60.96

t (min)	% **1**	% 2-Cl	% **1** + % **2-Cl**
0	100	0	100.0
10	90.78	9.65	100.4
22	89.3	14.03	103.3
32	84.82	15.82	100.6
42	83.54	16.69	100.2
60	82.46	17.39	99.85
80	82.5	17.04	99.54
105	81.38	17.51	98.89
120	78.56	17.72	96.28
150	77.88	17.94	95.82
180	77.46	17.43	94.89

Table S16. Percentages of compounds 1 and 2-Cl during photochemical degradation of chloroadamantane and PVC

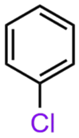

t (min)	% **1**	% 2-Cl	% **1** + % **2-Cl**
0	100	0.09	100.1
10	94	4.36	98.36
20	88.72	5.47	94.19
30	87.17	6.87	94.04
40	86.23	7.82	94.05
50	85.17	8.47	93.64
60	84.87	8.93	93.8
80	80.7	10.25	90.95
100	80.68	11.13	91.81
120	77.43	11.17	88.6
150	77.2	12.72	89.92
180	74.22	13.63	87.85

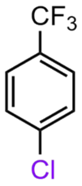

t (min)	% **1**	% 2-Cl	% **1** + % **2-Cl**
0	100	0.16	100.2
10	72.32	21.32	93.64
20	65.45	28.31	93.76
40	56.35	36.4	92.75
50	55.52	36.88	92.4
60	53.25	38.93	92.18
80	52.59	38.26	90.85
100	51.21	38.39	89.6
120	52.85	37.77	90.62
150	49.24	36.81	86.05
180	49.63	35.28	84.91

Table S17. Percentages of compounds **1** and **2-Cl** during photochemical degradation of chlorobenzene and 4-chloro-α,α,α-trifluorotoluene

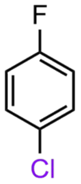

t (min)	% **1**	% 2-Cl	% **1** + % **2-Cl**
0	100	0.14	100.1
10	95	3.45	98.45
20	93.84	4.61	98.45
30	92.7	5.59	98.29
40	91.05	6.99	98.04
50	89.99	7.89	97.88
60	87.52	8.58	96.1
80	85.53	9.84	95.37
100	84.51	10.54	95.05
120	84.66	11.32	95.98
150	84.12	11.94	96.06
180	80.52	12.56	93.08

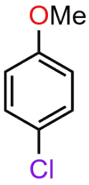

t (min)	% **1**	% 2-Cl	% **1** + % **2-Cl**
0	100	0	100.0
10	94.67	3.2	97.87
20	90.98	4.88	95.86
30	91.44	5.43	96.87
40	88.71	6.15	94.86
50	86.56	6.79	93.35
60	86.51	7.37	93.88
80	84.39	7.97	92.36
100	81.69	8.52	90.21
120	80.84	9.15	89.99
150	78.73	9.49	88.22
180	77.9	10.18	88.08

Table S18. Percentages of compounds **1** and **2-Cl** during photochemical degradation of 4-fluoro-chlorobenzene and 4-chloroanisole

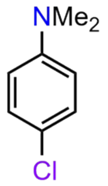

t (min)	% **1**	% 2-Cl	% **1** + % **2-Cl**
0	100	0.1	100.1
10	97.49	1.97	99.46
20	95.31	3.12	98.43
30	93.54	3.76	97.3
40	94.11	4.21	98.32
50	92.69	4.66	97.35
60	92.28	4.62	96.9
80	90.22	5.11	95.33
100	87.38	5.7	93.08
120	86.85	6.01	92.86
150	83.6	6.04	89.64
180	82.95	6.47	89.42

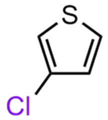

t (min)	% **1**	% 2-Cl	% RX	% **1** + % **2-Cl**
0	100	0.22	100	100.2
10	95.24	4.41	93.72	99.65
20	93.77	6.42	90.74	100.19
30	90.06	7.94	90.89	98
40	88.23	9.22	87.91	97.45
50	88.32	10.23	87.91	98.55
60	86.09	10.74	84.93	96.83
80	84.25	12.24	83.91	96.49
100	80.96	13.13	81.71	94.09
120	81.15	14	80.06	95.15
150	79.54	15.32	74.96	94.86
180	75.59	16.03	72.06	91.62

Table S19. Percentages of compounds **1** and **2-Cl** during photochemical degradation of 4-chloro-dimethylaniline and 2-chlorothiophene

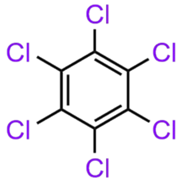

t (min)	% **1**	% 2-Cl	% **1** + % **2-Cl**
0	100	0.34	100.3
10	82.23	15.56	97.79
20	60.29	18.19	78.48
30	52.41	23.46	75.87
40	43.53	28.9	72.43
50	35.31	32.75	68.06
60	29.8	36.14	65.94
80	21.42	41.64	63.06
100	15.38	44.27	59.65
120	9.33	46.36	55.69
150	1.65	48.31	49.96
180	0	48.2	48.2

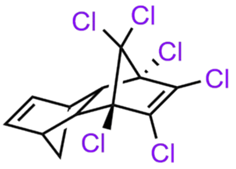

t (min)	% **1**	% 2-Cl	% **1** + % **2-Cl**
0	100	0	100
10	65.05	35.54	100.6
22	53.19	48.56	101.8
32	47.68	55.47	103.2
42	43.94	59.28	103.2
52	39.78	62.58	102.4
60	38.11	64.38	102.5
80	32.82	67.63	100.5
100	27.04	70.12	97.16
120	25.03	72	97.03
150	24.14	71.24	95.38
180	23.86	72	95.86

Table S20. Percentages of compounds **1** and **2-Cl** during photochemical degradation of hexachlorobenzene and Aldrin

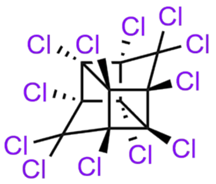

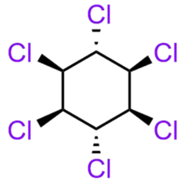

t (min)	% **1**	% 2-Cl	% **1** + % **2-Cl**
0	100	0	100
10	82.72	15.28	98
22	73.08	25.39	98.47
32	66.78	32.41	99.19
42	61.64	37.77	99.41
52	56.28	41.84	98.12
60	52.92	45.95	98.87
80	45.89	52.86	98.75
100	39.13	57.78	96.91
120	32.26	61.46	93.72
150	30.72	63.52	94.24
180	25.48	67.82	93.3

t (min)	% **1**	% 2-Cl	% **1** + % **2-Cl**
0	100	0	100.0
10	44.42	48.34	92.76
22	23.07	62.35	85.42
32	20.05	68.68	88.73
42	13.09	72.15	85.24
52	10.88	74.77	85.65
60	8.86	74.54	83.4
80	5.46	77.27	82.73
105	0	75.64	75.64
120	0	75.16	75.16
150	0	72.87	72.87
180	0	71.5	71.5

Table S21. Percentages of compounds 1 and 2-Cl during photochemical degradation of Mirex and Lindane

1) NMR spectra

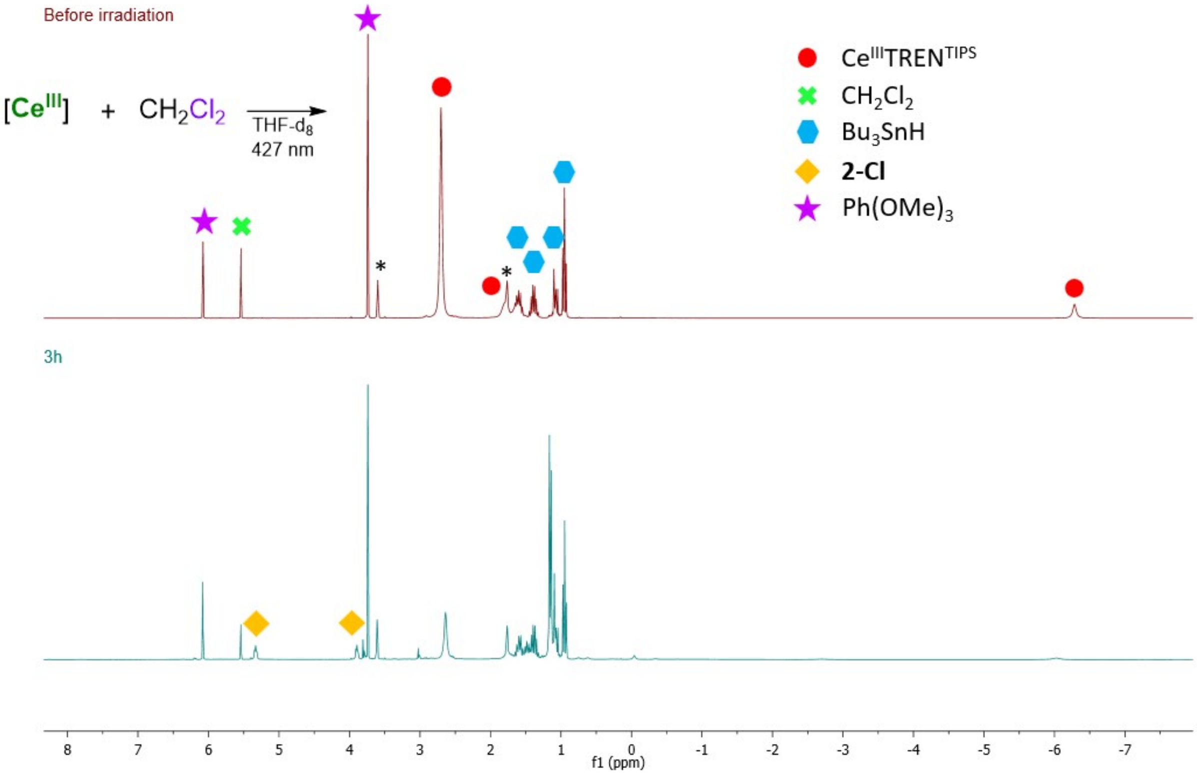

Figure S89. ^{1}H NMR spectrum of the reaction between **1** and dichloromethane under irradiation at 427 nm in THF-d$_8$. Solvent residual peaks are indicated with *

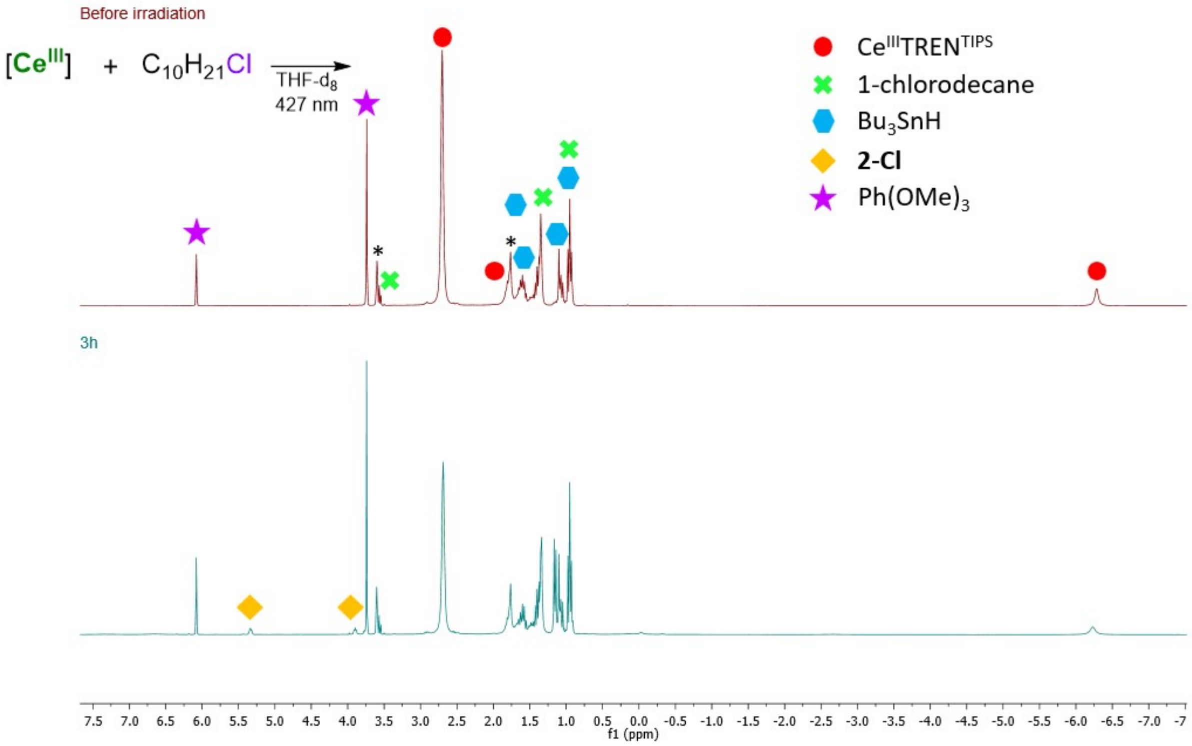

Figure S90. ^{1}H NMR spectrum of the reaction between **1** and 1-chlorodecane under irradiation at 427 nm in THF-d$_8$. Solvent residual peaks are indicated with *

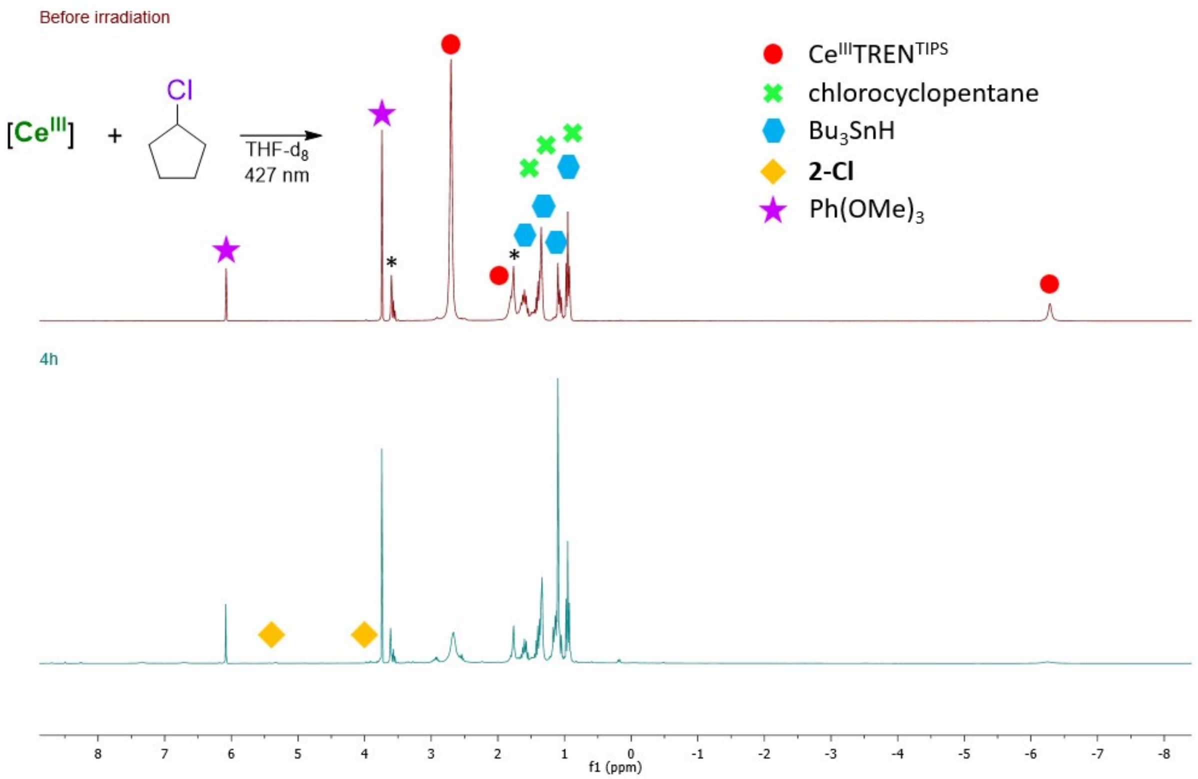

Figure S91. ^{1}H NMR spectrum of the reaction between **1** and chlorocyclopentane under irradiation at 427 nm in THF-d$_8$. Solvent residual peaks are indicated with *

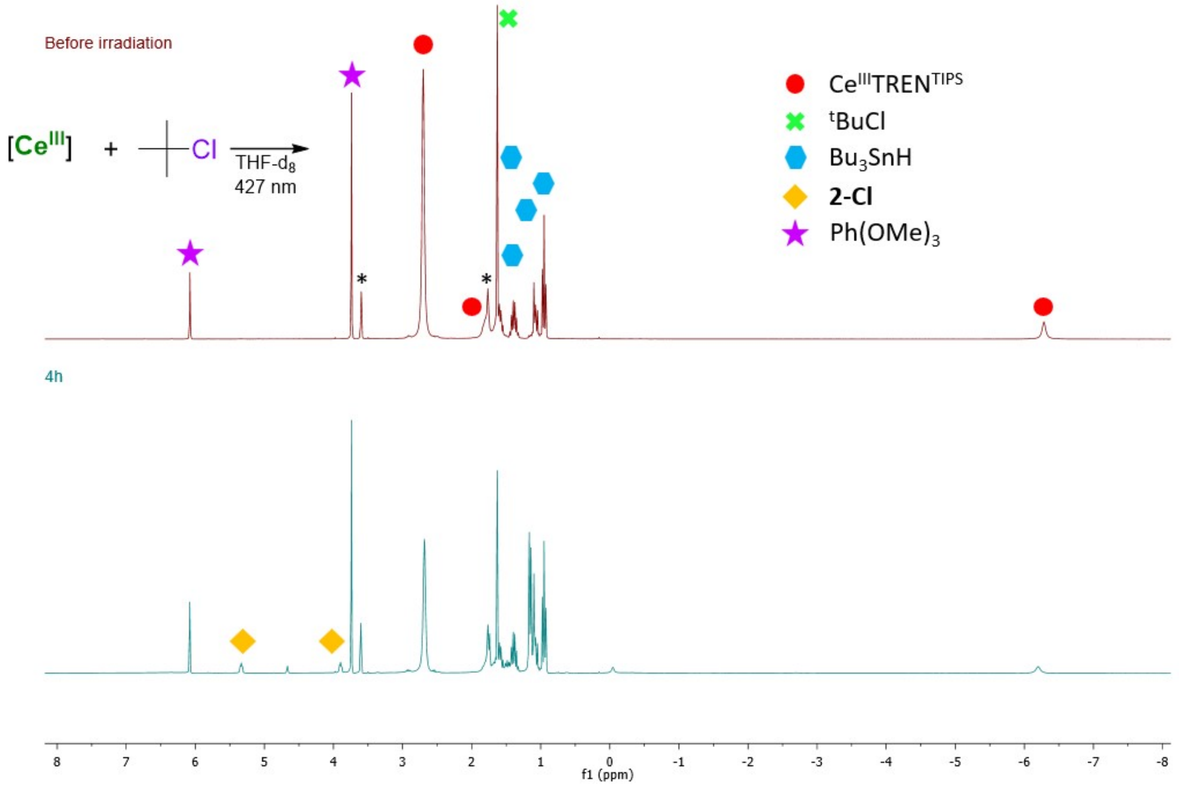

Figure S92. ^{1}H NMR spectrum of the reaction between **1** and 2-chloro-2-methyl-propane under irradiation at 427 nm in THF-d$_8$. Solvent residual peaks are indicated with *

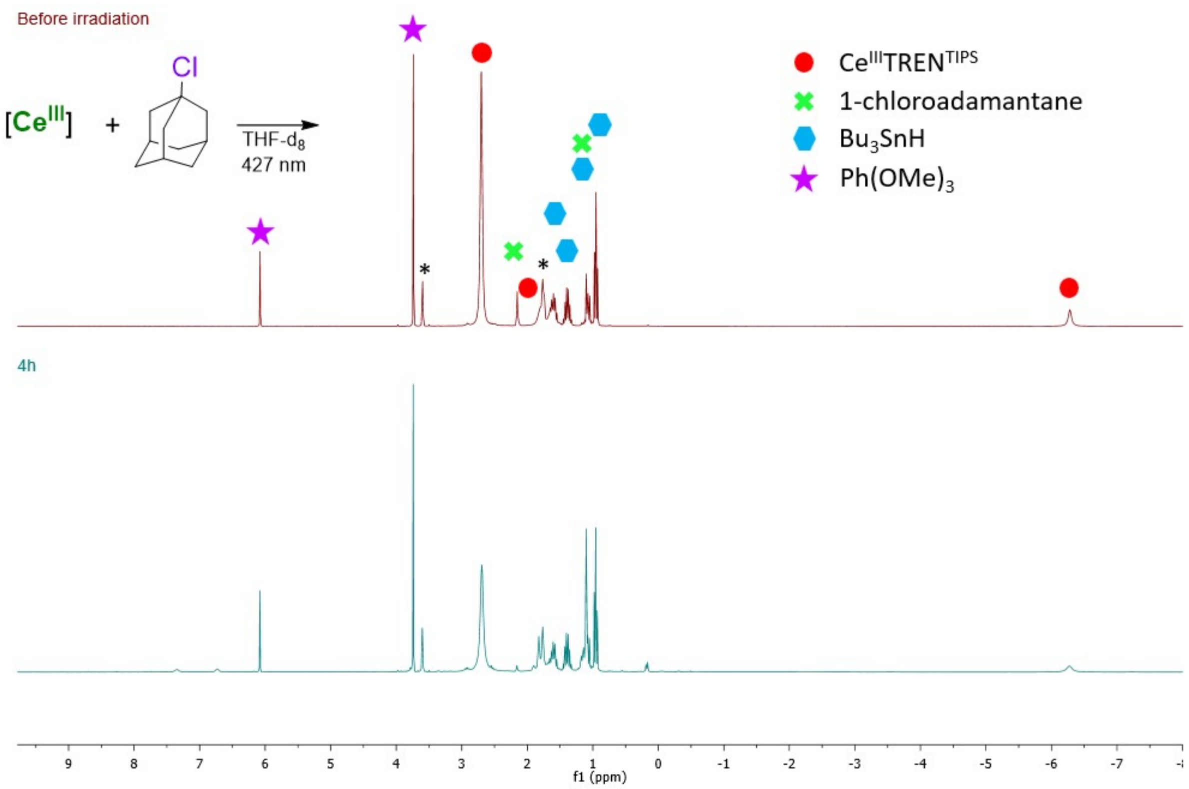

Figure S93. ^{1}H NMR spectrum of the reaction between **1** and 1-chloroadamantane under irradiation at 427 nm in THF-d$_8$. Solvent residual peaks are indicated with *

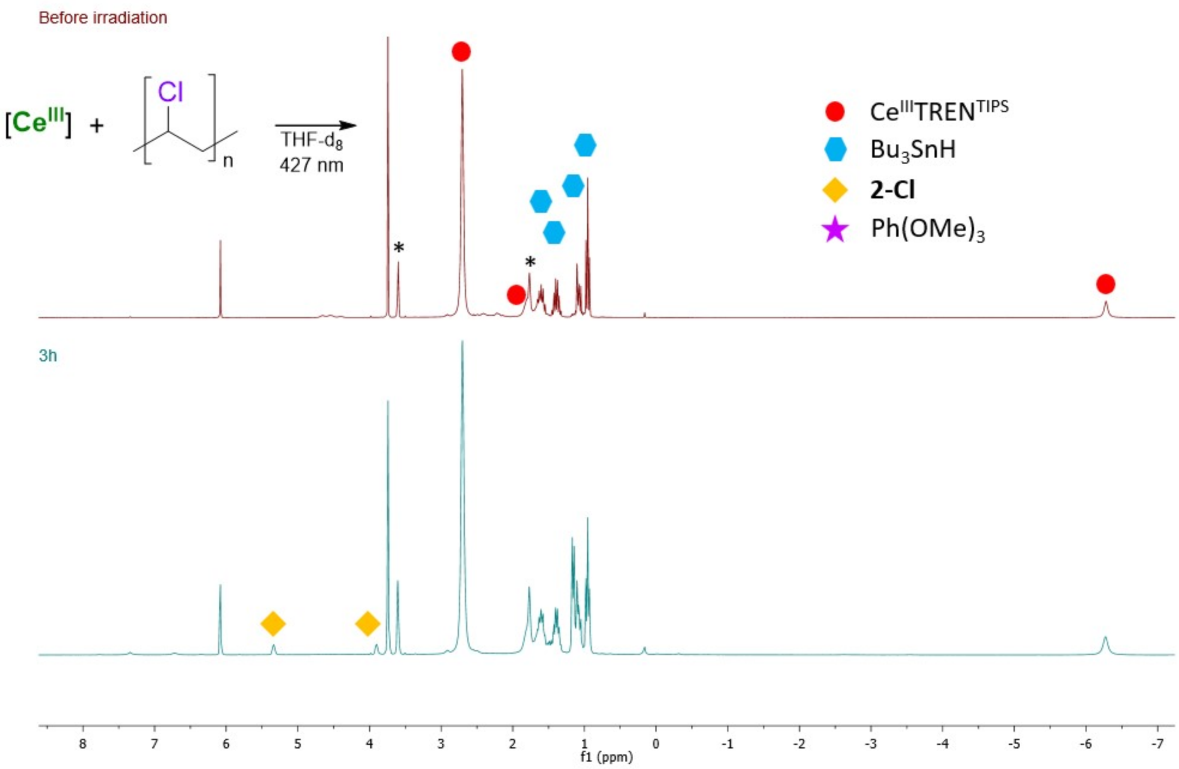

Figure S94. ^{1}H NMR spectrum of the reaction between **1** and PVC under irradiation at 427 nm in THF-d$_8$. Solvent residual peaks are indicated with *

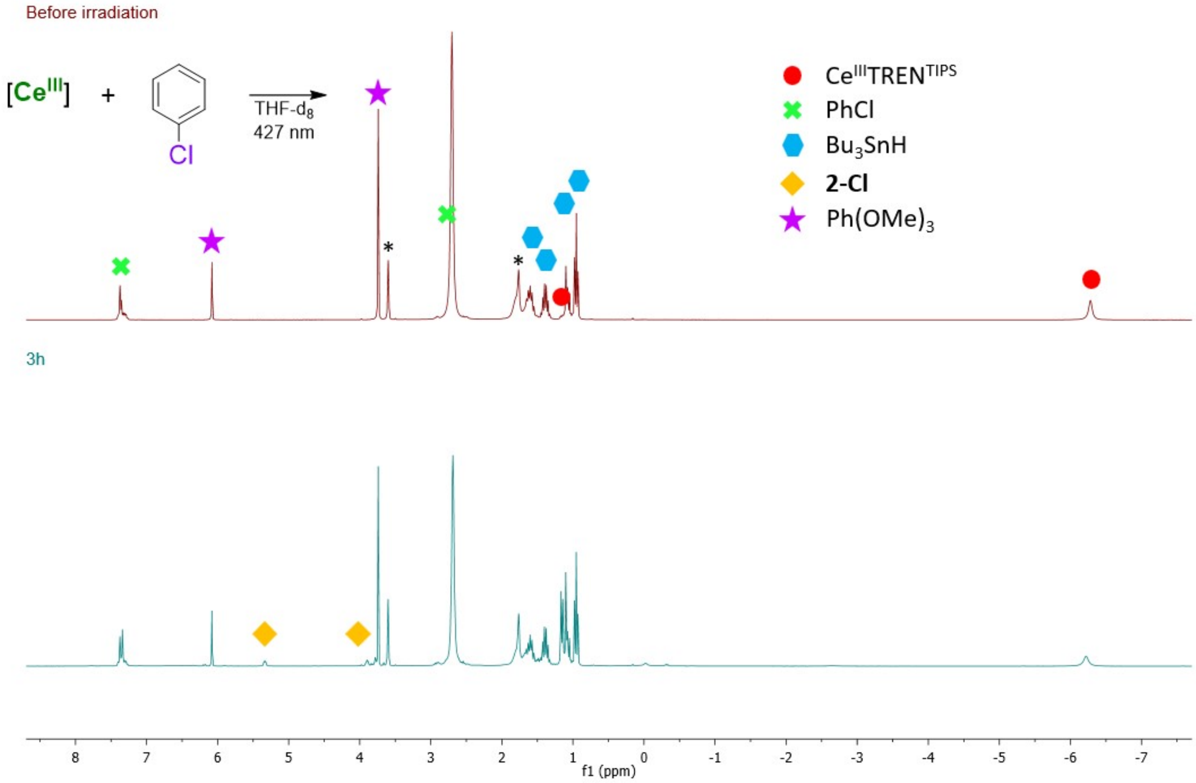

Figure S95. ^{1}H NMR spectrum of the reaction between **1** and chlorobenzene under irradiation at 427 nm in THF-d_8. Solvent residual peaks are indicated with *

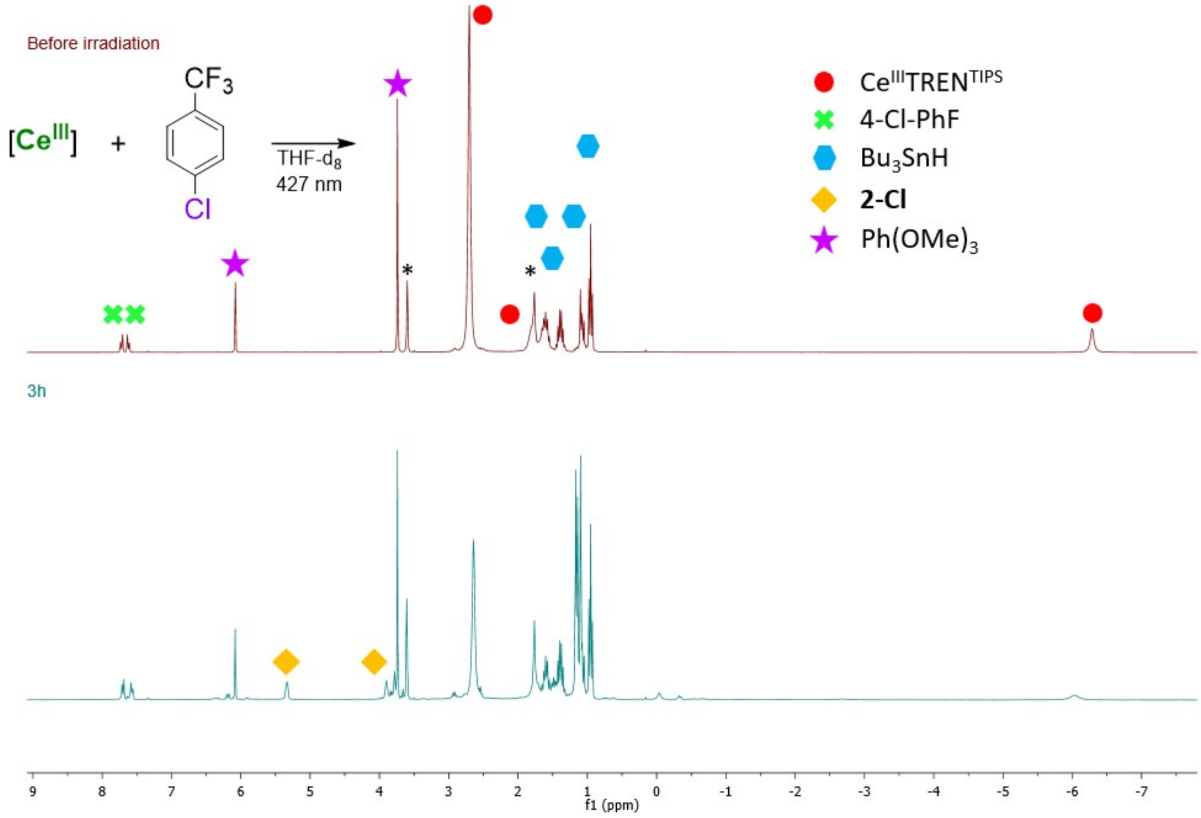

Figure S96. ^{1}H NMR spectrum of the reaction between **1** and 4-chloro-trifluoromethylbenzene under irradiation at 427 nm in THF-d_8. Solvent residual peaks are indicated with *

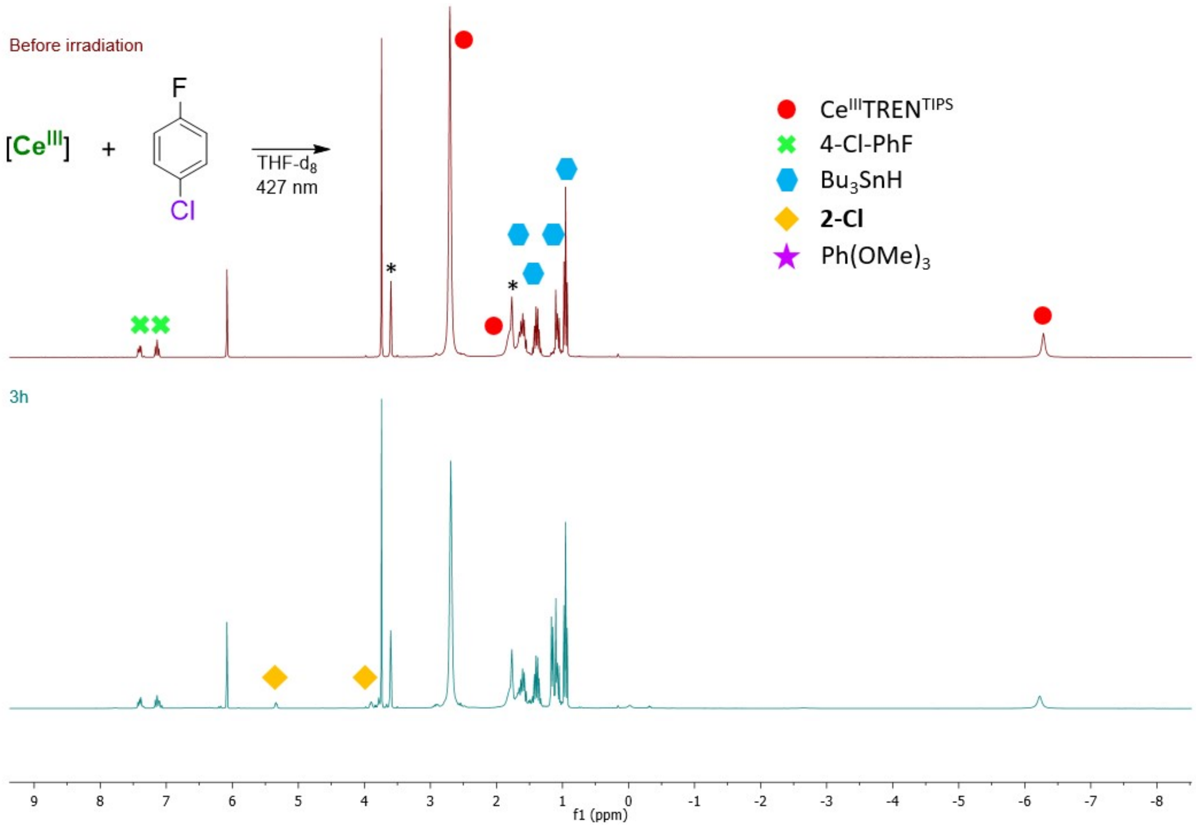

Figure S97. ^{1}H NMR spectrum of the reaction between **1** and 4-fluoro-chlorobenzene under irradiation at 427 nm in THF-d_8. Solvent residual peaks are indicated with *

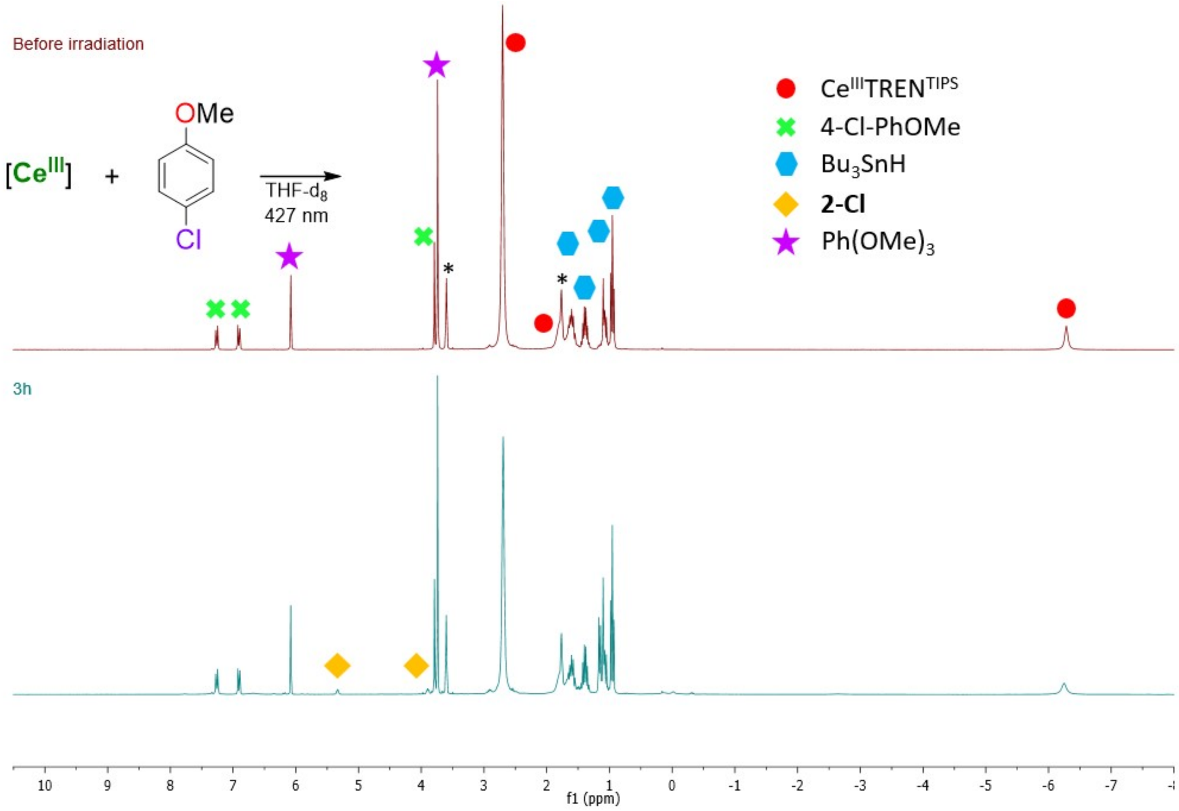

Figure S98. ^{1}H NMR spectrum of the reaction between **1** and 4-chloroanisole under irradiation at 427 nm in THF-d_8. Solvent residual peaks are indicated with *

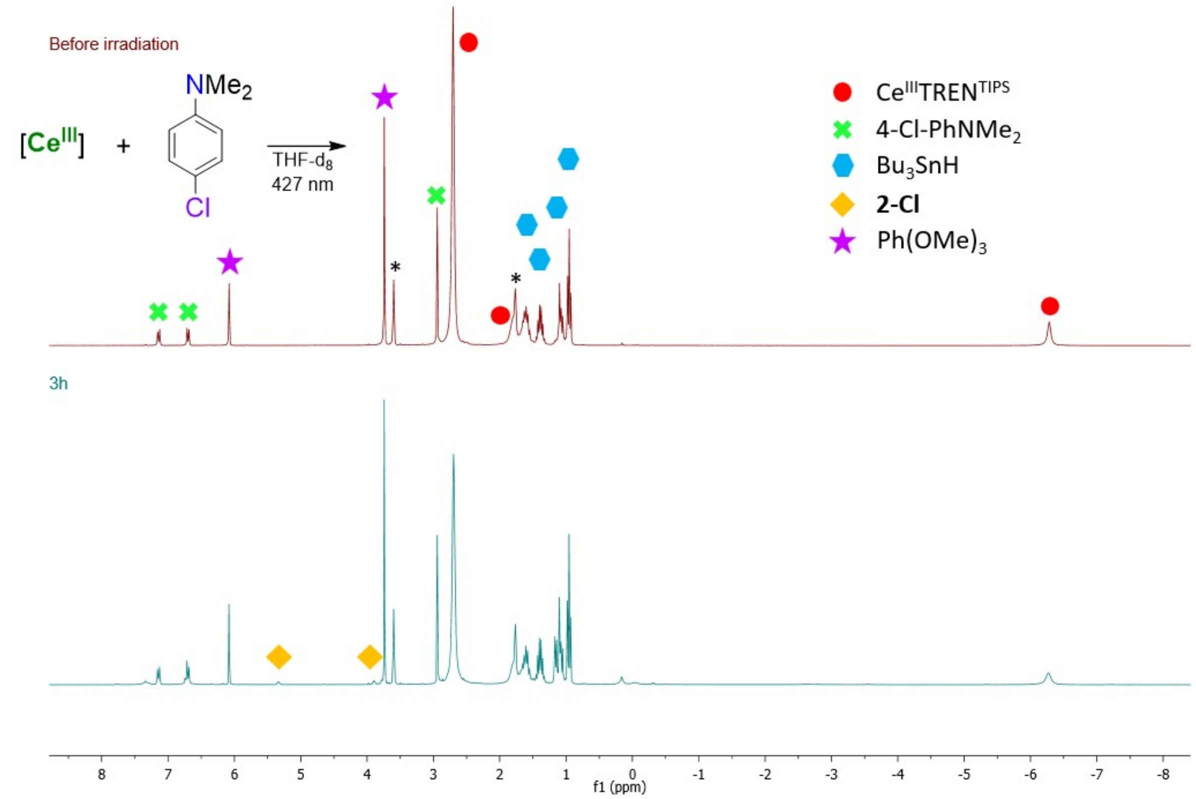

Figure S99. ^{1}H NMR spectrum of the reaction between **1** and 4-chloro-dimethylaniline under irradiation at 427 nm in THF-d_8. Solvent residual peaks are indicated with *

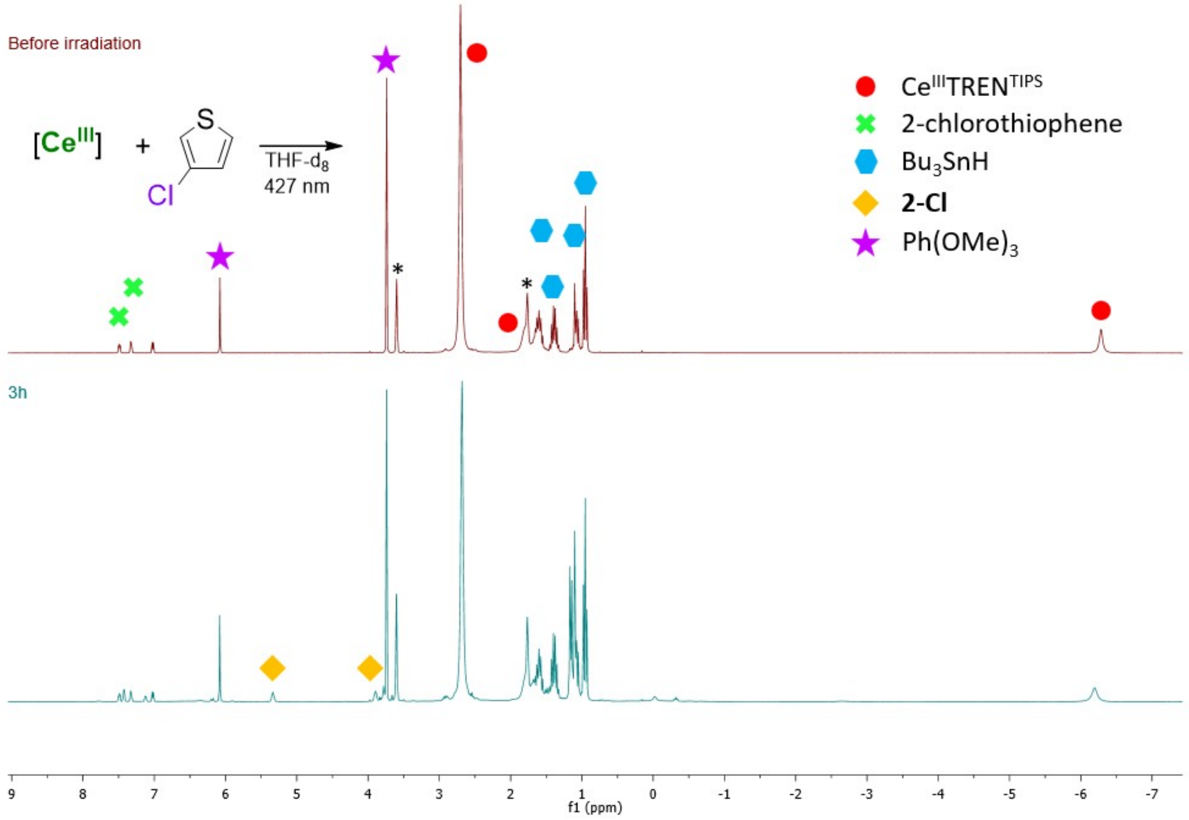

Figure S100. ^{1}H NMR spectrum of the reaction between **1** and 2-chlorothiophene under irradiation at 427 nm in THF-d_8. Solvent residual peaks are indicated with *

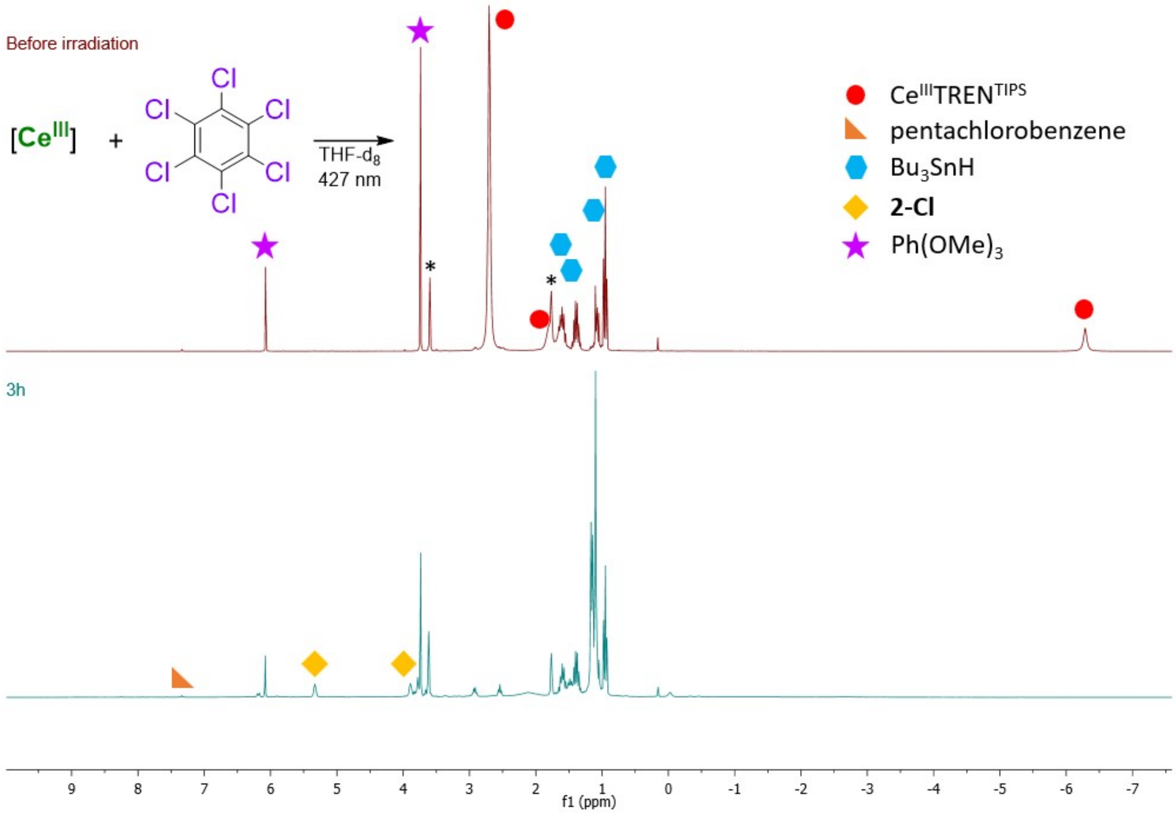

Figure S101. ^{1}H NMR spectrum of the reaction between **1** and hexachlorobenzene under irradiation at 427 nm in THF-d$_8$. Solvent residual peaks are indicated with *

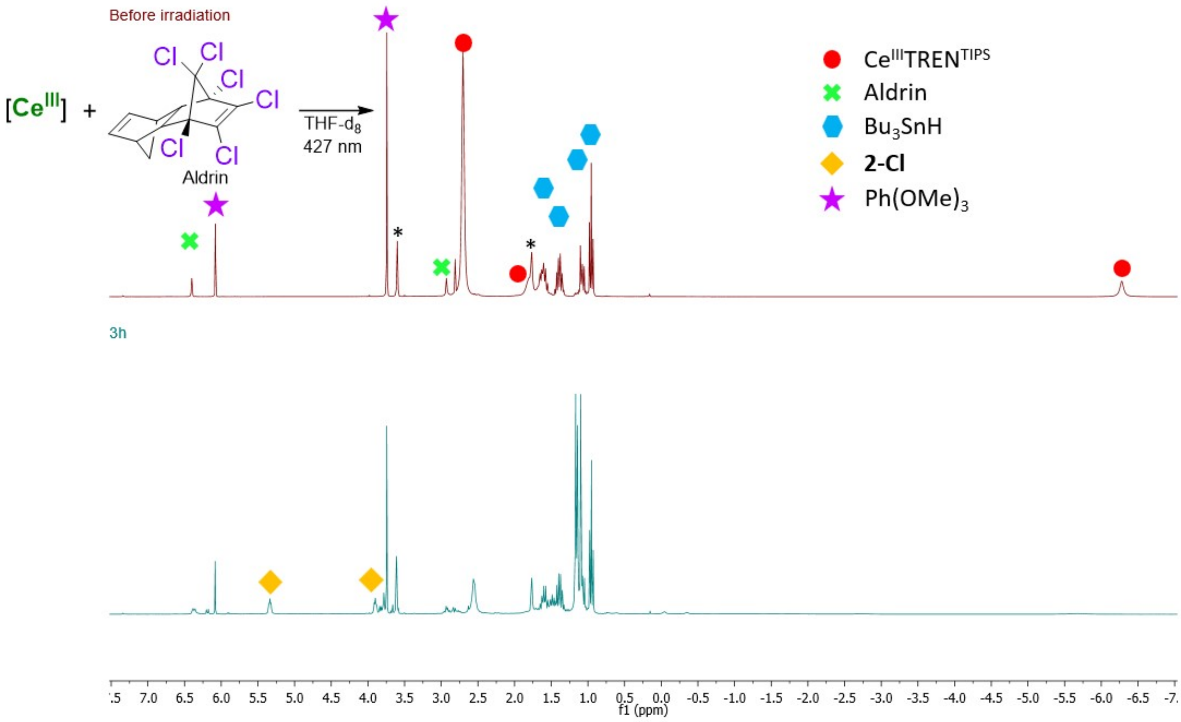

Figure S102. ^{1}H NMR spectrum of the reaction between **1** and Aldrin under irradiation at 427 nm in THF-d$_8$. Solvent residual peaks are indicated with *

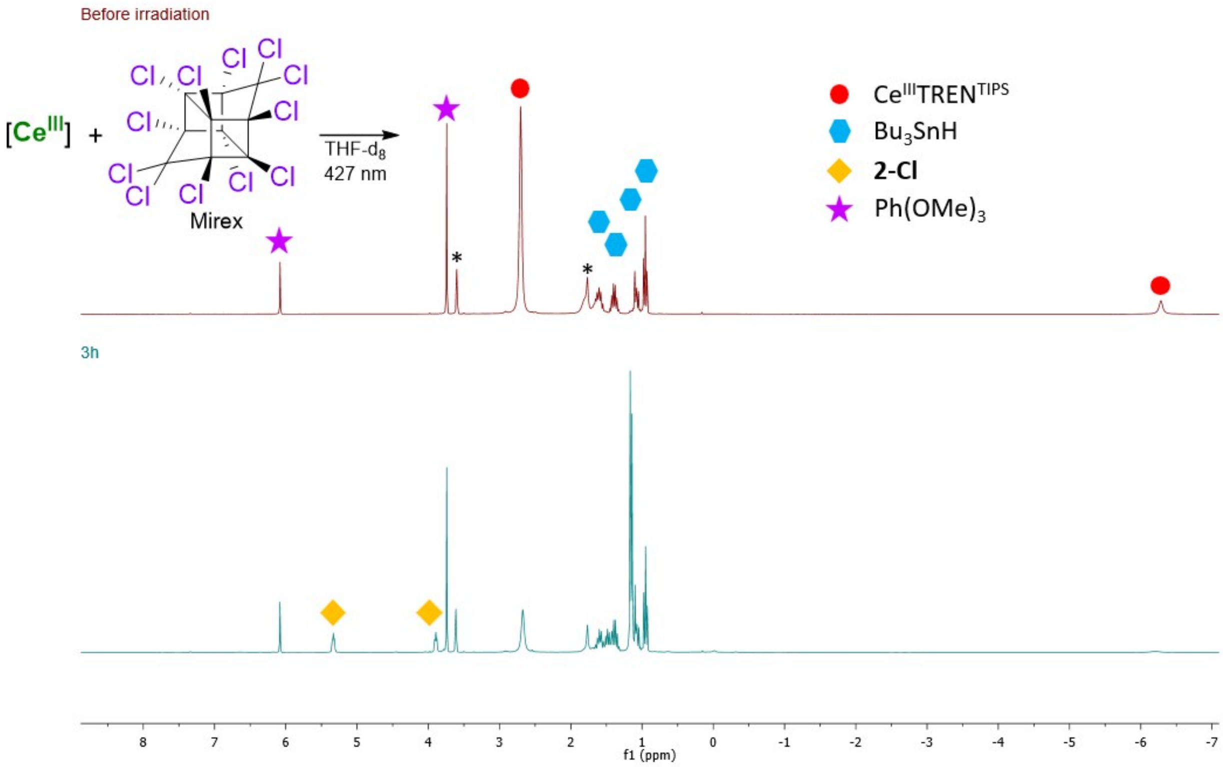

Figure S103. ^{1}H NMR spectrum of the reaction between **1** and Mirex under irradiation at 427 nm in THF-d_8. Solvent residual peaks are indicated with *

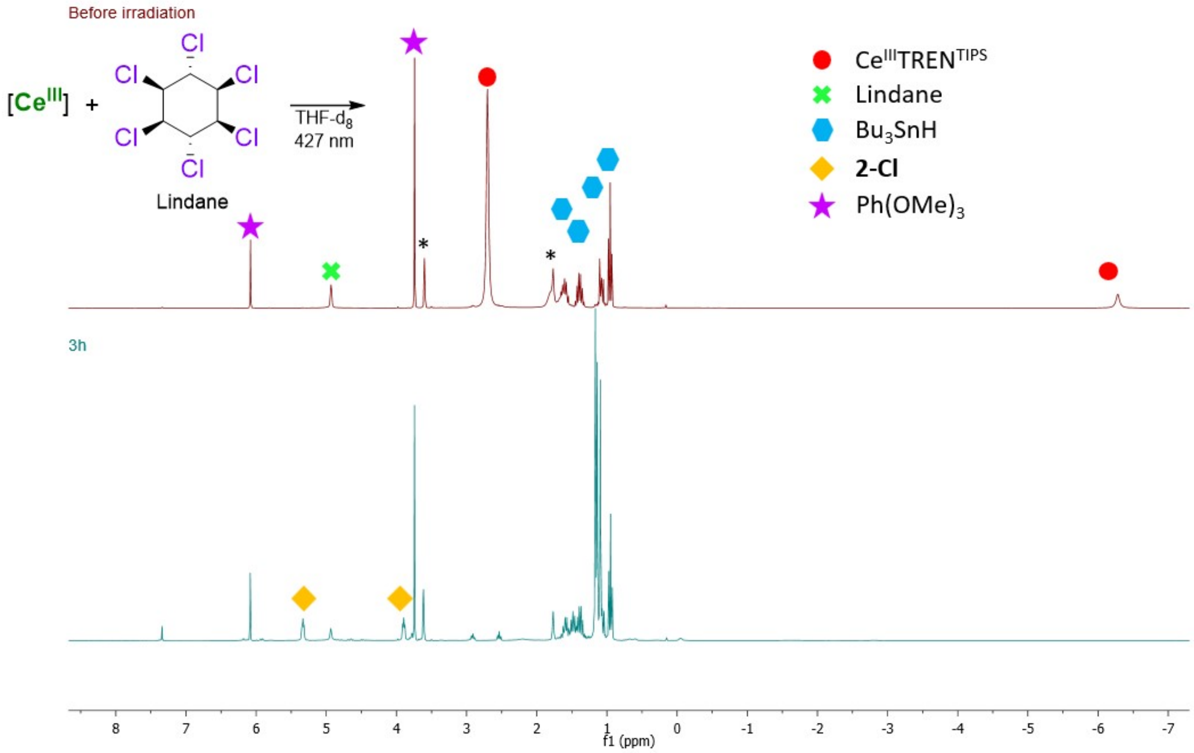

Figure S104. ^{1}H NMR spectrum of the reaction between **1** and Lindane under irradiation at 427 nm in THF-d_8. Solvent residual peaks are indicated with *

e) Reactivity with fluorinated substrates

1) Kinetic data

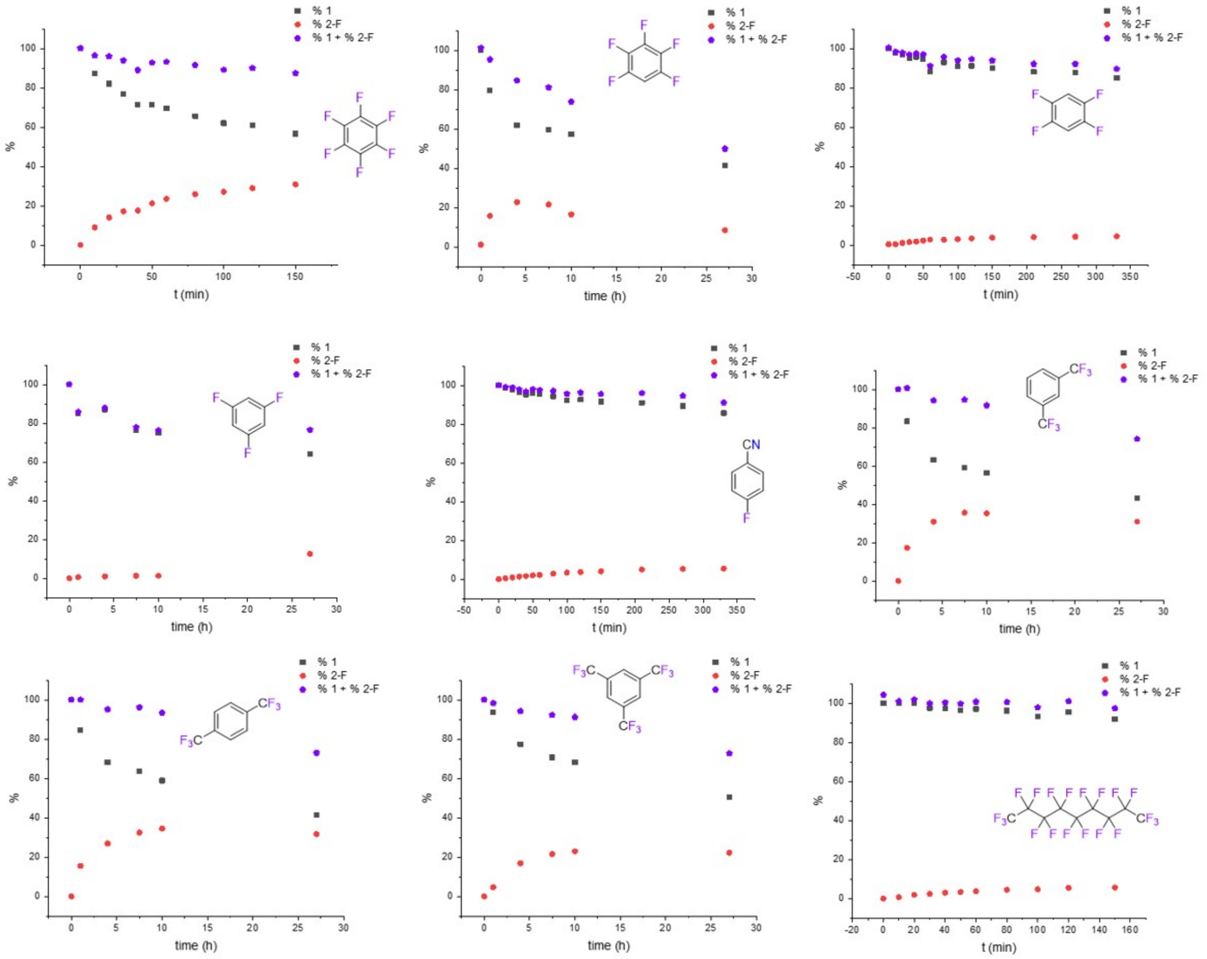

Figure S105. Evolution of quantities of **1**, **2-F** as function of irradiation time with various chlorinated substrates. Yields of formation of **2-F**, were calculated using the integration of a characteristic signal of the internal standard in ^{1}H NMR and calibrated on the initial integration of **1**

Note S1. For **Table S24**, yields of formation of **2-F** were calculated using the integration of a characteristic signal of internal standard in ^{1}H NMR and calibrated on the initial integration of **1**.

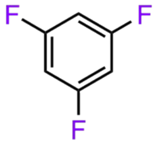

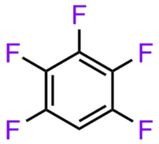

t (h)	% **1**	% **2-F**	% RX	% **1** + % **2-F**
0	100	0	100	100.0
1	85.17	0.63	98.68	85.80
4	87.07	0.95	97.37	88.01
7.5	76.66	1.26	100.00	77.92
10	75.08	1.26	98.68	76.34
27	64.04	12.62	92.11	76.66

t (h)	% **1**	% **2-F**	% RX	% **1** + % **2-F**
0	100	0	100	100.0
1	79.62	15.77	93.10	95.38
4	61.92	22.69	86.21	84.62
7.5	59.62	21.54	79.31	81.15
10	57.31	16.54	82.76	73.85
27	41.54	8.46	72.41	50.00

Table S22. Percentages of compounds **1** and **2-F** during photochemical degradation of 1,3,5-trifluorobenzene and pentafluorobenzene

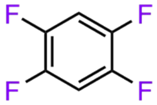

t (min)	% **1**	% **2-F**	% RX	% **1** + % **2-F**
0	100	0.45	100	100.5
10	97.86	0.5	102.51	98.36
20	96.7	1.15	100.04	97.85
30	95.19	1.66	101.44	96.85
40	95.67	1.81	99.33	97.48
50	94.71	2.28	97.49	96.99
60	88.32	2.83	95.49	91.15
80	92.94	2.75	98.00	95.69
100	90.93	3	95.19	93.93
120	91.16	3.39	95.49	94.55
150	90.17	3.74	94.12	93.91
210	88.11	4.04	93.90	92.15
270	87.89	4.29	94.20	92.18
330	85.07	4.48	93.49	89.55

t (min)	% **1**	% **2-F**	% RX	% **1** + % **2-F**
0	100	0	100	100.0
30	98.05	0.22	100.71	98.27
60	95.47	1.02	98.39	96.49
180	91.67	2.63	93.05	94.3
240	89.96	2.88	90.61	92.84
300	89.14	3.12	88.84	92.26
360	87.81	2.93	87.56	90.74
420	87.57	3	84.53	90.57
480	84.52	3	83.83	87.52
540	85.16	2.97	83.31	88.13
600	86	2.05	79.97	88.05
660	84.39	2.22	78.17	86.61
720	83.77	2.43	77.30	86.2

Table S23. Percentages of compounds **1** and **2-F** during photochemical degradation of 1,2,4,5-tetrafluorobenzene

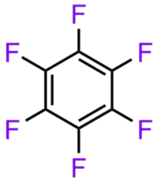

t (min)	% **1**	% **2-F**	% **1** + % **2-F**
0	100	0.04	100.0
10	87.32	9.01	96.33
20	82.01	14.04	96.05
30	76.8	17.17	93.97
40	71.46	17.58	89.04
50	71.48	21.25	92.73
60	69.62	23.54	93.16
80	65.64	25.93	91.57
100	62.03	27.13	89.16
120	61.02	28.97	89.99
150	56.56	30.9	87.46
210	42.86	24.83	67.69
270	36.71	26.08	62.79
330	32.67	26.63	59.3

t (min)	% **1**	% **2-F**	% **1** + % **2-F**
0	100	0	100.0
30	91.24	6.42	97.66
60	79.05	16.42	95.47
120	66.45	24.71	91.16
180	59.93	29.37	89.3
240	56.86	33.25	90.11
300	51.4	34.9	86.3
360	49.39	35.91	85.3
420	46.74	37.43	84.17
480	44.11	38.58	82.69
540	41.59	38.82	80.41
600	41.03	37.76	78.79
660	41.73	38.1	79.83
720	38.17	38.37	76.54

Table S24. Percentages of compounds **1** and **2-F** during photochemical degradation of hexafluorobenzene

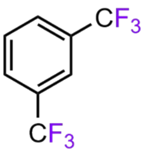

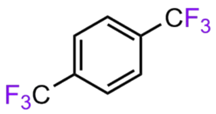

t (min)	% **1**	% **2-F**	% RX	% **1** + % **2-F**
0	100	0	100	100.0
1	83.45	17.27	86.73	100.7
4	63.31	30.94	73.47	94.24
7.5	58.99	35.61	66.33	94.60
10	56.47	35.25	64.29	91.73
27	43.17	30.94	46.94	74.10

t (min)	% **1**	% **2-F**	% RX	% **1** + % **2-F**
0	100	0	100	100.0
1	84.48	15.52	86.67	100.0
4	68.28	26.90	77.04	95.17
7.5	63.79	32.41	71.85	96.21
10	58.97	34.48	68.15	93.45
27	41.38	31.72	46.67	73.10

Table S25. Percentages of compounds **1** and **2-F** during photochemical degradation of 1,3-bis(trifluoromethyl)benzene and1,4-bis(trifluoromethyl)benzene

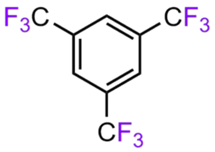

t (min)	% **1**	% **2-F**	% RX	% **1** + % **2-F**
0	100	0	100	100.0
1	93.60	4.71	95.65	98.32
4	77.44	16.84	83.70	94.28
7.5	70.71	21.55	79.35	92.26
10	68.35	22.90	77.17	91.25
27	50.51	22.22	58.70	72.73

Table S26. Percentages of compounds **1** and **2-F** during photochemical degradation of 1,3,5-tris(trifluoromethyl)benzene

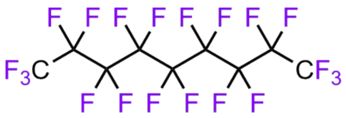

t (min)	% **1**	% 2-F	% **1** + % **2-F**
0	100	0	100.0
10	100.27	0.73	101
20	99.93	1.89	101.8
30	97.54	2.39	99.93
40	97.33	3.01	100.3
50	96.39	3.35	99.74
60	97.1	3.75	100.9
80	96.16	4.43	100.6
100	93.16	4.7	97.86
120	95.56	5.49	101.1
150	91.8	5.67	97.47

t (min)	% **1**	% 2-F	% **1** + % **2-F**
0	100	0	100.0
30	92.21	6.64	98.85
60	87.71	8.93	96.64
120	86.71	10.54	97.25
180	85.38	11.38	96.76
240	85.35	12.56	97.91
300	83.06	13.43	96.49
360	83.26	13.96	97.22
420	81.36	14.23	95.59
480	80.51	14.75	95.26
540	79.49	15.12	94.61
600	79.79	15.63	95.42
720	77.94	16.27	94.21

Table S27. Percentages of compounds **1** and **2-F** during photochemical degradation of perfluorononane

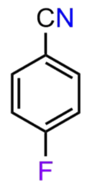

t (min)	% **1**	% 2-F	% **1** + % **2-F**
0	100	0	100.0
10	98.75	0.4	99.15
20	98.06	0.88	98.94
30	96.65	1.3	97.95
40	95.2	1.58	96.78
50	96.09	1.94	98.03
60	95.39	2.18	97.57
80	94.34	2.86	97.2
100	92.36	3.35	95.71
120	92.61	3.7	96.31
150	91.56	4.02	95.58
210	91.04	4.97	96.01
270	89.3	5.27	94.57
330	85.73	5.45	91.18

t (min)	% **1**	% 2-F	% **1** + % **2-F**
0	100	0	100.0
30	97.58	0.21	97.79
120	93.68	1.83	95.51
180	90.79	2.82	93.61
240	90.85	3.12	93.97
300	89.02	3.44	92.46
360	87.94	3.7	91.64
420	88.38	3.81	92.19
480	88.38	3.87	92.25
540	87.25	3.82	91.07
600	86.61	3.47	90.08
660	84.86	3.88	88.74
720	84.74	3.77	88.51

Table S28. Percentages of compounds **1** and **2-F** during photochemical degradation of 4-fluorobenzonitrile

2) NMR spectra

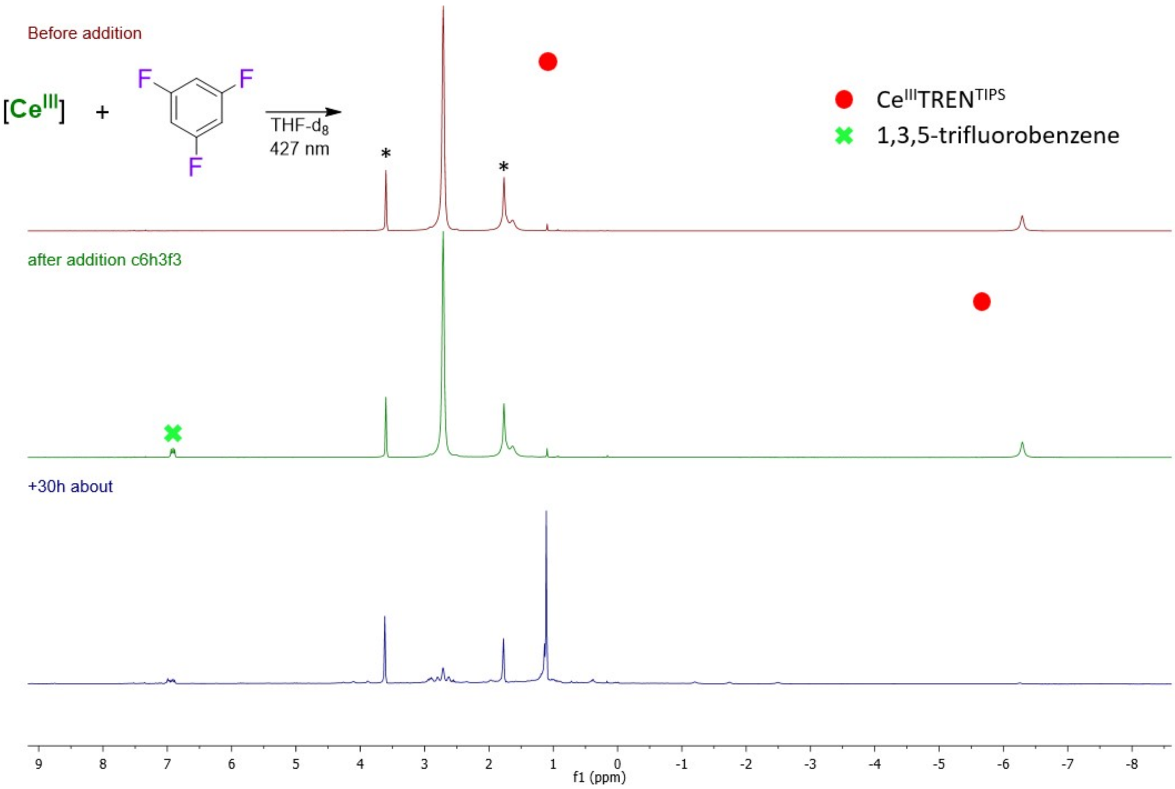

Figure S106. ^{1}H NMR spectrum of the **r**eaction between **1** and 1,3,5-trifluorobenzene under irradiation at 427 nm in THF-d_8. Solvent residual peaks are indicated with *

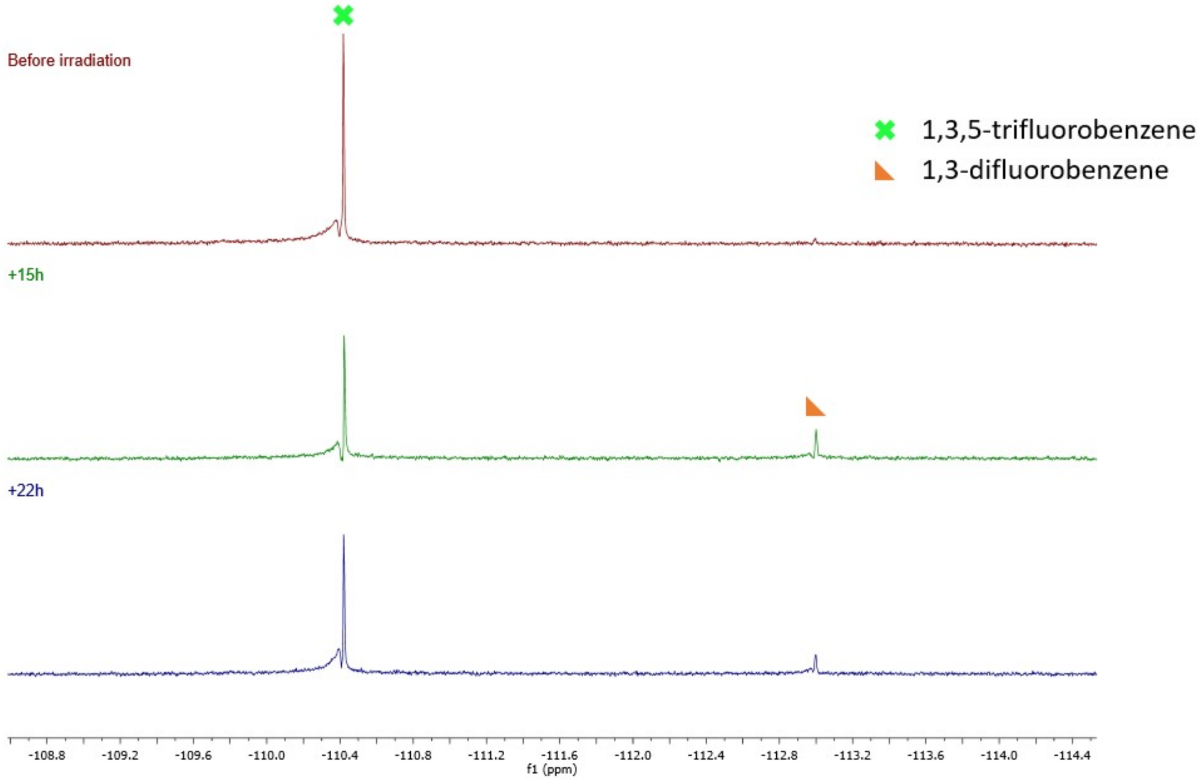

Figure S107. ^{19}F NMR spectrum of the **r**eaction between **1** and 1,3,5-trifluorobenzene under irradiation at 427 nm in THF-d_8

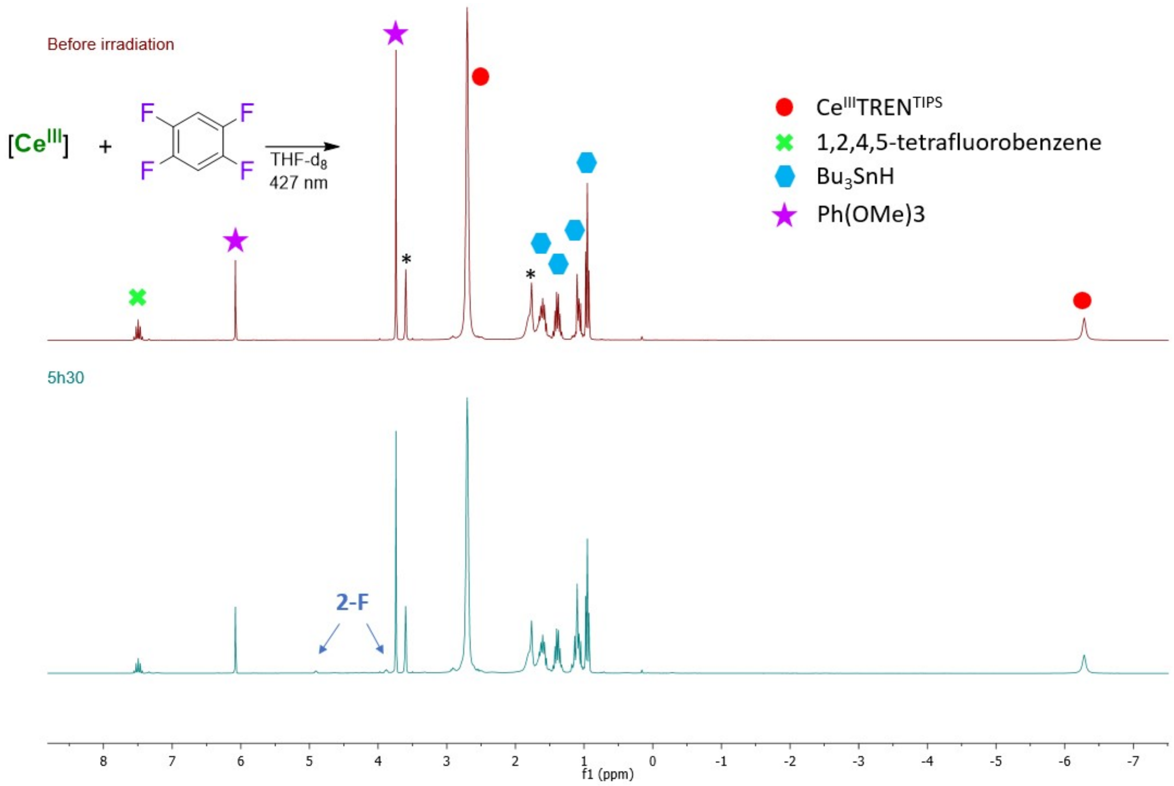

Figure S108. ^{1}H NMR spectrum of the reaction between **1** and 1,2,4,5-tetrafluorobenzene under irradiation at 427 nm in THF-d_8. Solvent residual peaks are indicated with *

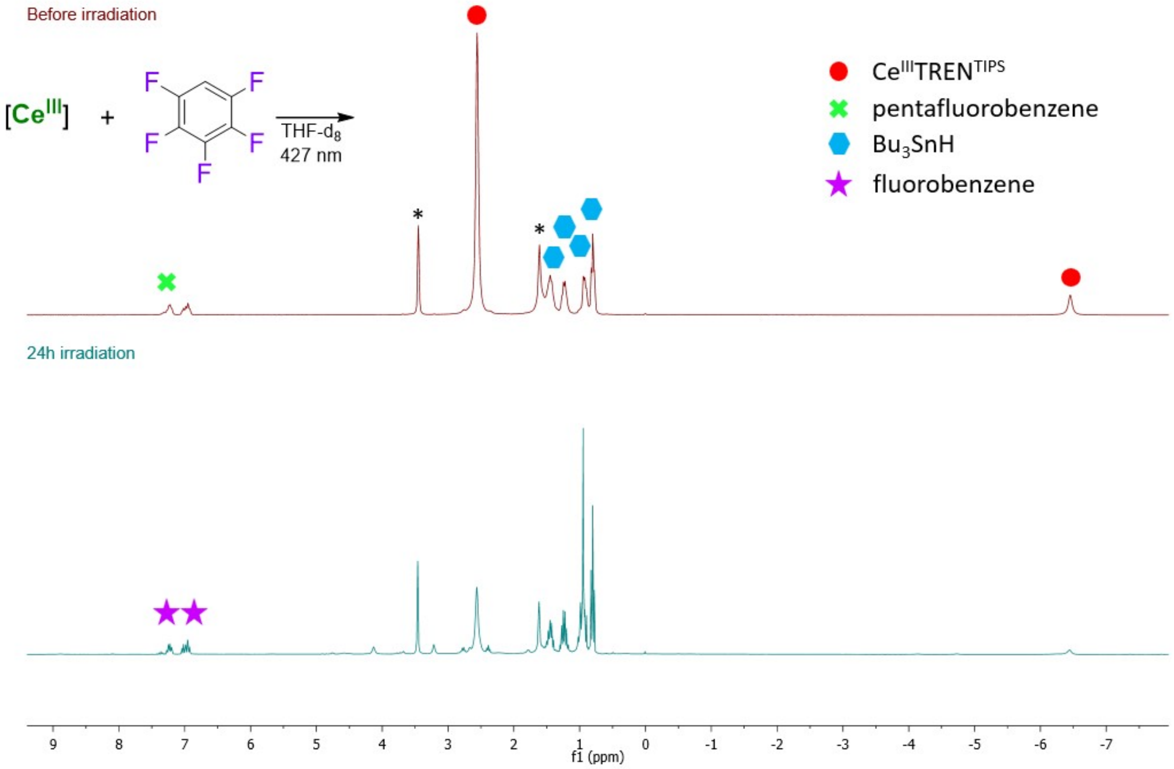

Figure S 109. ^{1}H NMR spectrum of the reaction between **1** and pentafluorobenzene under irradiation at 427 nm in THF-d$_8$. Solvent residual peaks are indicated with *

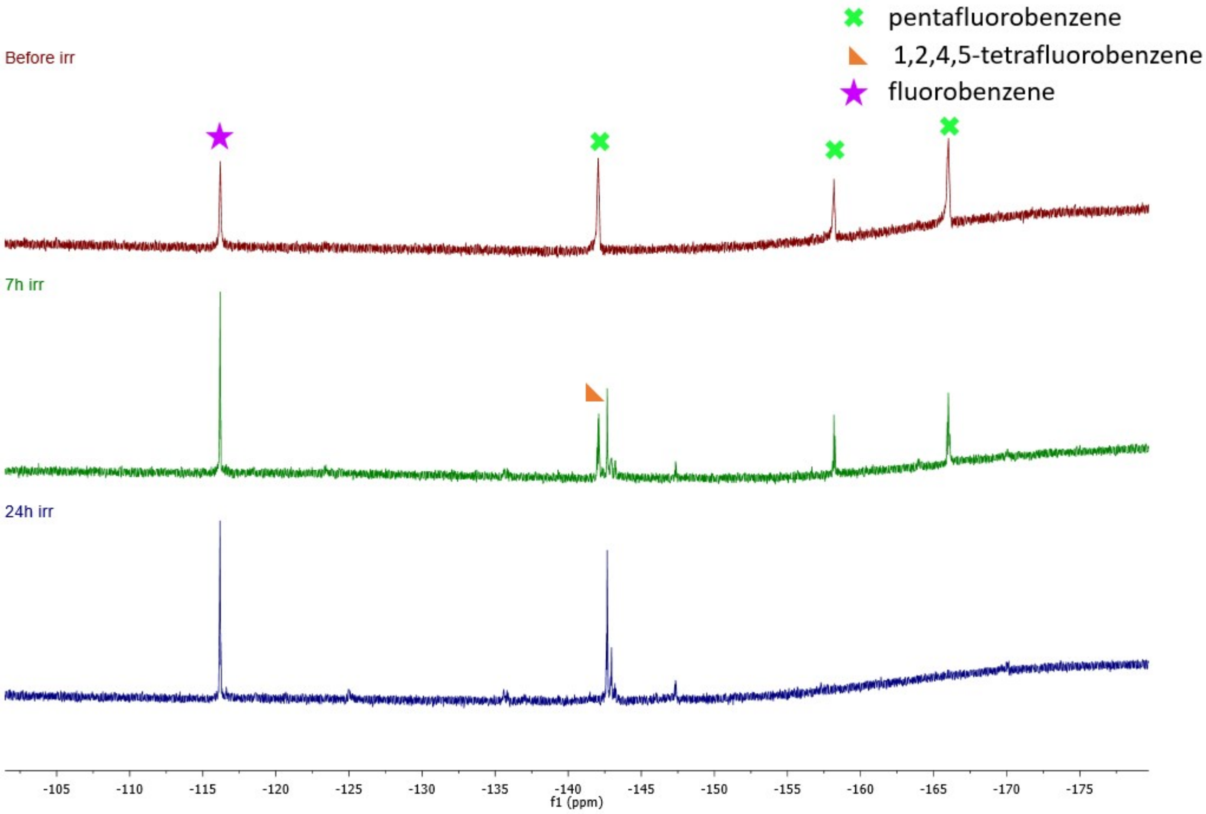

Figure S110. ^{19}F NMR spectrum of the **r**eaction between **1** and pentafluorobenzene under irradiation at 427 nm in THF-d$_8$

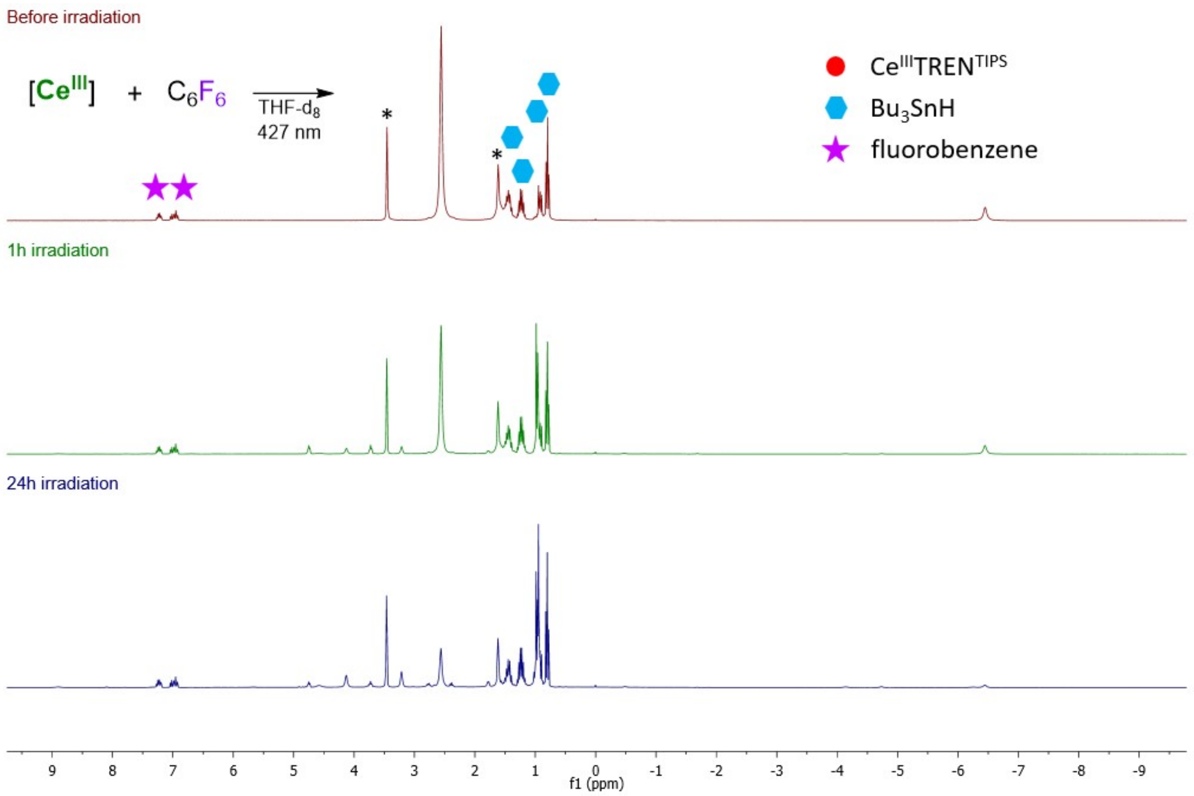

Figure S111. ^{1}H NMR spectrum of the reaction between **1** and hexafluorobenzene under irradiation at 427 nm in THF-d_8. Solvent residual peaks are indicated with *

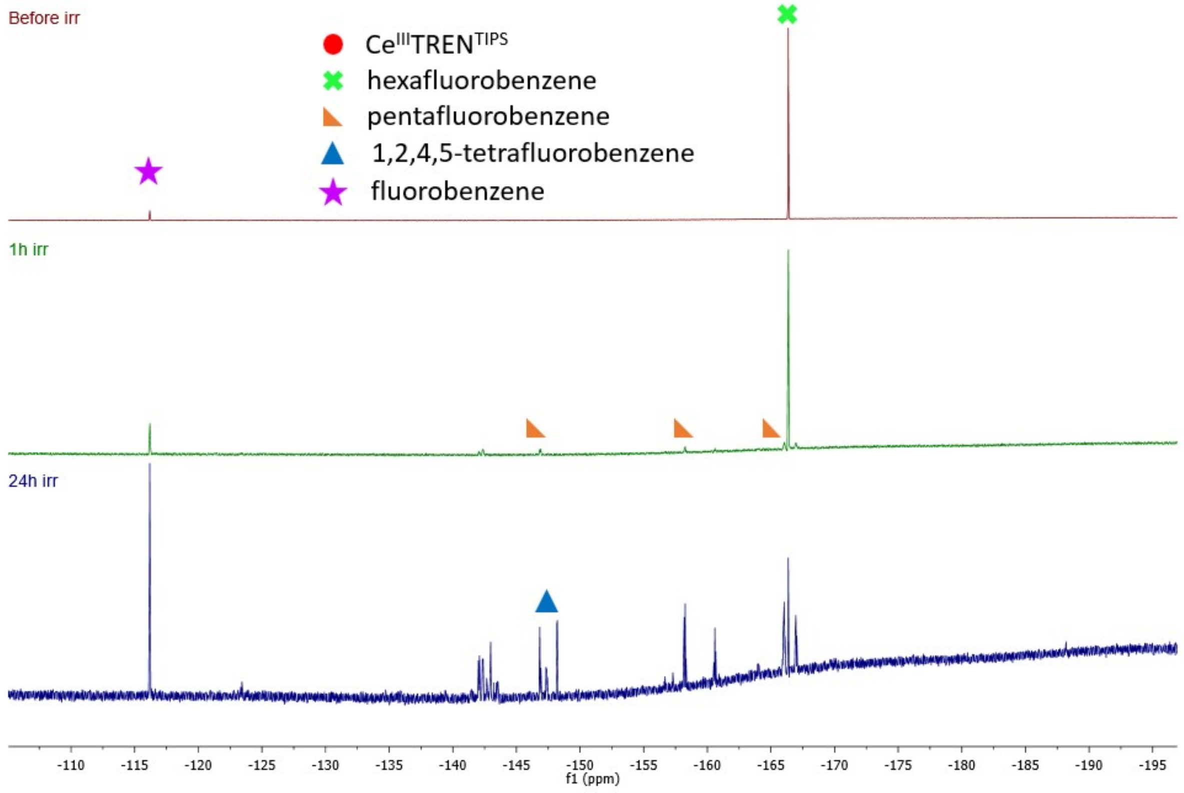

Figure S112. ^{19}F NMR spectrum of the **r**eaction between **1** and hexafluorobenzene under irradiation at 427 nm in THF-d_8

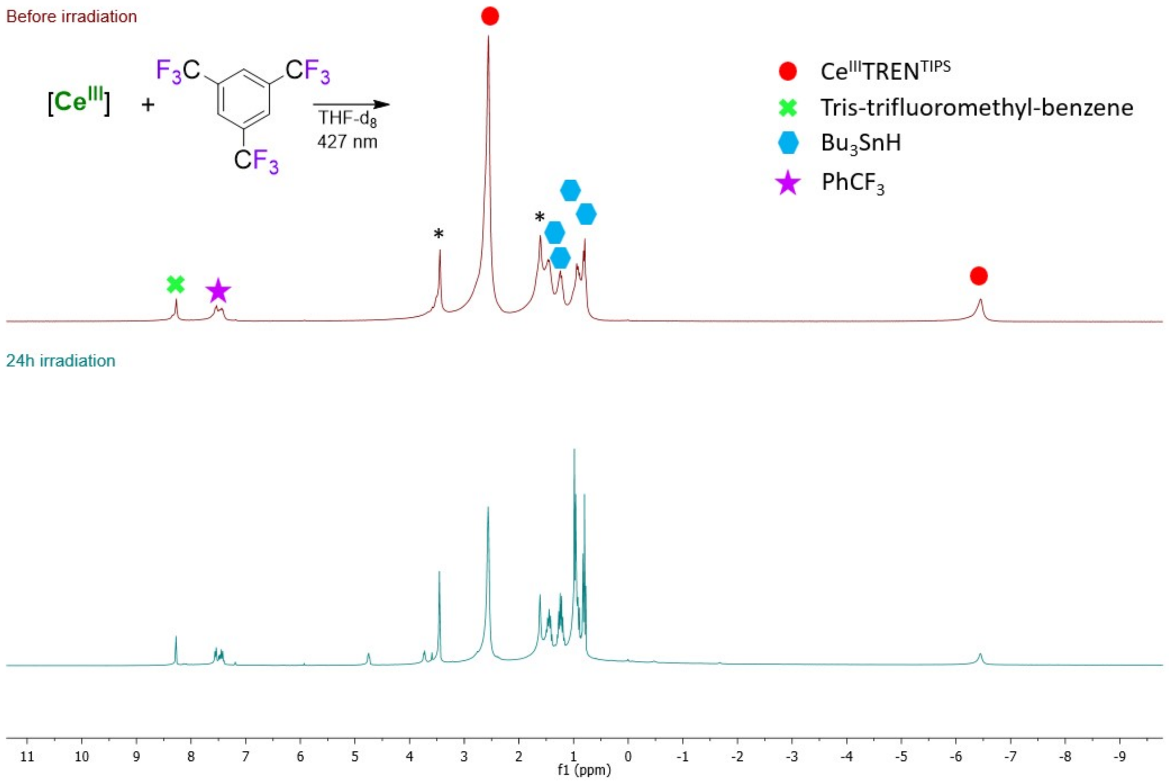

Figure S113. ^{1}H NMR spectrum of the reaction between **1** and tris-trifluoromethyl-benzene under irradiation at 427 nm in THF-d_8. Solvent residual peaks are indicated with *

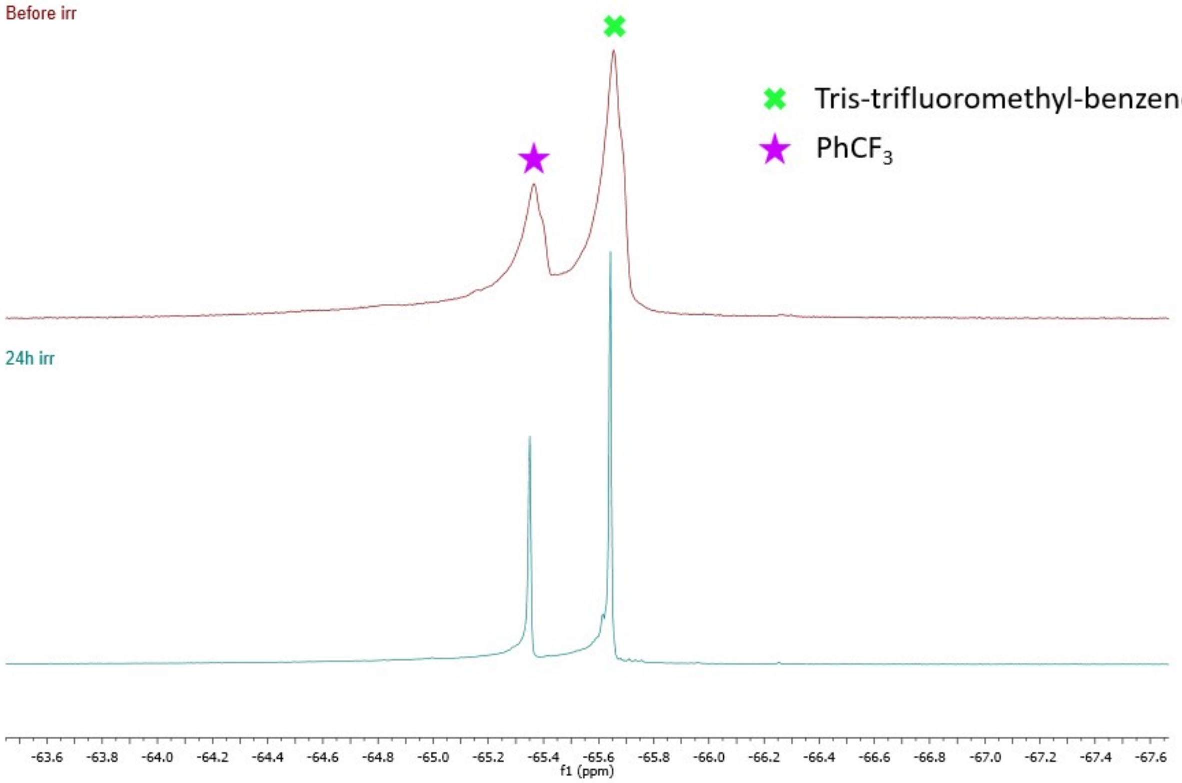

Figure S114. ^{19}F NMR spectrum of the **r**eaction between **1** and tris-trifluoromethyl-benzene under irradiation at 427 nm in THF-d_8

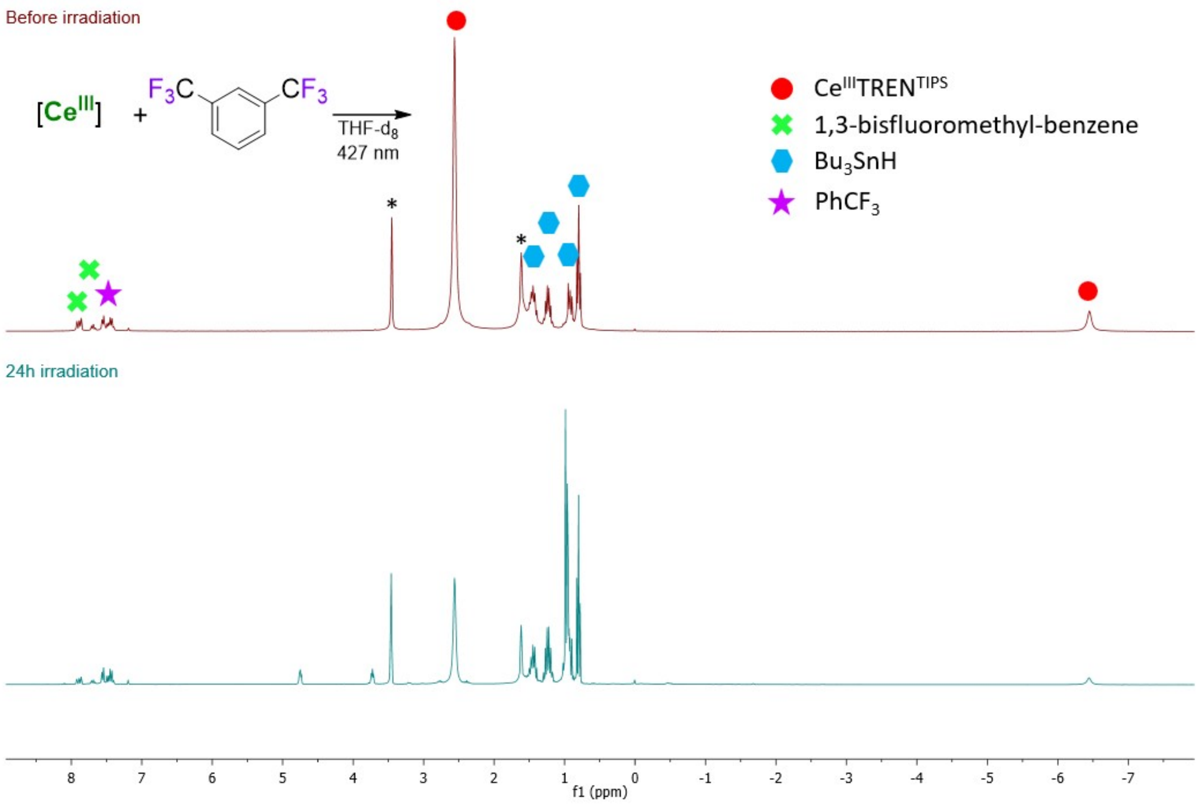

Figure S115. ^{1}H NMR spectrum of the reaction between **1** and 1,3-bis-trifluoromethyl-benzene under irradiation at 427 nm in THF-d_8. Solvent residual peaks are indicated with *

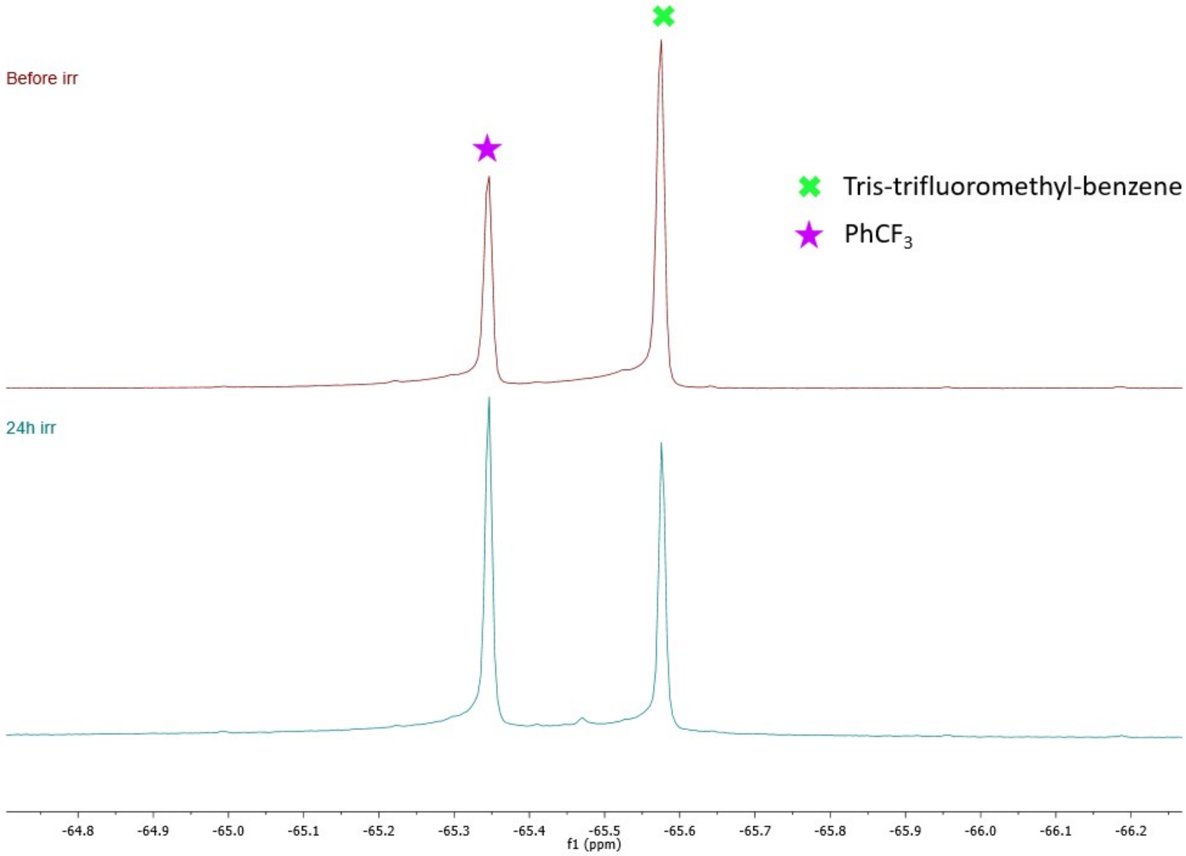

Figure S116. ^{19}F NMR spectrum of the **r**eaction between **1** and 1,3-bis-trifluoromethyl-benzene under irradiation at 427 nm in THF-d_8

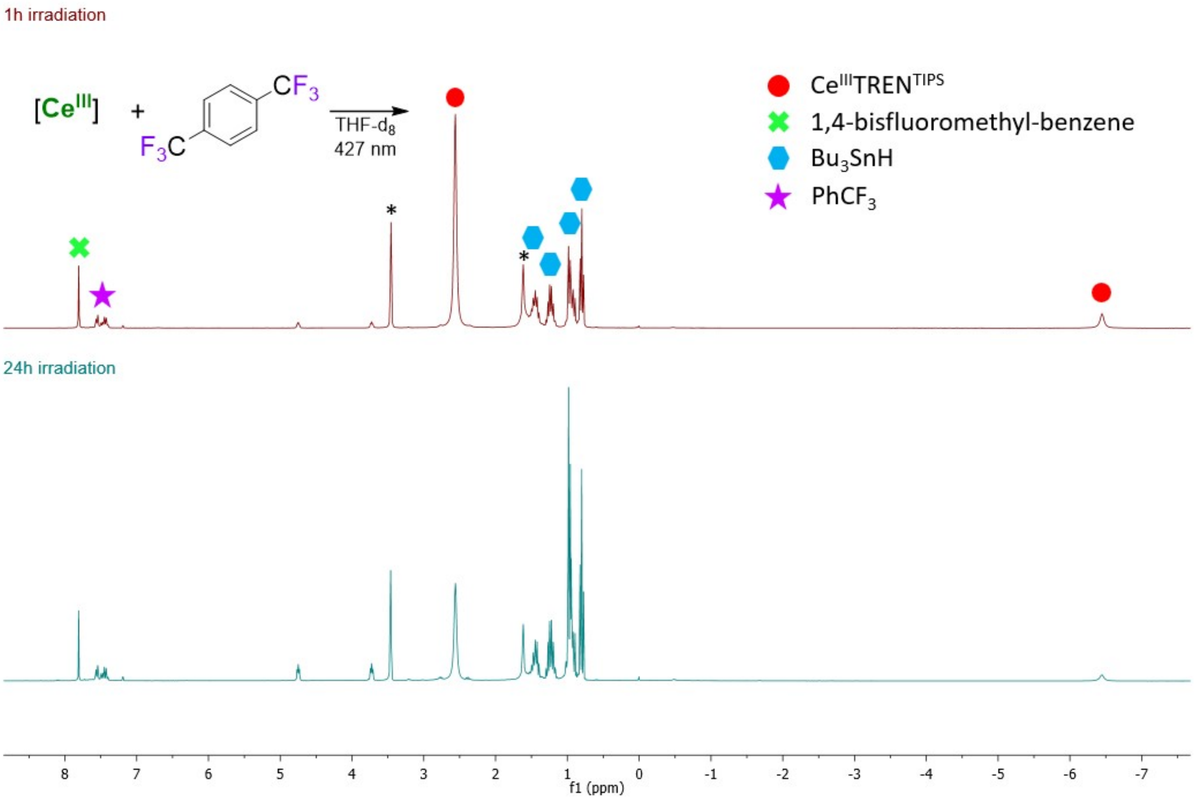

Figure S117. ^{1}H NMR spectrum of the reaction between **1** and 1,4-bis-trifluoromethyl-benzene under irradiation at 427 nm in THF-d_8. Solvent residual peaks are indicated with *

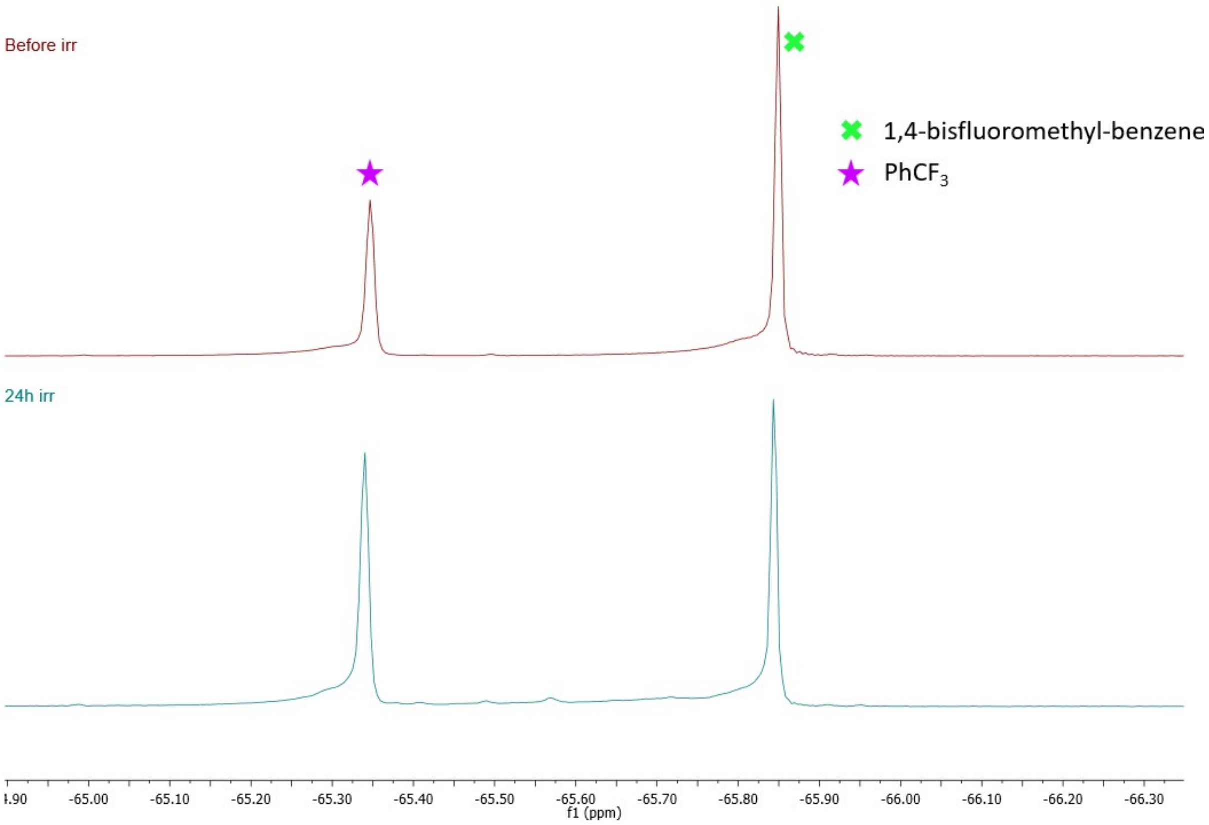

Figure S118. ^{19}F NMR spectrum of the reaction between **1** and 1,4-bis-trifluoromethyl-benzene under irradiation at 427 nm in THF-d_8

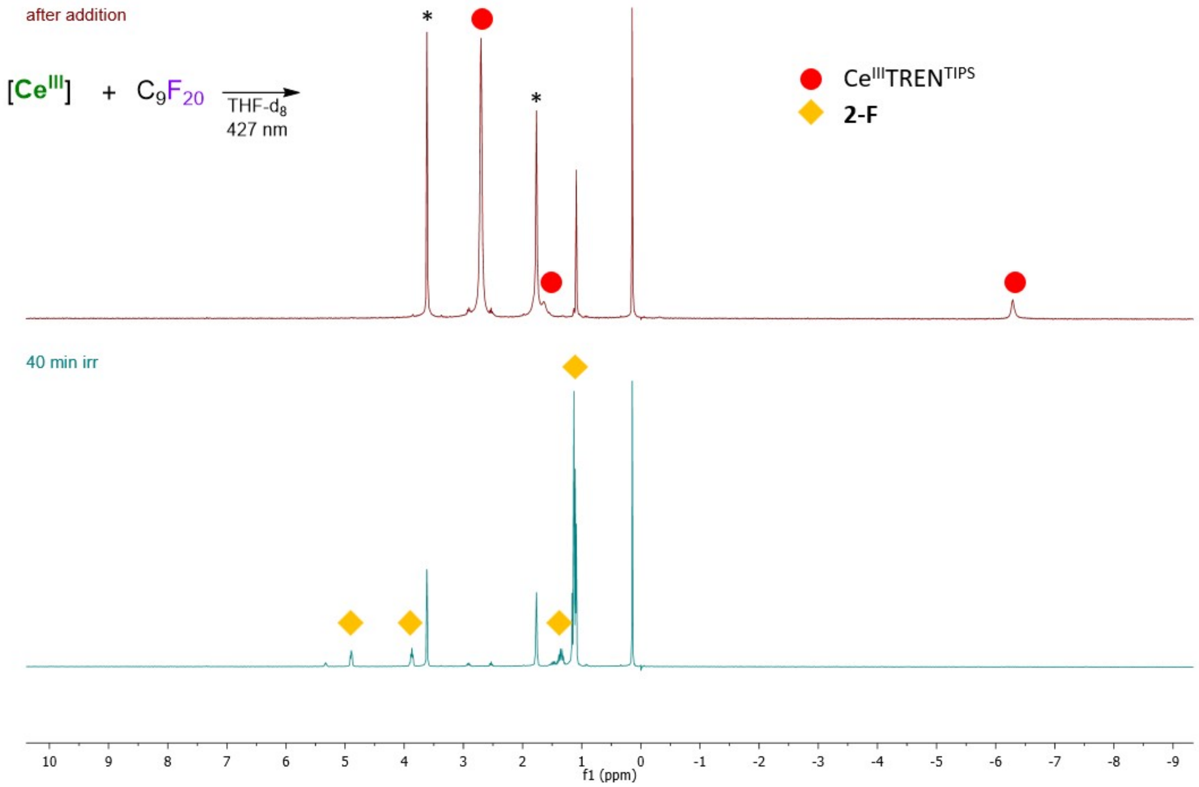

Figure S119. ^{1}H NMR spectrum of the reaction between **1** and perfluorononane under irradiation at 427 nm in THF-d_8. Solvent residual peaks are indicated with *

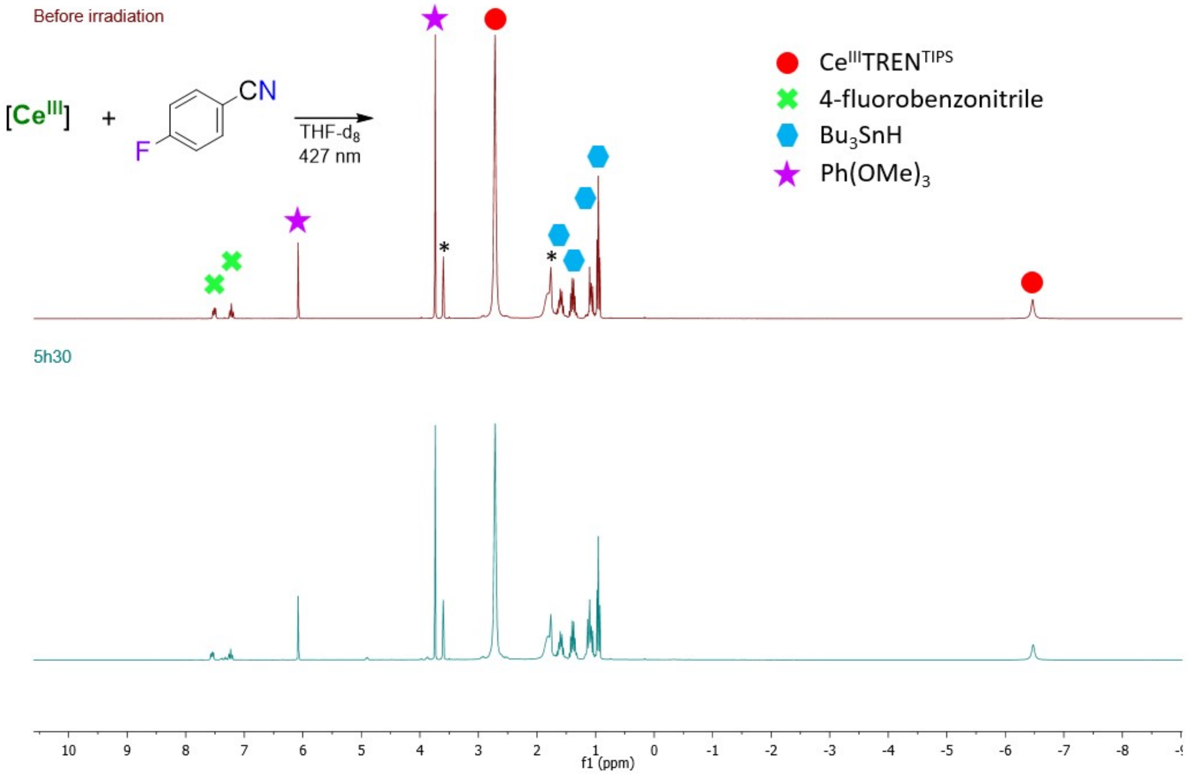

Figure S120. ^{1}H NMR spectrum of the reaction between **1** and 4-fluorobenzonitrile under irradiation at 427 nm in THF-d_8. Solvent residual peaks are indicated with *

3) Photochemical degradation of [FCe^{IV}TREN^{TIPS}] (2-F)

Compound 2-F (5 mg, 6.4 µmol, 1 eq.) was dissolved in 0.4 mL of THF-d8 with 3 drops of C6D6 in a J. Young NMR tube. The tube was irradiated at 427 nm and analysed by 1H NMR at designated times.

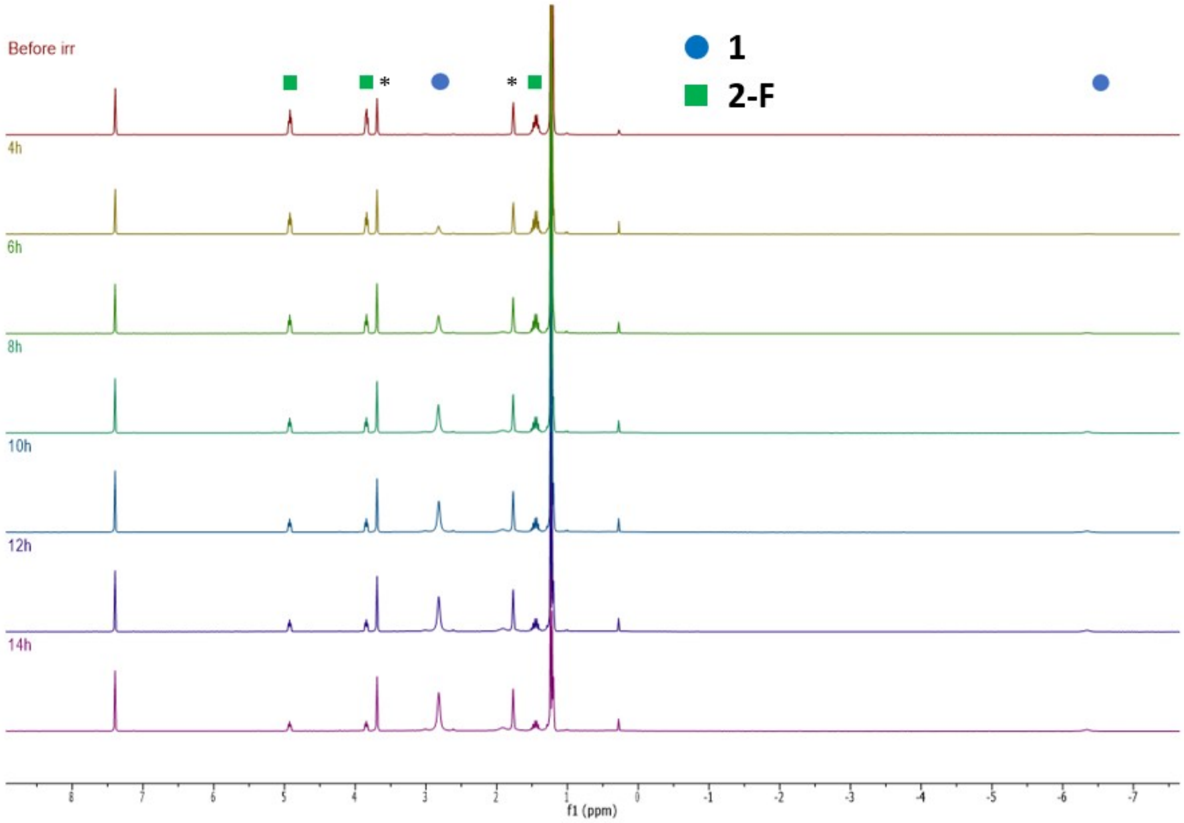

Figure S121. ^{1}H NMR spectra of the photochemical degradation of **2-F** (5 mg, 6.4 µmol) in THF-d_8 (0.4 mL) with 3 drops of C_6D_6. Solvent residual peaks are indicated with *

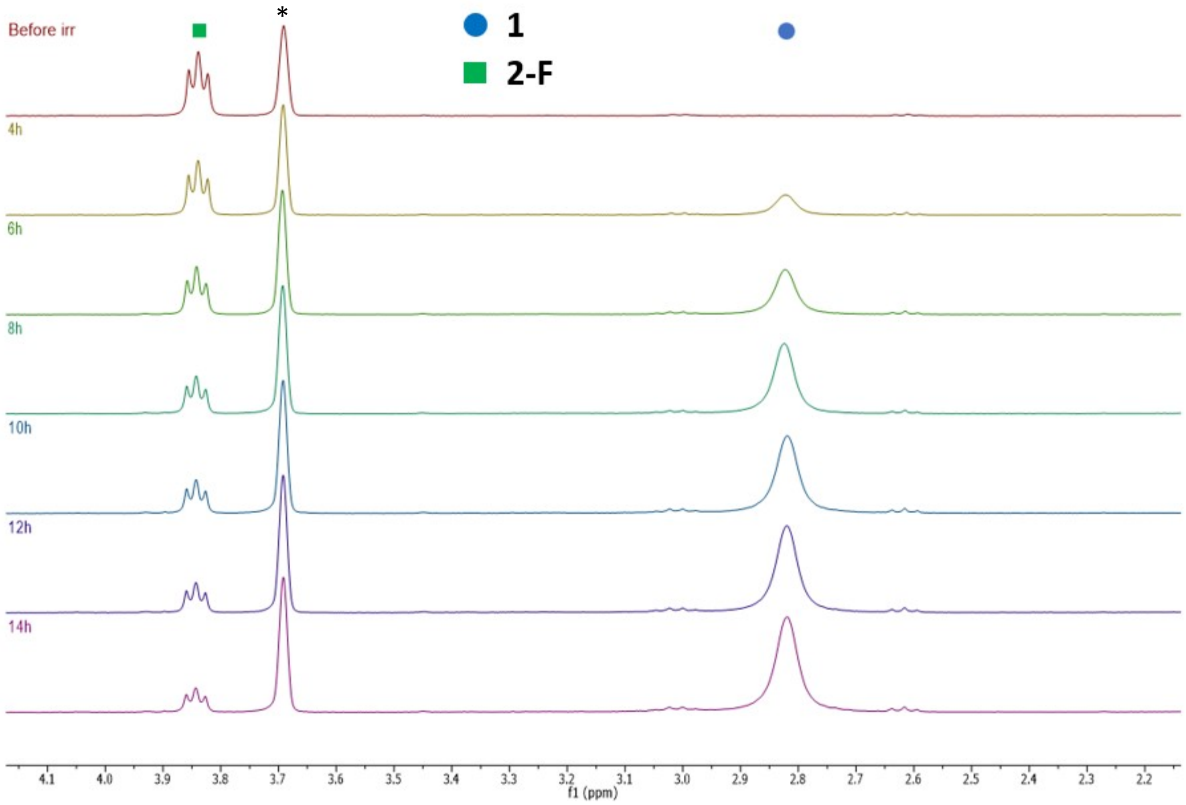

Figure S122. ^{1}H NMR spectra of the photochemical degradation of **2-F** (5 mg, 6.4 µmol) in THF-d_8 (0.4 mL) with 3 drops of C_6D_6. Zoom on the 2.2 ppm and 4.2 ppm region. Solvent residual peak is indicated with *

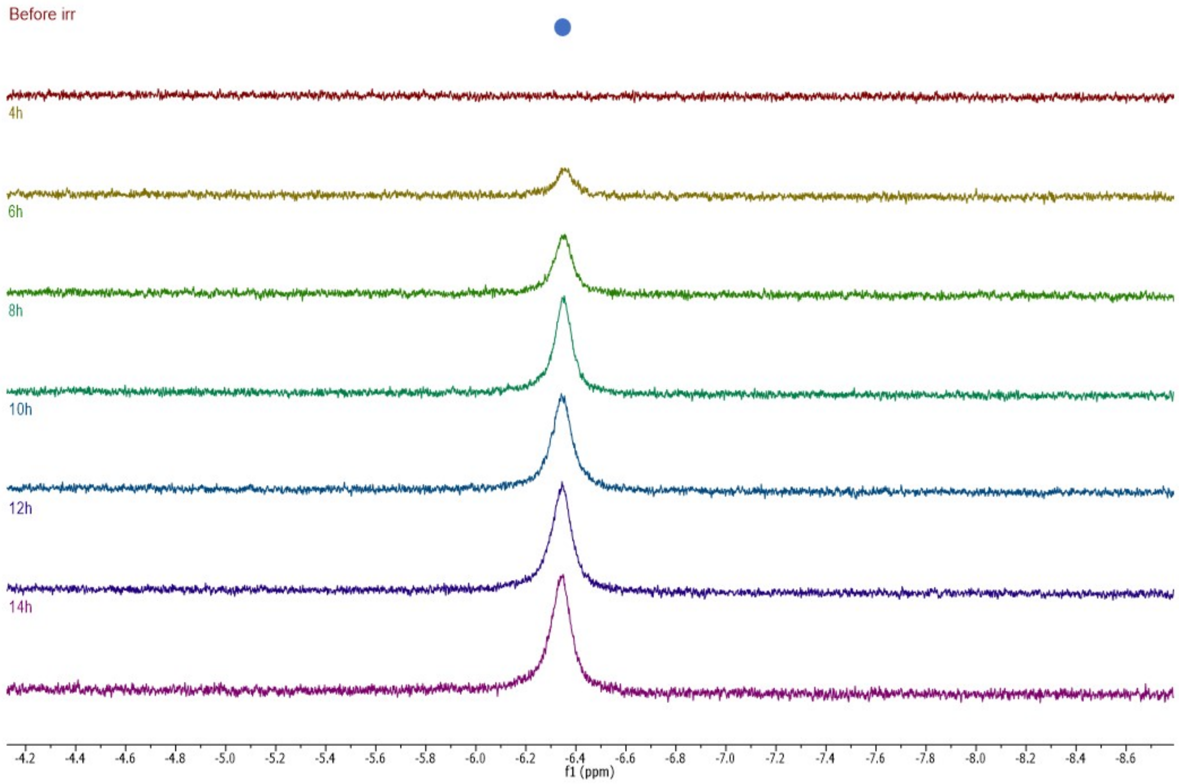

Figure S123. ^{1}H NMR spectra of the photochemical degradation of **2-F** (5 mg, 6.4 µmol) in THF-d_8 (0.4 mL) with 3 drops of C_6D_6. Zoom on the -8.6 ppm and -4.2 ppm region.

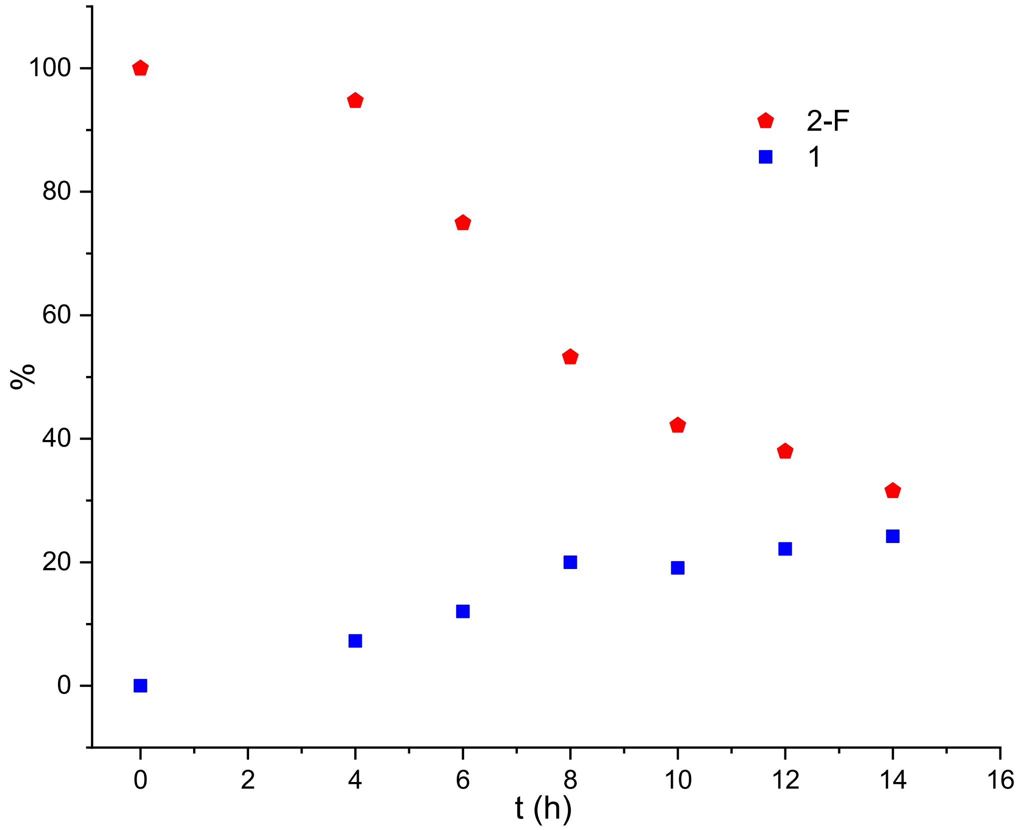

Figure S124. Photochemical degradation of **2-F** (5 mg, 6.4 µmol) with time in THF-d_8 (0.4 mL). NB : between 4 h and 6 h, and 8 and 10 h of irradiation, the NMR tube was stored at 0 °C overnight.

t (min)	% **2-Cl**	% **1**
0	100	0
4	94.73	7.25
6	74.94	12.02
8	53.22	19.99
10	42.17	19.08
12	37.96	22.15
14	31.57	24.21

Table S29. Experimental data of the photochemical degradation of **2-F** (5 mg, 6.3 µmol) in THF-d_8 (0.4 mL)

4) Catalytic experiment on cyclohexyl chloride

Cyclohexyl chloride (30 µL, 0.25 mmol), tris(trimethylsilyl)silane (77 µL, 0.25 mmol), 1,3,5-trimethoxybenzene (42 mg, 0.25 mmol), and **1** (9.3 mg, 0.0125 mmol) were dissolved in THF-d_8 (0.5 mL). The resulting solution was introduced in a J. Young NMR tube. The tube was irradiated at 427 nm for 16 h and analyzed by ^{1}H NMR.

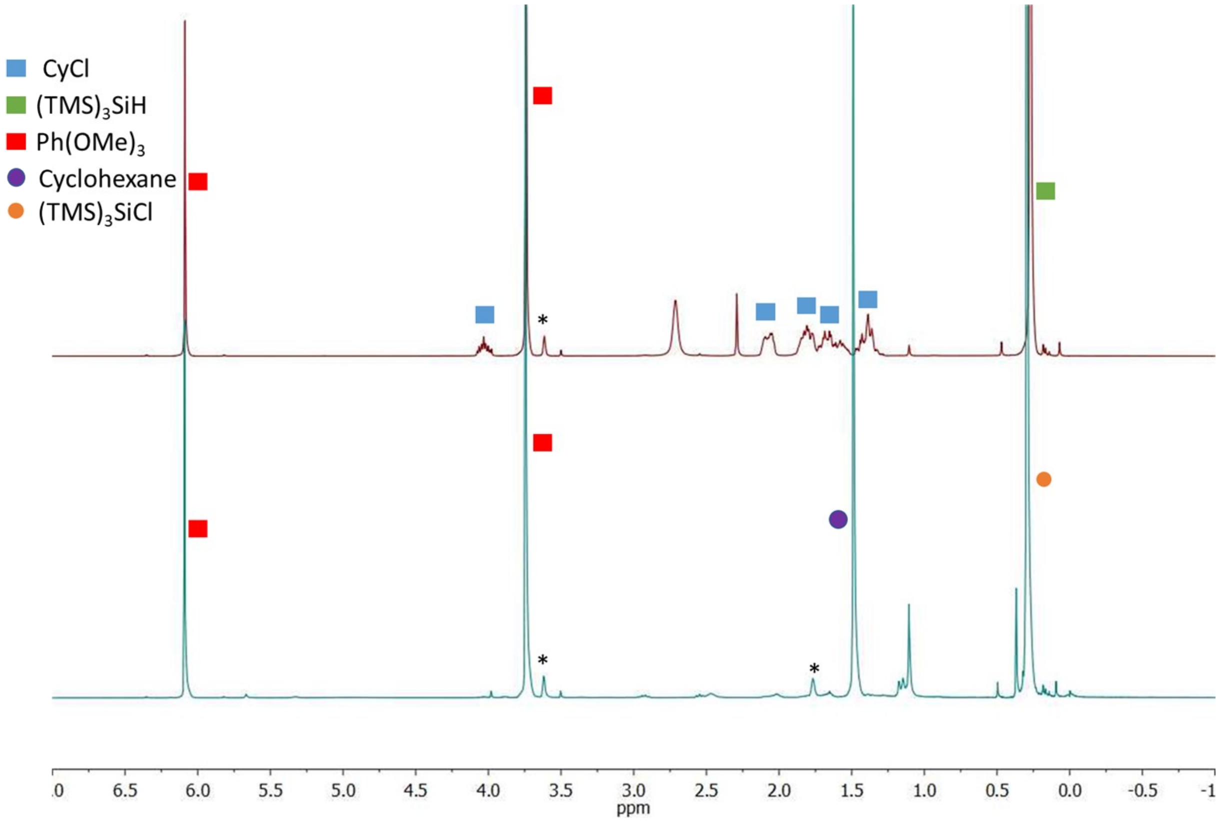

Figure S125. Stack of ^{1}H NMR spectra of the photochemical catalytic proto-dechlorination of cyclohexyl chloride (0.25 mmol) using $(TMS)_3SiH$ (0.25 mmol) and **1** (5 mol%) in THF-d_8 (0.5 mL) under 427 nm irradiation during 16h. 1,3,5-trimethoxybenzene (0.25 mmol) was used as internal standard. Solvent residual peaks are indicated with * and the peaks corresponding to **1** were omitted.

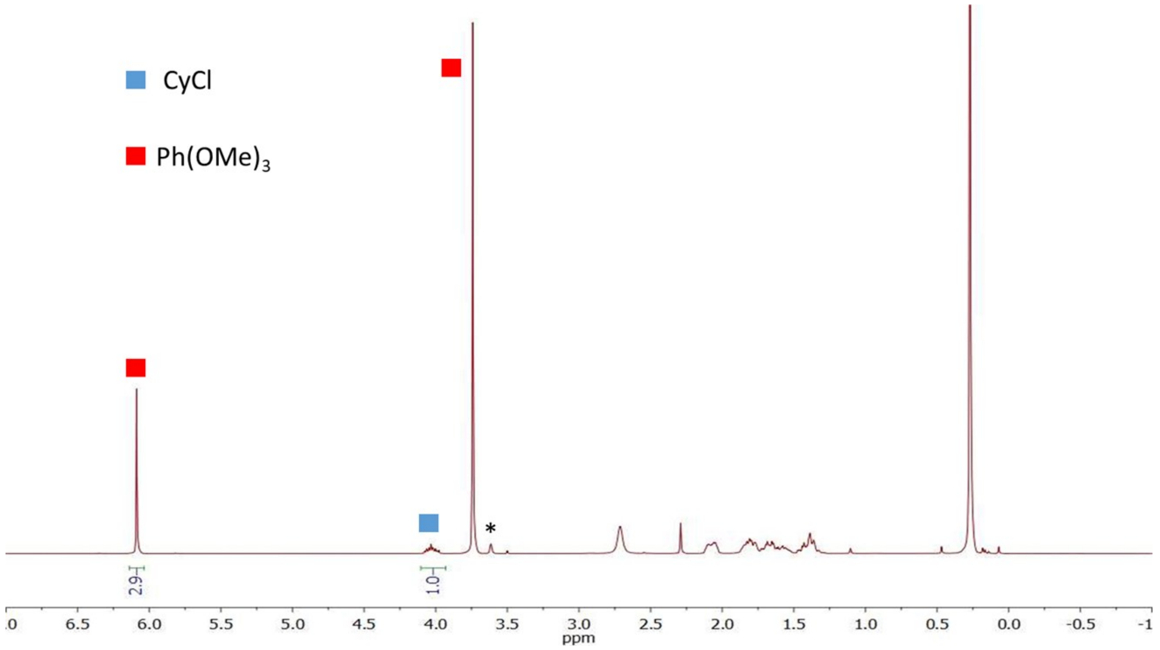

Figure S126. ^{1}H NMR spectra before irradiation of the photochemical catalytic proto-dechlorination of cyclohexyl chloride (0.25 mmol) using $(TMS)_3SiH$ (0.25 mmol) and **1** (5 mol%) in THF-d_8 (0.5 mL). 1,3,5-trimethoxybenzene (0.25 mmol) was used as internal standard. Solvent residual peaks are indicated with * and the peaks corresponding to **1** were omitted.

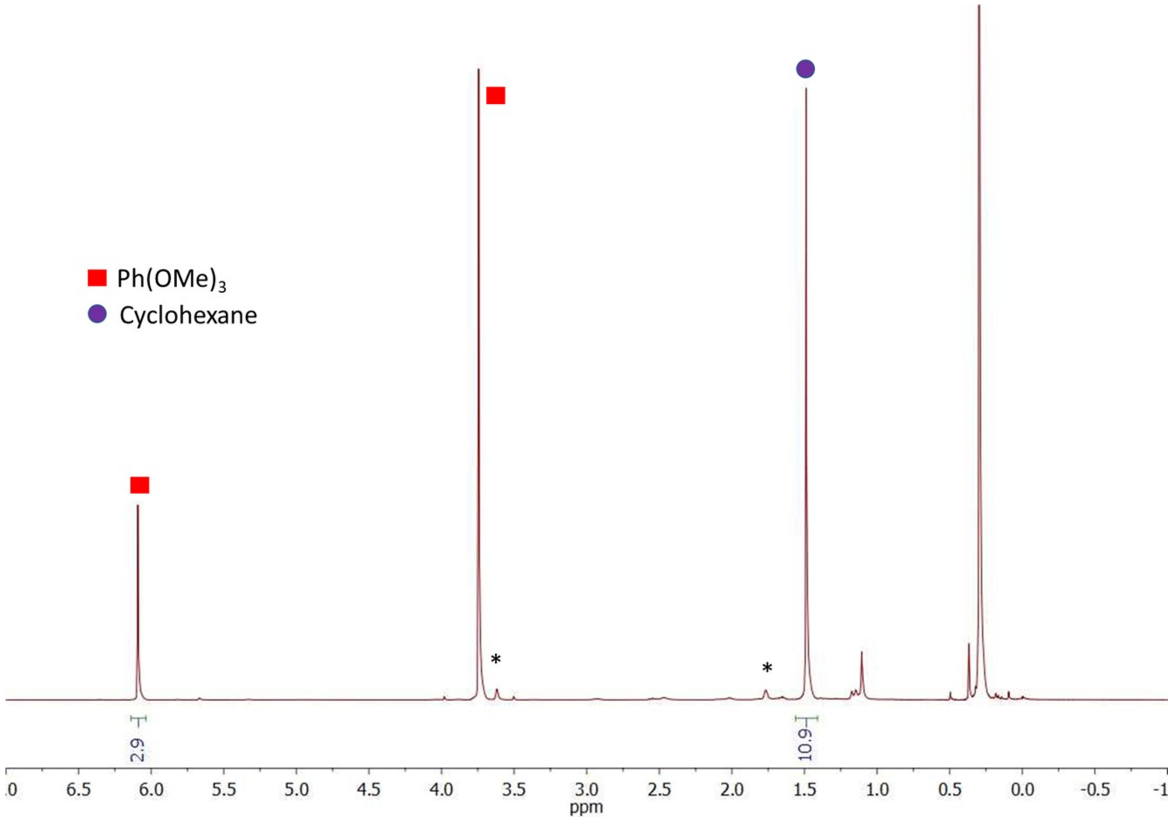

Figure S125. ^{1}H NMR spectra of the photochemical catalytic proto-dechlorination of cyclohexyl chloride (0.25 mmol) using $(TMS)_3SiH$ (0.25 mmol) and **1** (5 mol%) in THF-d_8 (0.5 mL) under 427 nm irradiation during 16h. 1,3,5-trimethoxybenzene (0.25 mmol) was used as internal standard. Solvent residual peaks are indicated with * and the peaks corresponding to **1** were omitted.

IX. Computational details

All DFT and TDDFT calculations were done using Gaussian 09 software package.[10] Optimizations, frequency calculations, and population analyses were performed using the B3PW91 functional.[11,12] Ce, Si, Cl, and Br atoms were treated with a Stuttgart effective core potential and the associated basis set.[13,14] In the case of Cl and Br, a set of polarization functions was added.[15] The 6-31G** basis set was employed for C, N, and H atoms.[16,17]

[CeTREN^{TIPS}] (1)

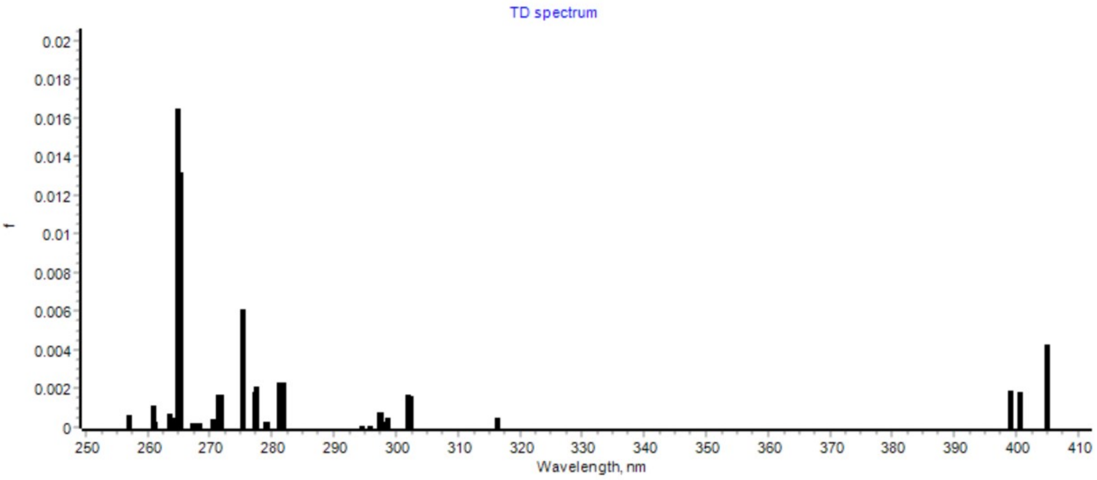

Excited State 7: 2.007-?Sym 3.0627 eV 404.82 nm f=0.0043 <S**2>=0.757

172A -> 173A 0.76992
172A -> 174A 0.50679
172A -> 178A -0.14706
172A -> 182A -0.2671
172A -> 190A 0.10370

	**MO 172A**
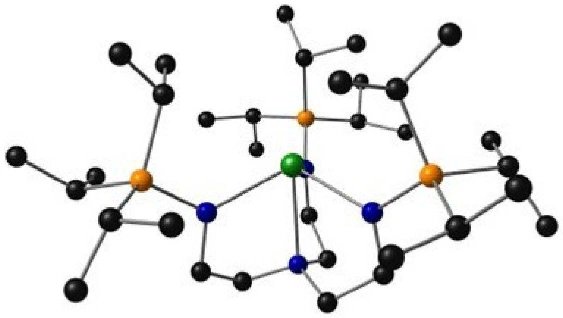	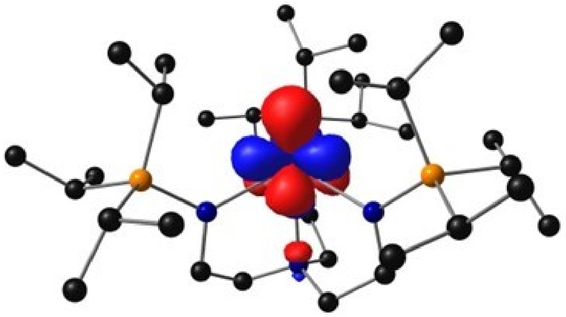
**MO173A**	**MO 174A**
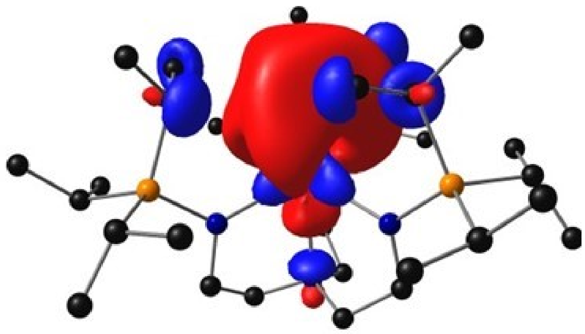	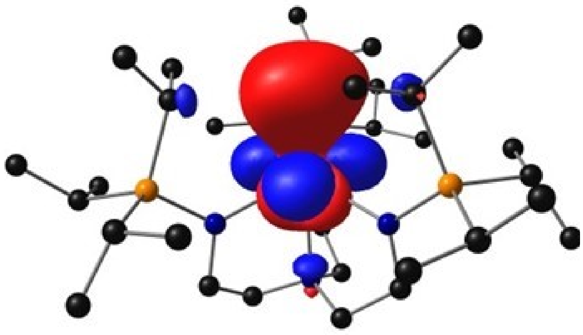

[CeTREN^{TIPS}] (1)-ClC$_6$H$_5$

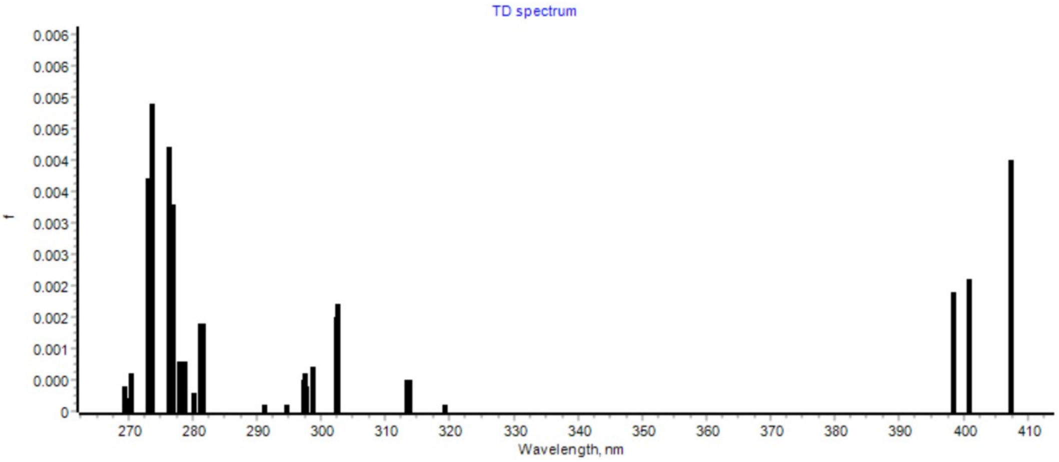

Excited State 7: 2.007-?Sym 3.0447 eV 407.22 nm f=0.0040 <S**2>=0.757

196A -> 198A -0.10157

196A -> 199A 0.78502

196A -> 201A -0.16134

196A -> 203A 0.35567

196A -> 205A 0.24500

196A -> 209A -0.11784

196A -> 210A 0.18153

196A -> 212A -0.24133

	MO 196A
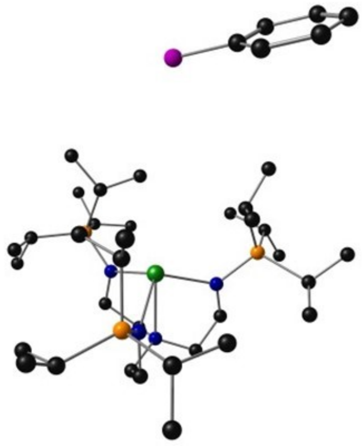	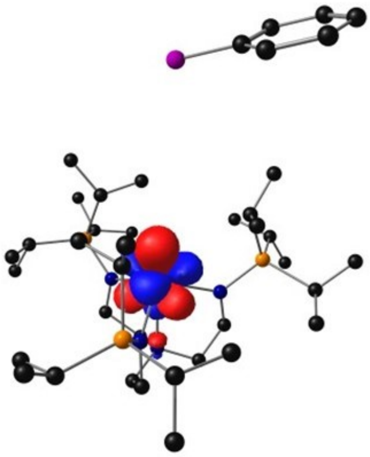
MO 199A	MO 203A
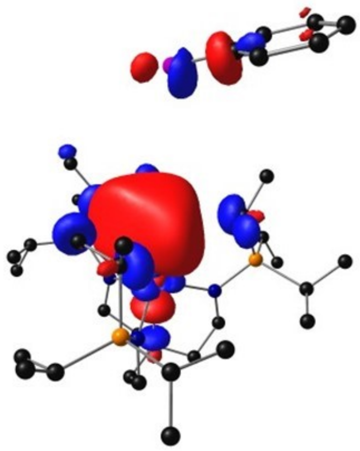	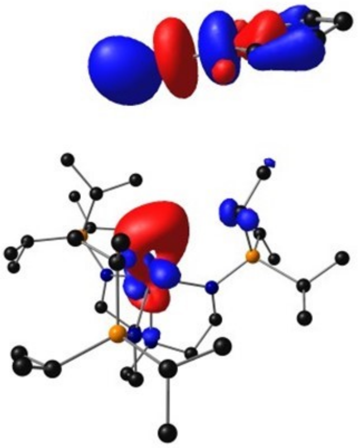

[ClCeTREN^{TIPS}] (2-Cl)

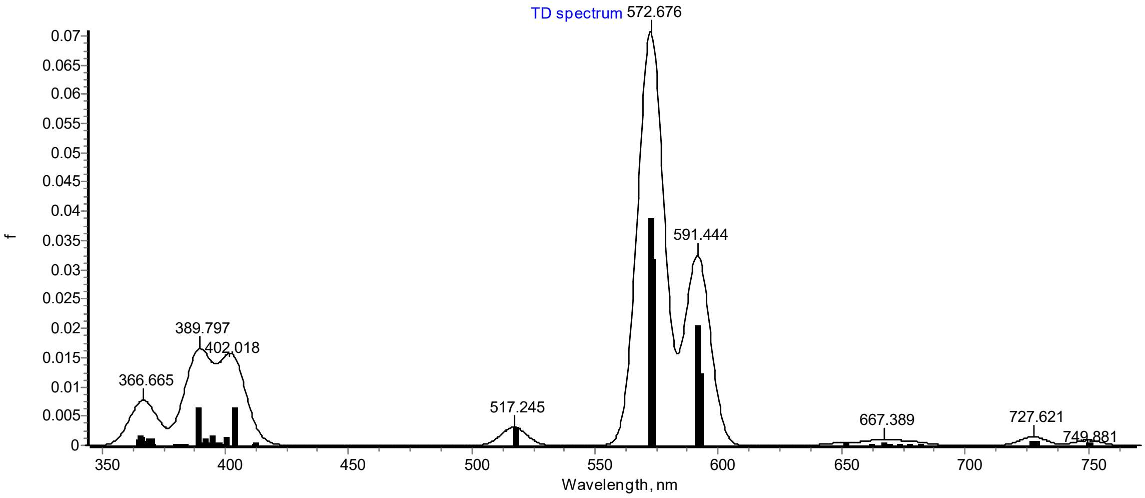


```
Excited State  17:      Singlet-?Sym    2.0934 eV  592.25 nm  f=0.0122  <S**2>=0.000
    174 -> 181         0.34377
    174 -> 182         0.15040
    175 -> 181        -0.28534
    175 -> 182         0.49810

Excited State  18:      Singlet-?Sym    2.0975 eV  591.10 nm  f=0.0204  <S**2>=0.000
    174 -> 181         0.21142
    174 -> 182        -0.34753
    175 -> 181         0.47563
    175 -> 182         0.25002

Excited State  19:      Singlet-?Sym    2.1643 eV  572.87 nm  f=0.0319  <S**2>=0.000
    174 -> 181        -0.26025
    174 -> 182         0.49170
    175 -> 181         0.32073
    175 -> 182         0.24058

Excited State  20:      Singlet-?Sym    2.1665 eV  572.27 nm  f=0.0389  <S**2>=0.000
    174 -> 181         0.46982
    174 -> 182         0.31367
    175 -> 181         0.16837
    175 -> 182        -0.34510
```

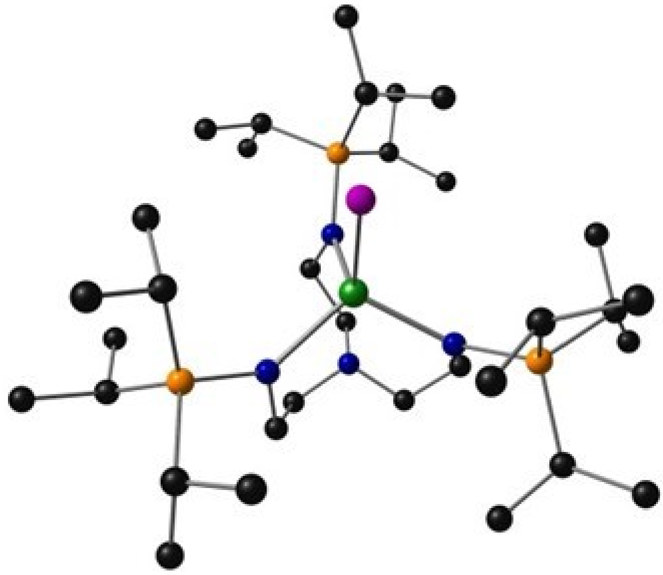

MO 174	MO 175
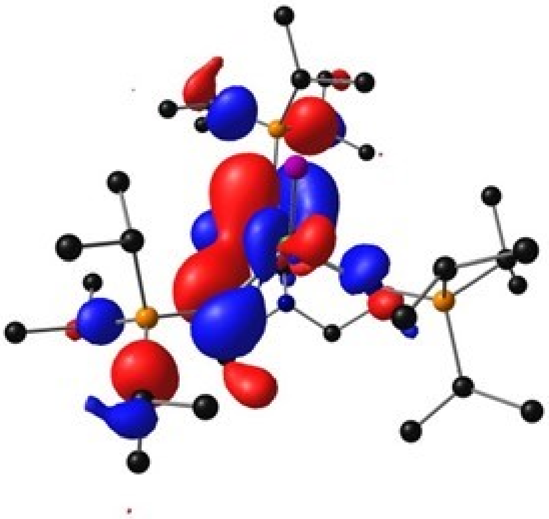	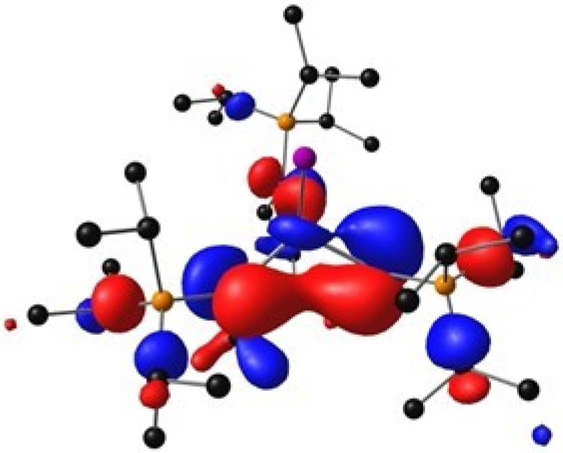

MO 181	MO 182
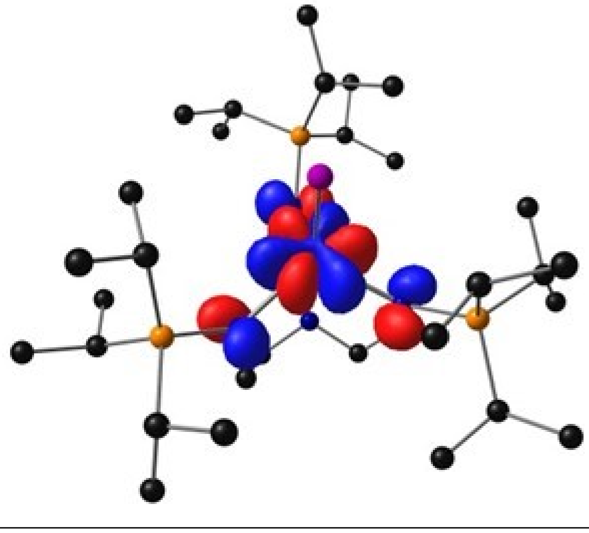	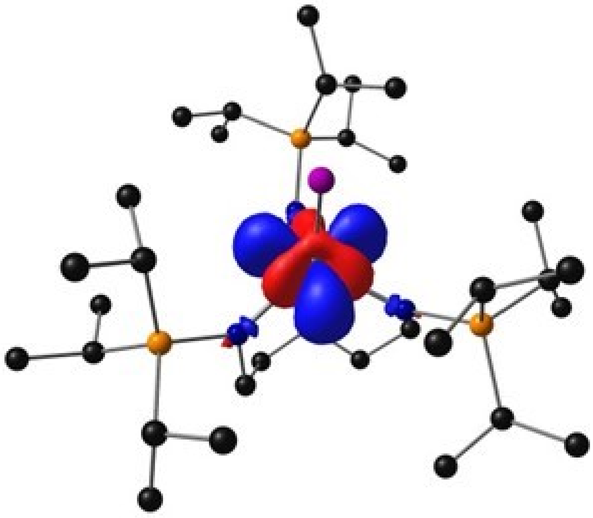

[BrCeTREN^{TIPS}] (2-Br)

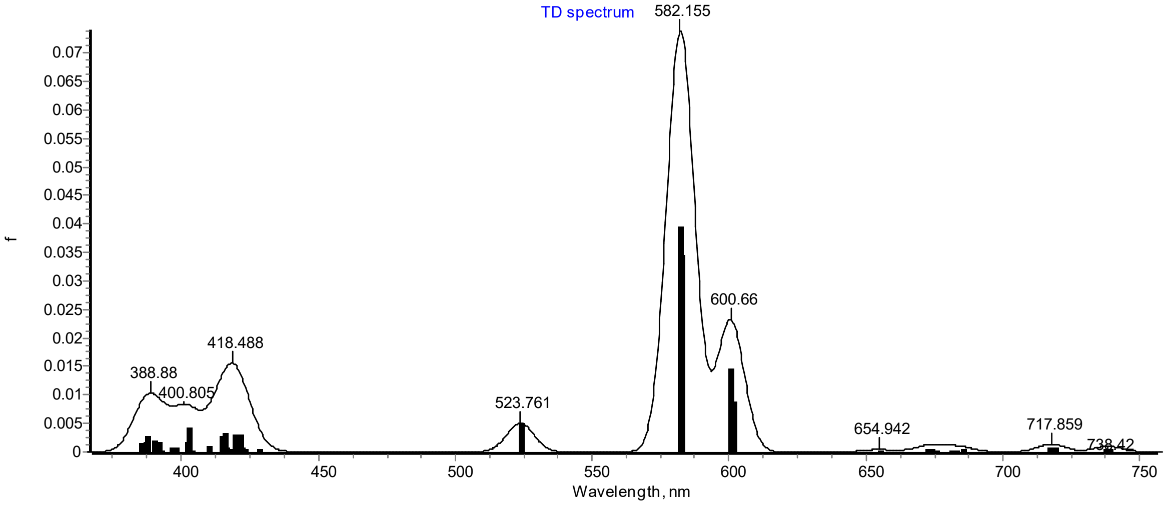


```
Excited State  17:      Singlet-?Sym    2.0621 eV  601.25 nm  f=0.0087  <S**2>=0.000
     174 -> 181        -0.39543
     175 -> 181        -0.10767
     175 -> 182         0.53911

Excited State  18:      Singlet-?Sym    2.0660 eV  600.12 nm  f=0.0146  <S**2>=0.000
     174 -> 181        -0.10287
     174 -> 182         0.46797
     175 -> 181         0.46304

Excited State  19:      Singlet-?Sym    2.1276 eV  582.73 nm  f=0.0346  <S**2>=0.000
     174 -> 181        -0.10355
     174 -> 182         0.48688
     175 -> 181        -0.44181
     175 -> 182        -0.12159

Excited State  20:      Singlet-?Sym    2.1308 eV  581.87 nm  f=0.0395  <S**2>=0.000
     174 -> 181         0.50245
     174 -> 182         0.13397
     175 -> 182         0.41798
```

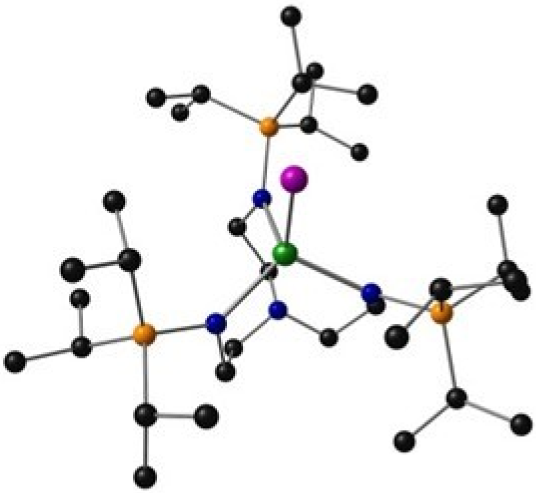

MO 174	MO 175
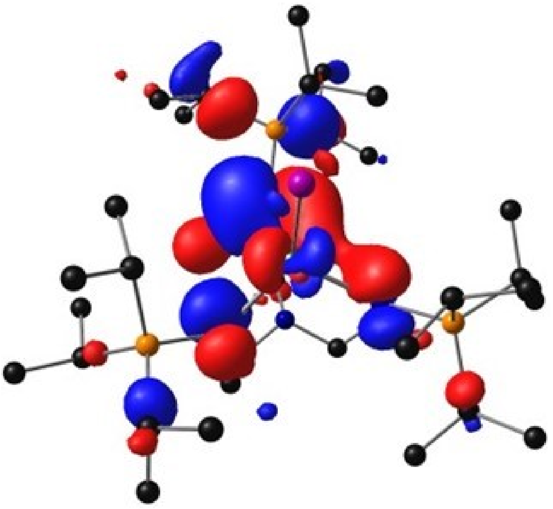	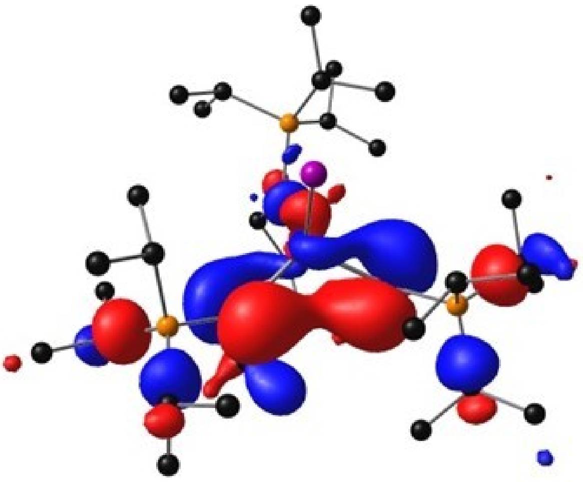
**MO 181**	**MO 182**
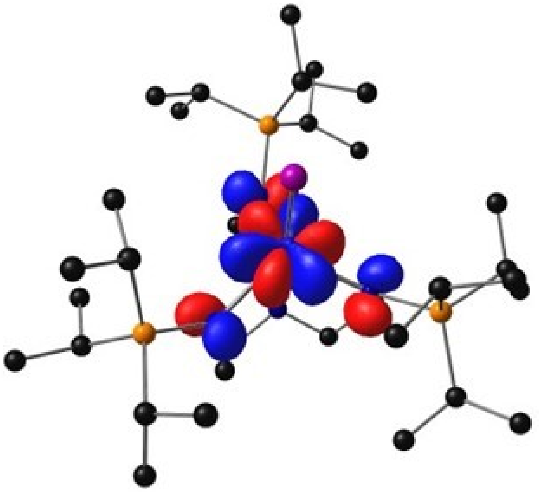	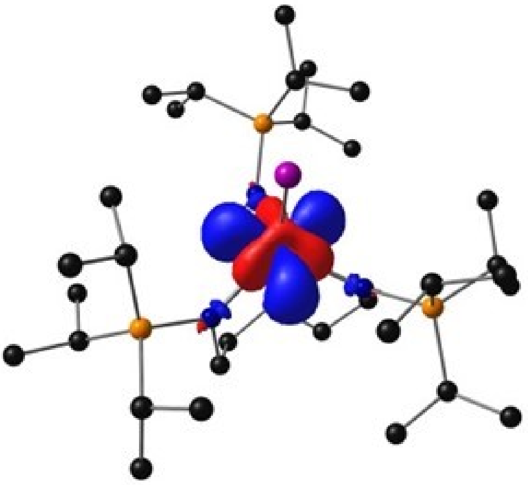

[FCeTREN^{TIPS}] (2-F)

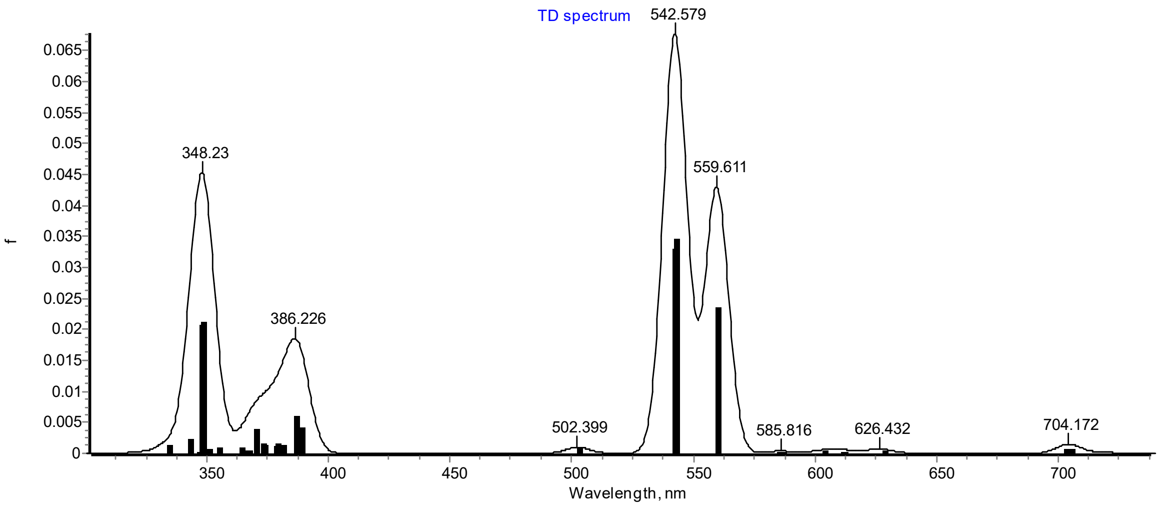


```
Excited State  17:      Singlet-?Sym    2.2149 eV  559.78 nm  f=0.0236  <S**2>=0.000
    174 -> 183        0.39720
    175 -> 181        0.54022
    175 -> 182       -0.11873

Excited State  18:      Singlet-?Sym    2.2166 eV  559.35 nm  f=0.0193  <S**2>=0.000
    174 -> 181        0.51485
    174 -> 182       -0.11277
    175 -> 183       -0.44242

Excited State  19:      Singlet-?Sym    2.2853 eV  542.52 nm  f=0.0346  <S**2>=0.000
    174 -> 181        0.34214
    174 -> 183       -0.27712
    175 -> 181        0.23657
    175 -> 183        0.47339

Excited State  20:      Singlet-?Sym    2.2864 eV  542.28 nm  f=0.0330  <S**2>=0.000
    174 -> 181        0.25998
    174 -> 183        0.49804
    175 -> 181       -0.29974
    175 -> 183        0.25805
```

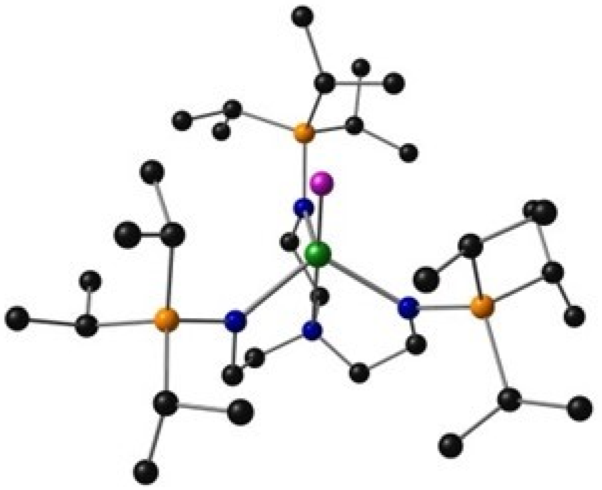

MO 174	MO 175
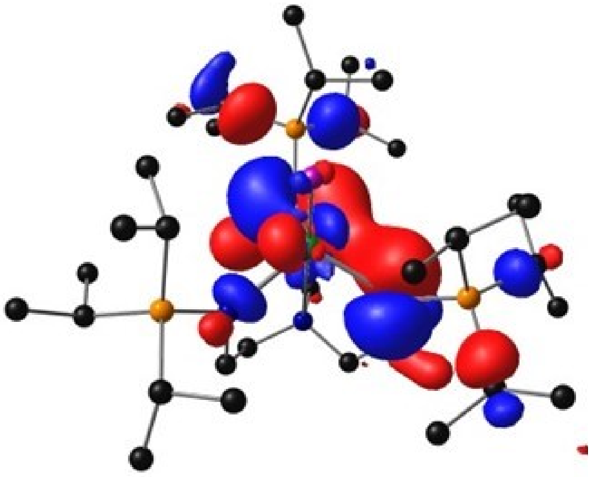	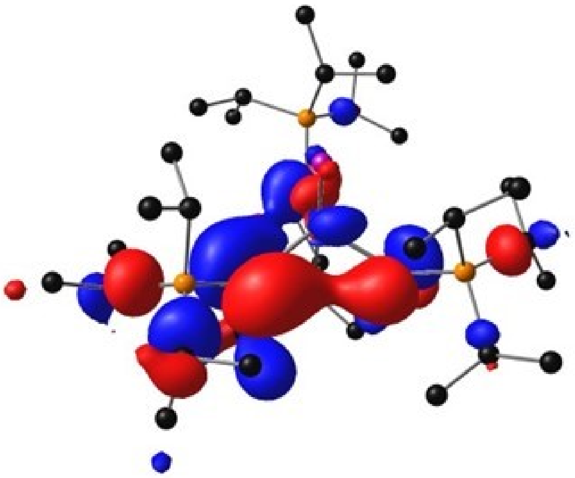
**MO 181**	**MO 183**
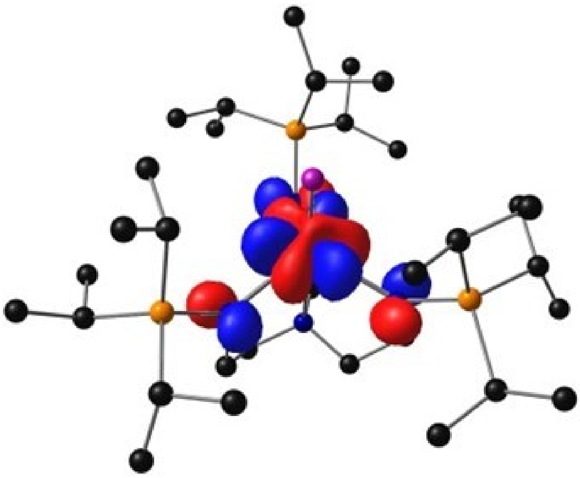	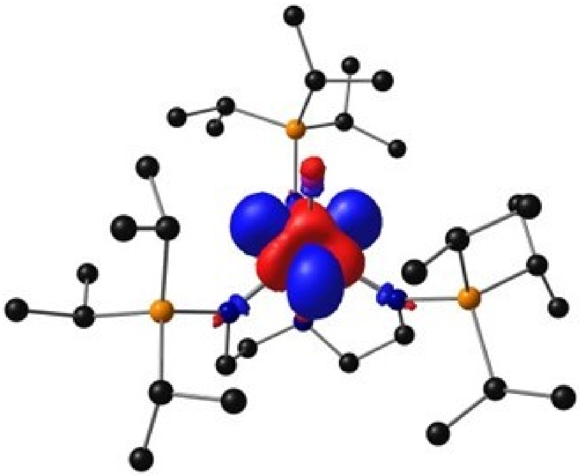

Cartesian coordinates of all optimized structures

[CeTREN^{TIPS}] (**1**)

Ce	6.298151000	6.498986000	8.354767000
Si	6.547390000	2.870214000	8.404666000
Si	6.537919000	8.264814000	5.179715000
Si	6.606317000	8.372443000	11.447273000
N	3.731161000	6.509074000	8.379889000
N	5.507459000	4.310824000	8.455291000
N	5.533458000	7.681203000	10.211032000
N	5.501024000	7.514773000	6.413614000
C	8.370044000	3.568385000	8.482029000
H	8.363206000	4.440047000	7.801016000
C	6.377884000	10.205385000	5.093104000
H	7.225822000	10.546988000	4.480557000
C	6.114314000	7.602057000	3.397520000
H	5.132674000	8.049977000	3.180743000
C	8.409794000	8.094639000	10.748676000
H	8.394871000	7.072418000	10.326052000
C	6.394647000	1.837161000	6.759500000
H	7.245238000	1.139314000	6.771008000
C	6.196540000	10.246062000	11.787466000
H	5.234651000	10.202273000	12.320481000
C	6.119631000	1.648597000	9.858934000
H	5.134835000	1.248575000	9.573030000
C	4.073146000	4.050784000	8.484597000
H	3.795212000	3.197917000	9.128957000
H	3.674303000	3.791938000	7.487287000
C	8.775184000	4.060235000	9.877473000
H	8.008829000	4.685203000	10.352507000
H	9.712809000	4.631328000	9.846831000
H	8.939806000	3.214710000	10.553946000
C	3.312822000	5.256071000	9.031808000
H	2.221643000	5.112531000	8.938480000
H	3.548121000	5.350430000	10.095792000
C	8.364503000	7.851548000	5.733937000
H	8.367312000	8.001786000	6.829357000
C	3.319945000	7.698702000	9.144833000
H	2.231965000	7.684788000	9.334697000
H	3.540443000	8.573709000	8.526599000
C	5.095434000	10.687005000	4.403354000
H	5.011212000	10.317711000	3.376076000
H	5.066604000	11.783955000	4.357882000
H	4.198311000	10.364692000	4.943478000
C	9.509326000	8.085293000	11.817740000
H	9.569410000	9.048975000	12.334937000
H	10.492872000	7.898425000	11.366791000
H	9.344978000	7.314919000	12.576253000
C	4.065907000	7.638273000	6.190847000
H	3.773377000	7.507812000	5.133586000
H	3.684639000	8.637962000	6.466506000
C	3.300297000	6.573788000	6.972528000
H	2.210300000	6.738543000	6.904226000
H	3.520859000	5.601598000	6.522973000
C	9.437325000	2.617046000	7.927319000
H	9.494922000	1.696021000	8.517494000

H	10.431224000	3.082679000	7.958021000
H	9.240740000	2.331679000	6.889979000
C	6.674704000	5.964993000	13.034444000
H	5.874559000	5.537413000	12.419836000
H	6.643983000	5.465838000	14.012103000
H	7.625933000	5.693189000	12.562566000
C	6.512153000	7.480983000	13.177231000
H	7.371514000	7.861289000	13.750021000
C	4.103222000	7.832271000	10.449032000
H	3.829689000	8.816428000	10.869012000
H	3.720009000	7.097644000	11.180076000
C	6.515538000	10.831843000	6.483390000
H	5.734760000	10.466422000	7.159923000
H	6.434275000	11.925994000	6.439857000
H	7.479971000	10.595829000	6.947186000
C	6.536315000	2.738581000	5.530121000
H	5.740211000	3.490978000	5.501443000
H	6.480575000	2.159494000	4.598831000
H	7.490668000	3.277407000	5.521093000
C	7.076898000	0.454513000	9.951724000
H	7.186217000	-0.067445000	8.994719000
H	6.720212000	-0.279773000	10.686134000
H	8.078181000	0.764179000	10.273902000
C	5.115805000	0.995888000	6.670957000
H	5.024597000	0.288284000	7.501202000
H	5.098466000	0.410877000	5.741640000
H	4.216884000	1.622234000	6.671714000
C	7.086183000	8.107573000	2.325174000
H	7.189206000	9.198348000	2.336405000
H	6.749628000	7.820994000	1.320025000
H	8.087163000	7.680959000	2.459461000
C	5.941977000	2.332783000	11.217157000
H	6.895579000	2.690156000	11.619526000
H	5.521010000	1.637392000	11.955493000
H	5.275112000	3.197999000	11.148501000
C	5.969673000	11.083300000	10.525977000
H	6.902619000	11.252978000	9.978793000
H	5.560232000	12.070467000	10.778416000
H	5.274904000	10.595970000	9.835207000
C	8.776897000	9.070216000	9.623620000
H	7.990216000	9.167512000	8.865115000
H	9.705496000	8.772599000	9.118387000
H	8.939866000	10.076499000	10.023681000
C	9.423197000	8.813924000	5.181436000
H	10.422546000	8.553238000	5.554147000
H	9.227781000	9.852018000	5.464895000
H	9.466406000	8.773813000	4.087697000
C	8.774466000	6.399618000	5.455483000
H	8.924084000	6.236550000	4.382747000
H	8.017424000	5.671131000	5.771473000
H	9.720852000	6.147097000	5.952272000
C	5.934947000	6.083447000	3.321012000
H	6.887358000	5.556002000	3.437229000
H	5.517914000	5.785426000	2.349827000
H	5.264361000	5.715988000	4.103966000
C	7.201790000	10.916806000	12.731074000

H	7.347425000	10.349699000	13.657077000
H	6.866435000	11.923663000	13.013200000
H	8.183641000	11.031262000	12.256315000
C	5.247549000	7.815859000	13.978176000
H	5.145659000	8.889539000	14.167349000
H	5.261898000	7.313596000	14.954694000
H	4.340075000	7.484775000	13.460807000

[ClCeTREN^{TIPS}] (**2-Cl**)

C	8.616345000	3.380634000	10.409397000
C	8.455698000	2.995231000	8.934731000
C	9.339404000	1.785126000	8.600892000
Si	6.612997000	2.764809000	8.388706000
C	5.558012000	1.722899000	9.656456000
C	5.238789000	2.447820000	10.967072000
N	5.808820000	4.389542000	8.178570000
C	4.418372000	4.228957000	7.720461000
C	3.474891000	5.142494000	8.487646000
N	3.920296000	6.541074000	8.393254000
C	3.502030000	7.170584000	7.130772000
C	4.482051000	8.261403000	6.725981000
N	5.855978000	7.740025000	6.639630000
Si	6.680265000	8.306897000	5.114817000
C	8.521989000	7.710839000	5.107475000
C	8.692930000	6.225513000	4.766597000
C	6.624708000	1.770623000	6.711968000
C	5.282087000	1.185973000	6.259113000
C	6.706198000	10.253355000	4.999012000
C	7.314662000	10.882977000	6.255341000
C	5.648564000	7.667795000	3.588479000
C	5.335380000	6.169177000	3.613480000
Ce	6.564992000	6.498637000	8.371578000
N	5.870039000	7.423849000	10.296997000
C	4.493261000	7.111922000	10.712955000
C	3.514645000	7.328190000	9.568299000
Si	6.730493000	8.441049000	11.543189000
C	6.862563000	7.500385000	13.244876000
C	5.559443000	7.366574000	14.040347000
C	8.533972000	8.806926000	10.944853000
C	8.610235000	9.826765000	9.803032000
C	5.679717000	10.052314000	11.868820000
C	6.363050000	10.983346000	12.877977000
C	5.380311000	10.934057000	4.640639000
C	6.277447000	8.079204000	2.251207000
C	9.493041000	9.200397000	12.076948000
C	7.267781000	2.587309000	5.587604000
C	5.276520000	10.816976000	10.605142000
C	6.173634000	0.343490000	9.923384000
C	9.433725000	8.563257000	4.213257000
C	7.533512000	6.135110000	13.072180000
H	8.828165000	3.839744000	8.341271000
H	7.396073000	10.441330000	4.162751000
H	4.687233000	8.197912000	3.667452000
H	8.894590000	7.851750000	10.542627000
H	7.286810000	0.918944000	6.928772000
H	4.753544000	9.691290000	12.340015000
H	4.601918000	1.550236000	9.139917000
H	4.040742000	3.205041000	7.855730000
H	4.329854000	4.424594000	6.639133000
H	7.986354000	4.228556000	10.696262000
H	9.654281000	3.666818000	10.618486000
H	8.364911000	2.544867000	11.072204000
H	2.434689000	5.038413000	8.134535000

H	3.494156000	4.850566000	9.541527000
H	8.871033000	7.844443000	6.139747000
H	2.480752000	7.082748000	9.864979000
H	3.535280000	8.386395000	9.293362000
H	4.919757000	10.514426000	3.739940000
H	5.538254000	12.004599000	4.454220000
H	4.650457000	10.860797000	5.454108000
H	9.200410000	10.136333000	12.565046000
H	10.503380000	9.348198000	11.674558000
H	9.563866000	8.430189000	12.850651000
H	4.119146000	8.667831000	5.770561000
H	4.421765000	9.101340000	7.436781000
H	2.472737000	7.559974000	7.210316000
H	3.504373000	6.402919000	6.351704000
H	9.004086000	0.873209000	9.107487000
H	10.370856000	1.974579000	8.924579000
H	9.372301000	1.575197000	7.527552000
H	6.901585000	5.464073000	12.479321000
H	7.706037000	5.650788000	14.042241000
H	8.500219000	6.207160000	12.562714000
H	7.536772000	8.136519000	13.837479000
H	4.146930000	7.737191000	11.548923000
H	4.415080000	6.075262000	11.079764000
H	6.665207000	10.728985000	7.124426000
H	7.446024000	11.966047000	6.132715000
H	8.293173000	10.457359000	6.501252000
H	6.647391000	3.452844000	5.328250000
H	7.387306000	1.985597000	4.677055000
H	8.256965000	2.967127000	5.864542000
H	6.376480000	-0.208136000	8.999105000
H	5.500013000	-0.273750000	10.532178000
H	7.117701000	0.425109000	10.473363000
H	4.798624000	0.586406000	7.037964000
H	5.423581000	0.532291000	5.388295000
H	4.578116000	1.968202000	5.955164000
H	6.474937000	9.155145000	2.195991000
H	5.615530000	7.824327000	1.413216000
H	7.227085000	7.560571000	2.078066000
H	6.130175000	2.563344000	11.591331000
H	4.501040000	1.886995000	11.556016000
H	4.835556000	3.449970000	10.790475000
H	6.143177000	11.273179000	10.116899000
H	4.574744000	11.626907000	10.844616000
H	4.796323000	10.165746000	9.868421000
H	7.915617000	9.602255000	8.986505000
H	9.619085000	9.845050000	9.372960000
H	8.384262000	10.840404000	10.153512000
H	10.462965000	8.185314000	4.262015000
H	9.461108000	9.612402000	4.522265000
H	9.129247000	8.533785000	3.160918000
H	8.475592000	6.029810000	3.710461000
H	8.044883000	5.573274000	5.360613000
H	9.725459000	5.907814000	4.955514000
H	6.231900000	5.564835000	3.444452000
H	4.614093000	5.906385000	2.828440000
H	4.912334000	5.860200000	4.574491000

H	6.642429000	10.465602000	13.801740000
H	5.700452000	11.813804000	13.154763000
H	7.273453000	11.425770000	12.458598000
H	5.062712000	8.329518000	14.200744000
H	5.756602000	6.935402000	15.030639000
H	4.845448000	6.701667000	13.542238000
Cl	9.209079000	6.430201000	8.374619000

[BrCeTREN^{TIPS}] (**2-Br**)

C	8.593155000	3.574060000	10.458118000
C	8.502303000	3.049673000	9.021319000
C	9.395475000	1.813107000	8.850187000
Si	6.692246000	2.789825000	8.395171000
C	5.551018000	1.820108000	9.643681000
C	5.222069000	2.575134000	10.934462000
N	5.932970000	4.415470000	8.066460000
C	4.576573000	4.280986000	7.507347000
C	3.573577000	5.106441000	8.300656000
N	3.991256000	6.517350000	8.337102000
C	3.557813000	7.256049000	7.140060000
C	4.557296000	8.356269000	6.814099000
N	5.907438000	7.796940000	6.628807000
Si	6.654754000	8.331711000	5.051817000
C	8.411967000	7.544420000	4.881291000
C	8.401374000	6.034815000	4.621268000
C	6.790873000	1.695027000	6.784649000
C	5.470363000	1.104334000	6.276998000
C	6.880052000	10.265694000	4.995167000
C	7.729589000	10.770326000	6.164326000
C	5.430281000	7.869002000	3.604334000
C	5.048278000	6.386657000	3.543320000
Ce	6.648896000	6.525940000	8.320545000
N	5.940558000	7.337258000	10.288026000
C	4.584970000	6.918346000	10.687537000
C	3.578613000	7.189162000	9.579640000
Si	6.708933000	8.392472000	11.566366000
C	6.796992000	7.483900000	13.288996000
C	5.472361000	7.311858000	14.040289000
C	8.523937000	8.823913000	11.054707000
C	8.621351000	9.854929000	9.924969000
C	5.567916000	9.955044000	11.815241000
C	6.095654000	10.904021000	12.899529000
C	5.590952000	11.086021000	4.872868000
C	5.927511000	8.356413000	2.237079000
C	9.408061000	9.248979000	12.235565000
C	7.526765000	2.413786000	5.650480000
C	5.252265000	10.723695000	10.528790000
C	6.091086000	0.418624000	9.957518000
C	9.307789000	8.252977000	3.855905000
C	7.538470000	6.147889000	13.191850000
H	8.909387000	3.833005000	8.367794000
H	7.452835000	10.424276000	4.069153000
H	4.511986000	8.431647000	3.830478000
H	8.937121000	7.883339000	10.666832000
H	7.417156000	0.849983000	7.108259000
H	4.617299000	9.541717000	12.184892000
H	4.605726000	1.686973000	9.095706000
H	4.212633000	3.243580000	7.511748000
H	4.554452000	4.586376000	6.448931000
H	7.942958000	4.436757000	10.638839000
H	9.617349000	3.893008000	10.685274000
H	8.318947000	2.800964000	11.184976000
H	2.553402000	5.010929000	7.892198000

H	3.552131000	4.729147000	9.326822000
H	8.873523000	7.696379000	5.866390000
H	2.560102000	6.881929000	9.871451000
H	3.555413000	8.266612000	9.394168000
H	4.955364000	10.754285000	4.044705000
H	5.823923000	12.144840000	4.699226000
H	4.995091000	11.040435000	5.790964000
H	9.043536000	10.162011000	12.719139000
H	10.426929000	9.454662000	11.883816000
H	9.482702000	8.473757000	13.003670000
H	4.183500000	8.877909000	5.921905000
H	4.547208000	9.115603000	7.612373000
H	2.538341000	7.655116000	7.274439000
H	3.530036000	6.558694000	6.298523000
H	9.039581000	0.959938000	9.438733000
H	10.414288000	2.037682000	9.190498000
H	9.467740000	1.490243000	7.807410000
H	6.958782000	5.421142000	12.612450000
H	7.705125000	5.714240000	14.186552000
H	8.515264000	6.245849000	12.706811000
H	7.415426000	8.167513000	13.889850000
H	4.220961000	7.444334000	11.581413000
H	4.563893000	5.850281000	10.956931000
H	7.195854000	10.646628000	7.112898000
H	7.959408000	11.838341000	6.055760000
H	8.680505000	10.234590000	6.252600000
H	6.937960000	3.260309000	5.281164000
H	7.699740000	1.740204000	4.800950000
H	8.499505000	2.806289000	5.964720000
H	6.302903000	-0.162376000	9.053905000
H	5.368114000	-0.153729000	10.553427000
H	7.016958000	0.470111000	10.541386000
H	4.899170000	0.598819000	7.063122000
H	5.659881000	0.365268000	5.487458000
H	4.826070000	1.873766000	5.837690000
H	6.169407000	9.424296000	2.233630000
H	5.164971000	8.191277000	1.464602000
H	6.824900000	7.813054000	1.921502000
H	6.095037000	2.651970000	11.589984000
H	4.435618000	2.059510000	11.501207000
H	4.877375000	3.594195000	10.734034000
H	6.128318000	11.262229000	10.155001000
H	4.463208000	11.467686000	10.700248000
H	4.916851000	10.057264000	9.728281000
H	7.978469000	9.613334000	9.072138000
H	9.648094000	9.910108000	9.544354000
H	8.344111000	10.856857000	10.272091000
H	10.307693000	7.800876000	3.858203000
H	9.435068000	9.317328000	4.074650000
H	8.919133000	8.167276000	2.834906000
H	8.077808000	5.805864000	3.599592000
H	7.739573000	5.489068000	5.302194000
H	9.407345000	5.617374000	4.748477000
H	5.886114000	5.770272000	3.203833000
H	4.220317000	6.223492000	2.840914000
H	4.739703000	6.000448000	4.519846000

H	6.292415000	10.393007000	13.848032000
H	5.370857000	11.703148000	13.103081000
H	7.026699000	11.389482000	12.586527000
H	4.911403000	8.248543000	14.125999000
H	5.654975000	6.948990000	15.060305000
H	4.822158000	6.575431000	13.555890000
Br	9.475516000	6.551116000	8.290214000

[FCeTREN^{TIPS}] (**2-F**)

C	8.787231000	3.754680000	10.157033000
C	8.499748000	3.322111000	8.714008000
C	9.489715000	2.232459000	8.279669000
Si	6.645085000	2.838686000	8.409054000
C	5.893215000	1.723503000	9.819038000
C	5.588483000	2.477183000	11.116672000
N	5.665506000	4.359337000	8.290126000
C	4.243888000	4.096395000	8.045447000
C	3.357226000	5.138463000	8.716531000
N	3.774945000	6.503875000	8.361859000
C	3.343159000	6.886085000	7.008344000
C	4.229611000	7.986057000	6.436810000
N	5.649843000	7.629469000	6.515457000
Si	6.608157000	8.286233000	5.122683000
C	8.458943000	7.739110000	5.323396000
C	8.709794000	6.271502000	4.954999000
C	6.566984000	1.795371000	6.764815000
C	5.244535000	1.061172000	6.517332000
C	6.572649000	10.233900000	5.085000000
C	7.020882000	10.820072000	6.426509000
C	5.795691000	7.675845000	3.459555000
C	5.461852000	6.181569000	3.438461000
Ce	6.394044000	6.502940000	8.334063000
N	5.687148000	7.521740000	10.230892000
C	4.270673000	7.432481000	10.598742000
C	3.367195000	7.490786000	9.373644000
Si	6.674133000	8.377844000	11.485987000
C	6.598759000	7.483673000	13.215147000
C	5.280030000	7.648129000	13.979857000
C	8.526436000	8.393794000	10.907622000
C	8.811837000	9.433754000	9.817367000
C	5.927995000	10.157210000	11.762679000
C	6.769207000	10.999801000	12.728984000
C	5.245032000	10.855181000	4.636891000
C	6.614903000	8.081496000	2.228177000
C	9.523784000	8.551864000	12.063821000
C	6.955386000	2.640280000	5.548029000
C	5.620042000	10.923269000	10.473025000
C	6.731630000	0.466917000	10.083823000
C	9.444938000	8.648322000	4.576863000
C	6.978124000	6.004381000	13.101342000
H	8.683383000	4.197703000	8.077789000
H	7.326434000	10.502906000	4.330120000
H	4.842543000	8.221883000	3.402459000
H	8.705696000	7.406999000	10.460082000
H	7.341793000	1.026144000	6.900396000
H	4.965076000	9.969186000	12.260844000
H	4.929451000	1.383505000	9.411488000
H	3.916162000	3.118589000	8.433635000
H	4.022971000	4.062010000	6.965243000
H	8.086570000	4.513777000	10.519628000
H	9.796791000	4.175552000	10.239893000
H	8.728218000	2.905823000	10.847508000
H	2.294132000	4.977512000	8.466178000

H	3.458532000	5.029154000	9.800273000
H	8.677384000	7.835946000	6.394740000
H	2.308368000	7.349599000	9.652760000
H	3.458027000	8.484513000	8.925876000
H	4.920474000	10.489362000	3.657138000
H	5.336673000	11.946819000	4.560958000
H	4.438729000	10.652926000	5.350266000
H	9.384549000	9.495678000	12.602705000
H	10.551883000	8.549511000	11.679177000
H	9.451131000	7.740392000	12.794377000
H	3.885120000	8.153151000	5.403734000
H	4.025640000	8.935619000	6.959740000
H	2.281657000	7.189587000	7.008345000
H	3.432264000	6.004715000	6.366760000
H	9.348281000	1.300357000	8.837903000
H	10.520534000	2.563593000	8.460228000
H	9.408413000	1.994753000	7.214724000
H	6.244226000	5.454581000	12.501094000
H	7.017556000	5.526628000	14.089203000
H	7.956325000	5.863523000	12.629188000
H	7.378775000	7.986619000	13.806508000
H	3.946598000	8.254664000	11.256819000
H	4.062251000	6.511542000	11.169442000
H	6.323693000	10.546899000	7.226970000
H	7.064847000	11.916517000	6.388232000
H	8.013044000	10.462368000	6.722253000
H	6.235007000	3.449708000	5.383889000
H	6.981279000	2.032874000	4.633700000
H	7.942409000	3.100977000	5.662509000
H	6.927569000	-0.103249000	9.169002000
H	6.219678000	-0.205308000	10.784918000
H	7.700508000	0.717460000	10.530494000
H	4.967011000	0.405859000	7.349427000
H	5.315997000	0.432330000	5.619918000
H	4.415398000	1.757542000	6.352095000
H	6.826159000	9.156250000	2.203569000
H	6.079874000	7.831989000	1.302410000
H	7.575297000	7.555134000	2.195183000
H	6.504339000	2.776848000	11.635600000
H	5.013323000	1.849137000	11.809834000
H	5.011121000	3.388121000	10.928234000
H	6.533978000	11.229983000	9.954575000
H	5.046804000	11.835362000	10.685980000
H	5.037569000	10.316252000	9.772289000
H	8.106818000	9.376248000	8.982271000
H	9.818125000	9.293091000	9.404199000
H	8.760547000	10.453626000	10.214938000
H	10.474533000	8.305467000	4.741707000
H	9.392531000	9.687744000	4.914263000
H	9.273184000	8.642922000	3.494812000
H	8.625378000	6.108342000	3.874508000
H	8.008957000	5.585818000	5.441576000
H	9.721178000	5.967849000	5.252287000
H	6.367133000	5.567266000	3.411620000
H	4.867736000	5.924140000	2.551612000
H	4.893359000	5.880319000	4.324078000

H	6.963137000	10.479620000	13.673457000
H	6.260521000	11.941832000	12.972574000
H	7.739208000	11.263117000	12.292727000
H	5.002752000	8.698463000	14.117704000
H	5.356099000	7.198946000	14.979059000
H	4.448233000	7.149728000	13.470265000
F	8.477730000	6.506144000	8.282968000

[CeTREN^{TIPS}] PhCl (**1** + C_6H_5Cl)

C	8.357565000	3.768309000	9.845515000
C	7.914455000	3.199908000	8.491727000
C	8.883178000	2.101196000	8.037505000
Si	6.032140000	2.683874000	8.446186000
C	5.490765000	1.602845000	9.975696000
C	5.367205000	2.390512000	11.282600000
N	5.138787000	4.218618000	8.392346000
C	3.685036000	4.103560000	8.405035000
C	3.042751000	5.338627000	9.032246000
N	3.567673000	6.587199000	8.451442000
C	3.049812000	6.818180000	7.090828000
C	3.915744000	7.803647000	6.309828000
N	5.325517000	7.438298000	6.387492000
Si	6.329461000	7.945415000	5.012088000
C	8.110831000	7.254104000	5.416598000
C	8.226386000	5.734138000	5.249795000
C	5.779407000	1.563274000	6.872796000
C	4.404154000	0.887500000	6.812325000
C	6.496339000	9.876397000	4.813916000
C	6.948404000	10.532545000	6.121268000
C	5.598070000	7.290097000	3.329218000
C	5.147314000	5.827773000	3.374097000
Ce	6.137475000	6.315275000	8.256495000
N	5.627810000	7.528791000	10.177018000
C	4.234475000	7.728484000	10.558442000
C	3.328385000	7.745789000	9.330553000
Si	6.832959000	8.224781000	11.282262000
C	6.843742000	7.410892000	13.054534000
C	5.659491000	7.833858000	13.932626000
C	8.562036000	7.859553000	10.450455000
C	8.877814000	8.789594000	9.272690000
C	6.505962000	10.123719000	11.570266000
C	7.594944000	10.803356000	12.408600000
C	5.230731000	10.554989000	4.277499000
C	6.510116000	7.560253000	2.127188000
C	9.738222000	7.838213000	11.434775000
C	6.055055000	2.343390000	5.584686000
C	6.209301000	10.910581000	10.290776000
C	6.348716000	0.345312000	10.157353000
C	9.249939000	7.954504000	4.665778000
C	6.930266000	5.885296000	12.967310000
H	7.998705000	4.004832000	7.738622000
H	7.290248000	10.024110000	4.066276000
H	4.692190000	7.899331000	3.188954000
H	8.476499000	6.828808000	10.059243000
H	6.532975000	0.765700000	6.955388000
H	5.584698000	10.133122000	12.172366000
H	4.477709000	1.264900000	9.709356000
H	3.318829000	3.241824000	8.990907000
H	3.272198000	3.950249000	7.391714000
H	7.657095000	4.507104000	10.255475000
H	9.349307000	4.235725000	9.780020000
H	8.428701000	2.973972000	10.596292000
H	1.942331000	5.302273000	8.943595000

H	3.288937000	5.345766000	10.097945000
H	8.262273000	7.495168000	6.485287000
H	2.264195000	7.797923000	9.622160000
H	3.559455000	8.645011000	8.752037000
H	4.924098000	10.156073000	3.305110000
H	5.391522000	11.633935000	4.150375000
H	4.385902000	10.435687000	4.964822000
H	9.868060000	8.812134000	11.919413000
H	10.677798000	7.607960000	10.916037000
H	9.605252000	7.093299000	12.224622000
H	3.518070000	7.804071000	5.279350000
H	3.730648000	8.827257000	6.682717000
H	1.999397000	7.157729000	7.129424000
H	3.067761000	5.858334000	6.566650000
H	8.837288000	1.230145000	8.699930000
H	9.919585000	2.463690000	8.049685000
H	8.668733000	1.754139000	7.022810000
H	6.067003000	5.473600000	12.432552000
H	6.951450000	5.427517000	13.965394000
H	7.830162000	5.552059000	12.438453000
H	7.760306000	7.777210000	13.540675000
H	4.054446000	8.685581000	11.079297000
H	3.882221000	6.954432000	11.263636000
H	6.218874000	10.360515000	6.920858000
H	7.063398000	11.618536000	6.005333000
H	7.910055000	10.139034000	6.469310000
H	5.369456000	3.192649000	5.484974000
H	5.927898000	1.708487000	4.697696000
H	7.075158000	2.742659000	5.554815000
H	6.418727000	-0.249304000	9.239792000
H	5.933403000	-0.306179000	10.937794000
H	7.370344000	0.597272000	10.465163000
H	4.209731000	0.260037000	7.688271000
H	4.325941000	0.243029000	5.926527000
H	3.594377000	1.622750000	6.746429000
H	6.808211000	8.612261000	2.057839000
H	6.008902000	7.299583000	1.185557000
H	7.426684000	6.960517000	2.175550000
H	6.346359000	2.697755000	11.664489000
H	4.891687000	1.783654000	12.064659000
H	4.771336000	3.299209000	11.150938000
H	7.106734000	11.039128000	9.676960000
H	5.831962000	11.915048000	10.524438000
H	5.463261000	10.404442000	9.669997000
H	8.042408000	8.893274000	8.569684000
H	9.756472000	8.442656000	8.713253000
H	9.104144000	9.800705000	9.628033000
H	10.224669000	7.556228000	4.976501000
H	9.262617000	9.033466000	4.844916000
H	9.167503000	7.800726000	3.584426000
H	8.221415000	5.457780000	4.189851000
H	7.393206000	5.183861000	5.705640000
H	9.163892000	5.352375000	5.675923000
H	5.998223000	5.142640000	3.449351000
H	4.595415000	5.558104000	2.463741000
H	4.497775000	5.632945000	4.233183000

H	7.786009000	10.277101000	13.350439000
H	7.312409000	11.834051000	12.661352000
H	8.545168000	10.859076000	11.864402000
H	5.610853000	8.918520000	14.074335000
H	5.732187000	7.378447000	14.929365000
H	4.703282000	7.514140000	13.503465000
Cl	13.096093000	6.221640000	7.707955000
C	13.758846000	7.662937000	8.464507000
C	14.153737000	7.608507000	9.798224000
C	14.676177000	8.757100000	10.391186000
C	14.800384000	9.936789000	9.658860000
C	14.400136000	9.969152000	8.323776000
C	13.875228000	8.830046000	7.715161000
H	14.054978000	6.684594000	10.357658000
H	14.986364000	8.724192000	11.431715000
H	15.208197000	10.827803000	10.126772000
H	14.494526000	10.884630000	7.746698000
H	13.561215000	8.843432000	6.677157000

X. References

(1) Wojdyr, M. (2010). *Fityk*: A General-Purpose Peak Fitting Program. J Appl Crystallogr 43 (5), 1126–1128. https://doi.org/10.1107/S0021889810030499.

(2) Siano, D. B.; Metzler, D. E. (1969). Band Shapes of the Electronic Spectra of Complex Molecules. The Journal of Chemical Physics 51 (5), 1856–1861. https://doi.org/10.1063/1.1672270.

(3) Chibisov, A. K. (1981). Electron Transfer in Photochemical Reactions. Russ. Chem. Rev. 50 (7), 615–629. https://doi.org/10.1070/RC1981v050n07ABEH002656.

(4) Braslavsky, S. E. (2007). Glossary of Terms Used in Photochemistry, 3rd Edition (IUPAC Recommendations 2006). Pure and Applied Chemistry 79 (3), 293–465. https://doi.org/10.1351/pac200779030293.

(5) Kavarnos, G. J. Fundamental Concepts of Photoinduced Electron Transfer. In *Photoinduced Electron Transfer I*; Mattay, J., Ed.; Dewar, M. J. S., Dunitz, J. D., Hafner, K., Itô, S., Lehn, J.-M., Niedenzu, K., Raymond, K. N., Rees, C. W., Vögtle, F., Series Eds.; Topics in Current Chemistry; Springer Berlin Heidelberg, (1990).; Vol. 156, pp 21–58. https://doi.org/10.1007/3-540-52379-0_2.

(6) Rehm, D.; Weller, A. (1970). Kinetics of Fluorescence Quenching by Electron and H-Atom Transfer. Isr. J. Chem. 8 (2), 259–271. https://doi.org/10.1002/ijch.197000029.

(7) Koopmans, T. (1934). Über die Zuordnung von Wellenfunktionen und Eigenwerten zu den Einzelnen Elektronen Eines Atoms. Physica 1 (1–6), 104–113. https://doi.org/10.1016/S0031-8914(34)90011-2.

(8) Washington University. https://depts.washington.edu/eooptic/linkfiles/dielectric_chart%5B1%5D.pdf.

(9) Zhang, J.; Yang, J.-D.; Cheng, J.-P. (2020). Exploiting the Radical Reactivity of Diazaphosphinanes in Hydrodehalogenations and Cascade Cyclizations. Chem. Sci. 11 (18), 4786–4790. https://doi.org/10.1039/D0SC01352H.

(10) Gaussian 09, Revision A.02, M. J. Frisch, G. W. Trucks, H. B. Schlegel, G. E. Scuseria, M. A. Robb, J. R. Cheeseman, G. Scalmani, V. Barone, G. A. Petersson, H. Nakatsuji, X. Li, M. Caricato, A. Marenich, J. Bloino, B. G. Janesko, R. Gomperts, B. Mennucci, H. P. Hratchian, J. V. Ortiz, A. F. Izmaylov, J. L. Sonnenberg, D. Williams-Young, F. Ding, F. Lipparini, F. Egidi, J. Goings, B. Peng, A. Petrone, T. Henderson, D. Ranasinghe, V. G. Zakrzewski, J. Gao, N. Rega, G. Zheng, W. Liang, M. Hada, M. Ehara, K. Toyota, R. Fukuda, J. Hasegawa, M. Ishida, T. Nakajima, Y. Honda, O. Kitao, H. Nakai, T. Vreven, K. Throssell, J. A. Montgomery, Jr., J. E. Peralta, F. Ogliaro, M. Bearpark, J. J. Heyd, E. Brothers, K. N. Kudin, V. N. Staroverov, T. Keith, R. Kobayashi, J. Normand, K. Raghavachari, A. Rendell, J. C. Burant, S. S. Iyengar, J. Tomasi, M. Cossi, J. M. Millam, M. Klene, C. Adamo, R. Cammi, J. W. Ochterski, R. L. Martin, K. Morokuma, O. Farkas, J. B. Foresman, and D. J. Fox, Gaussian, Inc., Wallingford CT, 2016.

(11) Becke, A. D. (1993). Density-Functional Thermochemistry. III. The Role of Exact Exchange. The Journal of Chemical Physics 98 (7), 5648–5652. https://doi.org/10.1063/1.464913.

(12) *Electronic Density Functional Theory: Recent Progress and New Directions*; Dobson, J. F., Vignale, G., Das, M. P., Eds.; Plenum Press, (1998).

(13) Cao, X.; Dolg, M.; Stoll, H. (2003). Valence Basis Sets for Relativistic Energy-Consistent Small-Core Actinide Pseudopotentials. The Journal of Chemical Physics 118 (2), 487–496. https://doi.org/10.1063/1.1521431.

(14) Küchle, W.; Dolg, M.; Stoll, H.; Preuss, H. (1994). Energy-Adjusted Pseudopotentials for the Actinides. Parameter Sets and Test Calculations for Thorium and Thorium Monoxide. The Journal of Chemical Physics 100 (10), 7535–7542. https://doi.org/10.1063/1.466847.

(15) Maron, L.; Teichteil, C. (1998). On the Accuracy of Averaged Relativistic Shape-Consistent Pseudopotentials. Chemical Physics 237 (1–2), 105–122. https://doi.org/10.1016/S0301-0104(98)00243-2.

(16) Hariharan, P. C.; Pople, J. A. (1973). The Influence of Polarization Functions on Molecular Orbital Hydrogenation Energies. Theoret. Chim. Acta 28 (3), 213–222. https://doi.org/10.1007/BF00533485.

(17) Hehre, W. J.; Ditchfield, R.; Pople, J. A. (1972). Self—Consistent Molecular Orbital Methods. XII. Further Extensions of Gaussian—Type Basis Sets for Use in Molecular Orbital Studies of Organic Molecules. The Journal of Chemical Physics 56 (5), 2257–2261. https://doi.org/10.1063/1.1677527.

(18) Dolomanov, O. V.; Bourhis, L. J.; Gildea, R. J.; Howard, J. A. K.; Puschmann, H. (2009). *OLEX2*: A Complete Structure Solution, Refinement and Analysis Program. J Appl Crystallogr 42 (2), 339–341. https://doi.org/10.1107/S0021889808042726.

(19) Sheldrick, G. M. (2015). Crystal Structure Refinement with *SHELXL*. Acta Crystallogr C Struct Chem 71 (1), 3–8. https://doi.org/10.1107/S2053229614024218.
(20) Sheldrick, G. M. (2015). *SHELXT* – Integrated Space-Group and Crystal-Structure Determination. Acta Crystallogr A Found Adv 71 (1), 3–8. https://doi.org/10.1107/S2053273314026370.
(21) King, D. M.; Tuna, F.; McInnes, E. J. L.; McMaster, J.; Lewis, W.; Blake, A. J.; Liddle, S. T. (2012). Synthesis and Structure of a Terminal Uranium Nitride Complex. Science 337 (6095), 717–720. https://doi.org/10.1126/science.1223488.
(22) Izod, K.; Liddle, S. T.; Clegg, W. (2004). A Convenient Route to Lanthanide Triiodide THF Solvates. Crystal Structures of $LnI_3(THF)_4$ [Ln = Pr] and LnI_3 $(THF)_{3.5}$ [Ln = Nd, Gd, Y]. Inorg. Chem. 43 (1), 214–218. https://doi.org/10.1021/ic034851u.
(23) Chang, R. K.; Clairmont, B. P.; Lin, S.; MacArthur, A. H. R. (2019). Amidation of Aryl Chlorides Using a Microwave-Assisted, Copper-Catalyzed Concurrent Tandem Catalytic Methodology. Organometallics 38 (22), 4448–4454. https://doi.org/10.1021/acs.organomet.9b00561.
